# Data-Driven Discovery of Molecular Targets for Antibody-Drug Conjugates in Cancer Treatment

**DOI:** 10.1155/2021/2670573

**Published:** 2021-01-02

**Authors:** Abolfazl Razzaghdoust, Shahabedin Rahmatizadeh, Bahram Mofid, Samad Muhammadnejad, Mahmoud Parvin, Peyman Mohammadi Torbati, Abbas Basiri

**Affiliations:** ^1^Urology and Nephrology Research Center, Shahid Beheshti University of Medical Sciences, Tehran, Iran; ^2^Department of Health Information Technology and Management, School of Allied Medical Sciences, Shahid Beheshti University of Medical Sciences, Tehran, Iran; ^3^Department of Oncology, Shohada-e-Tajrish Medical Center, School of Medicine, Shahid Beheshti University of Medical Sciences, Tehran, Iran; ^4^Gene Therapy Research Center, Digestive Diseases Research Institute, Tehran University of Medical Sciences, Tehran, Iran; ^5^Department of Pathology, Labbafinejad Hospital, Shahid Beheshti University of Medical Sciences, Tehran, Iran

## Abstract

Antibody-drug conjugate therapy has attracted considerable attention in recent years. Since the selection of appropriate targets is a critical aspect of antibody-drug conjugate research and development, a big data research for discovery of candidate targets per tumor type is outstanding and of high interest. Thus, the purpose of this study was to identify and prioritize candidate antibody-drug conjugate targets with translational potential across common types of cancer by mining the Human Protein Atlas, as a unique big data resource. To perform a multifaceted screening process, XML and TSV files including immunohistochemistry expression data for 45 normal tissues and 20 tumor types were downloaded from the Human Protein Atlas website. For genes without high protein expression across critical normal tissues, a quasi *H*-score (range, 0-300) was computed per tumor type. All genes with a quasi *H* − score ≥ 150 were extracted. Of these, genes with cell surface localization were selected and included in a multilevel validation process. Among 19670 genes that encode proteins, 5520 membrane protein-coding genes were included in this study. During a multistep data mining procedure, 332 potential targets were identified based on the level of the protein expression across critical normal tissues and 20 tumor types. After validation, 23 cell surface proteins were identified and prioritized as candidate antibody-drug conjugate targets of which two have interestingly been approved by the FDA for use in solid tumors, one has been approved for lymphoma, and four have currently been entered in clinical trials. In conclusion, we identified and prioritized several candidate targets with translational potential, which may yield new clinically effective and safe antibody-drug conjugates. This large-scale antibody-based proteomic study allows us to go beyond the RNA-seq studies, facilitates bench-to-clinic research of targeted anticancer therapeutics, and offers valuable insights into the development of new antibody-drug conjugates.

## 1. Introduction

Much recent interest has centred around research on antibody-drug conjugate (ADC) therapy as a promising targeted therapy for cancer [1–3]. The targeted delivery of highly potent cytotoxic agents to tumor cells makes possible the ADC therapy as an attractive choice of cancer treatment. However, despite considerable advances in the field, only few ADCs have been currently approved by the FDA owing to the lack of enough tumor response or excessive normal tissue toxicity observed in clinical trials [3]. Therefore, the expansion of effective and nontoxic ADCs is still a challenge for scientists and drug developers, and the discovery of novel ADC targets is of high interest.

The selection of appropriate target antigens is the first critical step of developing safe and effective ADCs [2, 4, 5]. There are only few large-scale studies which have identified or prioritized ADC targets. In an mRNA-level study, Fauteux et al. [6] aimed to identify and prioritize candidate ADC targets for breast cancer. Furthermore, in a data-driven prioritization study, only clinically relevant ADC targets were prioritized across different tumor (sub) types, using transcript-level evidence [7]. To the best of our knowledge, no big data research based on the protein-level evidence still exists for identification and prioritization of candidate ADC targets across a wide range of tumor types.

The Human Protein Atlas (HPA), a large-scale antibody-based proteomic resource, provides a unique opportunity to perform systematic discovery and validation of targets for different tumor types at the protein level [8–10]. The HPA has combined the antibody-based approach with transcriptomic data for an overview of global expression profiles [11]. Thus, in this study, we aimed to identify and prioritize candidate ADC targets per common tumor types by mining of the HPA database, as the unique big data recourse.

## 2. Materials and Methods

### 2.1. Discovery Approach

To systematically identify and prioritize candidate ADC targets across 20 tumor types, the following screening approach was applied:

1. Among data from 19670 genes encoding human proteins, the expression data for 5520 membrane protein-coding genes were downloaded from the HPA website (version 19) as an XML file (http://www.proteinatlas.org/search/protein_class%3APredicted+membrane+proteins)
2. A total of 2131 genes without the protein level evidence were excluded from the study, and remaining 3389 genes were monitored for their protein expression in the critical normal tissues
3. The protein-coding genes that showed the high protein expression (*n* = 1735) in one or more critical normal tissues including lung, gastrointestinal tract (i.e., oral mucosa, esophagus, stomach, duodenum, small intestine, colon, and rectum), liver, kidney, heart muscle, skin, and bone marrow were excluded, and remaining 1654 genes were retained in the data mining process
4. In the next step, the protein expression levels for remaining genes were monitored across 20 tumor types based on data extracted from the pathology TSV file, downloadable from the HPA website (http://www.proteinatlas.org/about/download). After calculating a quasi *H*-score (range, 0-300) per tumor type, as a proxy for the protein expression, 745 genes with a score ≥ 150 for at least one tumor type were included in the next step
5. In order to discriminate the target antigens localized on the cell surface from nonsurface membrane proteins, a file containing predicted set of human surfaceome (Supplementary Table S1), recently identified by Bausch-Fluck et al. [12], was downloaded from a public resource (http://wlab.ethz.ch/surfaceome). Consequently, a number of 332 potential target genes encoding surface proteins were extracted and included in an HPA-based validation process
6. After the three-step nonexperimental validation process, 23 candidate ADC targets were identified and prioritized across 20 tumor types

**Figure 1:**
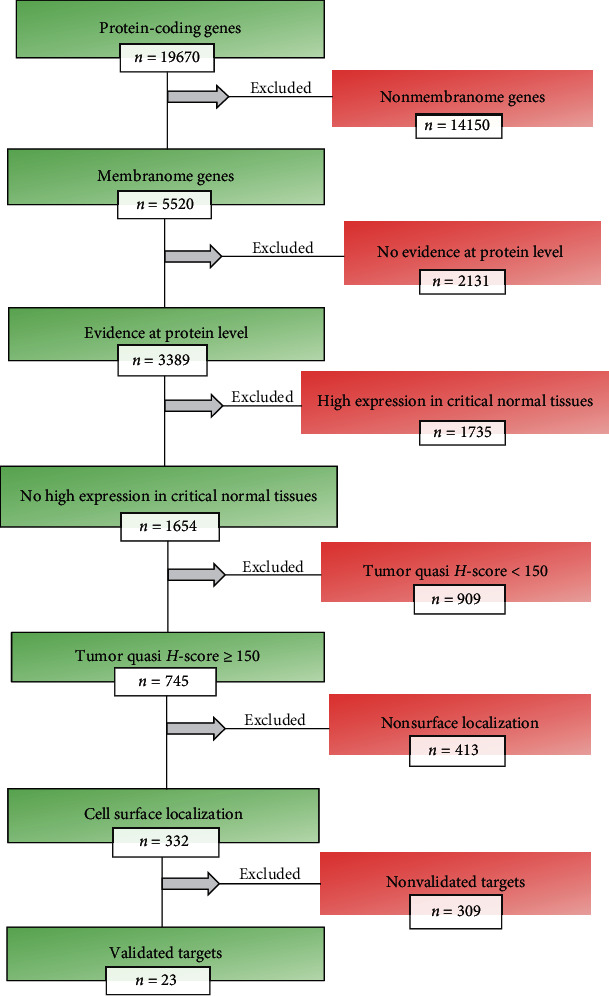
Methodology workflow.

The C# programming language was used to extract and summarize the required data from the XML and TSV files during the screening process. The workflow methodology of target discovery and validation is indicated in Figure 1. The study methodologies were approved by the ethics committee of Shahid Beheshti University of Medical Sciences. The informed consent requirement was waived. All procedures performed in the study involving human samples were in accordance with the 1964 Helsinki Declaration and its later amendments.

### 2.2. HPA-Based Validation Approach

A total of 332 potential targets were selected to be included in an extensive validation process for their level of expression based on the HPA database. We combined data at the protein level with transcript data to validate identified potential targets. Since the supportive evidence for the protein expression is often sought by mRNA profiling [13], the consistency between immunohistochemistry (IHC) data and RNA-seq data, retrieved from the HPA, was used as the first step of the validation. In addition to RNA consistency, literature conformity and verification of membrane localization were considered for expression validation of potential targets. Notably, the confirmation of membrane localization was previously stated as a critical step of target validation [13]. The IHC images and description of the staining pattern (available at the HPA database) for each antibody in cancer tissues were considered for verification of membrane localization. A predominant membranous staining in IHC samples was required for each potential target to pass the validation process. Only potential targets that passed three HPA-based validation steps were considered as candidate targets. Detailed methods regarding the strategy of antibody validation, accessible at the HPA website (http://www.proteinatlas.org), are provided in Supplementary Methods S1. Finally, for further comparison and validation of our quasi *H*-score, as a new method to discover ADC targets, we have investigated the correlation of target *H*-scores for different tumor types with corresponding FPKM (fragments per kilobase of transcript, per Million mapped reads) values of the TCGA (*The Cancer Genome Atlas*) datasets extracted from the HPA (http://www.proteinatlas.org).

### 2.3. Experimental Validation Approach

In addition to the HPA-based validation, a restricted experimental validation was applied. In order to logistical limitations, such as dataset accessibility for 20 tumor types and also 23 antibodies required for IHC examinations, only protein expressions of NECTIN4 and ERBB2, as two FDA-approved ADC targets for solid tumors, were experimentally validated in tissue microarray (TMA) samples of patients with urothelial carcinoma by using the IHC technique.

### 2.4. TMA Construction

The TMAs were assembled as previously described for the HPA project [14]. All hematoxylin and eosin- (H&E-) stained slides were reviewed by a pathologist with subspecialty expertise in urologic pathology to determine the best area for preparing the TMA of each sample. Tissue arrays were constructed by placing 1 mm diameter cores in recipient paraffin blocks. From each tumor, three tissue cores were extracted to account for intratumoral heterogeneity. First, reference histological slides with the specific area marked by the pathologist were aligned with the respective donor block. Second, three cores were extracted per tumor and assembled in recipient paraffin blocks using a tissue arrayer (Galileo TMA CK3500 Tissue Micro arrayer; ISETMA Software, Integrated System Engineering, Milan, Italy). Then, consecutive sections (with a thickness of 3 *μ*m) were cut from each TMA block, mounted on microscope slides, and immunohistochemically assayed (Supplementary Figure S1).

### 2.5. Immunohistochemistry

IHC was performed on the TMA slides with a standard technique as previously defined with some modifications [14, 15]. Briefly, tissue slices were deparaffinized at 55°C for 10 minutes, cleared in xylene, and were then rehydrated by incubating in solutions with decreasing alcohol content. Antigen retrieval was conducted by boiling the samples in Tris-EDTA buffer (pH 9.0) for 34 minutes in a standard microwave. The endogenous peroxidase was blocked with 3% H_2_O_2_ for 10 minutes. Samples were immunostained at 4°C in blocking solution with primary antibodies, anti-PVRL4/NECTIN4 (1 : 200 dilutions; HPA010775, Sigma-Aldrich, USA) and anti–ERBB2/HER2 (ready to use; MAD-000308QD, master diagnostica, Spain). After washing with PBS (3 times/5 min), the sections were incubated with appropriate secondary antibody (anti-rabbit) for 45 minutes. Then, the TMA slides were visualized with 3,3′-diaminobenzidine (DAB) substrate as chromogen for 10 minutes at the room temperature. The sections were counterstained with haematoxylin, dehydrated in alcohol, cleared with xylene, and mounted for examination. All tumors and normal tissues were scored by a pathologist.

### 2.6. Statistical Analysis

In the experimental validation process, the mean *H*-scores obtained for two selected proteins were compared with their HPA-derived quasi *H*-scores using one-sample *t*-test. The lack of statistically significant differences might be considered as the experimental validation of HPA-derived quasi *H*-scores for the two FDA-approved targets. Moreover, the correlation of target *H*-scores with corresponding FPKM values was assessed by Pearson correlation coefficient for 17 tumor types with available FPKM data on the HPA website. Data were analyzed using the IBM SPSS Statistics for Windows, V.23.0 (IBM Corp., Armonk, NY, USA). A *p* value of <0.05 was considered as statistically significant.

### 2.7. *H*-Score Calculation

The *H*-score is a semiquantitative scoring system that calculates an IHC expression score from 0 to 300 according to both the intensity of staining (0, not detected; 1, low; 2, moderate; 3, high) and the percentage of cells stained. *H*-scores reflecting the protein expression were calculated by multiplying the intensity in the percentage of the positive cells. In this study, the average of three cores per tumor resulted in a final *H*-score for each tumor ranging from 0 to 300. A score of 0 indicates no expression, and a score of 300 indicates the maximum possible expression.

### 2.8. Quasi *H*-Score Calculation

The percentage of samples with low, medium, and high protein expression for the HPA datasets was determined (only for genes without high protein expression across the critical normal tissues), and the quasi *H*-score was calculated per tumor type based on the following formula (1):

Quasi *H* − score (range, 0 − 300) = (1 × percentage of patients with low protein expression) + (2 × percentage of patients with medium protein expression) + (3 × percentage of patients with high protein expression)(1)

All surface protein-coding genes with a quasi *H*-score above the threshold (≥150) in at least one tumor type were considered as potential ADC targets. The cut − off ≥ 150 was defined based on the previous studies utilizing the *H*-score [16–18].

## 3. Results

### 3.1. Identified Candidate Targets

As shown in Figure 1, among 19670 protein-coding genes, only membranome genes (*n* = 5520) were included in our study. The IHC-based expression data for 3389 membrane protein-coding genes were available in the HPA. Among these, 1735 genes with high protein expression across 13 critical normal tissues were excluded. Then, a quasi *H*-score was calculated for remaining 1654 genes. The list of 745 genes with a quasi *H* − score ≥ 150 and their protein expression profile are listed in Supplementary Table S2. As the goal of this study was to identify cell surface proteins which were differentially overexpressed in common cancers, only 332 predicted surface proteins with a quasi *H* − score ≥ 150 were considered as potential ADC targets. At the end, only 23 final targets passed the entire validation process including the RNA consistency, literature conformity, and verification of the membrane localization. The list of 332 potential targets and their validation status are indicated in Supplementary Table S3.

Among 20 tumor types, the largest number of candidate targets (*n* = 8) was interestingly identified for pancreatic cancer, with currently lack of effective treatment options. At least one candidate target (up to six) was also identified for remaining cancer types.

### 3.2. Prioritized Targets Based on the Expression in Tumor Types

**Figure 2:**
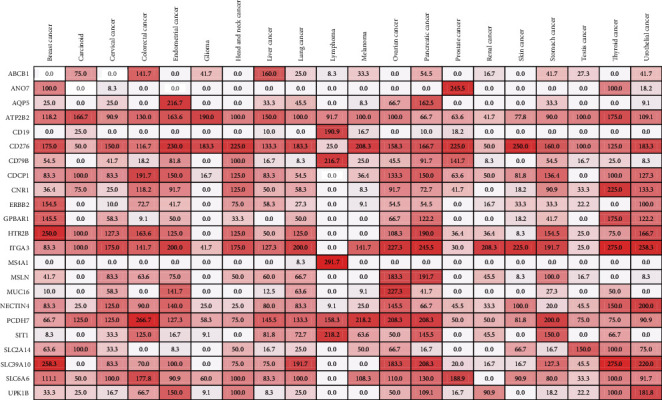
A heat map depicting the quasi *H*-score for candidate ADC targets across 20 tumor types.

The quasi *H*-score, as a proxy of the tumor overexpression, for 23 candidate ADC targets across 20 tumor types is indicated in Figure 2 as a heat map. Of 23 candidate targets, the highest quasi *H*-score was specified to MS4A1 (score, 291.7) for its expression in lymphoma. Also, a quasi *H* − score ≥ 200 was identified for 13 candidate targets. Moreover, a score ≥ 150 across 13 tumor types was observed for CD276, a clinically relevant ADC target. For five additional candidate targets including ITGA3 (*n* = 11), PCDH7 (*n* = 6), SLC39A10 (*n* = 6), ATP2B2 (*n* = 5), and HTR2B (*n* = 5), a quasi *H* − score ≥ 150 in at least 5 tumor types was also found.

### 3.3. Prioritized Targets Based on the Expression in Normal Tissues

**Figure 3:**
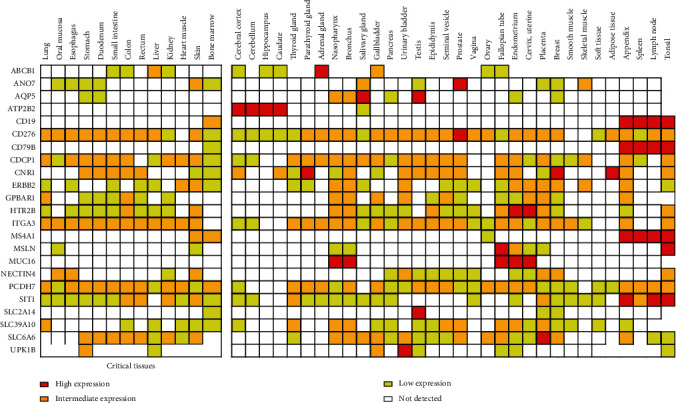
A heat map depicting the level of the protein expression for candidate ADC targets across 45 normal tissues.

To provide information for prediction of on-target off-tumor toxicity per each candidate target, the level of the target expression across 45 different normal tissues is shown in Figure 3 as a heat map. In order to exclusion of genes with high protein expression across 13 critical normal tissues during the screening process, no high level expression in the critical tissues was expectedly observed. Also, no medium expression across the critical tissues was observed for six candidate targets including AQP5, ATP2B2, CD79B, MSLN, MUC16, and SLC2A14. Besides, seven other candidate targets including CDCP1, ERBB2, GPBAR1, ITGA3, NECTIN4, PCDH7, and SLC39A10 showed no high expression across 45 normal tissues. Moreover, the high/medium expression in ≤5 normal tissues was observed for 10 candidate targets (Figure 3).

### 3.4. Clinical ADC Targets

As verification of the results, our list of candidate targets interestingly contains three pioneer targets, ERBB2 (HER2), NECTIN4 (PVRL4), and CD79B for which registered ADCs are currently in clinical use. Moreover, it contains four additional clinically relevant targets, CD19, CD276, MSLN, and MUC16, for which some ADCs are in clinical trials.

### 3.5. Experimental Validation

The paraffin blocks from 72 cases of urothelial cell carcinoma, including 68 tumors and 4 normal urinary bladder tissues, were retrospectively collected and assembled in TMAs. The characteristics of patients are summarized in Table 1.

**Figure 4:**
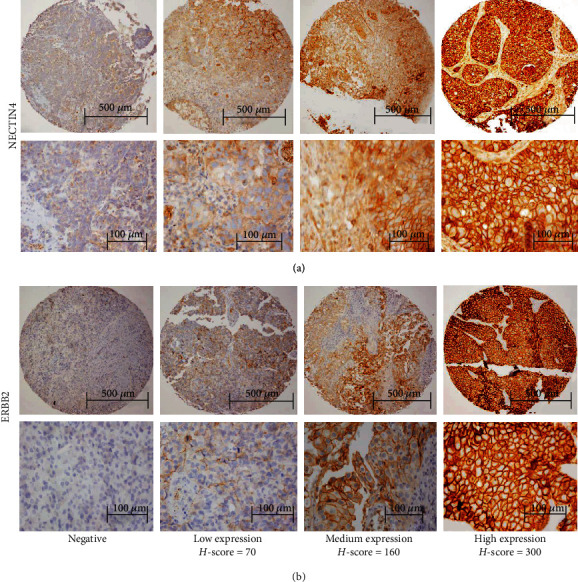
Experimental validation of the protein expression for (a) NECTIN4 and (b) ERBB2, as two FDA-approved antibody-drug conjugate targets, in representative samples of urothelial cell carcinoma.

The samples were immunohistochemically examined for the protein expression of NECTIN4 and ERBB2. Tumor tissue cores containing no tumor cells were excluded from the analysis. Membranous and cytoplasmic positivity was observed for both NECTIN4 and ERBB2. The IHC results confirmed the protein expression and localization of the NECTIN4 and ERBB2 in the bladder urothelial carcinoma (Figure 4).

Furthermore, the mean *H*-scores of these selected markers were compared with their quasi *H*-scores calculated from the HPA samples (Table 2**).** The lack of statistically significant differences shown in Table 2 might be considered as the experimental validation of HPA-derived quasi *H*-scores for the two FDA-approved targets in the urothelial cell carcinoma. As expected, intermediate staining was observed for NECTIN4 in the normal urinary bladder tissues. Also, low and intermediate ERBB2 immunoreactivity was detected in the normal bladder tissues.

### 3.6. Quasi *H*-Score Validation

For validation of the quasi *H*-score, the correlation of target *H*-scores with corresponding FPKM values, as a proxy of gene expression level, was assessed for different tumor types. The FPKM values for 23 ADC targets across 17 tumor types are indicated in Supplementary Figure S2 as a heat map. Interestingly, a significant correlation was found for 12/17 (70.5%) of tumor types with available FPKM values in the HPA database (Table 3).

## 4. Discussion

Increasing attention has been focused on ADC therapy due to the potential capacity of this type of targeted therapy to kill cancer cells [1, 2]. However, despite excessive efforts for development of effective drugs, most ADCs still have relatively narrow therapeutic index and limited clinical success [19]. The proper target identification is the first success factor for ADC development [4]. It should be noted that the identified target is a particular component of ADC development that is immutable and beyond the reach of the developer to refine or manipulate. Namely, if an ADC target is inappropriately selected, the development project is doomed to failure despite spending extensive time, effort, and money to refine the antibody, drug, or linker [5].

Recently, many efforts have been made in the discovery of molecular targets and prediction methods [20–23]. The accessibility of extensive open access biological data in the postgenomic era has revolutionized the field drug discovery. Since the identification of drug targets by computational methods saves a lot of financial resources, several computational approaches have been developed to complement experimental methods in discovery of novel drugs [20]. In this light, virtual screening methods such as molecular docking, pharmacophore modeling, quantitative structure–activity relationships (QSAR), and ligand-based in silico target prediction were applied [21]. Also, machine-learning methods can play a substantial role in the field [22, 23]. Some special features of ADC drugs raise the need for a unique algorithm to discover candidate ADC targets. Some special features of ADC drugs raise the need for a unique algorithm to discover candidate targets. An ideal ADC target should be expressed at the surface of tumor cells and have low expression on normal tissues to limit ontarget offtumor toxicity [1, 4]. The HPA project has provided in situ visualization of protein expression patterns using a standardized set of TMAs containing both normal human tissues and 20 most prevalent cancer types [24]. The combination of immunohistochemistry and TMA technology is known as an attractive strategy for high-throughput, antibody-based tissue proteomics [25]. Moreover, it is recognized that the highest impact on personalized medicine will be achieved by integrating a vast array of high-quality data; thus, there is increasing interest in applying big data to discover novel therapeutic targets [26]. The application of big data provided by the HPA could uncover novel targets missed during the laboratory discovery process and consequently may revolutionize the targeted therapy of cancer [9].

Since the expression levels of ADC targets in normal cells influence the drug distribution and safety profile, depicting the expression map of targets across different normal tissues is a critical clinical concern when selecting and prioritizing ADC targets for clinical use [4]. The expression map of candidate targets across 45 different normal tissues, shown in Figure 3, allows us to predict the potential for ontarget offtumor toxicity per individual target. To avoid on-target toxicity in critical normal tissues, the genes with high protein expression across 13 critical normal tissues were excluded during the screening. Besides, as another critical point, only predicted cell surface proteins were considered in this study for their potential draggability and accessibility to therapeutics.

Few data-driven studies have attempted to identify/prioritize ADC targets. Fauteux et al. [6], in a transcript-level study, have identified and prioritized some candidate ADC targets for breast cancer. The authors acknowledged that a proteomic study could provide better estimates. Also, Moek et al. [7], in a data-driven prioritization study, have prioritized 59 clinically relevant ADC targets across different tumor (sub) types by using functional genomic mRNA profiling. Of note, the gene expression alone cannot be a sole determinant of the target expression owing to the possibility of posttranscriptional and posttranslational changes; thus, high-quality IHC data from normal and tumor tissues is essential [4]. So, for the first time, this protein-level study was performed across a wide range of tumor types using the IHC data, as robust and clinically established evidence. Remarkably, we hypothesized that an *H*-score-like approach could provide some critical information about the level of the target expression and also target heterogeneity in the population, as two critical elements of ADC target selection [5]; thus, we innovatively used the quasi *H*-score to mathematically identify whether the selected proteins have high potential to qualify as ADC targets.

As a limitation, however, the final quasi *H*-scores may constitute from combined membranous/cytoplasmic staining, although only membrane proteins were included in this study. To overcome the scoring limitation, a visual predominant membranous staining in IHC samples was required for each potential target to pass the validation process. As another limitation, despite extensive HPA-based validation, our experimental validation only included two selected targets for a tumor type due to logistical constraints, such as dataset availability for different tumor types and also a large number of antibodies required for IHC examination.

Notably, the identification of several targets for which ADCs have registered or applied in clinical trials, using an unbiased systematic approach, is a good indicator of the validity of our findings. As previously mentioned, three ADCs targeting our identified targets have already been approved by the FDA for use in solid tumors and lymphoma. Also, four additional validated targets have been entered in clinical trials. Therefore, the presence of seven clinically relevant ADC targets in the list of candidate targets (corresponding to about one-third of all validated targets) serves as verification of the discovery approach and suggests that our list may also contain novel candidate targets with a high translational potential, shortening the therapeutic road from the laboratory to the clinic.

Of note, the antibody-based proteomic approach applied in this study opens up the possibility to identify and prioritize optimum combination of antibody-based therapeutics as the most rapidly growing drug class. Also, the list of candidate targets identified in this study may provide new avenues for development of similar antibody-related therapies such as radioimmunotherapy in which an antibody is labeled with a radionuclide to deliver cytotoxic radiation to a target cell. Interestingly, our list included the MS4A1 (CD20) as the only radioimmunotherapy target approved for clinical practice.

## 5. Conclusions

Our results showed that mining the HPA has the power to identify and prioritize candidate ADC targets with translational potential across different tumor types. Thus, this antibody-based large-scale study could help researchers and drug developers in deciding which targets should be taken for further investigation and consequently lead to development of new clinically effective and safe ADCs in the near future.

## Figures and Tables

**Table 1:** Characteristics of patients with urothelial carcinoma for experimental validation of two FDA-approved targets.

Characteristics	(*n* = 68)
Age,	

Mean (SD), years	68.03 (10.15)
Sex, *n* (%)	

Female	7 (10.3)

Male	61 (89.7)
T stage, *n* (%)	

T2	44 (64.7)

T3	19 (27.9)

T4a	5 (7.4)
Tumor grade, *n* (%)	

Low	3 (4.4)

High	65 (95.6)
N status, *n* (%)	

Negative	55 (80.9)

Positive	13 (19.1)

Abbreviation: SD: standard deviation.

**Table 2:** Comparison of the mean *H*-scores of the selected markers with their HPA-derived quasi *H*-scores.

Target protein	Mean *H*-score (SD)	Quasi *H*-score^∗^	Mean difference (95% CI)	*p*
NECTIN4	214.4 (86.7)	200	14.4 (-6.56 to 35.4)	0.175
ERBB2	82.0 (80.1)	100	-18.0 (-38.1 to 2.2)	0.081

Abbreviations: SD: standard deviation; CI: confidence interval. ^∗^Quasi *H*-scores calculated from the HPA for NECTIN4 and ERBB2 in patients with urothelial carcinoma.

**Table 3:** Correlation of target *H*-scores with corresponding FPKM values for different tumor types.

Tumor type	Correlation coefficient	*p*
Breast cancer	0.383	0.071
Cervical cancer	0.490	0.018
Colorectal cancer	0.318	0.139
Endometrial cancer	0.553	0.006
Glioma	0.597	0.003
Head and neck cancer	0.516	0.012
Liver cancer	0.581	0.004
Lung cancer	0.517	0.012
Melanoma	0.685	<0.001
Ovarian cancer	0.312	0.147
Pancreatic cancer	0.468	0.024
Prostate cancer	0.530	0.009
Renal cancer	0.738	<0.001
Stomach cancer	0.403	0.057
Testis cancer	0.358	0.093
Thyroid cancer	0.463	0.026
Urothelial cancer	0.608	0.002

## Data Availability

The data supporting the findings of the article is available in the HPA website at http://www.proteinatlas.org.

## Supplementary Materials

Supplementary Materials Supplementary Figure S1: process of tissue microarray construction. Supplementary Figure S2: a heat map depicting the FPKM values for candidate ADC targets across 17 tumor types. Supplementary Methods S1: HPA-based criteria for validation steps. Supplementary Table S1: list of predicted surfaceome proteins. Supplementary Table S2: list of differentially expressed genes with a quasi *H* − score ≥ 150 per tumor type. Supplementary Table S3: validation status for potential ADC targets.
