# Supplementary material for: Data-Driven Discovery of Molecular Targets for Antibody-Drug Conjugates in Cancer Treatment
Source: Biomed Res Int. 2021 Jan 2;2021:2670573. doi: 10.1155/2021/2670573 (PMC7801065; doi:10.1155/2021/2670573)
Supplement: Supplementary Materials — Supplementary Figure S1: process of tissue microarray construction. Supplementary Figure S2: a heat map depicting the FPKM values for candidate ADC targets across 17 tumor types. Supplementary Methods S1: HPA-based criteria for validation steps. Supplementary Table S1: list of predicted surfaceome proteins. Supplementary Table S2: list of differentially expressed genes with a quasi H − score ≥ 150 per tumor type. Supplementary Table S3: validation status for potential ADC targets. [file 2670573.f1.pdf]

Web Extra Materials

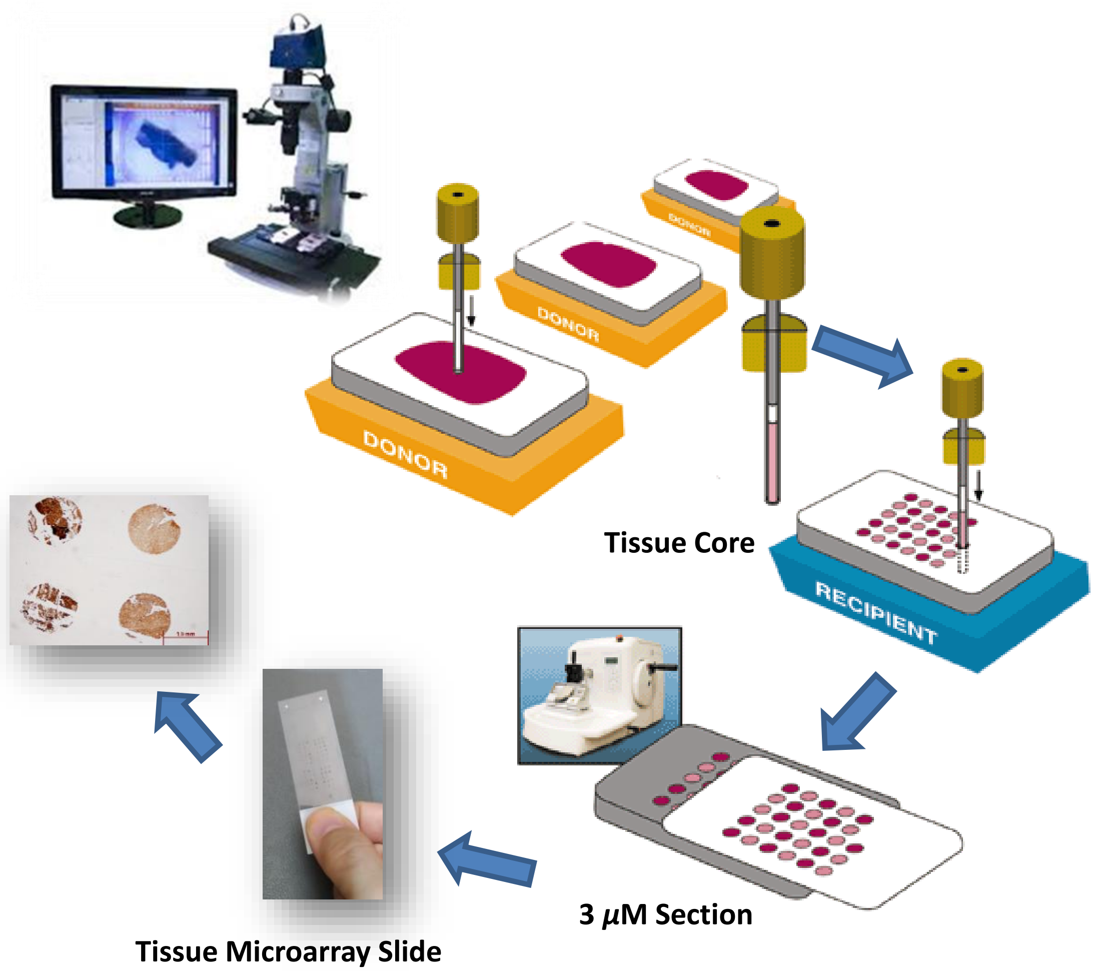

Supplementary Figure S1. Process of tissue microarray construction.
Representative areas of the tumor were identified by a pathologist on H&E stained sections. Reference histological slides with the specific area marked by the pathologist were aligned with the respective donor block. The corresponding areas were marked on paraffin blocks and three parallel tissue cores were obtained per tumor to account for intratumoral heterogeneity. Tissue arrays were constructed by placing 1 mm diameter cores in recipient paraffin blocks using a tissue arrayer (Galileo TMA CK3500 Tissue Micro arrayer; ISETMA Software, Integrated System Engineering, Milan, Italy), and limited to 72 cores per recipient block. Then, consecutive sections (with a thickness of 3 µm) were cut from each TMA block, and mounted on microscope slides. Finally, the unstained slides were deparaffinized and rehydratedand using standard methods, and immunohistochemically assayed.

	Breast cancer	Cervical cancer	Colorectal cancer	Endometrial cancer	Glioma	Head and neck cancer	Liver cancer	Lung cancer	Melanoma	Ovarian cancer	Pancreatic cancer	Prostate cancer	Renal cancer	Stomach cancer	Testis cancer	Thyroid cancer	Urothelial cancer
ABCB1	0.6	0.3	4.6	0.6	2.7	0.2	7.8	0.3	0.2	0.2	2.3	1.5	7.1	0.9	0.5	0.8	0.3
ANO7	0.3	0.3	0.8	0.6	0.3	0.2	0.4	0.4	0.4	0.1	0.6	27	0.5	0.8	0.5	0.4	0.3
AQP5	0.4	1.9	0.1	53.8	2.4	0.2	0	1.7	0.3	21.3	20.8	0.2	0	3.9	1.8	4.5	0.1
ATP2B2	0	0	0	0.4	3.2	0	6.7	0	0	0.9	0	0	0.8	0	0	0	0
CD19	0.2	0.3	0.3	0.4	0.1	0.2	0.1	0.9	0.1	0.3	0.3	0.1	0.1	0.7	1.9	0.1	0.2
CD276	23	14.8	16.5	26.7	25.2	25	8.9	20.9	30.1	17.9	22.8	21.6	13.6	15.7	20.3	13.2	20
CD79B	1.4	0.9	0.9	1	0.3	0.9	1	2.3	1.1	0.6	1.7	1	2	1.2	2.9	1.3	0.8
CDCP1	8.5	19	16.3	12.3	1.4	22	0.1	12.3	1.4	8.9	16.3	5	4.3	16.2	2.6	9.8	12.1
CNR1	0.1	0	0	0	8.7	0	0	0.4	0	0.1	0.4	0.1	0.2	0.2	0.4	0.1	0.1
ERBB2	34.9	22.2	23.7	23.1	5.9	14.3	7.2	19.1	7.5	18.9	23.1	26.2	15.4	22	7.3	30.4	35.2
GPBAR1	0.2	0.1	0.6	0.3	0.2	0.2	0.3	0.2	0.2	0.4	2.9	0.2	0.4	1.3	0.5	0.2	0.3
HTR2B	0.5	0.2	0.1	0.7	0.1	0.1	0.3	0.4	0.2	0.4	1.3	0.1	0.4	0.4	0.2	0.4	0.2
ITGA3	8.7	25	13.3	11.1	7.1	49	0.8	29.9	31.6	22.2	42.4	6.3	36.1	21.5	2.2	82.2	38.4
MS4A1	0.3	0.2	0.2	0.1	0	0.2	0.1	0.9	0	0	0.4	0.1	0.1	0.8	1	0.1	0.1
MSLN	0.2	23.9	13.6	17.5	0.5	1.5	0	7.5	0.1	290.9	89.7	0.4	0.5	19.7	0.2	0.2	0.4
MUC16	0.1	1	0	2.7	0	0.1	0	0.2	0	14.2	0.7	0	0	0	0.1	0	0
NECTIN4	21	46.2	5.2	12	0.1	33.1	0.1	21.9	0.7	6.6	15.5	10.2	0.1	4.3	2.3	8.7	53.3
PCDH7	1.5	2.9	0.6	4.4	1.2	4	0	1.6	5.9	3.2	3.4	1.3	0.2	3.2	0.6	2.1	1.1
SIT1	1.1	1.4	1.5	1	0.3	1.2	0.5	2	0.7	1	1.2	0.6	1.4	2	3.9	0.6	0.7
SLC2A14	0	0	0	0	0.1	0	0	0.1	0	0.1	0.1	0	0.1	0	3.9	0	0
SLC39A10	6	3.1	5.6	5.5	8.4	2.8	1.1	5.4	3.5	8.6	4.7	7.9	5.5	4.2	10.4	19.1	3.5
SLC6A6	6.9	4.5	13.4	10	2.7	6.5	0.6	11.5	4.6	4.7	15.3	1.6	6.9	8.9	11.2	8.3	6.9
UPK1B	0	0.1	0	5.2	0	0.5	0	0.3	0	4.2	1.8	0	0.4	0.2	0.1	0	62.3

Supplementary Figure S2. A heat map depicting the FPKM values for candidate ADC targets across 17 tumor types

Supplementary Methods S1. HPA-based criteria for validation steps

The immunostaining result of each antibody is compared with available gene/RNA/protein characterization data, resulting in different validations: Literature conformity and RNA consistency.

Literature conformity:

Literature conformity refers to the conformance of the expression pattern to available gene/protein characterization data in scientific literature and data from bioinformatic predictions. UniProt is used as the main source of gene/protein characterization data and when relevant, available publications and other sources of information are probed in depth. Extensive or sufficient gene/protein data requires that there is evidence of existence on a protein level and that a substantial quantity of published experimental data is available from literature and public databases. Limited protein/gene data does not require evidence of existence on a protein level and refers to genes for which only bioinformatic predictions and scarce published experimental data is available.

The different options of literature conformity are:

- Consistent with extensive gene/protein characterization data
- Consistent with gene/protein characterization data
- Partly consistent with extensive gene/protein characterization data
- Partly consistent with gene/protein characterization data
- No available gene/protein characterization data
- Not consistent with gene/protein characterization data

RNA consistency:

RNA consistency is based on a comparison of antibody staining in 44 normal tissues with RNA-seq data combined from HPA, GTEX and FANTOM.

RNA consistency is scored as follows:

- Consistent with RNA expression data
- Mainly consistent with RNA expression data
- Mainly not consistent with RNA expression data
- Not consistent with RNA expression data
- No internal RNA expression data available for correlation

Verification of membrane localization:

In addition to RNA consistency and literature conformity, verification of membrane localization were considered for expression validation of potential targets. As stated in the methods section, the IHC images and description of the staining pattern (available at the HPA database) for each antibody in cancer tissues were considered for verification of membrane localization. A predominant membranous staining in IHC samples was required for each potential target to pass the validation process. Only potential targets that passed three HPA-based validation steps were considered as candidate targets.

Supplementary Table S1. List of predicted surfaceome proteins

UniProt name	UniProt description	UniProt gene	Main-class
**S12A8_HUMAN**	Solute carrier family 12 member 8	SLC12A8	Transporters
**ESYT3_HUMAN**	Extended synaptotagmin-3	ESYT3	Unclassified
**SC5AA_HUMAN**	Sodium/glucose cotransporter 5	SLC5A10	Transporters
**CLRN2_HUMAN**	Clarin-2	CLRN2	Unclassified
**CC50C_HUMAN**	Cell cycle control protein 50C	TMEM30C	Miscellaneous
**ANO9_HUMAN**	Anoctamin-9	ANO9	Miscellaneous
**S22AN_HUMAN**	Solute carrier family 22 member 23	SLC22A23	Transporters
**TSN11_HUMAN**	Tetraspanin-11	TSPAN11	Miscellaneous
**TM213_HUMAN**	Transmembrane protein 213	TMEM213	Unclassified
**T131L_HUMAN**	Transmembrane protein 131-like	KIAA0922	
**OR2M5_HUMAN**	Olfactory receptor 2M5	OR2M5	Receptors
**O2A25_HUMAN**	Olfactory receptor 2A25	OR2A25	Receptors
**S35F4_HUMAN**	Solute carrier family 35 member F4	SLC35F4	Transporters
**5HT3E_HUMAN**	5-hydroxytryptamine receptor 3E	HTR3E	Transporters
**MEG11_HUMAN**	Multiple epidermal growth factor-like domains protein 11	MEGF11	Receptors
**CDHR4_HUMAN**	Cadherin-related family member 4	CDHR4	Miscellaneous
**T150B_HUMAN**	Transmembrane protein 150B	TMEM150B	Unclassified
**O6C74_HUMAN**	Olfactory receptor 6C74	OR6C74	Receptors
**JUNO_HUMAN**	Sperm-egg fusion protein Juno	IZUMO1R	Unclassified
**O14I1_HUMAN**	Olfactory receptor 14I1	OR14I1	Receptors
**LRIT2_HUMAN**	Leucine-rich repeat, immunoglobulin-like domain and transmembrane domain-containing protein 2	LRIT2	Miscellaneous
**O5H15_HUMAN**	Olfactory receptor 5H15	OR5H15	Receptors
**O6C68_HUMAN**	Olfactory receptor 6C68	OR6C68	Receptors
**MADL2_HUMAN**	Myeloid-associated differentiation marker-like protein 2	MYADML2	Unclassified
**TMM8B_HUMAN**	Transmembrane protein 8B	TMEM8B	Unclassified
**OR5K3_HUMAN**	Olfactory receptor 5K3	OR5K3	Receptors
**ANTRL_HUMAN**	Anthrax toxin receptor-like	ANTXRL	Unclassified
**OR6C6_HUMAN**	Olfactory receptor 6C6	OR6C6	Receptors
**TIKI2_HUMAN**	Metalloprotease TIKI2	TRABD2B	Unclassified
**TM235_HUMAN**	Transmembrane protein 235	TMEM235	Transporters
**MFS2B_HUMAN**	Major facilitator superfamily domain-containing protein 2B	MFSD2B	Unclassified
**GGT3_HUMAN**	Putative gamma-glutamyltranspeptidase 3	GGT3P	Unclassified
**O51F1_HUMAN**	Olfactory receptor 51F1	OR51F1	Receptors
**OR2T8_HUMAN**	Olfactory receptor 2T8	OR2T8	Receptors
**SERC4_HUMAN**	Serine incorporator 4	SERINC4	Transporters
**O4C46_HUMAN**	Olfactory receptor 4C46	OR4C46	Receptors

**O5H14_HUMAN**	Olfactory receptor 5H14	OR5H14	Receptors
**MANS4_HUMAN**	MANSC domain-containing protein 4	MANSC4	Unclassified
**LIRA5_HUMAN**	Leukocyte immunoglobulin-like receptor subfamily A member 5	LILRA5	Receptors
**O6C70_HUMAN**	Olfactory receptor 6C70	OR6C70	Receptors
**S15A5_HUMAN**	Solute carrier family 15 member 5	SLC15A5	Unclassified
**CD8BL_HUMAN**	Putative T-cell surface glycoprotein CD8 beta-2 chain	CD8BP	Unclassified
**O6C65_HUMAN**	Olfactory receptor 6C65	OR6C65	Receptors
**S22AK_HUMAN**	Solute carrier family 22 member 20	SLC22A20	Transporters
**PCX2_HUMAN**	Pecanex-like protein 2	PCNXL2	Unclassified
**FCGRC_HUMAN**	Putative high affinity immunoglobulin gamma Fc receptor IC	FCGR1C	Receptors
**OR5H1_HUMAN**	Olfactory receptor 5H1	OR5H1	Receptors
**O6C75_HUMAN**	Olfactory receptor 6C75	OR6C75	Receptors
**OR5BL_HUMAN**	Olfactory receptor 5B21	OR5B21	Receptors
**SHSA7_HUMAN**	Protein shisa-7	SHISA7	
**O2AG2_HUMAN**	Olfactory receptor 2AG2	OR2AG2	Receptors
**L37A2_HUMAN**	Leucine-rich repeat-containing protein 37A2	LRRC37A2	Miscellaneous
**CLD24_HUMAN**	Putative claudin-24	CLDN24	Miscellaneous
**O6C76_HUMAN**	Olfactory receptor 6C76	OR6C76	Receptors
**SIG16_HUMAN**	Sialic acid-binding Ig-like lectin 16	SIGLEC16	Miscellaneous
**OR5K4_HUMAN**	Olfactory receptor 5K4	OR5K4	Receptors
**L37A1_HUMAN**	Leucine-rich repeat-containing protein 37A	LRRC37A	Miscellaneous
**O52A4_HUMAN**	Putative olfactory receptor 52A4	OR52A4	Receptors
**O4C45_HUMAN**	Olfactory receptor 4C45	OR4C45	Receptors
**O2AT4_HUMAN**	Olfactory receptor 2AT4	OR2AT4	Receptors
**S38A8_HUMAN**	Putative sodium-coupled neutral amino acid transporter 8	SLC38A8	Transporters
**DISP2_HUMAN**	Protein dispatched homolog 2	DISP2	Unclassified
**GBRR3_HUMAN**	Gamma-aminobutyric acid receptor subunit rho-3	GABRR3	Transporters
**GSG1M_HUMAN**	Putative germ cell-specific gene 1-like protein 2		Unclassified
**HIDE1_HUMAN**	Protein HIDE1	HIDE1	Unclassified
**F1712_HUMAN**	Protein FAM171A2	FAM171A2	Unclassified
**HECA2_HUMAN**	HEPACAM family member 2	HEPACAM2	Unclassified
**BTNLA_HUMAN**	Butyrophilin-like protein 10	BTNL10	
**K132L_HUMAN**	UPF0577 protein KIAA1324-like	KIAA1324L	Unclassified
**VSTM5_HUMAN**	V-set and transmembrane domain-containing protein 5	VSTM5	Unclassified
**UPK3L_HUMAN**	Uroplakin-3b-like protein	UPK3BL	Miscellaneous
**O11HC_HUMAN**	Olfactory receptor 11H12	OR11H12	Receptors
**TM114_HUMAN**	Transmembrane protein 114	TMEM114	Unclassified
**SHSA9_HUMAN**	Protein shisa-9	SHISA9	Unclassified
**TARM1_HUMAN**	T-cell-interacting, activating receptor on myeloid cells protein 1	TARM1	Unclassified
**ERVV1_HUMAN**	Endogenous retrovirus group V member 1 Env polyprotein	ERVV-1	Miscellaneous

**ERVV2_HUMAN**	Endogenous retrovirus group V member 2 Env polyprotein	ERVV-2	Miscellaneous
**SHSA8_HUMAN**	Putative protein shisa-8	SHISA8	Unclassified
**KLRF2_HUMAN**	Killer cell lectin-like receptor subfamily F member 2	KLRF2	
**MUC22_HUMAN**	Mucin-22	MUC22	
**CA233_HUMAN**	Fibronectin type-III domain-containing transmembrane protein C1orf233	C1orf233	
**CC080_HUMAN**	Uncharacterized membrane protein C3orf80	C3orf80	
**SO1B7_HUMAN**	Putative solute carrier organic anion transporter family member 1B7	SLCO1B7	Transporters
**T178B_HUMAN**	Transmembrane protein 178B	TMEM178B	
**GFY_HUMAN**	Golgi-associated olfactory signaling regulator	GFY	
**FZD9_HUMAN**	Frizzled-9	FZD9	Receptors
**GPR25_HUMAN**	Probable G-protein coupled receptor 25	GPR25	Receptors
**TLR4_HUMAN**	Toll-like receptor 4	TLR4	Receptors
**TR10A_HUMAN**	Tumor necrosis factor receptor superfamily member 10A	TNFRSF10A	Receptors
**GRM8_HUMAN**	Metabotropic glutamate receptor 8	GRM8	Receptors
**SIRB1_HUMAN**	Signal-regulatory protein beta-1	SIRPB1	Miscellaneous
**PAR3_HUMAN**	Proteinase-activated receptor 3	F2RL2	Receptors
**GPR31_HUMAN**	12-(S)-hydroxy-5,8,10,14-eicosatetraenoic acid receptor	GPR31	Receptors
**UPK1A_HUMAN**	Uroplakin-1a	UPK1A	Miscellaneous
**S28A1_HUMAN**	Sodium/nucleoside cotransporter 1	SLC28A1	Transporters
**EAA5_HUMAN**	Excitatory amino acid transporter 5	SLC1A7	Transporters
**QSOX1_HUMAN**	Sulfhydryl oxidase 1	QSOX1	Enzymes
**P2Y10_HUMAN**	Putative P2Y purinoceptor 10	P2RY10	Receptors
**ACATN_HUMAN**	Acetyl-coenzyme A transporter 1	SLC33A1	Transporters
**CCRL2_HUMAN**	C-C chemokine receptor-like 2	CCRL2	Receptors
**GFRA2_HUMAN**	GDNF family receptor alpha-2	GFRA2	
**BT3A3_HUMAN**	Butyrophilin subfamily 3 member A3	BTN3A3	Miscellaneous
**BT3A1_HUMAN**	Butyrophilin subfamily 3 member A1	BTN3A1	Miscellaneous
**UPK2_HUMAN**	Uroplakin-2	UPK2	Unclassified
**NCHL1_HUMAN**	Neural cell adhesion molecule L1-like protein	CHL1	Miscellaneous
**DLL1_HUMAN**	Delta-like protein 1	DLL1	Miscellaneous
**CXCR6_HUMAN**	C-X-C chemokine receptor type 6	CXCR6	Receptors
**ACKR2_HUMAN**	Atypical chemokine receptor 2	ACKR2	Receptors
**GBRP_HUMAN**	Gamma-aminobutyric acid receptor subunit pi	GABRP	Transporters
**PODXL_HUMAN**	Podocalyxin	PODXL	Unclassified
**CLD4_HUMAN**	Claudin-4	CLDN4	Miscellaneous
**LPP1_HUMAN**	Lipid phosphate phosphohydrolase 1	PPAP2A	Enzymes
**LPP3_HUMAN**	Lipid phosphate phosphohydrolase 3	PPAP2B	Enzymes
**NRG2_HUMAN**	Pro-neuregulin-2, membrane-bound isoform	NRG2	Unclassified
**BAI1_HUMAN**	Brain-specific angiogenesis inhibitor 1	BAI1	Receptors
**PTPRT_HUMAN**	Receptor-type tyrosine-protein phosphatase T	PTPRT	Receptors

**T194A_HUMAN**	Transmembrane protein 194A	TMEM194A	Unclassified
**ASTN1_HUMAN**	Astrotactin-1	ASTN1	Unclassified
**OR7AH_HUMAN**	Olfactory receptor 7A17	OR7A17	Receptors
**GP171_HUMAN**	Probable G-protein coupled receptor 171	GPR171	Receptors
**ADA10_HUMAN**	Disintegrin and metalloproteinase domain-containing protein 10	ADAM10	Enzymes
**OPSX_HUMAN**	Visual pigment-like receptor peropsin	RRH	Receptors
**GBRD_HUMAN**	Gamma-aminobutyric acid receptor subunit delta	GABRD	Transporters
**NRP1_HUMAN**	Neuropilin-1	NRP1	Receptors
**TNF11_HUMAN**	Tumor necrosis factor ligand superfamily member 11	TNFSF11	Unclassified
**TR10C_HUMAN**	Tumor necrosis factor receptor superfamily member 10C	TNFRSF10C	Receptors
**TAAR5_HUMAN**	Trace amine-associated receptor 5	TAAR5	Receptors
**TSN4_HUMAN**	Tetraspanin-4	TSPAN4	Miscellaneous
**TR13B_HUMAN**	Tumor necrosis factor receptor superfamily member 13B	TNFRSF13B	Receptors
**FFAR1_HUMAN**	Free fatty acid receptor 1	FFAR1	Receptors
**FFAR3_HUMAN**	Free fatty acid receptor 3	FFAR3	Receptors
**T4S5_HUMAN**	Transmembrane 4 L6 family member 5	TM4SF5	Miscellaneous
**PCD17_HUMAN**	Protocadherin-17	PCDH17	Miscellaneous
**NCTR3_HUMAN**	Natural cytotoxicity triggering receptor 3	NCR3	Receptors
**EREG_HUMAN**	Proepiregulin	EREG	Unclassified
**PLXB2_HUMAN**	Plexin-B2	PLXNB2	Receptors
**NPC1_HUMAN**	Niemann-Pick C1 protein	NPC1	Unclassified
**MUSK_HUMAN**	Muscle, skeletal receptor tyrosine-protein kinase	MUSK	Receptors
**LRAD4_HUMAN**	Low-density lipoprotein receptor class A domain-containing protein 4	LDLRAD4	Unclassified
**EPHB6_HUMAN**	Ephrin type-B receptor 6	EPHB6	Receptors
**GP182_HUMAN**	G-protein coupled receptor 182	GPR182	Receptors
**S22A2_HUMAN**	Solute carrier family 22 member 2	SLC22A2	Transporters
**S22A1_HUMAN**	Solute carrier family 22 member 1	SLC22A1	Transporters
**GRM6_HUMAN**	Metabotropic glutamate receptor 6	GRM6	Receptors
**TM9S1_HUMAN**	Transmembrane 9 superfamily member 1	TM9SF1	Miscellaneous
**GPR37_HUMAN**	Prosaposin receptor GPR37	GPR37	Receptors
**MOT5_HUMAN**	Monocarboxylate transporter 5	SLC16A4	Transporters
**MOT6_HUMAN**	Monocarboxylate transporter 6	SLC16A5	Transporters
**SIGL5_HUMAN**	Sialic acid-binding Ig-like lectin 5	SIGLEC5	Miscellaneous
**NCAM2_HUMAN**	Neural cell adhesion molecule 2	NCAM2	Miscellaneous
**NMDE4_HUMAN**	Glutamate receptor ionotropic, NMDA 2D	GRIN2D	Transporters
**MOT7_HUMAN**	Monocarboxylate transporter 7	SLC16A6	Transporters
**COPT1_HUMAN**	High affinity copper uptake protein 1	SLC31A1	Transporters
**MRP3_HUMAN**	Canalicular multispecific organic anion transporter 2	ABCC3	Transporters
**MRP4_HUMAN**	Multidrug resistance-associated protein 4	ABCC4	Transporters
**MRP5_HUMAN**	Multidrug resistance-associated protein 5	ABCC5	Transporters

**TLR3_HUMAN**	Toll-like receptor 3	TLR3	Receptors
**GPR42_HUMAN**	G-protein coupled receptor 42	GPR42	Receptors
**P2RX6_HUMAN**	P2X purinoceptor 6	P2RX6	Transporters
**CLD3_HUMAN**	Claudin-3	CLDN3	Miscellaneous
**FFAR2_HUMAN**	Free fatty acid receptor 2	FFAR2	Receptors
**ENK18_HUMAN**	Endogenous retrovirus group K member 18 Env polyprotein	ERVK-18	Miscellaneous
**FLRT2_HUMAN**	Leucine-rich repeat transmembrane protein FLRT2	FLRT2	Miscellaneous
**PLXB1_HUMAN**	Plexin-B1	PLXNB1	Receptors
**ADA12_HUMAN**	Disintegrin and metalloproteinase domain-containing protein 12	ADAM12	Enzymes
**MTLR_HUMAN**	Motilin receptor	MLNR	Receptors
**GPR39_HUMAN**	G-protein coupled receptor 39	GPR39	Receptors
**CTR4_HUMAN**	Cationic amino acid transporter 4	SLC7A4	Transporters
**TREA_HUMAN**	Trehalase	TREH	
**SPIT2_HUMAN**	Kunitz-type protease inhibitor 2	SPINT2	Unclassified
**LRRT2_HUMAN**	Leucine-rich repeat transmembrane neuronal protein 2	LRRTM2	Miscellaneous
**ADCY6_HUMAN**	Adenylate cyclase type 6	ADCY6	Enzymes
**AQP9_HUMAN**	Aquaporin-9	AQP9	Transporters
**GRID2_HUMAN**	Glutamate receptor ionotropic, delta-2	GRID2	Transporters
**PROM1_HUMAN**	Prominin-1	PROM1	Unclassified
**TGON2_HUMAN**	Trans-Golgi network integral membrane protein 2	TGOLN2	Unclassified
**CAC1G_HUMAN**	Voltage-dependent T-type calcium channel subunit alpha-1G	CACNA1G	Transporters
**ADA20_HUMAN**	Disintegrin and metalloproteinase domain-containing protein 20	ADAM20	Enzymes
**S26A4_HUMAN**	Pendrin	SLC26A4	Transporters
**SGCE_HUMAN**	Epsilon-sarcoglycan	SGCE	Miscellaneous
**RNF13_HUMAN**	E3 ubiquitin-protein ligase RNF13	RNF13	Unclassified
**CAH12_HUMAN**	Carbonic anhydrase 12	CA12	Enzymes
**GALR2_HUMAN**	Galanin receptor type 2	GALR2	Receptors
**OX1R_HUMAN**	Orexin receptor type 1	HCRTR1	Receptors
**OX2R_HUMAN**	Orexin receptor type 2	HCRTR2	Receptors
**PSCA_HUMAN**	Prostate stem cell antigen	PSCA	Unclassified
**TSN6_HUMAN**	Tetraspanin-6	TSPAN6	Miscellaneous
**LPP2_HUMAN**	Lipid phosphate phosphohydrolase 2	PPAP2C	Enzymes
**SIGL6_HUMAN**	Sialic acid-binding Ig-like lectin 6	SIGLEC6	Miscellaneous
**OR1F1_HUMAN**	Olfactory receptor 1F1	OR1F1	Receptors
**G6PT1_HUMAN**	Glucose-6-phosphate translocase	SLC37A4	Transporters
**S28A2_HUMAN**	Sodium/nucleoside cotransporter 2	SLC28A2	Transporters
**OR2T1_HUMAN**	Olfactory receptor 2T1	OR2T1	Receptors
**XPP2_HUMAN**	Xaa-Pro aminopeptidase 2	XPNPEP2	
**EFNA2_HUMAN**	Ephrin-A2	EFNA2	Unclassified
**MFS11_HUMAN**	UNC93-like protein MFSD11	MFSD11	Unclassified

**TM11D_HUMAN**	Transmembrane protease serine 11D	TMPRSS11D	Enzymes
**BAI2_HUMAN**	Brain-specific angiogenesis inhibitor 2	BAI2	Receptors
**BAI3_HUMAN**	Brain-specific angiogenesis inhibitor 3	BAI3	Receptors
**PCDH7_HUMAN**	Protocadherin-7	PCDH7	Miscellaneous
**ADCY3_HUMAN**	Adenylate cyclase type 3	ADCY3	Enzymes
**SUSD5_HUMAN**	Sushi domain-containing protein 5	SUSD5	Unclassified
**L37A3_HUMAN**	Leucine-rich repeat-containing protein 37A3	LRRC37A3	Miscellaneous
**PCDGC_HUMAN**	Protocadherin gamma-A12	PCDHGA12	Miscellaneous
**FZD6_HUMAN**	Frizzled-6	FZD6	Receptors
**CCG3_HUMAN**	Voltage-dependent calcium channel gamma-3 subunit	CACNG3	Transporters
**NMD3B_HUMAN**	Glutamate receptor ionotropic, NMDA 3B	GRIN3B	Transporters
**O10H2_HUMAN**	Olfactory receptor 10H2	OR10H2	Receptors
**O10H3_HUMAN**	Olfactory receptor 10H3	OR10H3	Receptors
**OR7C2_HUMAN**	Olfactory receptor 7C2	OR7C2	Receptors
**OR1I1_HUMAN**	Olfactory receptor 1I1	OR1I1	Receptors
**LY75_HUMAN**	Lymphocyte antigen 75	LY75	Receptors
**NRP2_HUMAN**	Neuropilin-2	NRP2	Receptors
**DSCAM_HUMAN**	Down syndrome cell adhesion molecule	DSCAM	Miscellaneous
**G137B_HUMAN**	Integral membrane protein GPR137B	GPR137B	Unclassified
**PLXC1_HUMAN**	Plexin-C1	PLXNC1	Receptors
**MPZL2_HUMAN**	Myelin protein zero-like protein 2	MPZL2	Miscellaneous
**NPHN_HUMAN**	Nephrin	NPHS1	Unclassified
**ADCY9_HUMAN**	Adenylate cyclase type 9	ADCY9	Enzymes
**TLR5_HUMAN**	Toll-like receptor 5	TLR5	Receptors
**TLR2_HUMAN**	Toll-like receptor 2	TLR2	Receptors
**GFRA3_HUMAN**	GDNF family receptor alpha-3	GFRA3	Unclassified
**TSN1_HUMAN**	Tetraspanin-1	TSPAN1	Miscellaneous
**TSN2_HUMAN**	Tetraspanin-2	TSPAN2	Miscellaneous
**TSN3_HUMAN**	Tetraspanin-3	TSPAN3	Miscellaneous
**MOT2_HUMAN**	Monocarboxylate transporter 2	SLC16A7	Transporters
**ABCC9_HUMAN**	ATP-binding cassette sub-family C member 9	ABCC9	Transporters
**GALR3_HUMAN**	Galanin receptor type 3	GALR3	Receptors
**S19A2_HUMAN**	Thiamine transporter 1	SLC19A2	Transporters
**ETBR2_HUMAN**	Prosaposin receptor GPR37L1	GPR37L1	Receptors
**RAMP2_HUMAN**	Receptor activity-modifying protein 2	RAMP2	Receptors
**RAMP3_HUMAN**	Receptor activity-modifying protein 3	RAMP3	Receptors
**CTNS_HUMAN**	Cystinosin	CTNS	Unclassified
**SCN2B_HUMAN**	Sodium channel subunit beta-2	SCN2B	Transporters
**ENK19_HUMAN**	Endogenous retrovirus group K member 19 Env polyprotein	ERVK-19	Miscellaneous
**FCG3B_HUMAN**	Low affinity immunoglobulin gamma Fc region receptor III-B	FCGR3B	Receptors

**LIRA1_HUMAN**	Leukocyte immunoglobulin-like receptor subfamily A member 1	LILRA1	Receptors
**LIRB3_HUMAN**	Leukocyte immunoglobulin-like receptor subfamily B member 3	LILRB3	Receptors
**LIRB5_HUMAN**	Leukocyte immunoglobulin-like receptor subfamily B member 5	LILRB5	Receptors
**PLXA2_HUMAN**	Plexin-A2	PLXNA2	Receptors
**IGSF3_HUMAN**	Immunoglobulin superfamily member 3	IGSF3	Receptors
**LRP3_HUMAN**	Low-density lipoprotein receptor-related protein 3	LRP3	Receptors
**ADA23_HUMAN**	Disintegrin and metalloproteinase domain-containing protein 23	ADAM23	Enzymes
**ADA11_HUMAN**	Disintegrin and metalloproteinase domain-containing protein 11	ADAM11	Enzymes
**FZD7_HUMAN**	Frizzled-7	FZD7	Receptors
**LRP4_HUMAN**	Low-density lipoprotein receptor-related protein 4	LRP4	Receptors
**MFA3L_HUMAN**	Microfibrillar-associated protein 3-like	MFAP3L	Unclassified
**ASTN2_HUMAN**	Astrotactin-2	ASTN2	Unclassified
**ICOSL_HUMAN**	ICOS ligand	ICOSLG	Miscellaneous
**LRP5_HUMAN**	Low-density lipoprotein receptor-related protein 5	LRP5	Receptors
**CAD16_HUMAN**	Cadherin-16	CDH16	Miscellaneous
**GLRA3_HUMAN**	Glycine receptor subunit alpha-3	GLRA3	Transporters
**LRRN2_HUMAN**	Leucine-rich repeat neuronal protein 2	LRRN2	Miscellaneous
**SEM7A_HUMAN**	Semaphorin-7A	SEMA7A	
**ENTP3_HUMAN**	Ectonucleoside triphosphate diphosphohydrolase 3	ENTPD3	Enzymes
**LAT3_HUMAN**	Large neutral amino acids transporter small subunit 3	SLC43A1	Transporters
**GPR32_HUMAN**	Probable G-protein coupled receptor 32	GPR32	Receptors
**TECTA_HUMAN**	Alpha-tectorin	TECTA	Unclassified
**USH2A_HUMAN**	Usherin	USH2A	Unclassified
**LGR5_HUMAN**	Leucine-rich repeat-containing G-protein coupled receptor 5	LGR5	Receptors
**GPC4_HUMAN**	Glypican-4	GPC4	Unclassified
**CLD11_HUMAN**	Claudin-11	CLDN11	Miscellaneous
**TNR21_HUMAN**	Tumor necrosis factor receptor superfamily member 21	TNFRSF21	Receptors
**ITA10_HUMAN**	Integrin alpha-10	ITGA10	Receptors
**LRP6_HUMAN**	Low-density lipoprotein receptor-related protein 6	LRP6	Receptors
**UPK3A_HUMAN**	Uroplakin-3a	UPK3A	Miscellaneous
**CXB3_HUMAN**	Gap junction beta-3 protein	GJB3	Miscellaneous
**S22A3_HUMAN**	Solute carrier family 22 member 3	SLC22A3	Transporters
**UPK1B_HUMAN**	Uroplakin-1b	UPK1B	Miscellaneous
**CEAM4_HUMAN**	Carcinoembryonic antigen-related cell adhesion molecule 4	CEACAM4	Miscellaneous
**ATRN_HUMAN**	Attractin	ATRN	Unclassified
**GABR2_HUMAN**	Gamma-aminobutyric acid type B receptor subunit 2	GABBR2	Receptors
**TSN9_HUMAN**	Tetraspanin-9	TSPAN9	Miscellaneous
**CBPD_HUMAN**	Carboxypeptidase D	CPD	Enzymes
**OR2B3_HUMAN**	Putative olfactory receptor 2B3	OR2B3	Receptors
**OR2J3_HUMAN**	Olfactory receptor 2J3	OR2J3	Receptors

**OR2J2_HUMAN**	Olfactory receptor 2J2	OR2J2	Receptors
**NCTR1_HUMAN**	Natural cytotoxicity triggering receptor 1	NCR1	Receptors
**S22A5_HUMAN**	Solute carrier family 22 member 5	SLC22A5	Transporters
**OR7C1_HUMAN**	Olfactory receptor 7C1	OR7C1	Receptors
**OR7AA_HUMAN**	Olfactory receptor 7A10	OR7A10	Receptors
**LY6H_HUMAN**	Lymphocyte antigen 6H	LY6H	
**AQP8_HUMAN**	Aquaporin-8	AQP8	Transporters
**CNTN5_HUMAN**	Contactin-5	CNTN5	Unclassified
**NFASC_HUMAN**	Neurofascin	NFASC	Miscellaneous
**CSCL1_HUMAN**	CSC1-like protein 1	TMEM63A	Miscellaneous
**LRIG2_HUMAN**	Leucine-rich repeats and immunoglobulin-like domains protein 2	LRIG2	Miscellaneous
**LPHN1_HUMAN**	Latrophilin-1	LPHN1	Receptors
**ABCA8_HUMAN**	ATP-binding cassette sub-family A member 8	ABCA8	Transporters
**SLIK3_HUMAN**	SLIT and NTRK-like protein 3	SLITRK3	Miscellaneous
**SO2B1_HUMAN**	Solute carrier organic anion transporter family member 2B1	SLCO2B1	Transporters
**CSTN1_HUMAN**	Calsyntenin-1	CLSTN1	Miscellaneous
**SLIK5_HUMAN**	SLIT and NTRK-like protein 5	SLITRK5	Miscellaneous
**OR2F2_HUMAN**	Olfactory receptor 2F2	OR2F2	Receptors
**OR6B1_HUMAN**	Olfactory receptor 6B1	OR6B1	Receptors
**O4F21_HUMAN**	Olfactory receptor 4F21	OR4F21	Receptors
**OR2A4_HUMAN**	Olfactory receptor 2A4	OR2A4	Receptors
**S1PR2_HUMAN**	Sphingosine 1-phosphate receptor 2	S1PR2	Receptors
**TNF15_HUMAN**	Tumor necrosis factor ligand superfamily member 15	TNFSF15	Unclassified
**UNC5C_HUMAN**	Netrin receptor UNC5C	UNC5C	Receptors
**CSPG5_HUMAN**	Chondroitin sulfate proteoglycan 5	CSPG5	Unclassified
**PCDH8_HUMAN**	Protocadherin-8	PCDH8	Miscellaneous
**OR5F1_HUMAN**	Olfactory receptor 5F1	OR5F1	Receptors
**OR6A2_HUMAN**	Olfactory receptor 6A2	OR6A2	Receptors
**I18RA_HUMAN**	Interleukin-18 receptor accessory protein	IL18RAP	Receptors
**5HT3B_HUMAN**	5-hydroxytryptamine receptor 3B	HTR3B	Transporters
**LYPD3_HUMAN**	Ly6/PLAUR domain-containing protein 3	LYPD3	Unclassified
**KCNK5_HUMAN**	Potassium channel subfamily K member 5	KCNK5	Transporters
**MPZL1_HUMAN**	Myelin protein zero-like protein 1	MPZL1	Miscellaneous
**ABCBB_HUMAN**	Bile salt export pump	ABCB11	Transporters
**OR2C1_HUMAN**	Olfactory receptor 2C1	OR2C1	Receptors
**CXB5_HUMAN**	Gap junction beta-5 protein	GJB5	Miscellaneous
**NPT2B_HUMAN**	Sodium-dependent phosphate transport protein 2B	SLC34A2	Transporters
**CXB6_HUMAN**	Gap junction beta-6 protein	GJB6	Miscellaneous
**CLD7_HUMAN**	Claudin-7	CLDN7	Miscellaneous
**ABCA1_HUMAN**	ATP-binding cassette sub-family A member 1	ABCA1	Transporters

**CLD9_HUMAN**	Claudin-9	CLDN9	Miscellaneous
**LPHN2_HUMAN**	Latrophilin-2	LPHN2	Receptors
**VNN1_HUMAN**	Pantetheinase	VNN1	Unclassified
**VNN2_HUMAN**	Vascular non-inflammatory molecule 2	VNN2	Unclassified
**GTR10_HUMAN**	Solute carrier family 2, facilitated glucose transporter member 10	SLC2A10	Transporters
**ADCY5_HUMAN**	Adenylate cyclase type 5	ADCY5	Enzymes
**NTR2_HUMAN**	Neurotensin receptor type 2	NTSR2	Receptors
**CRTAM_HUMAN**	Cytotoxic and regulatory T-cell molecule	CRTAM	Miscellaneous
**SEM4F_HUMAN**	Semaphorin-4F	SEMA4F	Miscellaneous
**GPR75_HUMAN**	Probable G-protein coupled receptor 75	GPR75	Receptors
**CLD1_HUMAN**	Claudin-1	CLDN1	Miscellaneous
**GLP2R_HUMAN**	Glucagon-like peptide 2 receptor	GLP2R	Receptors
**TSN13_HUMAN**	Tetraspanin-13	TSPAN13	Miscellaneous
**TSN15_HUMAN**	Tetraspanin-15	TSPAN15	Miscellaneous
**G6B_HUMAN**	Protein G6b	G6B	Unclassified
**LY66C_HUMAN**	Lymphocyte antigen 6 complex locus protein G6c	LY6G6C	Unclassified
**LY66D_HUMAN**	Lymphocyte antigen 6 complex locus protein G6d	LY6G6D	Unclassified
**MOT3_HUMAN**	Monocarboxylate transporter 3	SLC16A8	Transporters
**OR2H2_HUMAN**	Olfactory receptor 2H2	OR2H2	Receptors
**NCTR2_HUMAN**	Natural cytotoxicity triggering receptor 2	NCR2	Receptors
**BY55_HUMAN**	CD160 antigen	CD160	
**IGSF6_HUMAN**	Immunoglobulin superfamily member 6	IGSF6	Receptors
**S1PR4_HUMAN**	Sphingosine 1-phosphate receptor 4	S1PR4	Receptors
**RECK_HUMAN**	Reversion-inducing cysteine-rich protein with Kazal motifs	RECK	
**EGFR_HUMAN**	Epidermal growth factor receptor	EGFR	Receptors
**LDLR_HUMAN**	Low-density lipoprotein receptor	LDLR	Receptors
**EGF_HUMAN**	Pro-epidermal growth factor	EGF	Receptors
**TGFA_HUMAN**	Protransforming growth factor alpha	TGFA	Unclassified
**IL2RA_HUMAN**	Interleukin-2 receptor subunit alpha	IL2RA	Receptors
**CD4_HUMAN**	T-cell surface glycoprotein CD4	CD4	Unclassified
**CD8A_HUMAN**	T-cell surface glycoprotein CD8 alpha chain	CD8A	Unclassified
**PIGR_HUMAN**	Polymeric immunoglobulin receptor	PIGR	Receptors
**1B07_HUMAN**	HLA class I histocompatibility antigen, B-7 alpha chain	HLA-B	Miscellaneous
**1A68_HUMAN**	HLA class I histocompatibility antigen, A-68 alpha chain	HLA-A	Miscellaneous
**1A02_HUMAN**	HLA class I histocompatibility antigen, A-2 alpha chain	HLA-A	Miscellaneous
**HLAH_HUMAN**	Putative HLA class I histocompatibility antigen, alpha chain H	HLA-H	Miscellaneous
**DRA_HUMAN**	HLA class II histocompatibility antigen, DR alpha chain	HLA-DRA	Miscellaneous
**DQA2_HUMAN**	HLA class II histocompatibility antigen, DQ alpha 2 chain	HLA-DQA2	Miscellaneous
**DQA1_HUMAN**	HLA class II histocompatibility antigen, DQ alpha 1 chain	HLA-DQA1	Miscellaneous
**2B1F_HUMAN**	HLA class II histocompatibility antigen, DRB1-15 beta chain	HLA-DRB1	Miscellaneous

**2B13_HUMAN**	HLA class II histocompatibility antigen, DRB1-3 chain	HLA-DRB1	Miscellaneous
**DQB1_HUMAN**	HLA class II histocompatibility antigen, DQ beta 1 chain	HLA-DQB1	Miscellaneous
**ACHA_HUMAN**	Acetylcholine receptor subunit alpha	CHRNA1	Transporters
**GLPA_HUMAN**	Glycophorin-A	GYPA	Unclassified
**B3AT_HUMAN**	Band 3 anion transport protein	SLC4A1	Transporters
**TRGC2_HUMAN**	T-cell receptor gamma-2 chain C region	TRGC2	Receptors
**1B27_HUMAN**	HLA class I histocompatibility antigen, B-27 alpha chain	HLA-B	Miscellaneous
**OPSB_HUMAN**	Short-wave-sensitive opsin 1	OPN1SW	Receptors
**OPSR_HUMAN**	Long-wave-sensitive opsin 1	OPN1LW	Receptors
**OPSG_HUMAN**	Medium-wave-sensitive opsin 1	OPN1MW	Receptors
**PRIO_HUMAN**	Major prion protein	PRNP	Unclassified
**MAS_HUMAN**	Proto-oncogene Mas	MAS1	Receptors
**THY1_HUMAN**	Thy-1 membrane glycoprotein	THY1	
**1C03_HUMAN**	HLA class I histocompatibility antigen, Cw-3 alpha chain	HLA-C	Miscellaneous
**2B11_HUMAN**	HLA class II histocompatibility antigen, DRB1-1 beta chain	HLA-DRB1	Miscellaneous
**HG2A_HUMAN**	HLA class II histocompatibility antigen gamma chain	CD74	Unclassified
**CD3D_HUMAN**	T-cell surface glycoprotein CD3 delta chain	CD3D	
**1A03_HUMAN**	HLA class I histocompatibility antigen, A-3 alpha chain	HLA-A	Miscellaneous
**DPB1_HUMAN**	HLA class II histocompatibility antigen, DP beta 1 chain	HLA-DPB1	Miscellaneous
**ERBB2_HUMAN**	Receptor tyrosine-protein kinase erbB-2	ERBB2	Receptors
**NTRK1_HUMAN**	High affinity nerve growth factor receptor	NTRK1	Receptors
**CY24B_HUMAN**	Cytochrome b-245 heavy chain	CYBB	Enzymes
**RPN1_HUMAN**	Dolichyl-diphosphooligosaccharide--protein glycosyltransferase subunit 1	RPN1	Enzymes
**GLPC_HUMAN**	Glycophorin-C	GYPC	Unclassified
**AT1A1_HUMAN**	Sodium/potassium-transporting ATPase subunit alpha-1	ATP1A1	Transporters
**AT1B1_HUMAN**	Sodium/potassium-transporting ATPase subunit beta-1	ATP1B1	Transporters
**A4_HUMAN**	Amyloid beta A4 protein	APP	Unclassified
**ITB3_HUMAN**	Integrin beta-3	ITGB3	Receptors
**ITB2_HUMAN**	Integrin beta-2	ITGB2	Receptors
**PPBT_HUMAN**	Alkaline phosphatase, tissue-nonspecific isozyme	ALPL	
**PPB1_HUMAN**	Alkaline phosphatase, placental type	ALPP	
**ICAM1_HUMAN**	Intercellular adhesion molecule 1	ICAM1	Unclassified
**1A24_HUMAN**	HLA class I histocompatibility antigen, A-24 alpha chain	HLA-A	Miscellaneous
**DQB2_HUMAN**	HLA class II histocompatibility antigen, DQ beta 2 chain	HLA-DQB2	Unclassified
**ITB1_HUMAN**	Integrin beta-1	ITGB1	Receptors
**CD1A_HUMAN**	T-cell surface glycoprotein CD1a	CD1A	Receptors
**CD5_HUMAN**	T-cell surface glycoprotein CD5	CD5	Unclassified
**INSR_HUMAN**	Insulin receptor	INSR	Receptors
**DOA_HUMAN**	HLA class II histocompatibility antigen, DO alpha chain	HLA-DOA	Miscellaneous
**CD2_HUMAN**	T-cell surface antigen CD2	CD2	Unclassified

**CEAM5_HUMAN**	Carcinoembryonic antigen-related cell adhesion molecule 5	CEACAM5	Miscellaneous
**ITAV_HUMAN**	Integrin alpha-V	ITGAV	Receptors
**LIPL_HUMAN**	Lipoprotein lipase	LPL	
**PERT_HUMAN**	Thyroid peroxidase	TPO	Enzymes
**TRBM_HUMAN**	Thrombomodulin	THBD	Unclassified
**ASGR1_HUMAN**	Asialoglycoprotein receptor 1	ASGR1	Receptors
**ASGR2_HUMAN**	Asialoglycoprotein receptor 2	ASGR2	Receptors
**CSF1R_HUMAN**	Macrophage colony-stimulating factor 1 receptor	CSF1R	Receptors
**GP1BA_HUMAN**	Platelet glycoprotein Ib alpha chain	GP1BA	Miscellaneous
**ACHG_HUMAN**	Acetylcholine receptor subunit gamma	CHRNG	Transporters
**ADRB2_HUMAN**	Beta-2 adrenergic receptor	ADRB2	Receptors
**UROM_HUMAN**	Uromodulin	UMOD	Unclassified
**RET_HUMAN**	Proto-oncogene tyrosine-protein kinase receptor Ret	RET	Receptors
**CXB1_HUMAN**	Gap junction beta-1 protein	GJB1	Miscellaneous
**IGF1R_HUMAN**	Insulin-like growth factor 1 receptor	IGF1R	Receptors
**OPSD_HUMAN**	Rhodopsin	RHO	Receptors
**TNR16_HUMAN**	Tumor necrosis factor receptor superfamily member 16	NGFR	Receptors
**ACM2_HUMAN**	Muscarinic acetylcholine receptor M2	CHRM2	Receptors
**ACM4_HUMAN**	Muscarinic acetylcholine receptor M4	CHRM4	Receptors
**DAF_HUMAN**	Complement decay-accelerating factor	CD55	
**MDR1_HUMAN**	Multidrug resistance protein 1	ABCB1	Transporters
**4F2_HUMAN**	4F2 cell-surface antigen heavy chain	SLC3A2	Transporters
**SYPH_HUMAN**	Synaptophysin	SYP	Miscellaneous
**NEP_HUMAN**	Neprilysin	MME	Enzymes
**ITA2B_HUMAN**	Integrin alpha-IIb	ITGA2B	Receptors
**CD14_HUMAN**	Monocyte differentiation antigen CD14	CD14	
**PTPRC_HUMAN**	Receptor-type tyrosine-protein phosphatase C	PTPRC	Receptors
**MET_HUMAN**	Hepatocyte growth factor receptor	MET	Receptors
**TRFM_HUMAN**	Melanotransferrin	MFI2	
**ADRB1_HUMAN**	Beta-1 adrenergic receptor	ADRB1	Receptors
**FCG3A_HUMAN**	Low affinity immunoglobulin gamma Fc region receptor III-A	FCGR3A	Receptors
**ITA5_HUMAN**	Integrin alpha-5	ITGA5	Receptors
**STS_HUMAN**	Steryl-sulfatase	STS	Enzymes
**IL6RA_HUMAN**	Interleukin-6 receptor subunit alpha	IL6R	Receptors
**5HT1A_HUMAN**	5-hydroxytryptamine receptor 1A	HTR1A	Receptors
**ACM5_HUMAN**	Muscarinic acetylcholine receptor M5	CHRM5	Receptors
**ADA2A_HUMAN**	Alpha-2A adrenergic receptor	ADRA2A	Receptors
**ROS1_HUMAN**	Proto-oncogene tyrosine-protein kinase ROS	ROS1	Receptors
**CD63_HUMAN**	CD63 antigen	CD63	Miscellaneous
**PKHD1_HUMAN**	Fibrocystin	PKHD1	Unclassified

**P3_HUMAN**	P3 protein	SLC10A3	Transporters
**CD48_HUMAN**	CD48 antigen	CD48	Unclassified
**CD7_HUMAN**	T-cell antigen CD7	CD7	Unclassified
**CSF1_HUMAN**	Macrophage colony-stimulating factor 1	CSF1	Unclassified
**PGFRB_HUMAN**	Platelet-derived growth factor receptor beta	PDGFRB	Receptors
**CD3G_HUMAN**	T-cell surface glycoprotein CD3 gamma chain	CD3G	Unclassified
**TACD2_HUMAN**	Tumor-associated calcium signal transducer 2	TACSTD2	Unclassified
**LPH_HUMAN**	Lactase-phlorizin hydrolase	LCT	Enzymes
**PPBI_HUMAN**	Intestinal-type alkaline phosphatase	ALPI	
**FURIN_HUMAN**	Furin	FURIN	Enzymes
**OR4A8_HUMAN**	Putative olfactory receptor 4A8	OR4A8P	Receptors
**O5AL1_HUMAN**	Olfactory receptor 5AL1	OR5AL1	Receptors
**OR4Q2_HUMAN**	Olfactory receptor 4Q2	OR4Q2	Receptors
**OR5G3_HUMAN**	Olfactory receptor 5G3	OR5G3	Receptors
**O5AC1_HUMAN**	Olfactory receptor 5AC1	OR5AC1	Receptors
**O10J4_HUMAN**	Olfactory receptor 10J4	OR10J4	Receptors
**OR4E1_HUMAN**	Olfactory receptor 4E1	OR4E1	Receptors
**O52Z1_HUMAN**	Olfactory receptor 52Z1	OR52Z1	Receptors
**LIGO3_HUMAN**	Leucine-rich repeat and immunoglobulin-like domain-containing nogo receptor-interacting protein 3	LINGO3	Miscellaneous
**OR8U8_HUMAN**	Olfactory receptor 8U8	OR8U8	Receptors
**OR8U9_HUMAN**	Olfactory receptor 8U9	OR8U9	Receptors
**OR9G9_HUMAN**	Olfactory receptor 9G9	OR9G9	Receptors
**OR2T7_HUMAN**	Olfactory receptor 2T7	OR2T7	Receptors
**O56A5_HUMAN**	Olfactory receptor 56A5	OR56A5	Receptors
**ELFN1_HUMAN**	Protein ELFN1	ELFN1	Miscellaneous
**ZH11B_HUMAN**	Probable palmitoyltransferase ZDHHC11B	ZDHHC11B	Enzymes
**CFC1_HUMAN**	Cryptic protein	CFC1	Unclassified
**TPBGL_HUMAN**	Trophoblast glycoprotein-like	TPBGL	Miscellaneous
**1A32_HUMAN**	HLA class I histocompatibility antigen, A-32 alpha chain	HLA-A	Miscellaneous
**1A69_HUMAN**	HLA class I histocompatibility antigen, A-69 alpha chain	HLA-A	Miscellaneous
**1B58_HUMAN**	HLA class I histocompatibility antigen, B-58 alpha chain	HLA-B	Miscellaneous
**1C07_HUMAN**	HLA class I histocompatibility antigen, Cw-7 alpha chain	HLA-C	Miscellaneous
**PTPRF_HUMAN**	Receptor-type tyrosine-protein phosphatase F	PTPRF	Receptors
**TFPI1_HUMAN**	Tissue factor pathway inhibitor	TFPI	
**PPBN_HUMAN**	Alkaline phosphatase, placental-like	ALPPL2	
**KIT_HUMAN**	Mast/stem cell growth factor receptor Kit	KIT	Receptors
**CD28_HUMAN**	T-cell-specific surface glycoprotein CD28	CD28	Unclassified
**GHR_HUMAN**	Growth hormone receptor	GHR	Receptors
**CD8B_HUMAN**	T-cell surface glycoprotein CD8 beta chain	CD8B	Unclassified
**CD37_HUMAN**	Leukocyte antigen CD37	CD37	Miscellaneous

**PPAL_HUMAN**	Lysosomal acid phosphatase	ACP2	Enzymes
**GTR1_HUMAN**	Solute carrier family 2, facilitated glucose transporter member 1	SLC2A1	Transporters
**GTR2_HUMAN**	Solute carrier family 2, facilitated glucose transporter member 2	SLC2A2	Transporters
**GTR3_HUMAN**	Solute carrier family 2, facilitated glucose transporter member 3	SLC2A3	Transporters
**ITAM_HUMAN**	Integrin alpha-M	ITGAM	Receptors
**ACM1_HUMAN**	Muscarinic acetylcholine receptor M1	CHRM1	Receptors
**ACHB_HUMAN**	Acetylcholine receptor subunit beta	CHRNB1	Transporters
**LAMP1_HUMAN**	Lysosome-associated membrane glycoprotein 1	LAMP1	Receptors
**FGFR1_HUMAN**	Fibroblast growth factor receptor 1	FGFR1	Receptors
**MPRI_HUMAN**	Cation-independent mannose-6-phosphate receptor	IGF2R	Unclassified
**CD20_HUMAN**	B-lymphocyte antigen CD20	MS4A1	Miscellaneous
**CD79A_HUMAN**	B-cell antigen receptor complex-associated protein alpha chain	CD79A	Unclassified
**FCGR1_HUMAN**	High affinity immunoglobulin gamma Fc receptor I	FCGR1A	Receptors
**FCG2A_HUMAN**	Low affinity immunoglobulin gamma Fc region receptor II-a	FCGR2A	Receptors
**FCERA_HUMAN**	High affinity immunoglobulin epsilon receptor subunit alpha	FCER1A	Receptors
**ACE_HUMAN**	Angiotensin-converting enzyme	ACE	Enzymes
**CADH1_HUMAN**	Cadherin-1	CDH1	Miscellaneous
**GP1BB_HUMAN**	Platelet glycoprotein Ib beta chain	GP1BB	Miscellaneous
**TDGF1_HUMAN**	Teratocarcinoma-derived growth factor 1	TDGF1	Unclassified
**LAMP2_HUMAN**	Lysosome-associated membrane glycoprotein 2	LAMP2	Receptors
**NCAM1_HUMAN**	Neural cell adhesion molecule 1	NCAM1	Miscellaneous
**ICAM2_HUMAN**	Intercellular adhesion molecule 2	ICAM2	Unclassified
**ITA4_HUMAN**	Integrin alpha-4	ITGA4	Receptors
**AT1A3_HUMAN**	Sodium/potassium-transporting ATPase subunit alpha-3	ATP1A3	Transporters
**CEAM1_HUMAN**	Carcinoembryonic antigen-related cell adhesion molecule 1	CEACAM1	Miscellaneous
**TF_HUMAN**	Tissue factor	F3	Unclassified
**1A11_HUMAN**	HLA class I histocompatibility antigen, A-11 alpha chain	HLA-A	Miscellaneous
**HLAE_HUMAN**	HLA class I histocompatibility antigen, alpha chain E	HLA-E	Miscellaneous
**2B14_HUMAN**	HLA class II histocompatibility antigen, DRB1-4 beta chain	HLA-DRB1	Miscellaneous
**2B17_HUMAN**	HLA class II histocompatibility antigen, DRB1-7 beta chain	HLA-DRB1	Miscellaneous
**DRB4_HUMAN**	HLA class II histocompatibility antigen, DR beta 4 chain	HLA-DRB4	Miscellaneous
**DOB_HUMAN**	HLA class II histocompatibility antigen, DO beta chain	HLA-DOB	Miscellaneous
**SC5A1_HUMAN**	Sodium/glucose cotransporter 1	SLC5A1	Transporters
**ADRB3_HUMAN**	Beta-3 adrenergic receptor	ADRB3	Receptors
**CD59_HUMAN**	CD59 glycoprotein	CD59	
**LYAM1_HUMAN**	L-selectin	SELL	Receptors
**FOLR2_HUMAN**	Folate receptor beta	FOLR2	Unclassified
**CBPM_HUMAN**	Carboxypeptidase M	CPM	Enzymes
**AT1B2_HUMAN**	Sodium/potassium-transporting ATPase subunit beta-2	ATP1B2	Transporters
**DRD2_HUMAN**	D(2) dopamine receptor	DRD2	Receptors

**INSRR_HUMAN**	Insulin receptor-related protein	INSRR	Receptors
**GTR4_HUMAN**	Solute carrier family 2, facilitated glucose transporter member 4	SLC2A4	Transporters
**TYRO_HUMAN**	Tyrosinase	TYR	Enzymes
**IL1R1_HUMAN**	Interleukin-1 receptor type 1	IL1R1	Receptors
**IL2RB_HUMAN**	Interleukin-2 receptor subunit beta	IL2RB	Receptors
**GBRA1_HUMAN**	Gamma-aminobutyric acid receptor subunit alpha-1	GABRA1	Transporters
**AMPN_HUMAN**	Aminopeptidase N	ANPEP	Enzymes
**PVR_HUMAN**	Poliovirus receptor	PVR	Receptors
**INGR1_HUMAN**	Interferon gamma receptor 1	IFNGR1	Receptors
**FOLR1_HUMAN**	Folate receptor alpha	FOLR1	Unclassified
**CD19_HUMAN**	B-lymphocyte antigen CD19	CD19	Unclassified
**CSF2R_HUMAN**	Granulocyte-macrophage colony-stimulating factor receptor subunit alpha	CSF2RA	Receptors
**AREG_HUMAN**	Amphiregulin	AREG	Unclassified
**MCP_HUMAN**	Membrane cofactor protein	CD46	Unclassified
**CD1E_HUMAN**	T-cell surface glycoprotein CD1e, membrane-associated	CD1E	Receptors
**CD1D_HUMAN**	Antigen-presenting glycoprotein CD1d	CD1D	Receptors
**MUC1_HUMAN**	Mucin-1	MUC1	Unclassified
**ANPRA_HUMAN**	Atrial natriuretic peptide receptor 1	NPR1	Receptors
**CD44_HUMAN**	CD44 antigen	CD44	Unclassified
**LYAM3_HUMAN**	P-selectin	SELP	Receptors
**ITB4_HUMAN**	Integrin beta-4	ITGB4	Receptors
**LEUK_HUMAN**	Leukosialin	SPN	Unclassified
**1A30_HUMAN**	HLA class I histocompatibility antigen, A-30 alpha chain	HLA-A	Miscellaneous
**1A31_HUMAN**	HLA class I histocompatibility antigen, A-31 alpha chain	HLA-A	Miscellaneous
**1A33_HUMAN**	HLA class I histocompatibility antigen, A-33 alpha chain	HLA-A	Miscellaneous
**PGFRA_HUMAN**	Platelet-derived growth factor receptor alpha	PDGFRA	Receptors
**PECA1_HUMAN**	Platelet endothelial cell adhesion molecule	PECAM1	Unclassified
**CTLA4_HUMAN**	Cytotoxic T-lymphocyte protein 4	CTLA4	Unclassified
**EPCAM_HUMAN**	Epithelial cell adhesion molecule	EPCAM	Unclassified
**DPEP1_HUMAN**	Dipeptidase 1	DPEP1	Enzymes
**PRLR_HUMAN**	Prolactin receptor	PRLR	Receptors
**TSHR_HUMAN**	Thyrotropin receptor	TSHR	Receptors
**LYAM2_HUMAN**	E-selectin	SELE	Receptors
**CD36_HUMAN**	Platelet glycoprotein 4	CD36	Receptors
**IL7RA_HUMAN**	Interleukin-7 receptor subunit alpha	IL7R	Receptors
**INAR1_HUMAN**	Interferon alpha/beta receptor 1	IFNAR1	Receptors
**ITA2_HUMAN**	Integrin alpha-2	ITGA2	Receptors
**CXA1_HUMAN**	Gap junction alpha-1 protein	GJA1	Miscellaneous
**ANPRC_HUMAN**	Atrial natriuretic peptide receptor 3	NPR3	Receptors
**TYRP1_HUMAN**	5,6-dihydroxyindole-2-carboxylic acid oxidase	TYRP1	Unclassified

**HLAG_HUMAN**	HLA class I histocompatibility antigen, alpha chain G	HLA-G	Miscellaneous
**ACHB2_HUMAN**	Neuronal acetylcholine receptor subunit beta-2	CHRNB2	Transporters
**EGLN_HUMAN**	Endoglin	ENG	Unclassified
**CR1_HUMAN**	Complement receptor type 1	CR1	Unclassified
**VGFR1_HUMAN**	Vascular endothelial growth factor receptor 1	FLT1	Receptors
**ITB5_HUMAN**	Integrin beta-5	ITGB5	Receptors
**ADA2B_HUMAN**	Alpha-2B adrenergic receptor	ADRA2B	Receptors
**PTPRA_HUMAN**	Receptor-type tyrosine-protein phosphatase alpha	PTPRA	Receptors
**1A25_HUMAN**	HLA class I histocompatibility antigen, A-25 alpha chain	HLA-A	Miscellaneous
**1B37_HUMAN**	HLA class I histocompatibility antigen, B-37 alpha chain	HLA-B	Miscellaneous
**1B51_HUMAN**	HLA class I histocompatibility antigen, B-51 alpha chain	HLA-B	Miscellaneous
**1B57_HUMAN**	HLA class I histocompatibility antigen, B-57 alpha chain	HLA-B	Miscellaneous
**GBRB1_HUMAN**	Gamma-aminobutyric acid receptor subunit beta-1	GABRB1	Transporters
**GBRG2_HUMAN**	Gamma-aminobutyric acid receptor subunit gamma-2	GABRG2	Transporters
**ITB6_HUMAN**	Integrin beta-6	ITGB6	Receptors
**LAG3_HUMAN**	Lymphocyte activation gene 3 protein	LAG3	Unclassified
**ADA2C_HUMAN**	Alpha-2C adrenergic receptor	ADRA2C	Receptors
**SDC1_HUMAN**	Syndecan-1	SDC1	Receptors
**AMD_HUMAN**	Peptidyl-glycine alpha-amidating monooxygenase	PAM	Enzymes
**CADH2_HUMAN**	Cadherin-2	CDH2	Miscellaneous
**TSN8_HUMAN**	Tetraspanin-8	TSPAN8	Miscellaneous
**EPOR_HUMAN**	Erythropoietin receptor	EPOR	Receptors
**LFA3_HUMAN**	Lymphocyte function-associated antigen 3	CD58	Unclassified
**VCAM1_HUMAN**	Vascular cell adhesion protein 1	VCAM1	Unclassified
**CD53_HUMAN**	Leukocyte surface antigen CD53	CD53	Miscellaneous
**TNR1A_HUMAN**	Tumor necrosis factor receptor superfamily member 1A	TNFRSF1A	Receptors
**GGT1_HUMAN**	Gamma-glutamyltranspeptidase 1	GGT1	Unclassified
**SL9A1_HUMAN**	Sodium/hydrogen exchanger 1	SLC9A1	Transporters
**CR2_HUMAN**	Complement receptor type 2	CR2	Unclassified
**DPA1_HUMAN**	HLA class II histocompatibility antigen, DP alpha 1 chain	HLA-DPA1	Miscellaneous
**2B1B_HUMAN**	HLA class II histocompatibility antigen, DRB1-11 beta chain	HLA-DRB1	Miscellaneous
**CD33_HUMAN**	Myeloid cell surface antigen CD33	CD33	Miscellaneous
**CD22_HUMAN**	B-cell receptor CD22	CD22	Miscellaneous
**ACM3_HUMAN**	Muscarinic acetylcholine receptor M3	CHRM3	Receptors
**TNR1B_HUMAN**	Tumor necrosis factor receptor superfamily member 1B	TNFRSF1B	Receptors
**ANPRB_HUMAN**	Atrial natriuretic peptide receptor 2	NPR2	Receptors
**MPRD_HUMAN**	Cation-dependent mannose-6-phosphate receptor	M6PR	Unclassified
**ITAL_HUMAN**	Integrin alpha-L	ITGAL	Receptors
**ITAX_HUMAN**	Integrin alpha-X	ITGAX	Receptors
**EFNA1_HUMAN**	Ephrin-A1	EFNA1	Unclassified

**MAG_HUMAN**	Myelin-associated glycoprotein	MAG	Miscellaneous
**MDR3_HUMAN**	Multidrug resistance protein 3	ABCB4	Transporters
**NK2R_HUMAN**	Substance-K receptor	TACR2	Receptors
**S1PR1_HUMAN**	Sphingosine 1-phosphate receptor 1	S1PR1	Receptors
**FPR1_HUMAN**	fMet-Leu-Phe receptor	FPR1	Receptors
**CNR1_HUMAN**	Cannabinoid receptor 1	CNR1	Receptors
**SCF_HUMAN**	Kit ligand	KITLG	Unclassified
**5NTD_HUMAN**	5'-nucleotidase	NT5E	Enzymes
**EPHA1_HUMAN**	Ephrin type-A receptor 1	EPHA1	Receptors
**DRD1_HUMAN**	D(1A) dopamine receptor	DRD1	Receptors
**C5AR1_HUMAN**	C5a anaphylatoxin chemotactic receptor 1	C5AR1	Receptors
**TA2R_HUMAN**	Thromboxane A2 receptor	TBXA2R	Receptors
**ZP3_HUMAN**	Zona pellucida sperm-binding protein 3	ZP3	Unclassified
**MSRE_HUMAN**	Macrophage scavenger receptor types I and II	MSR1	Receptors
**FGFR2_HUMAN**	Fibroblast growth factor receptor 2	FGFR2	Receptors
**ERBB3_HUMAN**	Receptor tyrosine-protein kinase erbB-3	ERBB3	Receptors
**DRD4_HUMAN**	D(4) dopamine receptor	DRD4	Receptors
**DRD5_HUMAN**	D(1B) dopamine receptor	DRD5	Receptors
**CD9_HUMAN**	CD9 antigen	CD9	Miscellaneous
**KCNA3_HUMAN**	Potassium voltage-gated channel subfamily A member 3	KCNA3	Transporters
**CADH3_HUMAN**	Cadherin-3	CDH3	Miscellaneous
**ACES_HUMAN**	Acetylcholinesterase	ACHE	Unclassified
**ENPP1_HUMAN**	Ectonucleotide pyrophosphatase/phosphodiesterase family member 1	ENPP1	Enzymes
**FGFR4_HUMAN**	Fibroblast growth factor receptor 4	FGFR4	Receptors
**FGFR3_HUMAN**	Fibroblast growth factor receptor 3	FGFR3	Receptors
**GTR5_HUMAN**	Solute carrier family 2, facilitated glucose transporter member 5	SLC2A5	Transporters
**CAH4_HUMAN**	Carbonic anhydrase 4	CA4	Enzymes
**EVI2A_HUMAN**	Protein EVI2A	EVI2A	Unclassified
**LSHR_HUMAN**	Lutropin-choriogonadotropic hormone receptor	LHCGR	Receptors
**MRC1_HUMAN**	Macrophage mannose receptor 1	MRC1	Receptors
**ITA6_HUMAN**	Integrin alpha-6	ITGA6	Receptors
**KELL_HUMAN**	Kell blood group glycoprotein	KEL	Enzymes
**GLRA1_HUMAN**	Glycine receptor subunit alpha-1	GLRA1	Transporters
**GLRA2_HUMAN**	Glycine receptor subunit alpha-2	GLRA2	Transporters
**PTPRB_HUMAN**	Receptor-type tyrosine-protein phosphatase beta	PTPRB	Receptors
**PTPRD_HUMAN**	Receptor-type tyrosine-protein phosphatase delta	PTPRD	Receptors
**PTPRG_HUMAN**	Receptor-type tyrosine-protein phosphatase gamma	PTPRG	Receptors
**PTPRZ_HUMAN**	Receptor-type tyrosine-protein phosphatase zeta	PTPRZ1	Receptors
**TNFL4_HUMAN**	Tumor necrosis factor ligand superfamily member 4	TNFSF4	Unclassified
**OMGP_HUMAN**	Oligodendrocyte-myelin glycoprotein	OMG	

**AT2B4_HUMAN**	Plasma membrane calcium-transporting ATPase 4	ATP2B4	Transporters
**PRPH2_HUMAN**	Peripherin-2	PRPH2	Miscellaneous
**FSHR_HUMAN**	Follicle-stimulating hormone receptor	FSHR	Receptors
**SC6A2_HUMAN**	Sodium-dependent noradrenaline transporter	SLC6A2	Transporters
**GBRR1_HUMAN**	Gamma-aminobutyric acid receptor subunit rho-1	GABRR1	Transporters
**FCAR_HUMAN**	Immunoglobulin alpha Fc receptor	FCAR	Unclassified
**IL4RA_HUMAN**	Interleukin-4 receptor subunit alpha	IL4R	Receptors
**EDNRB_HUMAN**	Endothelin B receptor	EDNRB	Receptors
**HRH2_HUMAN**	Histamine H2 receptor	HRH2	Receptors
**CXCR1_HUMAN**	C-X-C chemokine receptor type 1	CXCR1	Receptors
**CXCR2_HUMAN**	C-X-C chemokine receptor type 2	CXCR2	Receptors
**CD24_HUMAN**	Signal transducer CD24	CD24	
**FPR3_HUMAN**	N-formyl peptide receptor 3	FPR3	Receptors
**FPR2_HUMAN**	N-formyl peptide receptor 2	FPR2	Receptors
**GUC2C_HUMAN**	Heat-stable enterotoxin receptor	GUCY2C	Receptors
**ADA1D_HUMAN**	Alpha-1D adrenergic receptor	ADRA1D	Receptors
**EDNRA_HUMAN**	Endothelin-1 receptor	EDNRA	Receptors
**NK1R_HUMAN**	Substance-P receptor	TACR1	Receptors
**PTAFR_HUMAN**	Platelet-activating factor receptor	PTAFR	Receptors
**ACKR3_HUMAN**	Atypical chemokine receptor 3	ACKR3	Receptors
**PAR1_HUMAN**	Proteinase-activated receptor 1	F2R	Receptors
**MYP0_HUMAN**	Myelin protein P0	MPZ	Miscellaneous
**TNR6_HUMAN**	Tumor necrosis factor receptor superfamily member 6	FAS	Receptors
**NPY1R_HUMAN**	Neuropeptide Y receptor type 1	NPY1R	Receptors
**TNR5_HUMAN**	Tumor necrosis factor receptor superfamily member 5	CD40	Receptors
**ITA3_HUMAN**	Integrin alpha-3	ITGA3	Receptors
**ITB7_HUMAN**	Integrin beta-7	ITGB7	Receptors
**ITB8_HUMAN**	Integrin beta-8	ITGB8	Receptors
**NKG2A_HUMAN**	NKG2-A/NKG2-B type II integral membrane protein	KLRC1	Receptors
**NKG2C_HUMAN**	NKG2-C type II integral membrane protein	KLRC2	Receptors
**NKG2D_HUMAN**	NKG2-D type II integral membrane protein	KLRK1	Receptors
**CD27_HUMAN**	CD27 antigen	CD27	Receptors
**IL3RA_HUMAN**	Interleukin-3 receptor subunit alpha	IL3RA	Receptors
**CNTFR_HUMAN**	Ciliary neurotrophic factor receptor subunit alpha	CNTFR	Receptors
**AVR2A_HUMAN**	Activin receptor type-2A	ACVR2A	Receptors
**DPP4_HUMAN**	Dipeptidyl peptidase 4	DPP4	
**CD82_HUMAN**	CD82 antigen	CD82	Miscellaneous
**IL1R2_HUMAN**	Interleukin-1 receptor type 2	IL1R2	Receptors
**DMA_HUMAN**	HLA class II histocompatibility antigen, DM alpha chain	HLA-DMA	Miscellaneous
**DMB_HUMAN**	HLA class II histocompatibility antigen, DM beta chain	HLA-DMB	Miscellaneous

**5HT1D_HUMAN**	5-hydroxytryptamine receptor 1D	HTR1D	Receptors
**5HT1B_HUMAN**	5-hydroxytryptamine receptor 1B	HTR1B	Receptors
**5HT2A_HUMAN**	5-hydroxytryptamine receptor 2A	HTR2A	Receptors
**5HT2C_HUMAN**	5-hydroxytryptamine receptor 2C	HTR2C	Receptors
**NMBR_HUMAN**	Neuromedin-B receptor	NMBR	Receptors
**GBRB3_HUMAN**	Gamma-aminobutyric acid receptor subunit beta-3	GABRB3	Transporters
**GBRR2_HUMAN**	Gamma-aminobutyric acid receptor subunit rho-2	GABRR2	Transporters
**5HT1E_HUMAN**	5-hydroxytryptamine receptor 1E	HTR1E	Receptors
**PTPRM_HUMAN**	Receptor-type tyrosine-protein phosphatase mu	PTPRM	Receptors
**CD34_HUMAN**	Hematopoietic progenitor cell antigen CD34	CD34	Unclassified
**CD38_HUMAN**	ADP-ribosyl cyclase/cyclic ADP-ribose hydrolase 1	CD38	Enzymes
**TNR8_HUMAN**	Tumor necrosis factor receptor superfamily member 8	TNFRSF8	Receptors
**CD1B_HUMAN**	T-cell surface glycoprotein CD1b	CD1B	Receptors
**CD1C_HUMAN**	T-cell surface glycoprotein CD1c	CD1C	Receptors
**CXB2_HUMAN**	Gap junction beta-2 protein	GJB2	Miscellaneous
**AA2AR_HUMAN**	Adenosine receptor A2a	ADORA2A	Receptors
**AA2BR_HUMAN**	Adenosine receptor A2b	ADORA2B	Receptors
**EPHA2_HUMAN**	Ephrin type-A receptor 2	EPHA2	Receptors
**EPHA3_HUMAN**	Ephrin type-A receptor 3	EPHA3	Receptors
**EPHA8_HUMAN**	Ephrin type-A receptor 8	EPHA8	Receptors
**EPHB2_HUMAN**	Ephrin type-B receptor 2	EPHB2	Receptors
**NK3R_HUMAN**	Neuromedin-K receptor	TACR3	Receptors
**LTK_HUMAN**	Leukocyte tyrosine kinase receptor	LTK	Receptors
**CD40L_HUMAN**	CD40 ligand	CD40LG	Unclassified
**AQP1_HUMAN**	Aquaporin-1	AQP1	Transporters
**CD6_HUMAN**	T-cell differentiation antigen CD6	CD6	Unclassified
**MIP_HUMAN**	Lens fiber major intrinsic protein	MIP	Transporters
**T4S1_HUMAN**	Transmembrane 4 L6 family member 1	TM4SF1	Miscellaneous
**BKRB2_HUMAN**	B2 bradykinin receptor	BDKRB2	Receptors
**1A01_HUMAN**	HLA class I histocompatibility antigen, A-1 alpha chain	HLA-A	Miscellaneous
**1A23_HUMAN**	HLA class I histocompatibility antigen, A-23 alpha chain	HLA-A	Miscellaneous
**1A26_HUMAN**	HLA class I histocompatibility antigen, A-26 alpha chain	HLA-A	Miscellaneous
**1A34_HUMAN**	HLA class I histocompatibility antigen, A-34 alpha chain	HLA-A	Miscellaneous
**1A36_HUMAN**	HLA class I histocompatibility antigen, A-36 alpha chain	HLA-A	Miscellaneous
**1A43_HUMAN**	HLA class I histocompatibility antigen, A-43 alpha chain	HLA-A	Miscellaneous
**1A66_HUMAN**	HLA class I histocompatibility antigen, A-66 alpha chain	HLA-A	Miscellaneous
**1A74_HUMAN**	HLA class I histocompatibility antigen, A-74 alpha chain	HLA-A	Miscellaneous
**1B08_HUMAN**	HLA class I histocompatibility antigen, B-8 alpha chain	HLA-B	Miscellaneous
**1B13_HUMAN**	HLA class I histocompatibility antigen, B-13 alpha chain	HLA-B	Miscellaneous
**1B14_HUMAN**	HLA class I histocompatibility antigen, B-14 alpha chain	HLA-B	Miscellaneous

**1B15_HUMAN**	HLA class I histocompatibility antigen, B-15 alpha chain	HLA-B	Miscellaneous
**1B18_HUMAN**	HLA class I histocompatibility antigen, B-18 alpha chain	HLA-B	Miscellaneous
**1B39_HUMAN**	HLA class I histocompatibility antigen, B-39 alpha chain	HLA-B	Miscellaneous
**1B41_HUMAN**	HLA class I histocompatibility antigen, B-41 alpha chain	HLA-B	Miscellaneous
**1B42_HUMAN**	HLA class I histocompatibility antigen, B-42 alpha chain	HLA-B	Miscellaneous
**1B44_HUMAN**	HLA class I histocompatibility antigen, B-44 alpha chain	HLA-B	Miscellaneous
**1B45_HUMAN**	HLA class I histocompatibility antigen, B-45 alpha chain	HLA-B	Miscellaneous
**1B46_HUMAN**	HLA class I histocompatibility antigen, B-46 alpha chain	HLA-B	Miscellaneous
**1B47_HUMAN**	HLA class I histocompatibility antigen, B-47 alpha chain	HLA-B	Miscellaneous
**1B48_HUMAN**	HLA class I histocompatibility antigen, B-48 alpha chain	HLA-B	Miscellaneous
**1B49_HUMAN**	HLA class I histocompatibility antigen, B-49 alpha chain	HLA-B	Miscellaneous
**1B50_HUMAN**	HLA class I histocompatibility antigen, B-50 alpha chain	HLA-B	Miscellaneous
**1B52_HUMAN**	HLA class I histocompatibility antigen, B-52 alpha chain	HLA-B	Miscellaneous
**1B53_HUMAN**	HLA class I histocompatibility antigen, B-53 alpha chain	HLA-B	Miscellaneous
**1B54_HUMAN**	HLA class I histocompatibility antigen, B-54 alpha chain	HLA-B	Miscellaneous
**1B55_HUMAN**	HLA class I histocompatibility antigen, B-55 alpha chain	HLA-B	Miscellaneous
**1B56_HUMAN**	HLA class I histocompatibility antigen, B-56 alpha chain	HLA-B	Miscellaneous
**1B78_HUMAN**	HLA class I histocompatibility antigen, B-78 alpha chain	HLA-B	Miscellaneous
**1C01_HUMAN**	HLA class I histocompatibility antigen, Cw-1 alpha chain	HLA-C	Miscellaneous
**1C02_HUMAN**	HLA class I histocompatibility antigen, Cw-2 alpha chain	HLA-C	Miscellaneous
**1C04_HUMAN**	HLA class I histocompatibility antigen, Cw-4 alpha chain	HLA-C	Miscellaneous
**1C08_HUMAN**	HLA class I histocompatibility antigen, Cw-8 alpha chain	HLA-C	Miscellaneous
**1C12_HUMAN**	HLA class I histocompatibility antigen, Cw-12 alpha chain	HLA-C	Miscellaneous
**1C14_HUMAN**	HLA class I histocompatibility antigen, Cw-14 alpha chain	HLA-C	Miscellaneous
**HLAF_HUMAN**	HLA class I histocompatibility antigen, alpha chain F	HLA-F	Miscellaneous
**1A29_HUMAN**	HLA class I histocompatibility antigen, A-29 alpha chain	HLA-A	Miscellaneous
**V2R_HUMAN**	Vasopressin V2 receptor	AVPR2	Receptors
**UFO_HUMAN**	Tyrosine-protein kinase receptor UFO	AXL	Receptors
**SC6A1_HUMAN**	Sodium- and chloride-dependent GABA transporter 1	SLC6A1	Transporters
**ACHA5_HUMAN**	Neuronal acetylcholine receptor subunit alpha-5	CHRNA5	Transporters
**AA1R_HUMAN**	Adenosine receptor A1	ADORA1	Receptors
**GRPR_HUMAN**	Gastrin-releasing peptide receptor	GRPR	Receptors
**AGTR1_HUMAN**	Type-1 angiotensin II receptor	AGTR1	Receptors
**OXYR_HUMAN**	Oxytocin receptor	OXTR	Receptors
**1B35_HUMAN**	HLA class I histocompatibility antigen, B-35 alpha chain	HLA-B	Miscellaneous
**CTR1_HUMAN**	High affinity cationic amino acid transporter 1	SLC7A1	Transporters
**SSR1_HUMAN**	Somatostatin receptor type 1	SSTR1	Receptors
**SSR2_HUMAN**	Somatostatin receptor type 2	SSTR2	Receptors
**ACHB4_HUMAN**	Neuronal acetylcholine receptor subunit beta-4	CHRNB4	Transporters
**5HT1F_HUMAN**	5-hydroxytryptamine receptor 1F	HTR1F	Receptors

**OR1E1_HUMAN**	Olfactory receptor 1E1	OR1E1	Receptors
**O10J1_HUMAN**	Olfactory receptor 10J1	OR10J1	Receptors
**GNRHR_HUMAN**	Gonadotropin-releasing hormone receptor	GNRHR	Receptors
**CALCR_HUMAN**	Calcitonin receptor	CALCR	Receptors
**NTR1_HUMAN**	Neurotensin receptor type 1	NTSR1	Receptors
**CD52_HUMAN**	CAMPATH-1 antigen	CD52	Unclassified
**SSR4_HUMAN**	Somatostatin receptor type 4	SSTR4	Receptors
**SC5A2_HUMAN**	Sodium/glucose cotransporter 2	SLC5A2	Transporters
**SC6A6_HUMAN**	Sodium- and chloride-dependent taurine transporter	SLC6A6	Transporters
**GBRA5_HUMAN**	Gamma-aminobutyric acid receptor subunit alpha-5	GABRA5	Transporters
**SC6A4_HUMAN**	Sodium-dependent serotonin transporter	SLC6A4	Transporters
**IL2RG_HUMAN**	Cytokine receptor common subunit gamma	IL2RG	Receptors
**FCG2B_HUMAN**	Low affinity immunoglobulin gamma Fc region receptor II-b	FCGR2B	Receptors
**FCG2C_HUMAN**	Low affinity immunoglobulin gamma Fc region receptor II-c	FCGR2C	Receptors
**CEAM8_HUMAN**	Carcinoembryonic antigen-related cell adhesion molecule 8	CEACAM8	Miscellaneous
**L1CAM_HUMAN**	Neural cell adhesion molecule L1	L1CAM	Miscellaneous
**CCKAR_HUMAN**	Cholecystokinin receptor type A	CCKAR	Receptors
**GASR_HUMAN**	Gastrin/cholecystokinin type B receptor	CCKBR	Receptors
**VIPR1_HUMAN**	Vasoactive intestinal polypeptide receptor 1	VIPR1	Receptors
**MC4R_HUMAN**	Melanocortin receptor 4	MC4R	Receptors
**CCR1_HUMAN**	C-C chemokine receptor type 1	CCR1	Receptors
**BRS3_HUMAN**	Bombesin receptor subtype-3	BRS3	Receptors
**CCR7_HUMAN**	C-C chemokine receptor type 7	CCR7	Receptors
**GP183_HUMAN**	G-protein coupled receptor 183	GPR183	Receptors
**ACHA3_HUMAN**	Neuronal acetylcholine receptor subunit alpha-3	CHRNA3	Transporters
**CXCR5_HUMAN**	C-X-C chemokine receptor type 5	CXCR5	Receptors
**NAC1_HUMAN**	Sodium/calcium exchanger 1	SLC8A1	Transporters
**SSR3_HUMAN**	Somatostatin receptor type 3	SSTR3	Receptors
**DSG3_HUMAN**	Desmoglein-3	DSG3	Miscellaneous
**IL3RB_HUMAN**	Cytokine receptor common subunit beta	CSF2RB	Receptors
**ICAM3_HUMAN**	Intercellular adhesion molecule 3	ICAM3	Receptors
**CD70_HUMAN**	CD70 antigen	CD70	Unclassified
**TNFL8_HUMAN**	Tumor necrosis factor ligand superfamily member 8	TNFSF8	Unclassified
**MC5R_HUMAN**	Melanocortin receptor 5	MC5R	Receptors
**CADH5_HUMAN**	Cadherin-5	CDH5	Miscellaneous
**MRP1_HUMAN**	Multidrug resistance-associated protein 1	ABCC1	Transporters
**CD80_HUMAN**	T-lymphocyte activation antigen CD80	CD80	Unclassified
**AA3R_HUMAN**	Adenosine receptor A3	ADORA3	
**SDC2_HUMAN**	Syndecan-2	SDC2	Receptors
**CD68_HUMAN**	Macrosialin	CD68	Receptors

**GBRA3_HUMAN**	Gamma-aminobutyric acid receptor subunit alpha-3	GABRA3	Transporters
**EVI2B_HUMAN**	Protein EVI2B	EVI2B	Unclassified
**RYK_HUMAN**	Tyrosine-protein kinase RYK	RYK	Receptors
**5HT7R_HUMAN**	5-hydroxytryptamine receptor 7	HTR7	Receptors
**CNR2_HUMAN**	Cannabinoid receptor 2	CNR2	Receptors
**TRFR_HUMAN**	Thyrotropin-releasing hormone receptor	TRHR	Receptors
**OR1D2_HUMAN**	Olfactory receptor 1D2	OR1D2	Receptors
**PE2R1_HUMAN**	Prostaglandin E2 receptor EP1 subtype	PTGER1	Receptors
**CRFR1_HUMAN**	Corticotropin-releasing factor receptor 1	CRHR1	Receptors
**GPC1_HUMAN**	Glypican-1	GPC1	
**BTC_HUMAN**	Probetacellulin	BTC	Unclassified
**CXA4_HUMAN**	Gap junction alpha-4 protein	GJA4	Miscellaneous
**SSR5_HUMAN**	Somatostatin receptor type 5	SSTR5	Receptors
**ADA1A_HUMAN**	Alpha-1A adrenergic receptor	ADRA1A	Receptors
**HRH1_HUMAN**	Histamine H1 receptor	HRH1	Receptors
**ADA1B_HUMAN**	Alpha-1B adrenergic receptor	ADRA1B	Receptors
**OPRM_HUMAN**	Mu-type opioid receptor	OPRM1	Receptors
**PE2R4_HUMAN**	Prostaglandin E2 receptor EP4 subtype	PTGER4	Receptors
**MAS1L_HUMAN**	Mas-related G-protein coupled receptor MRG	MAS1L	Receptors
**APJ_HUMAN**	Apelin receptor	APLNR	Receptors
**DRD3_HUMAN**	D(3) dopamine receptor	DRD3	Receptors
**SCN1A_HUMAN**	Sodium channel protein type 1 subunit alpha	SCN1A	Transporters
**SCN4A_HUMAN**	Sodium channel protein type 4 subunit alpha	SCN4A	Transporters
**TIE1_HUMAN**	Tyrosine-protein kinase receptor Tie-1	TIE1	Receptors
**BASI_HUMAN**	Basigin	BSG	Unclassified
**VGFR3_HUMAN**	Vascular endothelial growth factor receptor 3	FLT4	Receptors
**VGFR2_HUMAN**	Vascular endothelial growth factor receptor 2	KDR	Receptors
**ACHA7_HUMAN**	Neuronal acetylcholine receptor subunit alpha-7	CHRNA7	Transporters
**FLT3_HUMAN**	Receptor-type tyrosine-protein kinase FLT3	FLT3	Receptors
**BMR1A_HUMAN**	Bone morphogenetic protein receptor type-1A	BMPR1A	Receptors
**ACV1B_HUMAN**	Activin receptor type-1B	ACVR1B	Receptors
**TGFR1_HUMAN**	TGF-beta receptor type-1	TGFBR1	Receptors
**TNR3_HUMAN**	Tumor necrosis factor receptor superfamily member 3	LTBR	Receptors
**ACVL1_HUMAN**	Serine/threonine-protein kinase receptor R3	ACVRL1	Receptors
**SCNNA_HUMAN**	Amiloride-sensitive sodium channel subunit alpha	SCNN1A	Unclassified
**TGFR2_HUMAN**	TGF-beta receptor type-2	TGFBR2	Receptors
**V1AR_HUMAN**	Vasopressin V1a receptor	AVPR1A	Receptors
**INGR2_HUMAN**	Interferon gamma receptor 2	IFNGR2	Receptors
**HYALP_HUMAN**	Hyaluronidase PH-20	SPAM1	Enzymes
**ITAE_HUMAN**	Integrin alpha-E	ITGAE	Receptors

**GRIK1_HUMAN**	Glutamate receptor ionotropic, kainate 1	GRIK1	Transporters
**TYRP2_HUMAN**	L-dopachrome tautomerase	DCT	Enzymes
**IL6RB_HUMAN**	Interleukin-6 receptor subunit beta	IL6ST	Receptors
**GPV_HUMAN**	Platelet glycoprotein V	GP5	Miscellaneous
**CEAM3_HUMAN**	Carcinoembryonic antigen-related cell adhesion molecule 3	CEACAM3	Miscellaneous
**CEAM6_HUMAN**	Carcinoembryonic antigen-related cell adhesion molecule 6	CEACAM6	Miscellaneous
**TACT_HUMAN**	T-cell surface protein tactile	CD96	Unclassified
**TPOR_HUMAN**	Thrombopoietin receptor	MPL	Receptors
**CD79B_HUMAN**	B-cell antigen receptor complex-associated protein beta chain	CD79B	Unclassified
**S26A3_HUMAN**	Chloride anion exchanger	SLC26A3	Transporters
**PMEL_HUMAN**	Melanocyte protein PMEL	PMEL	Unclassified
**OPRD_HUMAN**	Delta-type opioid receptor	OPRD1	Receptors
**OPRK_HUMAN**	Kappa-type opioid receptor	OPRK1	Receptors
**OPRX_HUMAN**	Nociceptin receptor	OPRL1	Receptors
**CASR_HUMAN**	Extracellular calcium-sensing receptor	CASR	Receptors
**AQP2_HUMAN**	Aquaporin-2	AQP2	Transporters
**OX2G_HUMAN**	OX-2 membrane glycoprotein	CD200	Miscellaneous
**P2RY2_HUMAN**	P2Y purinoceptor 2	P2RY2	Receptors
**S19A1_HUMAN**	Folate transporter 1	SLC19A1	Transporters
**PACR_HUMAN**	Pituitary adenylate cyclase-activating polypeptide type I receptor	ADCYAP1R1	Receptors
**VIPR2_HUMAN**	Vasoactive intestinal polypeptide receptor 2	VIPR2	Receptors
**GRM5_HUMAN**	Metabotropic glutamate receptor 5	GRM5	Receptors
**5HT2B_HUMAN**	5-hydroxytryptamine receptor 2B	HTR2B	Receptors
**CCR2_HUMAN**	C-C chemokine receptor type 2	CCR2	Unclassified
**TSN7_HUMAN**	Tetraspanin-7	TSPAN7	Miscellaneous
**MC3R_HUMAN**	Melanocortin receptor 3	MC3R	Receptors
**CD86_HUMAN**	T-lymphocyte activation antigen CD86	CD86	Miscellaneous
**GRIA1_HUMAN**	Glutamate receptor 1	GRIA1	Transporters
**GRIA2_HUMAN**	Glutamate receptor 2	GRIA2	Transporters
**GRIA3_HUMAN**	Glutamate receptor 3	GRIA3	Transporters
**DPP6_HUMAN**	Dipeptidyl aminopeptidase-like protein 6	DPP6	Unclassified
**I12R1_HUMAN**	Interleukin-12 receptor subunit beta-1	IL12RB1	Receptors
**LIFR_HUMAN**	Leukemia inhibitory factor receptor	LIFR	Receptors
**ECE1_HUMAN**	Endothelin-converting enzyme 1	ECE1	Enzymes
**EAA1_HUMAN**	Excitatory amino acid transporter 1	SLC1A3	Transporters
**EAA2_HUMAN**	Excitatory amino acid transporter 2	SLC1A2	Transporters
**EAA3_HUMAN**	Excitatory amino acid transporter 3	SLC1A1	Transporters
**SATT_HUMAN**	Neutral amino acid transporter A	SLC1A4	Transporters
**PF2R_HUMAN**	Prostaglandin F2-alpha receptor	PTGFR	Receptors
**PE2R3_HUMAN**	Prostaglandin E2 receptor EP3 subtype	PTGER3	Receptors

**PE2R2_HUMAN**	Prostaglandin E2 receptor EP2 subtype	PTGER2	Receptors
**PI2R_HUMAN**	Prostacyclin receptor	PTGIR	Receptors
**MUC18_HUMAN**	Cell surface glycoprotein MUC18	MCAM	Unclassified
**DCC_HUMAN**	Netrin receptor DCC	DCC	Receptors
**GLP1R_HUMAN**	Glucagon-like peptide 1 receptor	GLP1R	Receptors
**SSRA_HUMAN**	Translocon-associated protein subunit alpha	SSR1	Unclassified
**TNR4_HUMAN**	Tumor necrosis factor receptor superfamily member 4	TNFRSF4	Receptors
**KI2L1_HUMAN**	Killer cell immunoglobulin-like receptor 2DL1	KIR2DL1	Receptors
**KI2L2_HUMAN**	Killer cell immunoglobulin-like receptor 2DL2	KIR2DL2	Receptors
**KI2L3_HUMAN**	Killer cell immunoglobulin-like receptor 2DL3	KIR2DL3	Receptors
**KI3L1_HUMAN**	Killer cell immunoglobulin-like receptor 3DL1	KIR3DL1	Receptors
**KI3L2_HUMAN**	Killer cell immunoglobulin-like receptor 3DL2	KIR3DL2	Receptors
**KI2S2_HUMAN**	Killer cell immunoglobulin-like receptor 2DS2	KIR2DS2	Receptors
**KI2S4_HUMAN**	Killer cell immunoglobulin-like receptor 2DS4	KIR2DS4	Receptors
**LPAR6_HUMAN**	Lysophosphatidic acid receptor 6	LPAR6	Receptors
**ACHA4_HUMAN**	Neuronal acetylcholine receptor subunit alpha-4	CHRNA4	Transporters
**S15A1_HUMAN**	Solute carrier family 15 member 1	SLC15A1	Transporters
**GPR3_HUMAN**	G-protein coupled receptor 3	GPR3	Receptors
**GPR1_HUMAN**	G-protein coupled receptor 1	GPR1	Receptors
**CCR10_HUMAN**	C-C chemokine receptor type 10	CCR10	Receptors
**GPR4_HUMAN**	G-protein coupled receptor 4	GPR4	Receptors
**XCR1_HUMAN**	Chemokine XC receptor 1	XCR1	Receptors
**GPR6_HUMAN**	G-protein coupled receptor 6	GPR6	Receptors
**5HT3A_HUMAN**	5-hydroxytryptamine receptor 3A	HTR3A	Transporters
**NOTC1_HUMAN**	Neurogenic locus notch homolog protein 1	NOTCH1	Receptors
**BKRB1_HUMAN**	B1 bradykinin receptor	BDKRB1	Receptors
**SO1A2_HUMAN**	Solute carrier organic anion transporter family member 1A2	SLCO1A2	Transporters
**GALR1_HUMAN**	Galanin receptor type 1	GALR1	Receptors
**GPR12_HUMAN**	G-protein coupled receptor 12	GPR12	Receptors
**RGR_HUMAN**	RPE-retinal G protein-coupled receptor	RGR	Receptors
**GBRA2_HUMAN**	Gamma-aminobutyric acid receptor subunit alpha-2	GABRA2	Transporters
**GBRB2_HUMAN**	Gamma-aminobutyric acid receptor subunit beta-2	GABRB2	Transporters
**GLR_HUMAN**	Glucagon receptor	GCGR	Unclassified
**SCTR_HUMAN**	Secretin receptor	SCTR	Receptors
**OR3A1_HUMAN**	Olfactory receptor 3A1	OR3A1	Receptors
**OR3A4_HUMAN**	Putative olfactory receptor 3A4	OR3A4P	Receptors
**OR1D4_HUMAN**	Olfactory receptor 1D4	OR1D4	Receptors
**OR1E2_HUMAN**	Olfactory receptor 1E2	OR1E2	Receptors
**OR3A3_HUMAN**	Olfactory receptor 3A3	OR3A3	Receptors
**OR1G1_HUMAN**	Olfactory receptor 1G1	OR1G1	Receptors

**OR3A2_HUMAN**	Olfactory receptor 3A2	OR3A2	Receptors
**5HT5A_HUMAN**	5-hydroxytryptamine receptor 5A	HTR5A	Receptors
**P2RY1_HUMAN**	P2Y purinoceptor 1	P2RY1	Receptors
**V1BR_HUMAN**	Vasopressin V1b receptor	AVPR1B	Receptors
**TNFL6_HUMAN**	Tumor necrosis factor ligand superfamily member 6	FASLG	Unclassified
**SC6A8_HUMAN**	Sodium- and chloride-dependent creatine transporter 1	SLC6A8	Transporters
**MTR1A_HUMAN**	Melatonin receptor type 1A	MTNR1A	Receptors
**KCNJ4_HUMAN**	Inward rectifier potassium channel 4	KCNJ4	Transporters
**GRIA4_HUMAN**	Glutamate receptor 4	GRIA4	Transporters
**GLIP1_HUMAN**	Glioma pathogenesis-related protein 1	GLIPR1	Unclassified
**S6A12_HUMAN**	Sodium- and chloride-dependent betaine transporter	SLC6A12	Transporters
**S6A11_HUMAN**	Sodium- and chloride-dependent GABA transporter 3	SLC6A11	Transporters
**SC6A9_HUMAN**	Sodium- and chloride-dependent glycine transporter 1	SLC6A9	Transporters
**NPBW1_HUMAN**	Neuropeptides B/W receptor type 1	NPBWR1	Receptors
**NPBW2_HUMAN**	Neuropeptides B/W receptor type 2	NPBWR2	Receptors
**GLRB_HUMAN**	Glycine receptor subunit beta	GLRB	Transporters
**GBRA4_HUMAN**	Gamma-aminobutyric acid receptor subunit alpha-4	GABRA4	Transporters
**T4S4_HUMAN**	Transmembrane 4 L6 family member 4	TM4SF4	Miscellaneous
**LEPR_HUMAN**	Leptin receptor	LEPR	Receptors
**CD151_HUMAN**	CD151 antigen	CD151	Miscellaneous
**GIPR_HUMAN**	Gastric inhibitory polypeptide receptor	GIPR	Receptors
**KCNJ3_HUMAN**	G protein-activated inward rectifier potassium channel 1	KCNJ3	Transporters
**INAR2_HUMAN**	Interferon alpha/beta receptor 2	IFNAR2	Receptors
**EAA4_HUMAN**	Excitatory amino acid transporter 4	SLC1A6	Transporters
**SL9A3_HUMAN**	Sodium/hydrogen exchanger 3	SLC9A3	Transporters
**CD97_HUMAN**	CD97 antigen	CD97	Receptors
**HCAR3_HUMAN**	Hydroxycarboxylic acid receptor 3	HCAR3	Receptors
**NPY2R_HUMAN**	Neuropeptide Y receptor type 2	NPY2R	Receptors
**PTH2R_HUMAN**	Parathyroid hormone 2 receptor	PTH2R	Receptors
**CX3C1_HUMAN**	CX3C chemokine receptor 1	CX3CR1	Receptors
**NRAM1_HUMAN**	Natural resistance-associated macrophage protein 1	SLC11A1	Transporters
**NRAM2_HUMAN**	Natural resistance-associated macrophage protein 2	SLC11A2	Transporters
**MTR1B_HUMAN**	Melatonin receptor type 1B	MTNR1B	Receptors
**CXCR3_HUMAN**	C-X-C chemokine receptor type 3	CXCR3	Receptors
**PRLHR_HUMAN**	Prolactin-releasing peptide receptor	PRLHR	Receptors
**GPR15_HUMAN**	G-protein coupled receptor 15	GPR15	Receptors
**PSN1_HUMAN**	Presenilin-1	PSEN1	Unclassified
**FLT3L_HUMAN**	Fms-related tyrosine kinase 3 ligand	FLT3LG	Unclassified
**PSN2_HUMAN**	Presenilin-2	PSEN2	Unclassified
**ENTP1_HUMAN**	Ectonucleoside triphosphate diphosphohydrolase 1	ENTPD1	Enzymes

**AGTR2_HUMAN**	Type-2 angiotensin II receptor	AGTR2	Receptors
**MMP14_HUMAN**	Matrix metalloproteinase-14	MMP14	Enzymes
**NPY4R_HUMAN**	Neuropeptide Y receptor type 4	NPY4R	Receptors
**5HT6R_HUMAN**	5-hydroxytryptamine receptor 6	HTR6	Receptors
**S26A2_HUMAN**	Sulfate transporter	SLC26A2	Transporters
**BCAM_HUMAN**	Basal cell adhesion molecule	BCAM	Miscellaneous
**AT1A2_HUMAN**	Sodium/potassium-transporting ATPase subunit alpha-2	ATP1A2	Transporters
**ATP4B_HUMAN**	Potassium-transporting ATPase subunit beta	ATP4B	Transporters
**SCNNB_HUMAN**	Amiloride-sensitive sodium channel subunit beta	SCNN1B	Unclassified
**SCNNG_HUMAN**	Amiloride-sensitive sodium channel subunit gamma	SCNN1G	Unclassified
**SCNND_HUMAN**	Amiloride-sensitive sodium channel subunit delta	SCNN1D	Unclassified
**MMP16_HUMAN**	Matrix metalloproteinase-16	MMP16	Enzymes
**P2RX1_HUMAN**	P2X purinoceptor 1	P2RX1	Transporters
**P2RY4_HUMAN**	P2Y purinoceptor 4	P2RY4	Receptors
**GPC3_HUMAN**	Glypican-3	GPC3	
**GPM6A_HUMAN**	Neuronal membrane glycoprotein M6-a	GPM6A	Unclassified
**CCR3_HUMAN**	C-C chemokine receptor type 3	CCR3	Receptors
**CCR4_HUMAN**	C-C chemokine receptor type 4	CCR4	Receptors
**CCR5_HUMAN**	C-C chemokine receptor type 5	CCR5	Unclassified
**CCR6_HUMAN**	C-C chemokine receptor type 6	CCR6	Receptors
**CCR8_HUMAN**	C-C chemokine receptor type 8	CCR8	Receptors
**CCR9_HUMAN**	C-C chemokine receptor type 9	CCR9	Receptors
**APLP1_HUMAN**	Amyloid-like protein 1	APLP1	Unclassified
**CLCKB_HUMAN**	Chloride channel protein ClC-Kb	CLCNKB	Transporters
**PLXA3_HUMAN**	Plexin-A3	PLXNA3	Receptors
**GP143_HUMAN**	G-protein coupled receptor 143	GPR143	Receptors
**ADCY7_HUMAN**	Adenylate cyclase type 7	ADCY7	Enzymes
**CTR2_HUMAN**	Cationic amino acid transporter 2	SLC7A2	Transporters
**EFNA3_HUMAN**	Ephrin-A3	EFNA3	Unclassified
**EFNA4_HUMAN**	Ephrin-A4	EFNA4	Unclassified
**EFNB2_HUMAN**	Ephrin-B2	EFNB2	Miscellaneous
**EFNA5_HUMAN**	Ephrin-A5	EFNA5	Unclassified
**NAR1_HUMAN**	GPI-linked NAD(P)(+)--arginine ADP-ribosyltransferase 1	ART1	Enzymes
**ITA8_HUMAN**	Integrin alpha-8	ITGA8	Receptors
**SC5A3_HUMAN**	Sodium/myo-inositol cotransporter	SLC5A3	Transporters
**PTTG_HUMAN**	Pituitary tumor-transforming gene 1 protein-interacting protein	PTTG1IP	Unclassified
**MOT1_HUMAN**	Monocarboxylate transporter 1	SLC16A1	Transporters
**VMAT1_HUMAN**	Chromaffin granule amine transporter	SLC18A1	Transporters
**AT1B3_HUMAN**	Sodium/potassium-transporting ATPase subunit beta-3	ATP1B3	Transporters
**EPHB3_HUMAN**	Ephrin type-B receptor 3	EPHB3	Receptors

**EPHA5_HUMAN**	Ephrin type-A receptor 5	EPHA5	Receptors
**EPHB4_HUMAN**	Ephrin type-B receptor 4	EPHB4	Receptors
**EPHB1_HUMAN**	Ephrin type-B receptor 1	EPHB1	Receptors
**EPHA4_HUMAN**	Ephrin type-A receptor 4	EPHA4	Receptors
**GAS1_HUMAN**	Growth arrest-specific protein 1	GAS1	
**EMP1_HUMAN**	Epithelial membrane protein 1	EMP1	Miscellaneous
**EMP2_HUMAN**	Epithelial membrane protein 2	EMP2	Miscellaneous
**EMP3_HUMAN**	Epithelial membrane protein 3	EMP3	Miscellaneous
**S12A2_HUMAN**	Solute carrier family 12 member 2	SLC12A2	Transporters
**S12A3_HUMAN**	Solute carrier family 12 member 3	SLC12A3	Transporters
**AQP5_HUMAN**	Aquaporin-5	AQP5	Transporters
**MFAP3_HUMAN**	Microfibril-associated glycoprotein 3	MFAP3	Unclassified
**PAR2_HUMAN**	Proteinase-activated receptor 2	F2RL1	Receptors
**AQP4_HUMAN**	Aquaporin-4	AQP4	Transporters
**GP2_HUMAN**	Pancreatic secretory granule membrane major glycoprotein GP2	GP2	Unclassified
**CADH4_HUMAN**	Cadherin-4	CDH4	Miscellaneous
**CADH6_HUMAN**	Cadherin-6	CDH6	Miscellaneous
**CADH8_HUMAN**	Cadherin-8	CDH8	Miscellaneous
**CAD11_HUMAN**	Cadherin-11	CDH11	Miscellaneous
**CAD12_HUMAN**	Cadherin-12	CDH12	Miscellaneous
**CAD13_HUMAN**	Cadherin-13	CDH13	Miscellaneous
**CAD15_HUMAN**	Cadherin-15	CDH15	Miscellaneous
**LMIP_HUMAN**	Lens fiber membrane intrinsic protein	LIM2	Miscellaneous
**FCGRN_HUMAN**	IgG receptor FcRn large subunit p51	FCGRT	Receptors
**GFRA1_HUMAN**	GDNF family receptor alpha-1	GFRA1	
**ITA1_HUMAN**	Integrin alpha-1	ITGA1	Receptors
**P2RX3_HUMAN**	P2X purinoceptor 3	P2RX3	Transporters
**CLD15_HUMAN**	Claudin-15	CLDN15	Miscellaneous
**CLD6_HUMAN**	Claudin-6	CLDN6	Miscellaneous
**CLD8_HUMAN**	Claudin-8	CLDN8	Miscellaneous
**CLD12_HUMAN**	Claudin-12	CLDN12	Miscellaneous
**BACE1_HUMAN**	Beta-secretase 1	BACE1	Enzymes
**CLD18_HUMAN**	Claudin-18	CLDN18	Miscellaneous
**CLD20_HUMAN**	Claudin-20	CLDN20	Miscellaneous
**GLPT_HUMAN**	Glycerol-3-phosphate transporter	SLC37A1	Transporters
**JAM2_HUMAN**	Junctional adhesion molecule B	JAM2	Miscellaneous
**NAC3_HUMAN**	Sodium/calcium exchanger 3	SLC8A3	Transporters
**CLD2_HUMAN**	Claudin-2	CLDN2	Miscellaneous
**OR1D5_HUMAN**	Olfactory receptor 1D5	OR1D5	Receptors
**OR2B6_HUMAN**	Olfactory receptor 2B6	OR2B6	Receptors

**OR4D2_HUMAN**	Olfactory receptor 4D2	OR4D2	Receptors
**O10A3_HUMAN**	Olfactory receptor 10A3	OR10A3	Receptors
**O12D2_HUMAN**	Olfactory receptor 12D2	OR12D2	Receptors
**ANTR2_HUMAN**	Anthrax toxin receptor 2	ANTXR2	Unclassified
**NRX1B_HUMAN**	Neurexin-1-beta	NRXN1	Receptors
**NRX2B_HUMAN**	Neurexin-2-beta	NRXN2	Receptors
**CLRN1_HUMAN**	Clarin-1	CLRN1	Unclassified
**EVA1C_HUMAN**	Protein eva-1 homolog C	EVA1C	Unclassified
**S26A5_HUMAN**	Prestin	SLC26A5	Transporters
**T2R38_HUMAN**	Taste receptor type 2 member 38	TAS2R38	Receptors
**T2R39_HUMAN**	Taste receptor type 2 member 39	TAS2R39	Receptors
**T2R46_HUMAN**	Taste receptor type 2 member 46	TAS2R46	Receptors
**T2R30_HUMAN**	Taste receptor type 2 member 30	TAS2R30	Receptors
**T2R19_HUMAN**	Taste receptor type 2 member 19	TAS2R19	Receptors
**T2R20_HUMAN**	Taste receptor type 2 member 20	TAS2R20	Receptors
**LIRA4_HUMAN**	Leukocyte immunoglobulin-like receptor subfamily A member 4	LILRA4	Receptors
**OR2B8_HUMAN**	Putative olfactory receptor 2B8	OR2B8P	Receptors
**MYPR_HUMAN**	Myelin proteolipid protein	PLP1	Unclassified
**EFC1_HUMAN**	Endogenous retrovirus group FC1 Env polyprotein	ERVFC1	Miscellaneous
**SYCY2_HUMAN**	Syncytin-2	ERVFRD-1	Miscellaneous
**ERB1_HUMAN**	Endogenous retrovirus group PABLB member 1 Env polyprotein	ERVPABLB-1	Miscellaneous
**ZP1_HUMAN**	Zona pellucida sperm-binding protein 1	ZP1	Unclassified
**GPR85_HUMAN**	Probable G-protein coupled receptor 85	GPR85	Receptors
**CXCR4_HUMAN**	C-X-C chemokine receptor type 4	CXCR4	Receptors
**ENVT1_HUMAN**	Endogenous retrovirus group S71 member 1 Env polyprotein	ERVS71-1	Miscellaneous
**ENK21_HUMAN**	Endogenous retrovirus group K member 21 Env polyprotein	ERVK-21	Miscellaneous
**ENK24_HUMAN**	Endogenous retrovirus group K member 24 Env polyprotein	ERVK-24	Miscellaneous
**ENK7_HUMAN**	Endogenous retrovirus group K member 7 Env polyprotein	ERVK-7	Enzymes
**ENK25_HUMAN**	Endogenous retrovirus group K member 25 Env polyprotein	ERVK-25	Miscellaneous
**TSN5_HUMAN**	Tetraspanin-5	TSPAN5	Miscellaneous
**CCG7_HUMAN**	Voltage-dependent calcium channel gamma-7 subunit	CACNG7	Transporters
**CXAR_HUMAN**	Coxsackievirus and adenovirus receptor	CXADR	Receptors
**SHPS1_HUMAN**	Tyrosine-protein phosphatase non-receptor type substrate 1	SIRPA	Miscellaneous
**ADAM8_HUMAN**	Disintegrin and metalloproteinase domain-containing protein 8	ADAM8	Enzymes
**GPC5_HUMAN**	Glypican-5	GPC5	
**GBRE_HUMAN**	Gamma-aminobutyric acid receptor subunit epsilon	GABRE	Transporters
**ASIC1_HUMAN**	Acid-sensing ion channel 1	ASIC1	Unclassified
**CNTP1_HUMAN**	Contactin-associated protein 1	CNTNAP1	Receptors
**ABCA4_HUMAN**	Retinal-specific ATP-binding cassette transporter	ABCA4	Transporters
**CLD10_HUMAN**	Claudin-10	CLDN10	Miscellaneous

**OLR1_HUMAN**	Oxidized low-density lipoprotein receptor 1	OLR1	Receptors
**BT3A2_HUMAN**	Butyrophilin subfamily 3 member A2	BTN3A2	Miscellaneous
**X3CL1_HUMAN**	Fractalkine	CX3CL1	Unclassified
**JAG1_HUMAN**	Protein jagged-1	JAG1	Miscellaneous
**ADA17_HUMAN**	Disintegrin and metalloproteinase domain-containing protein 17	ADAM17	Enzymes
**I13R1_HUMAN**	Interleukin-13 receptor subunit alpha-1	IL13RA1	Receptors
**PHEX_HUMAN**	Phosphate-regulating neutral endopeptidase	PHEX	Enzymes
**DRB3_HUMAN**	HLA class II histocompatibility antigen, DR beta 3 chain	HLA-DRB3	Miscellaneous
**DLK1_HUMAN**	Protein delta homolog 1	DLK1	Miscellaneous
**F189B_HUMAN**	Protein FAM189B	FAM189B	Unclassified
**BAT1_HUMAN**	b(0,+)-type amino acid transporter 1	SLC7A9	Transporters
**CRUM1_HUMAN**	Protein crumbs homolog 1	CRB1	Miscellaneous
**ENTK_HUMAN**	Enteropeptidase	TMPRSS15	Enzymes
**IDD_HUMAN**	Integral membrane protein DGCR2/IDD	DGCR2	Receptors
**VLDLR_HUMAN**	Very low-density lipoprotein receptor	VLDLR	Receptors
**PKD1_HUMAN**	Polycystin-1	PKD1	Transporters
**LRP2_HUMAN**	Low-density lipoprotein receptor-related protein 2	LRP2	Receptors
**EFNB1_HUMAN**	Ephrin-B1	EFNB1	Miscellaneous
**IL9R_HUMAN**	Interleukin-9 receptor	IL9R	Receptors
**SCN7A_HUMAN**	Sodium channel protein type 7 subunit alpha	SCN7A	Transporters
**CD83_HUMAN**	CD83 antigen	CD83	Unclassified
**IL5RA_HUMAN**	Interleukin-5 receptor subunit alpha	IL5RA	Receptors
**ILRL1_HUMAN**	Interleukin-1 receptor-like 1	IL1RL1	Receptors
**LAT1_HUMAN**	Large neutral amino acids transporter small subunit 1	SLC7A5	Transporters
**ACTHR_HUMAN**	Adrenocorticotropic hormone receptor	MC2R	Receptors
**MSHR_HUMAN**	Melanocyte-stimulating hormone receptor	MC1R	Receptors
**AT2B2_HUMAN**	Plasma membrane calcium-transporting ATPase 2	ATP2B2	Transporters
**SC6A3_HUMAN**	Sodium-dependent dopamine transporter	SLC6A3	Transporters
**ROR1_HUMAN**	Tyrosine-protein kinase transmembrane receptor ROR1	ROR1	Receptors
**ROR2_HUMAN**	Tyrosine-protein kinase transmembrane receptor ROR2	ROR2	Receptors
**RHAG_HUMAN**	Ammonium transporter Rh type A	RHAG	Transporters
**TNR17_HUMAN**	Tumor necrosis factor receptor superfamily member 17	TNFRSF17	Receptors
**CNTN2_HUMAN**	Contactin-2	CNTN2	Unclassified
**NRG1_HUMAN**	Pro-neuregulin-1, membrane-bound isoform	NRG1	Unclassified
**DSG1_HUMAN**	Desmoglein-1	DSG1	Miscellaneous
**DSC2_HUMAN**	Desmocollin-2	DSC2	Miscellaneous
**MUC3A_HUMAN**	Mucin-3A	MUC3A	Unclassified
**GHRHR_HUMAN**	Growth hormone-releasing hormone receptor	GHRHR	Receptors
**TIE2_HUMAN**	Angiopoietin-1 receptor	TEK	Receptors
**TGBR3_HUMAN**	Transforming growth factor beta receptor type 3	TGFBR3	Unclassified

**UPAR_HUMAN**	Urokinase plasminogen activator surface receptor	PLAUR	
**PTH1R_HUMAN**	Parathyroid hormone/parathyroid hormone-related peptide receptor	PTH1R	Receptors
**FOLH1_HUMAN**	Glutamate carboxypeptidase 2	FOLH1	Enzymes
**NOTC2_HUMAN**	Neurogenic locus notch homolog protein 2	NOTCH2	Receptors
**ACVR1_HUMAN**	Activin receptor type-1	ACVR1	Receptors
**1B40_HUMAN**	HLA class I histocompatibility antigen, B-40 alpha chain	HLA-B	Miscellaneous
**ACHE_HUMAN**	Acetylcholine receptor subunit epsilon	CHRNE	Transporters
**MUC24_HUMAN**	Sialomucin core protein 24	CD164	Unclassified
**RON_HUMAN**	Macrophage-stimulating protein receptor	MST1R	Receptors
**NMDZ1_HUMAN**	Glutamate receptor ionotropic, NMDA 1	GRIN1	Transporters
**ACHB3_HUMAN**	Neuronal acetylcholine receptor subunit beta-3	CHRNB3	Transporters
**VMAT2_HUMAN**	Synaptic vesicular amine transporter	SLC18A2	Transporters
**ZP2_HUMAN**	Zona pellucida sperm-binding protein 2	ZP2	Unclassified
**TYRO3_HUMAN**	Tyrosine-protein kinase receptor TYRO3	TYRO3	Receptors
**CCG1_HUMAN**	Voltage-dependent calcium channel gamma-1 subunit	CACNG1	Transporters
**APLP2_HUMAN**	Amyloid-like protein 2	APLP2	Unclassified
**NPT2A_HUMAN**	Sodium-dependent phosphate transport protein 2A	SLC34A1	Transporters
**1C15_HUMAN**	HLA class I histocompatibility antigen, Cw-15 alpha chain	HLA-C	Miscellaneous
**ACHD_HUMAN**	Acetylcholine receptor subunit delta	CHRND	Transporters
**TNR9_HUMAN**	Tumor necrosis factor receptor superfamily member 9	TNFRSF9	Receptors
**AMPE_HUMAN**	Glutamyl aminopeptidase	ENPEP	Enzymes
**CD69_HUMAN**	Early activation antigen CD69	CD69	Receptors
**NKG2E_HUMAN**	NKG2-E type II integral membrane protein	KLRC3	Receptors
**SCN1B_HUMAN**	Sodium channel subunit beta-1	SCN1B	Transporters
**SLC31_HUMAN**	Neutral and basic amino acid transport protein rBAT	SLC3A1	Transporters
**LRP1_HUMAN**	Prolow-density lipoprotein receptor-related protein 1	LRP1	Receptors
**PCDH1_HUMAN**	Protocadherin-1	PCDH1	Miscellaneous
**I10R2_HUMAN**	Interleukin-10 receptor subunit beta	IL10RB	Receptors
**DDR1_HUMAN**	Epithelial discoidin domain-containing receptor 1	DDR1	Receptors
**S20A2_HUMAN**	Sodium-dependent phosphate transporter 2	SLC20A2	Transporters
**ADCY2_HUMAN**	Adenylate cyclase type 2	ADCY2	Enzymes
**DSC1_HUMAN**	Desmocollin-1	DSC1	Miscellaneous
**CLM6_HUMAN**	CMRF35-like molecule 6	CD300C	Receptors
**CD47_HUMAN**	Leukocyte surface antigen CD47	CD47	Unclassified
**S38AB_HUMAN**	Putative sodium-coupled neutral amino acid transporter 11	SLC38A11	Transporters
**SIG14_HUMAN**	Sialic acid-binding Ig-like lectin 14	SIGLEC14	Miscellaneous
**1A80_HUMAN**	HLA class I histocompatibility antigen, A-80 alpha chain	HLA-A	Miscellaneous
**NIPA4_HUMAN**	Magnesium transporter NIPA4	NIPAL4	Transporters
**TMM62_HUMAN**	Transmembrane protein 62	TMEM62	Unclassified
**BST1_HUMAN**	ADP-ribosyl cyclase/cyclic ADP-ribose hydrolase 2	BST1	

**BST2_HUMAN**	Bone marrow stromal antigen 2	BST2	Unclassified
**SCAP_HUMAN**	Sterol regulatory element-binding protein cleavage-activating protein	SCAP	Unclassified
**ZP4_HUMAN**	Zona pellucida sperm-binding protein 4	ZP4	Unclassified
**CNTN1_HUMAN**	Contactin-1	CNTN1	Unclassified
**CAD17_HUMAN**	Cadherin-17	CDH17	Miscellaneous
**MERTK_HUMAN**	Tyrosine-protein kinase Mer	MERTK	Receptors
**NMDE1_HUMAN**	Glutamate receptor ionotropic, NMDA 2A	GRIN2A	Transporters
**SEPR_HUMAN**	Prolyl endopeptidase FAP	FAP	
**HYAL2_HUMAN**	Hyaluronidase-2	HYAL2	
**LMAN2_HUMAN**	Vesicular integral-membrane protein VIP36	LMAN2	Unclassified
**NTCP2_HUMAN**	Ileal sodium/bile acid cotransporter	SLC10A2	Transporters
**PTPRJ_HUMAN**	Receptor-type tyrosine-protein phosphatase eta	PTPRJ	Receptors
**KLRB1_HUMAN**	Killer cell lectin-like receptor subfamily B member 1	KLRB1	Receptors
**TSN31_HUMAN**	Tetraspanin-31	TSPAN31	Miscellaneous
**GRIK2_HUMAN**	Glutamate receptor ionotropic, kainate 2	GRIK2	Transporters
**GRIK3_HUMAN**	Glutamate receptor ionotropic, kainate 3	GRIK3	Transporters
**PLA2R_HUMAN**	Secretory phospholipase A2 receptor	PLA2R1	Receptors
**BAMBI_HUMAN**	BMP and activin membrane-bound inhibitor homolog	BAMBI	Unclassified
**S13A2_HUMAN**	Solute carrier family 13 member 2	SLC13A2	Transporters
**NMDE2_HUMAN**	Glutamate receptor ionotropic, NMDA 2B	GRIN2B	Transporters
**GRM1_HUMAN**	Metabotropic glutamate receptor 1	GRM1	Receptors
**PD2R_HUMAN**	Prostaglandin D2 receptor	PTGDR	Receptors
**I15RA_HUMAN**	Interleukin-15 receptor subunit alpha	IL15RA	Receptors
**CLN3_HUMAN**	Battenin	CLN3	Unclassified
**SLAF1_HUMAN**	Signaling lymphocytic activation molecule	SLAMF1	Unclassified
**GPR17_HUMAN**	Uracil nucleotide/cysteinyl leukotriene receptor	GPR17	Receptors
**PTK7_HUMAN**	Inactive tyrosine-protein kinase 7	PTK7	Receptors
**CRFR2_HUMAN**	Corticotropin-releasing factor receptor 2	CRHR2	Receptors
**PTPRS_HUMAN**	Receptor-type tyrosine-protein phosphatase S	PTPRS	Receptors
**UT1_HUMAN**	Urea transporter 1	SLC14A1	Transporters
**ITAD_HUMAN**	Integrin alpha-D	ITGAD	Receptors
**BT1A1_HUMAN**	Butyrophilin subfamily 1 member A1	BTN1A1	Miscellaneous
**MSLN_HUMAN**	Mesothelin	MSLN	
**S39A6_HUMAN**	Zinc transporter ZIP6	SLC39A6	Transporters
**ADAM9_HUMAN**	Disintegrin and metalloproteinase domain-containing protein 9	ADAM9	Enzymes
**ADA15_HUMAN**	Disintegrin and metalloproteinase domain-containing protein 15	ADAM15	Enzymes
**LSAMP_HUMAN**	Limbic system-associated membrane protein	LSAMP	Unclassified
**FZD5_HUMAN**	Frizzled-5	FZD5	Receptors
**IL18R_HUMAN**	Interleukin-18 receptor 1	IL18R1	Receptors
**VPP3_HUMAN**	V-type proton ATPase 116 kDa subunit a isoform 3	TCIRG1	Unclassified

**GPM6B_HUMAN**	Neuronal membrane glycoprotein M6-b	GPM6B	Unclassified
**NAR3_HUMAN**	Ecto-ADP-ribosyltransferase 3	ART3	Enzymes
**SERC3_HUMAN**	Serine incorporator 3	SERINC3	Transporters
**PKD2_HUMAN**	Polycystin-2	PKD2	Transporters
**MTR1L_HUMAN**	Melatonin-related receptor	GPR50	Receptors
**STIM1_HUMAN**	Stromal interaction molecule 1	STIM1	Unclassified
**SEM5A_HUMAN**	Semaphorin-5A	SEMA5A	Miscellaneous
**OR5I1_HUMAN**	Olfactory receptor 5I1	OR5I1	Receptors
**OR2F1_HUMAN**	Olfactory receptor 2F1	OR2F1	Receptors
**S12A1_HUMAN**	Solute carrier family 12 member 1	SLC12A1	Transporters
**CAD18_HUMAN**	Cadherin-18	CDH18	Miscellaneous
**PTC1_HUMAN**	Protein patched homolog 1	PTCH1	Receptors
**5HT4R_HUMAN**	5-hydroxytryptamine receptor 4	HTR4	Receptors
**TPBG_HUMAN**	Trophoblast glycoprotein	TPBG	Miscellaneous
**I10R1_HUMAN**	Interleukin-10 receptor subunit alpha	IL10RA	Receptors
**ITA7_HUMAN**	Integrin alpha-7	ITGA7	Receptors
**AVR2B_HUMAN**	Activin receptor type-2B	ACVR2B	Receptors
**AT1A4_HUMAN**	Sodium/potassium-transporting ATPase subunit alpha-4	ATP1A4	Transporters
**CD166_HUMAN**	CD166 antigen	ALCAM	Unclassified
**ITA9_HUMAN**	Integrin alpha-9	ITGA9	Receptors
**BMPR2_HUMAN**	Bone morphogenetic protein receptor type-2	BMPR2	Receptors
**CAC1C_HUMAN**	Voltage-dependent L-type calcium channel subunit alpha-1C	CACNA1C	Transporters
**CEAM7_HUMAN**	Carcinoembryonic antigen-related cell adhesion molecule 7	CEACAM7	Miscellaneous
**SCRB2_HUMAN**	Lysosome membrane protein 2	SCARB2	Receptors
**LRP8_HUMAN**	Low-density lipoprotein receptor-related protein 8	LRP8	Receptors
**DAG1_HUMAN**	Dystroglycan	DAG1	Unclassified
**DSG2_HUMAN**	Desmoglein-2	DSG2	Miscellaneous
**SREC_HUMAN**	Scavenger receptor class F member 1	SCARF1	Receptors
**LY6D_HUMAN**	Lymphocyte antigen 6D	LY6D	Unclassified
**SELPL_HUMAN**	P-selectin glycoprotein ligand 1	SELPLG	Unclassified
**EMR1_HUMAN**	EGF-like module-containing mucin-like hormone receptor-like 1	EMR1	Receptors
**GPR18_HUMAN**	N-arachidonyl glycine receptor	GPR18	Receptors
**FZD2_HUMAN**	Frizzled-2	FZD2	Receptors
**LRC32_HUMAN**	Leucine-rich repeat-containing protein 32	LRRC32	Miscellaneous
**GRM2_HUMAN**	Metabotropic glutamate receptor 2	GRM2	Receptors
**GP176_HUMAN**	Probable G-protein coupled receptor 176	GPR176	Receptors
**KCJ12_HUMAN**	ATP-sensitive inward rectifier potassium channel 12	KCNJ12	Transporters
**FAT1_HUMAN**	Protocadherin Fat 1	FAT1	Miscellaneous
**SCN5A_HUMAN**	Sodium channel protein type 5 subunit alpha	SCN5A	Transporters
**S29A2_HUMAN**	Equilibrative nucleoside transporter 2	SLC29A2	Transporters

**DSC3_HUMAN**	Desmocollin-3	DSC3	Miscellaneous
**I11RA_HUMAN**	Interleukin-11 receptor subunit alpha	IL11RA	Receptors
**I13R2_HUMAN**	Interleukin-13 receptor subunit alpha-2	IL13RA2	Receptors
**SSPN_HUMAN**	Sarcospan	SSPN	Unclassified
**ICAM4_HUMAN**	Intercellular adhesion molecule 4	ICAM4	Unclassified
**GRM7_HUMAN**	Metabotropic glutamate receptor 7	GRM7	Receptors
**GRM3_HUMAN**	Metabotropic glutamate receptor 3	GRM3	Receptors
**GRM4_HUMAN**	Metabotropic glutamate receptor 4	GRM4	Receptors
**NPT1_HUMAN**	Sodium-dependent phosphate transport protein 1	SLC17A1	Transporters
**KI3S1_HUMAN**	Killer cell immunoglobulin-like receptor 3DS1	KIR3DS1	Receptors
**KI2S3_HUMAN**	Killer cell immunoglobulin-like receptor 2DS3	KIR2DS3	Receptors
**KI2S5_HUMAN**	Killer cell immunoglobulin-like receptor 2DS5	KIR2DS5	Receptors
**KI2S1_HUMAN**	Killer cell immunoglobulin-like receptor 2DS1	KIR2DS1	Receptors
**GPNMB_HUMAN**	Transmembrane glycoprotein NMB	GPNMB	Unclassified
**NMDE3_HUMAN**	Glutamate receptor ionotropic, NMDA 2C	GRIN2C	Transporters
**NTCP_HUMAN**	Sodium/bile acid cotransporter	SLC10A1	Transporters
**OPCM_HUMAN**	Opioid-binding protein/cell adhesion molecule	OPCML	Unclassified
**T132D_HUMAN**	Transmembrane protein 132D	TMEM132D	Miscellaneous
**CLCA4_HUMAN**	Calcium-activated chloride channel regulator 4	CLCA4	Unclassified
**HECAM_HUMAN**	Hepatocyte cell adhesion molecule	HEPACAM	Unclassified
**T132B_HUMAN**	Transmembrane protein 132B	TMEM132B	Miscellaneous
**S39AE_HUMAN**	Zinc transporter ZIP14	SLC39A14	
**P2RY6_HUMAN**	P2Y purinoceptor 6	P2RY6	Receptors
**RAGE_HUMAN**	Advanced glycosylation end product-specific receptor	AGER	Receptors
**PDCD1_HUMAN**	Programmed cell death protein 1	PDCD1	Unclassified
**EBP_HUMAN**	3-beta-hydroxysteroid-Delta(8),Delta(7)-isomerase	EBP	Enzymes
**PVRL1_HUMAN**	Nectin-1	PVRL1	Receptors
**PTPRR_HUMAN**	Receptor-type tyrosine-protein phosphatase R	PTPRR	Receptors
**PTPRK_HUMAN**	Receptor-type tyrosine-protein phosphatase kappa	PTPRK	Receptors
**ERBB4_HUMAN**	Receptor tyrosine-protein kinase erbB-4	ERBB4	Receptors
**EPHA7_HUMAN**	Ephrin type-A receptor 7	EPHA7	Receptors
**P2Y14_HUMAN**	P2Y purinoceptor 14	P2RY14	Receptors
**TLR1_HUMAN**	Toll-like receptor 1	TLR1	Receptors
**OR1Q1_HUMAN**	Olfactory receptor 1Q1	OR1Q1	Receptors
**OR8G2_HUMAN**	Olfactory receptor 8G2	OR8G2	Receptors
**OR4D1_HUMAN**	Olfactory receptor 4D1	OR4D1	Receptors
**OR8G1_HUMAN**	Olfactory receptor 8G1	OR8G1	Receptors
**OR1C1_HUMAN**	Olfactory receptor 1C1	OR1C1	Receptors
**OR8B8_HUMAN**	Olfactory receptor 8B8	OR8B8	Receptors
**OR7A5_HUMAN**	Olfactory receptor 7A5	OR7A5	Receptors

**LT4R1_HUMAN**	Leukotriene B4 receptor 1	LTB4R	Receptors
**OGR1_HUMAN**	Ovarian cancer G-protein coupled receptor 1	GPR68	Receptors
**AAAT_HUMAN**	Neutral amino acid transporter B(0)	SLC1A5	Transporters
**GPR19_HUMAN**	Probable G-protein coupled receptor 19	GPR19	Receptors
**NPY5R_HUMAN**	Neuropeptide Y receptor type 5	NPY5R	Receptors
**CD226_HUMAN**	CD226 antigen	CD226	Receptors
**EFNB3_HUMAN**	Ephrin-B3	EFNB3	Miscellaneous
**ACHA2_HUMAN**	Neuronal acetylcholine receptor subunit alpha-2	CHRNA2	Transporters
**ACHA6_HUMAN**	Neuronal acetylcholine receptor subunit alpha-6	CHRNA6	Transporters
**UT2_HUMAN**	Urea transporter 2	SLC14A2	Transporters
**SCN9A_HUMAN**	Sodium channel protein type 9 subunit alpha	SCN9A	Transporters
**GRIK4_HUMAN**	Glutamate receptor ionotropic, kainate 4	GRIK4	Transporters
**NTRK3_HUMAN**	NT-3 growth factor receptor	NTRK3	Receptors
**S15A2_HUMAN**	Solute carrier family 15 member 2	SLC15A2	Transporters
**GBRA6_HUMAN**	Gamma-aminobutyric acid receptor subunit alpha-6	GABRA6	Transporters
**GRIK5_HUMAN**	Glutamate receptor ionotropic, kainate 5	GRIK5	Transporters
**GP162_HUMAN**	Probable G-protein coupled receptor 162	GPR162	Receptors
**LY6E_HUMAN**	Lymphocyte antigen 6E	LY6E	Unclassified
**KCMB1_HUMAN**	Calcium-activated potassium channel subunit beta-1	KCNMB1	Transporters
**SYPL1_HUMAN**	Synaptophysin-like protein 1	SYPL1	Miscellaneous
**ACKR1_HUMAN**	Atypical chemokine receptor 1	ACKR1	Receptors
**VACHT_HUMAN**	Vesicular acetylcholine transporter	SLC18A3	Transporters
**C3AR_HUMAN**	C3a anaphylatoxin chemotactic receptor	C3AR1	Receptors
**SGCB_HUMAN**	Beta-sarcoglycan	SGCB	Miscellaneous
**SGCA_HUMAN**	Alpha-sarcoglycan	SGCA	Miscellaneous
**CALRL_HUMAN**	Calcitonin gene-related peptide type 1 receptor	CALCRL	Receptors
**NTRK2_HUMAN**	BDNF/NT-3 growth factors receptor	NTRK2	Receptors
**PRSS8_HUMAN**	Prostasin	PRSS8	Unclassified
**MOG_HUMAN**	Myelin-oligodendrocyte glycoprotein	MOG	Unclassified
**AMHR2_HUMAN**	Anti-Muellerian hormone type-2 receptor	AMHR2	Receptors
**AT2B3_HUMAN**	Plasma membrane calcium-transporting ATPase 3	ATP2B3	Transporters
**MEP1A_HUMAN**	Meprin A subunit alpha	MEP1A	Enzymes
**MEP1B_HUMAN**	Meprin A subunit beta	MEP1B	Enzymes
**PTPRO_HUMAN**	Receptor-type tyrosine-protein phosphatase O	PTPRO	Receptors
**DDR2_HUMAN**	Discoidin domain-containing receptor 2	DDR2	Receptors
**PTPRN_HUMAN**	Receptor-type tyrosine-protein phosphatase-like N	PTPRN	Receptors
**AOC3_HUMAN**	Membrane primary amine oxidase	AOC3	
**CGT_HUMAN**	2-hydroxyacylsphingosine 1-beta-galactosyltransferase	UGT8	Enzymes
**F187B_HUMAN**	Protein FAM187B	FAM187B	Unclassified
**LY6K_HUMAN**	Lymphocyte antigen 6K	LY6K	Unclassified

**ECSCR_HUMAN**	Endothelial cell-specific chemotaxis regulator	ECSCR	
**SC5AC_HUMAN**	Sodium-coupled monocarboxylate transporter 2	SLC5A12	Transporters
**DOXA1_HUMAN**	Dual oxidase maturation factor 1	DUOXA1	Unclassified
**T132A_HUMAN**	Transmembrane protein 132A	TMEM132A	Miscellaneous
**1B82_HUMAN**	HLA class I histocompatibility antigen, B-82 alpha chain	HLA-B	Miscellaneous
**1B67_HUMAN**	HLA class I histocompatibility antigen, B-67 alpha chain	HLA-B	Miscellaneous
**1C18_HUMAN**	HLA class I histocompatibility antigen, Cw-18 alpha chain	HLA-C	Miscellaneous
**1B59_HUMAN**	HLA class I histocompatibility antigen, B-59 alpha chain	HLA-B	Miscellaneous
**1C16_HUMAN**	HLA class I histocompatibility antigen, Cw-16 alpha chain	HLA-C	Miscellaneous
**1C06_HUMAN**	HLA class I histocompatibility antigen, Cw-6 alpha chain	HLA-C	Miscellaneous
**2B1G_HUMAN**	HLA class II histocompatibility antigen, DRB1-16 beta chain	HLA-DRB1	Miscellaneous
**MICB_HUMAN**	MHC class I polypeptide-related sequence B	MICB	Miscellaneous
**MICA_HUMAN**	MHC class I polypeptide-related sequence A	MICA	Miscellaneous
**CL12B_HUMAN**	C-type lectin domain family 12 member B	CLEC12B	Receptors
**GSG1_HUMAN**	Germ cell-specific gene 1 protein	GSG1	Unclassified
**MPEG1_HUMAN**	Macrophage-expressed gene 1 protein	MPEG1	Unclassified
**ABCB5_HUMAN**	ATP-binding cassette sub-family B member 5	ABCB5	Transporters
**SC5A9_HUMAN**	Sodium/glucose cotransporter 4	SLC5A9	Transporters
**PRTG_HUMAN**	Protogenin	PRTG	Receptors
**S4A8_HUMAN**	Electroneutral sodium bicarbonate exchanger 1	SLC4A8	Transporters
**2B18_HUMAN**	HLA class II histocompatibility antigen, DRB1-8 beta chain	HLA-DRB1	Miscellaneous
**DRB5_HUMAN**	HLA class II histocompatibility antigen, DR beta 5 chain	HLA-DRB5	Miscellaneous
**2B1A_HUMAN**	HLA class II histocompatibility antigen, DRB1-10 beta chain	HLA-DRB1	Miscellaneous
**HFE_HUMAN**	Hereditary hemochromatosis protein	HFE	Unclassified
**1B81_HUMAN**	HLA class I histocompatibility antigen, B-81 alpha chain	HLA-B	Miscellaneous
**1B73_HUMAN**	HLA class I histocompatibility antigen, B-73 alpha chain	HLA-B	Miscellaneous
**LPPR5_HUMAN**	Lipid phosphate phosphatase-related protein type 5	LPPR5	Enzymes
**PTHD3_HUMAN**	Patched domain-containing protein 3	PTCHD3	Unclassified
**TMM95_HUMAN**	Transmembrane protein 95	TMEM95	Unclassified
**SOAT_HUMAN**	Solute carrier family 10 member 6	SLC10A6	Transporters
**CEA21_HUMAN**	Carcinoembryonic antigen-related cell adhesion molecule 21	CEACAM21	Miscellaneous
**GRM1B_HUMAN**	GRAM domain-containing protein 1B	GRAMD1B	Unclassified
**DPCR1_HUMAN**	Diffuse panbronchiolitis critical region protein 1	DPCR1	Unclassified
**K1644_HUMAN**	Uncharacterized protein KIAA1644	KIAA1644	Unclassified
**LRIT3_HUMAN**	Leucine-rich repeat, immunoglobulin-like domain and transmembrane domain-containing protein 3	LRIT3	Miscellaneous
**F174B_HUMAN**	Membrane protein FAM174B	FAM174B	Unclassified
**ZACN_HUMAN**	Zinc-activated ligand-gated ion channel	ZACN	Transporters
**TIGIT_HUMAN**	T-cell immunoreceptor with Ig and ITIM domains	TIGIT	Miscellaneous
**S36A2_HUMAN**	Proton-coupled amino acid transporter 2	SLC36A2	Transporters
**S36A3_HUMAN**	Proton-coupled amino acid transporter 3	SLC36A3	Transporters

**MMEL1_HUMAN**	Membrane metallo-endopeptidase-like 1	MMEL1	Enzymes
**CLM2_HUMAN**	CMRF35-like molecule 2	CD300E	Receptors
**NRN1L_HUMAN**	Neuritin-like protein	NRN1L	
**SV2C_HUMAN**	Synaptic vesicle glycoprotein 2C	SV2C	Transporters
**GPR33_HUMAN**	Probable G-protein coupled receptor 33	GPR33	Receptors
**SRCRM_HUMAN**	Scavenger receptor cysteine-rich domain-containing protein SCART1		Unclassified
**CDON_HUMAN**	Cell adhesion molecule-related/down-regulated by oncogenes	CDON	Receptors
**ANO6_HUMAN**	Anoctamin-6	ANO6	Miscellaneous
**NKAI1_HUMAN**	Sodium/potassium-transporting ATPase subunit beta-1-interacting protein 1	NKAIN1	Transporters
**S22A6_HUMAN**	Solute carrier family 22 member 6	SLC22A6	Transporters
**AT135_HUMAN**	Probable cation-transporting ATPase 13A5	ATP13A5	Transporters
**S39AC_HUMAN**	Zinc transporter ZIP12	SLC39A12	Transporters
**LRC24_HUMAN**	Leucine-rich repeat-containing protein 24	LRRC24	Miscellaneous
**SEZ6_HUMAN**	Seizure protein 6 homolog	SEZ6	Unclassified
**CTL4_HUMAN**	Choline transporter-like protein 4	SLC44A4	Transporters
**T4S20_HUMAN**	Transmembrane 4 L6 family member 20	TM4SF20	Miscellaneous
**SDK2_HUMAN**	Protein sidekick-2	SDK2	Miscellaneous
**SPRN_HUMAN**	Shadow of prion protein	SPRN	
**S45A4_HUMAN**	Solute carrier family 45 member 4	SLC45A4	Transporters
**UROL1_HUMAN**	Uromodulin-like 1	UMODL1	Unclassified
**IGS11_HUMAN**	Immunoglobulin superfamily member 11	IGSF11	Receptors
**PRRT3_HUMAN**	Proline-rich transmembrane protein 3	PRRT3	Unclassified
**XKR3_HUMAN**	XK-related protein 3	XKR3	Transporters
**MKS3_HUMAN**	Meckelin	TMEM67	Unclassified
**CRUM2_HUMAN**	Protein crumbs homolog 2	CRB2	Miscellaneous
**OR2BB_HUMAN**	Olfactory receptor 2B11	OR2B11	Receptors
**O10J3_HUMAN**	Olfactory receptor 10J3	OR10J3	Receptors
**T255A_HUMAN**	Transmembrane protein 255A	TMEM255A	Unclassified
**SIRB2_HUMAN**	Signal-regulatory protein beta-2	SIRPB2	Miscellaneous
**GLRA4_HUMAN**	Glycine receptor subunit alpha-4	GLRA4	Transporters
**EPHAA_HUMAN**	Ephrin type-A receptor 10	EPHA10	Receptors
**RNFT1_HUMAN**	RING finger and transmembrane domain-containing protein 1	RNFT1	Miscellaneous
**FFAR4_HUMAN**	Free fatty acid receptor 4	FFAR4	Receptors
**NTCP5_HUMAN**	Sodium/bile acid cotransporter 5	SLC10A5	Transporters
**CL12A_HUMAN**	C-type lectin domain family 12 member A	CLEC12A	Receptors
**PPR29_HUMAN**	Protein phosphatase 1 regulatory subunit 29	ELFN2	Miscellaneous
**LY66F_HUMAN**	Lymphocyte antigen 6 complex locus protein G6f	LY6G6F	Unclassified
**MUC21_HUMAN**	Mucin-21	MUC21	Unclassified
**CA101_HUMAN**	Uncharacterized protein C1orf101	C1orf101	Unclassified
**FREM2_HUMAN**	FRAS1-related extracellular matrix protein 2	FREM2	Unclassified

**NCMAP_HUMAN**	Noncompact myelin-associated protein	NCMAP	Unclassified
**TRML2_HUMAN**	Trem-like transcript 2 protein	TREML2	Receptors
**CSCL2_HUMAN**	CSC1-like protein 2	TMEM63B	Miscellaneous
**MRP7_HUMAN**	Multidrug resistance-associated protein 7	ABCC10	Transporters
**CXG2_HUMAN**	Gap junction gamma-2 protein	GJC2	Miscellaneous
**ZFY27_HUMAN**	Protrudin	ZFYVE27	Unclassified
**GP110_HUMAN**	Probable G-protein coupled receptor 110	GPR110	Receptors
**GPC6A_HUMAN**	G-protein coupled receptor family C group 6 member A	GPRC6A	Receptors
**GP158_HUMAN**	Probable G-protein coupled receptor 158	GPR158	Receptors
**NAGT1_HUMAN**	Sodium-dependent glucose transporter 1	NAGLT1	Unclassified
**SIRBL_HUMAN**	Signal-regulatory protein beta-1 isoform 3	SIRPB1	Miscellaneous
**OR2G6_HUMAN**	Olfactory receptor 2G6	OR2G6	Receptors
**GP157_HUMAN**	Probable G-protein coupled receptor 157	GPR157	Receptors
**LRC38_HUMAN**	Leucine-rich repeat-containing protein 38	LRRC38	Miscellaneous
**VSIG8_HUMAN**	V-set and immunoglobulin domain-containing protein 8	VSIG8	Miscellaneous
**P210L_HUMAN**	Nuclear pore membrane glycoprotein 210-like	NUP210L	Unclassified
**CAHD1_HUMAN**	VWFA and cache domain-containing protein 1	CACHD1	Transporters
**F1711_HUMAN**	Protein FAM171A1	FAM171A1	Unclassified
**K0319_HUMAN**	Dyslexia-associated protein KIAA0319	KIAA0319	Unclassified
**ATRN1_HUMAN**	Attractin-like protein 1	ATRNL1	Unclassified
**GP107_HUMAN**	Protein GPR107	GPR107	Receptors
**IL23R_HUMAN**	Interleukin-23 receptor	IL23R	Receptors
**SUSD4_HUMAN**	Sushi domain-containing protein 4	SUSD4	Unclassified
**NKAI2_HUMAN**	Sodium/potassium-transporting ATPase subunit beta-1-interacting protein 2	NKAIN2	Transporters
**PEAR1_HUMAN**	Platelet endothelial aggregation receptor 1	PEAR1	Receptors
**RET1L_HUMAN**	Retinoic acid early transcript 1L protein	RAET1L	Miscellaneous
**MALR1_HUMAN**	MAM and LDL-receptor class A domain-containing protein 1	MALRD1	Receptors
**IZUM3_HUMAN**	Izumo sperm-egg fusion protein 3	IZUMO3	Unclassified
**ANO1_HUMAN**	Anoctamin-1	ANO1	Miscellaneous
**2B1D_HUMAN**	HLA class II histocompatibility antigen, DRB1-13 beta chain	HLA-DRB1	Miscellaneous
**CD276_HUMAN**	CD276 antigen	CD276	Miscellaneous
**MUC17_HUMAN**	Mucin-17	MUC17	Unclassified
**STEA4_HUMAN**	Metalloreductase STEAP4	STEAP4	Transporters
**NR3L1_HUMAN**	Natural cytotoxicity triggering receptor 3 ligand 1	NCR3LG1	Unclassified
**LMBD2_HUMAN**	LMBR1 domain-containing protein 2	LMBRD2	Unclassified
**RNF43_HUMAN**	E3 ubiquitin-protein ligase RNF43	RNF43	Unclassified
**ENK6_HUMAN**	Endogenous retrovirus group K member 6 Env polyprotein	ERVK-6	Miscellaneous
**S6A19_HUMAN**	Sodium-dependent neutral amino acid transporter B(0)AT1	SLC6A19	Transporters
**FCRL6_HUMAN**	Fc receptor-like protein 6	FCRL6	Receptors
**GP139_HUMAN**	Probable G-protein coupled receptor 139	GPR139	Receptors

**VASN_HUMAN**	Vasorin	VASN	Miscellaneous
**LAIR1_HUMAN**	Leukocyte-associated immunoglobulin-like receptor 1	LAIR1	Unclassified
**TM225_HUMAN**	Transmembrane protein 225	TMEM225	Unclassified
**RET1G_HUMAN**	Retinoic acid early transcript 1G protein	RAET1G	Miscellaneous
**SIGIR_HUMAN**	Single Ig IL-1-related receptor	SIGIRR	Receptors
**TM211_HUMAN**	Transmembrane protein 211	TMEM211	Miscellaneous
**T132E_HUMAN**	Transmembrane protein 132E	TMEM132E	Miscellaneous
**OR5MA_HUMAN**	Olfactory receptor 5M10	OR5M10	Receptors
**OR4CB_HUMAN**	Olfactory receptor 4C11	OR4C11	Receptors
**OR4F3_HUMAN**	Olfactory receptor 4F3/4F16/4F29	OR4F3	Receptors
**OR2T5_HUMAN**	Olfactory receptor 2T5	OR2T5	Receptors
**OR2T2_HUMAN**	Olfactory receptor 2T2	OR2T2	Receptors
**OR2A2_HUMAN**	Olfactory receptor 2A2	OR2A2	Receptors
**O52W1_HUMAN**	Olfactory receptor 52W1	OR52W1	Receptors
**O4A47_HUMAN**	Olfactory receptor 4A47	OR4A47	Receptors
**O10K2_HUMAN**	Olfactory receptor 10K2	OR10K2	Receptors
**O52E8_HUMAN**	Olfactory receptor 52E8	OR52E8	Receptors
**OR6B2_HUMAN**	Olfactory receptor 6B2	OR6B2	Receptors
**O7E24_HUMAN**	Olfactory receptor 7E24	OR7E24	Receptors
**PTCRA_HUMAN**	Pre T-cell antigen receptor alpha	PTCRA	
**ANO7_HUMAN**	Anoctamin-7	ANO7	Miscellaneous
**NCKX6_HUMAN**	Sodium/potassium/calcium exchanger 6, mitochondrial	SLC8B1	Transporters
**HPHL1_HUMAN**	Hephaestin-like protein 1	HEPHL1	Unclassified
**TEN4_HUMAN**	Teneurin-4	TENM4	Miscellaneous
**MFSD5_HUMAN**	Molybdate-anion transporter	MFSD5	Unclassified
**CK087_HUMAN**	Uncharacterized protein C11orf87	C11orf87	Unclassified
**TECT3_HUMAN**	Tectonic-3	TCTN3	Unclassified
**MFS12_HUMAN**	Major facilitator superfamily domain-containing protein 12	MFSD12	Unclassified
**GP153_HUMAN**	Probable G-protein coupled receptor 153	GPR153	Receptors
**NIPA3_HUMAN**	Magnesium transporter NIPA3	NIPAL1	Transporters
**RGMB_HUMAN**	RGM domain family member B	RGMB	
**PLB1_HUMAN**	Phospholipase B1, membrane-associated	PLB1	Unclassified
**NPAL3_HUMAN**	NIPA-like protein 3	NIPAL3	Transporters
**CNNM4_HUMAN**	Metal transporter CNNM4	CNNM4	Unclassified
**S39A4_HUMAN**	Zinc transporter ZIP4	SLC39A4	Transporters
**F171B_HUMAN**	Protein FAM171B	FAM171B	Unclassified
**TM154_HUMAN**	Transmembrane protein 154	TMEM154	Unclassified
**EMB_HUMAN**	Embigin	EMB	Unclassified
**CXB7_HUMAN**	Gap junction beta-7 protein	GJB7	Miscellaneous
**LIRA6_HUMAN**	Leukocyte immunoglobulin-like receptor subfamily A member 6	LILRA6	Receptors

**TRAT1_HUMAN**	T-cell receptor-associated transmembrane adapter 1	TRAT1	Unclassified
**RHDF2_HUMAN**	Inactive rhomboid protein 2	RHBDF2	Unclassified
**LRFN4_HUMAN**	Leucine-rich repeat and fibronectin type-III domain-containing protein 4	LRFN4	Miscellaneous
**GP179_HUMAN**	Probable G-protein coupled receptor 179	GPR179	Receptors
**GTR7_HUMAN**	Solute carrier family 2, facilitated glucose transporter member 7	SLC2A7	Transporters
**MO2R2_HUMAN**	Cell surface glycoprotein CD200 receptor 2	CD200R1L	Receptors
**GP133_HUMAN**	Probable G-protein coupled receptor 133	GPR133	Receptors
**S22AP_HUMAN**	Solute carrier family 22 member 25	SLC22A25	Transporters
**OPN5_HUMAN**	Opsin-5	OPN5	Receptors
**S4A10_HUMAN**	Sodium-driven chloride bicarbonate exchanger	SLC4A10	Transporters
**PLET1_HUMAN**	Placenta-expressed transcript 1 protein	PLET1	
**CSPG4_HUMAN**	Chondroitin sulfate proteoglycan 4	CSPG4	Unclassified
**ARAID_HUMAN**	All-trans retinoic acid-induced differentiation factor	ATRAID	Unclassified
**EPGN_HUMAN**	Epigen	EPGN	Unclassified
**I27RA_HUMAN**	Interleukin-27 receptor subunit alpha	IL27RA	Unclassified
**PARM1_HUMAN**	Prostate androgen-regulated mucin-like protein 1	PARM1	Unclassified
**TMCO3_HUMAN**	Transmembrane and coiled-coil domain-containing protein 3	TMCO3	Unclassified
**C16L2_HUMAN**	CD164 sialomucin-like 2 protein	CD164L2	Unclassified
**SUSD1_HUMAN**	Sushi domain-containing protein 1	SUSD1	Unclassified
**KIRR2_HUMAN**	Kin of IRRE-like protein 2	KIRREL2	Miscellaneous
**LYPD4_HUMAN**	Ly6/PLAUR domain-containing protein 4	LYPD4	
**LYPD5_HUMAN**	Ly6/PLAUR domain-containing protein 5	LYPD5	Unclassified
**ENPP6_HUMAN**	Ectonucleotide pyrophosphatase/phosphodiesterase family member 6	ENPP6	
**MPZL3_HUMAN**	Myelin protein zero-like protein 3	MPZL3	Miscellaneous
**LMBRL_HUMAN**	Protein LMBR1L	LMBR1L	Unclassified
**LAYN_HUMAN**	Layilin	LAYN	Receptors
**VSTM1_HUMAN**	V-set and transmembrane domain-containing protein 1	VSTM1	Miscellaneous
**PXDC2_HUMAN**	Plexin domain-containing protein 2	PLXDC2	Unclassified
**LYPD8_HUMAN**	Ly6/PLAUR domain-containing protein 8	LYPD8	Unclassified
**LYPD2_HUMAN**	Ly6/PLAUR domain-containing protein 2	LYPD2	
**CLC4G_HUMAN**	C-type lectin domain family 4 member G	CLEC4G	Receptors
**PI16_HUMAN**	Peptidase inhibitor 16	PI16	Unclassified
**AEGP_HUMAN**	Apical endosomal glycoprotein	MAMDC4	Unclassified
**SE6L2_HUMAN**	Seizure 6-like protein 2	SEZ6L2	Unclassified
**MFSD7_HUMAN**	Major facilitator superfamily domain-containing protein 7	MFSD7	Unclassified
**BTNL3_HUMAN**	Butyrophilin-like protein 3	BTNL3	Miscellaneous
**TM108_HUMAN**	Transmembrane protein 108	TMEM108	Unclassified
**K1324_HUMAN**	UPF0577 protein KIAA1324	KIAA1324	Unclassified
**CLM9_HUMAN**	CMRF35-like molecule 9	CD300LG	Receptors
**BTNL9_HUMAN**	Butyrophilin-like protein 9	BTNL9	Miscellaneous

**ISLR2_HUMAN**	Immunoglobulin superfamily containing leucine-rich repeat protein 2	ISLR2	Miscellaneous
**LRRN1_HUMAN**	Leucine-rich repeat neuronal protein 1	LRRN1	Miscellaneous
**I20RB_HUMAN**	Interleukin-20 receptor subunit beta	IL20RB	Receptors
**LRIG3_HUMAN**	Leucine-rich repeats and immunoglobulin-like domains protein 3	LRIG3	Miscellaneous
**CLC9A_HUMAN**	C-type lectin domain family 9 member A	CLEC9A	Receptors
**GSG1L_HUMAN**	Germ cell-specific gene 1-like protein	GSG1L	Unclassified
**GFRAL_HUMAN**	GDNF family receptor alpha-like	GFRAL	Unclassified
**TMIG1_HUMAN**	Transmembrane and immunoglobulin domain-containing protein 1	TMIGD1	Unclassified
**CLM4_HUMAN**	CMRF35-like molecule 4	CD300LD	Receptors
**UNC5D_HUMAN**	Netrin receptor UNC5D	UNC5D	Receptors
**CEA20_HUMAN**	Carcinoembryonic antigen-related cell adhesion molecule 20	CEACAM20	Miscellaneous
**DLK2_HUMAN**	Protein delta homolog 2	DLK2	Miscellaneous
**LIGO4_HUMAN**	Leucine-rich repeat and immunoglobulin-like domain-containing nogo receptor-interacting protein 4	LINGO4	Miscellaneous
**FAT4_HUMAN**	Protocadherin Fat 4	FAT4	Miscellaneous
**PCD23_HUMAN**	Protocadherin-23	DCHS2	Miscellaneous
**NPSR1_HUMAN**	Neuropeptide S receptor	NPSR1	Receptors
**S36A4_HUMAN**	Proton-coupled amino acid transporter 4	SLC36A4	Transporters
**CD109_HUMAN**	CD109 antigen	CD109	
**T184A_HUMAN**	Transmembrane protein 184A	TMEM184A	Unclassified
**SIG15_HUMAN**	Sialic acid-binding Ig-like lectin 15	SIGLEC15	Miscellaneous
**SPNS3_HUMAN**	Protein spinster homolog 3	SPNS3	Transporters
**S39A5_HUMAN**	Zinc transporter ZIP5	SLC39A5	Transporters
**GLDN_HUMAN**	Gliomedin	GLDN	
**SCAR5_HUMAN**	Scavenger receptor class A member 5	SCARA5	Unclassified
**UNC5A_HUMAN**	Netrin receptor UNC5A	UNC5A	Receptors
**FRRS1_HUMAN**	Ferric-chelate reductase 1	FRRS1	Unclassified
**LAAT1_HUMAN**	Lysosomal amino acid transporter 1 homolog	PQLC2	Unclassified
**TM182_HUMAN**	Transmembrane protein 182	TMEM182	Unclassified
**SO4C1_HUMAN**	Solute carrier organic anion transporter family member 4C1	SLCO4C1	Transporters
**CTSRG_HUMAN**	Cation channel sperm-associated protein subunit gamma	CATSPERG	Unclassified
**QSOX2_HUMAN**	Sulfhydryl oxidase 2	QSOX2	Enzymes
**CL17A_HUMAN**	C-type lectin domain family 17, member A	CLEC17A	Receptors
**SHSA6_HUMAN**	Protein shisa-6 homolog	SHISA6	
**MOT12_HUMAN**	Monocarboxylate transporter 12	SLC16A12	Transporters
**MFSD6_HUMAN**	Major facilitator superfamily domain-containing protein 6	MFSD6	Unclassified
**CDHR3_HUMAN**	Cadherin-related family member 3	CDHR3	Unclassified
**TMM26_HUMAN**	Transmembrane protein 26	TMEM26	Unclassified
**K154L_HUMAN**	UPF0606 protein KIAA1549L	KIAA1549L	Unclassified
**RGMC_HUMAN**	Hemojuvelin	HFE2	Unclassified
**PTHD4_HUMAN**	Patched domain-containing protein 4	PTCHD4	Unclassified

**5HT3D_HUMAN**	5-hydroxytryptamine receptor 3D	HTR3D	Transporters
**NCKX5_HUMAN**	Sodium/potassium/calcium exchanger 5	SLC24A5	Transporters
**PGAP1_HUMAN**	GPI inositol-deacylase	PGAP1	Unclassified
**ANO5_HUMAN**	Anoctamin-5	ANO5	Miscellaneous
**BT2A1_HUMAN**	Butyrophilin subfamily 2 member A1	BTN2A1	Miscellaneous
**SV2A_HUMAN**	Synaptic vesicle glycoprotein 2A	SV2A	Transporters
**TRIL_HUMAN**	TLR4 interactor with leucine rich repeats	TRIL	Miscellaneous
**SV2B_HUMAN**	Synaptic vesicle glycoprotein 2B	SV2B	Transporters
**LIGO2_HUMAN**	Leucine-rich repeat and immunoglobulin-like domain-containing nogo receptor-interacting protein 2	LINGO2	Miscellaneous
**S26A9_HUMAN**	Solute carrier family 26 member 9	SLC26A9	Transporters
**OTOP1_HUMAN**	Otopetrin-1	OTOP1	Unclassified
**OTOP2_HUMAN**	Otopetrin-2	OTOP2	Unclassified
**S29A4_HUMAN**	Equilibrative nucleoside transporter 4	SLC29A4	Transporters
**OTOAN_HUMAN**	Otoancorin	OTOA	
**TS1R3_HUMAN**	Taste receptor type 1 member 3	TAS1R3	Receptors
**TS1R1_HUMAN**	Taste receptor type 1 member 1	TAS1R1	Receptors
**PRS41_HUMAN**	Putative serine protease 41	PRSS41	
**LPPR4_HUMAN**	Lipid phosphate phosphatase-related protein type 4	LPPR4	Enzymes
**S36A1_HUMAN**	Proton-coupled amino acid transporter 1	SLC36A1	Transporters
**ERMP1_HUMAN**	Endoplasmic reticulum metallopeptidase 1	ERMP1	Unclassified
**NEGR1_HUMAN**	Neuronal growth regulator 1	NEGR1	Unclassified
**ATG9A_HUMAN**	Autophagy-related protein 9A	ATG9A	Unclassified
**LYSM3_HUMAN**	LysM and putative peptidoglycan-binding domain-containing protein 3	LYSMD3	Unclassified
**GP155_HUMAN**	Integral membrane protein GPR155	GPR155	Unclassified
**S46A3_HUMAN**	Solute carrier family 46 member 3	SLC46A3	Transporters
**OR2W3_HUMAN**	Olfactory receptor 2W3	OR2W3	Receptors
**TMC7_HUMAN**	Transmembrane channel-like protein 7	TMC7	Unclassified
**CSMD2_HUMAN**	CUB and sushi domain-containing protein 2	CSMD2	Miscellaneous
**KCNKI_HUMAN**	Potassium channel subfamily K member 18	KCNK18	Transporters
**PK1L2_HUMAN**	Polycystic kidney disease protein 1-like 2	PKD1L2	Transporters
**PK1L3_HUMAN**	Polycystic kidney disease protein 1-like 3	PKD1L3	Transporters
**LRP10_HUMAN**	Low-density lipoprotein receptor-related protein 10	LRP10	Receptors
**MDGA2_HUMAN**	MAM domain-containing glycosylphosphatidylinositol anchor protein 2	MDGA2	Unclassified
**VN1R4_HUMAN**	Vomeronasal type-1 receptor 4	VN1R4	Receptors
**SDK1_HUMAN**	Protein sidekick-1	SDK1	Miscellaneous
**GP142_HUMAN**	Probable G-protein coupled receptor 142	GPR142	Receptors
**GP141_HUMAN**	Probable G-protein coupled receptor 141	GPR141	Receptors
**CEA19_HUMAN**	Carcinoembryonic antigen-related cell adhesion molecule 19	CEACAM19	Miscellaneous
**BTLA_HUMAN**	B- and T-lymphocyte attenuator	BTLA	Unclassified

**MILR1_HUMAN**	Allergin-1	MILR1	Miscellaneous
**VTCN1_HUMAN**	V-set domain-containing T-cell activation inhibitor 1	VTCN1	Miscellaneous
**LHPL4_HUMAN**	Lipoma HMGIC fusion partner-like 4 protein	LHFPL4	Miscellaneous
**MEGF8_HUMAN**	Multiple epidermal growth factor-like domains protein 8	MEGF8	Unclassified
**GP144_HUMAN**	Probable G-protein coupled receptor 144	GPR144	Receptors
**T179B_HUMAN**	Transmembrane protein 179B	TMEM179B	Unclassified
**AMGO2_HUMAN**	Amphoterin-induced protein 2	AMIGO2	Miscellaneous
**DSG4_HUMAN**	Desmoglein-4	DSG4	Miscellaneous
**MRGRG_HUMAN**	Mas-related G-protein coupled receptor member G	MRGPRG	Receptors
**MRGRE_HUMAN**	Mas-related G-protein coupled receptor member E	MRGPRE	Receptors
**GP149_HUMAN**	Probable G-protein coupled receptor 149	GPR149	Unclassified
**EMR4_HUMAN**	Putative EGF-like module-containing mucin-like hormone receptor-like 4	EMR4P	Receptors
**GP126_HUMAN**	G-protein coupled receptor 126	GPR126	Receptors
**GP123_HUMAN**	Probable G-protein coupled receptor 123	GPR123	Receptors
**ILDR1_HUMAN**	Immunoglobulin-like domain-containing receptor 1	ILDR1	Unclassified
**CLC14_HUMAN**	C-type lectin domain family 14 member A	CLEC14A	Unclassified
**TM11B_HUMAN**	Transmembrane protease serine 11B	TMPRSS11B	Enzymes
**T150A_HUMAN**	Transmembrane protein 150A	TMEM150A	Unclassified
**TM110_HUMAN**	Transmembrane protein 110	TMEM110	Unclassified
**CN037_HUMAN**	Uncharacterized protein C14orf37	C14orf37	Unclassified
**TSN33_HUMAN**	Tetraspanin-33	TSPAN33	Miscellaneous
**SO6A1_HUMAN**	Solute carrier organic anion transporter family member 6A1	SLCO6A1	Transporters
**ABCAC_HUMAN**	ATP-binding cassette sub-family A member 12	ABCA12	Transporters
**LBN_HUMAN**	Limbin	EVC2	Unclassified
**R4RL1_HUMAN**	Reticulon-4 receptor-like 1	RTN4RL1	
**R4RL2_HUMAN**	Reticulon-4 receptor-like 2	RTN4RL2	
**CAD24_HUMAN**	Cadherin-24	CDH24	Miscellaneous
**CUZD1_HUMAN**	CUB and zona pellucida-like domain-containing protein 1	CUZD1	Unclassified
**ABCAD_HUMAN**	ATP-binding cassette sub-family A member 13	ABCA13	Transporters
**OSTA_HUMAN**	Organic solute transporter subunit alpha	SLC51A	Unclassified
**OSTB_HUMAN**	Organic solute transporter subunit beta	SLC51B	Unclassified
**ADR2_HUMAN**	Adiponectin receptor protein 2	ADIPOR2	Receptors
**TIKI1_HUMAN**	Metalloprotease TIKI1	TRABD2A	Unclassified
**GP180_HUMAN**	Integral membrane protein GPR180	GPR180	Unclassified
**C163A_HUMAN**	Scavenger receptor cysteine-rich type 1 protein M130	CD163	Unclassified
**SERC5_HUMAN**	Serine incorporator 5	SERINC5	Transporters
**LRRT4_HUMAN**	Leucine-rich repeat transmembrane neuronal protein 4	LRRTM4	Miscellaneous
**LRRT3_HUMAN**	Leucine-rich repeat transmembrane neuronal protein 3	LRRTM3	Miscellaneous
**VS10L_HUMAN**	V-set and immunoglobulin domain-containing protein 10-like	VSIG10L	Miscellaneous
**S22AG_HUMAN**	Solute carrier family 22 member 16	SLC22A16	Transporters

**P2RY8_HUMAN**	P2Y purinoceptor 8	P2RY8	Receptors
**LRP11_HUMAN**	Low-density lipoprotein receptor-related protein 11	LRP11	Receptors
**TPRA1_HUMAN**	Transmembrane protein adipocyte-associated 1	TPRA1	Unclassified
**KCMB4_HUMAN**	Calcium-activated potassium channel subunit beta-4	KCNMB4	Transporters
**UN93A_HUMAN**	Protein unc-93 homolog A	UNC93A	Unclassified
**OSTM1_HUMAN**	Osteopetrosis-associated transmembrane protein 1	OSTM1	Unclassified
**LHPL1_HUMAN**	Lipoma HMGIC fusion partner-like 1 protein	LHFPL1	Miscellaneous
**PKHL1_HUMAN**	Fibrocystin-L	PKHD1L1	Unclassified
**AMGO1_HUMAN**	Amphoterin-induced protein 1	AMIGO1	Miscellaneous
**AMGO3_HUMAN**	Amphoterin-induced protein 3	AMIGO3	Miscellaneous
**VSIG1_HUMAN**	V-set and immunoglobulin domain-containing protein 1	VSIG1	Miscellaneous
**CTSRD_HUMAN**	Cation channel sperm-associated protein subunit delta	CATSPERD	Unclassified
**PRIMA_HUMAN**	Proline-rich membrane anchor 1	PRIMA1	Unclassified
**GOLI_HUMAN**	E3 ubiquitin-protein ligase RNF130	RNF130	Unclassified
**TM219_HUMAN**	Insulin-like growth factor-binding protein 3 receptor	TMEM219	Unclassified
**FRAS1_HUMAN**	Extracellular matrix protein FRAS1	FRAS1	Unclassified
**GPR97_HUMAN**	Probable G-protein coupled receptor 97	GPR97	Receptors
**NRROS_HUMAN**	Negative regulator of reactive oxygen species	NRROS	Miscellaneous
**TMM25_HUMAN**	Transmembrane protein 25	TMEM25	Unclassified
**LRAD3_HUMAN**	Low-density lipoprotein receptor class A domain-containing protein 3	LDLRAD3	Unclassified
**JAML1_HUMAN**	Junctional adhesion molecule-like	AMICA1	Miscellaneous
**INLR1_HUMAN**	Interferon lambda receptor 1	IFNLR1	Receptors
**TMPS6_HUMAN**	Transmembrane protease serine 6	TMPRSS6	Enzymes
**ABCA9_HUMAN**	ATP-binding cassette sub-family A member 9	ABCA9	Transporters
**SPP2C_HUMAN**	Signal peptide peptidase-like 2C	SPPL2C	Unclassified
**PLDX1_HUMAN**	Plexin domain-containing protein 1	PLXDC1	Unclassified
**RELL1_HUMAN**	RELT-like protein 1	RELL1	Unclassified
**HDBP1_HUMAN**	Glycosylphosphatidylinositol-anchored high density lipoprotein-binding protein 1	GPIHBP1	
**S41A1_HUMAN**	Solute carrier family 41 member 1	SLC41A1	Transporters
**S22A9_HUMAN**	Solute carrier family 22 member 9	SLC22A9	Transporters
**IGDC3_HUMAN**	Immunoglobulin superfamily DCC subclass member 3	IGDCC3	Receptors
**SPNS2_HUMAN**	Protein spinster homolog 2	SPNS2	Transporters
**VSTM4_HUMAN**	V-set and transmembrane domain-containing protein 4	VSTM4	Unclassified
**SLIK4_HUMAN**	SLIT and NTRK-like protein 4	SLITRK4	Miscellaneous
**CTL2_HUMAN**	Choline transporter-like protein 2	SLC44A2	Transporters
**MFS6L_HUMAN**	Major facilitator superfamily domain-containing protein 6-like	MFSD6L	Unclassified
**GP125_HUMAN**	Probable G-protein coupled receptor 125	GPR125	Receptors
**SCN4B_HUMAN**	Sodium channel subunit beta-4	SCN4B	Transporters
**CNTN4_HUMAN**	Contactin-4	CNTN4	Unclassified
**CD302_HUMAN**	CD302 antigen	CD302	Receptors

**OR4N5_HUMAN**	Olfactory receptor 4N5	OR4N5	Receptors
**CAD26_HUMAN**	Cadherin-like protein 26	CDH26	Miscellaneous
**S15A3_HUMAN**	Solute carrier family 15 member 3	SLC15A3	Transporters
**PSYR_HUMAN**	Psychosine receptor	GPR65	Receptors
**TEFF1_HUMAN**	Tomoregulin-1	TMEFF1	Unclassified
**IZUM1_HUMAN**	Izumo sperm-egg fusion protein 1	IZUMO1	Unclassified
**S22AF_HUMAN**	Solute carrier family 22 member 15	SLC22A15	Transporters
**NALCN_HUMAN**	Sodium leak channel non-selective protein	NALCN	Unclassified
**GP116_HUMAN**	Probable G-protein coupled receptor 116	GPR116	Receptors
**GP115_HUMAN**	Probable G-protein coupled receptor 115	GPR115	Receptors
**GP114_HUMAN**	Probable G-protein coupled receptor 114	GPR114	Receptors
**GP113_HUMAN**	Probable G-protein coupled receptor 113	GPR113	Receptors
**GP112_HUMAN**	Probable G-protein coupled receptor 112	GPR112	Receptors
**GP111_HUMAN**	Probable G-protein coupled receptor 111	GPR111	Receptors
**UNC5B_HUMAN**	Netrin receptor UNC5B	UNC5B	Receptors
**GPR64_HUMAN**	G-protein coupled receptor 64	GPR64	Receptors
**KIRR3_HUMAN**	Kin of IRRE-like protein 3	KIRREL3	Miscellaneous
**ABCA7_HUMAN**	ATP-binding cassette sub-family A member 7	ABCA7	Transporters
**APCD1_HUMAN**	Protein APCDD1	APCDD1	Unclassified
**NLGNX_HUMAN**	Neuroligin-4, X-linked	NLGN4X	Miscellaneous
**OR4N4_HUMAN**	Olfactory receptor 4N4	OR4N4	Receptors
**OR8I2_HUMAN**	Olfactory receptor 8I2	OR8I2	Receptors
**VSI10_HUMAN**	V-set and immunoglobulin domain-containing protein 10	VSIG10	Unclassified
**KI2LA_HUMAN**	Killer cell immunoglobulin-like receptor 2DL5A	KIR2DL5A	Receptors
**CADM3_HUMAN**	Cell adhesion molecule 3	CADM3	Miscellaneous
**O5AS1_HUMAN**	Olfactory receptor 5AS1	OR5AS1	Receptors
**NPT2C_HUMAN**	Sodium-dependent phosphate transport protein 2C	SLC34A3	Transporters
**PORIM_HUMAN**	Porimin	TMEM123	Unclassified
**ABCA6_HUMAN**	ATP-binding cassette sub-family A member 6	ABCA6	Transporters
**OR8H3_HUMAN**	Olfactory receptor 8H3	OR8H3	Receptors
**OR6V1_HUMAN**	Olfactory receptor 6V1	OR6V1	Receptors
**LIRA2_HUMAN**	Leukocyte immunoglobulin-like receptor subfamily A member 2	LILRA2	Receptors
**GPC2_HUMAN**	Glypican-2	GPC2	
**OR8H2_HUMAN**	Olfactory receptor 8H2	OR8H2	Receptors
**GBRG1_HUMAN**	Gamma-aminobutyric acid receptor subunit gamma-1	GABRG1	Transporters
**DYNAP_HUMAN**	Dynactin-associated protein	DYNAP	Unclassified
**PROM2_HUMAN**	Prominin-2	PROM2	Unclassified
**LYPD1_HUMAN**	Ly6/PLAUR domain-containing protein 1	LYPD1	Unclassified
**NLGN1_HUMAN**	Neuroligin-1	NLGN1	Miscellaneous
**OR2LD_HUMAN**	Olfactory receptor 2L13	OR2L13	Receptors

**LAT4_HUMAN**	Large neutral amino acids transporter small subunit 4	SLC43A2	Transporters
**LRC25_HUMAN**	Leucine-rich repeat-containing protein 25	LRRC25	Miscellaneous
**MUC15_HUMAN**	Mucin-15	MUC15	Unclassified
**G137C_HUMAN**	Integral membrane protein GPR137C	GPR137C	Unclassified
**CADM2_HUMAN**	Cell adhesion molecule 2	CADM2	Miscellaneous
**T132C_HUMAN**	Transmembrane protein 132C	TMEM132C	Miscellaneous
**LIRB2_HUMAN**	Leukocyte immunoglobulin-like receptor subfamily B member 2	LILRB2	Receptors
**SVOPL_HUMAN**	Putative transporter SVOPL	SVOPL	Transporters
**FGRL1_HUMAN**	Fibroblast growth factor receptor-like 1	FGFRL1	Unclassified
**MFSD4_HUMAN**	Major facilitator superfamily domain-containing protein 4	MFSD4	Unclassified
**RPRML_HUMAN**	Reprimo-like protein	RPRML	Unclassified
**CTL3_HUMAN**	Choline transporter-like protein 3	SLC44A3	Transporters
**FA26E_HUMAN**	Protein FAM26E	FAM26E	Enzymes
**M4A15_HUMAN**	Membrane-spanning 4-domains subfamily A member 15	MS4A15	Miscellaneous
**DPP10_HUMAN**	Inactive dipeptidyl peptidase 10	DPP10	Unclassified
**OR2C3_HUMAN**	Olfactory receptor 2C3	OR2C3	Receptors
**SC5A8_HUMAN**	Sodium-coupled monocarboxylate transporter 1	SLC5A8	Transporters
**S15A4_HUMAN**	Solute carrier family 15 member 4	SLC15A4	Transporters
**IGSF1_HUMAN**	Immunoglobulin superfamily member 1	IGSF1	Receptors
**CLD19_HUMAN**	Claudin-19	CLDN19	Miscellaneous
**I22R1_HUMAN**	Interleukin-22 receptor subunit alpha-1	IL22RA1	Receptors
**CD177_HUMAN**	CD177 antigen	CD177	
**GP161_HUMAN**	G-protein coupled receptor 161	GPR161	Receptors
**PCD20_HUMAN**	Protocadherin-20	PCDH20	Miscellaneous
**KI3L3_HUMAN**	Killer cell immunoglobulin-like receptor 3DL3	KIR3DL3	Receptors
**LRC52_HUMAN**	Leucine-rich repeat-containing protein 52	LRRC52	Miscellaneous
**TM217_HUMAN**	Transmembrane protein 217	TMEM217	Unclassified
**PLD5_HUMAN**	Inactive phospholipase D5	PLD5	Unclassified
**CLD22_HUMAN**	Claudin-22	CLDN22	Miscellaneous
**SIGL1_HUMAN**	SIGLEC family-like protein 1	SIGLECL1	Unclassified
**NKAI3_HUMAN**	Sodium/potassium-transporting ATPase subunit beta-1-interacting protein 3	NKAIN3	Transporters
**LSME1_HUMAN**	Leucine-rich single-pass membrane protein 1	LSMEM1	Unclassified
**DCBD1_HUMAN**	Discoidin, CUB and LCCL domain-containing protein 1	DCBLD1	Unclassified
**LRTM2_HUMAN**	Leucine-rich repeat and transmembrane domain-containing protein 2	LRTM2	Miscellaneous
**NLS1_HUMAN**	Sodium-dependent lysophosphatidylcholine symporter 1	MFSD2A	Unclassified
**I17RC_HUMAN**	Interleukin-17 receptor C	IL17RC	Receptors
**FNDC5_HUMAN**	Fibronectin type III domain-containing protein 5	FNDC5	Unclassified
**S43A3_HUMAN**	Solute carrier family 43 member 3	SLC43A3	Transporters
**SIDT2_HUMAN**	SID1 transmembrane family member 2	SIDT2	Unclassified
**T178A_HUMAN**	Transmembrane protein 178A	TMEM178A	Unclassified

**UBAC2_HUMAN**	Ubiquitin-associated domain-containing protein 2	UBAC2	Unclassified
**TM87A_HUMAN**	Transmembrane protein 87A	TMEM87A	Unclassified
**P5I13_HUMAN**	Tumor protein p53-inducible protein 13	TP53I13	Unclassified
**TM145_HUMAN**	Transmembrane protein 145	TMEM145	Unclassified
**S38A9_HUMAN**	Putative sodium-coupled neutral amino acid transporter 9	SLC38A9	Transporters
**CLC1A_HUMAN**	C-type lectin domain family 1 member A	CLEC1A	Receptors
**RN149_HUMAN**	E3 ubiquitin-protein ligase RNF149	RNF149	Unclassified
**KCT2_HUMAN**	Keratinocyte-associated transmembrane protein 2	KCT2	Unclassified
**NETO2_HUMAN**	Neuropilin and tolloid-like protein 2	NETO2	Unclassified
**SPX3_HUMAN**	Sugar phosphate exchanger 3	SLC37A3	Transporters
**DGLB_HUMAN**	Sn1-specific diacylglycerol lipase beta	DAGLB	Unclassified
**TM116_HUMAN**	Transmembrane protein 116	TMEM116	Unclassified
**CTL5_HUMAN**	Choline transporter-like protein 5	SLC44A5	Transporters
**KREM2_HUMAN**	Kremen protein 2	KREMEN2	Unclassified
**LRN4L_HUMAN**	LRRN4 C-terminal-like protein	LRRN4CL	Unclassified
**GPR26_HUMAN**	G-protein coupled receptor 26	GPR26	Receptors
**VGLU3_HUMAN**	Vesicular glutamate transporter 3	SLC17A8	Transporters
**TM104_HUMAN**	Transmembrane protein 104	TMEM104	Transporters
**CNNM3_HUMAN**	Metal transporter CNNM3	CNNM3	Unclassified
**POPD1_HUMAN**	Blood vessel epicardial substance	BVES	Unclassified
**CS018_HUMAN**	Uncharacterized protein C19orf18	C19orf18	Unclassified
**ACV1C_HUMAN**	Activin receptor type-1C	ACVR1C	Receptors
**NFAM1_HUMAN**	NFAT activation molecule 1	NFAM1	Unclassified
**NCKX4_HUMAN**	Sodium/potassium/calcium exchanger 4	SLC24A4	Transporters
**RAI3_HUMAN**	Retinoic acid-induced protein 3	GPRC5A	Receptors
**PKR2_HUMAN**	Prokineticin receptor 2	PROKR2	Receptors
**CXG3_HUMAN**	Gap junction gamma-3 protein	GJC3	Miscellaneous
**I17RD_HUMAN**	Interleukin-17 receptor D	IL17RD	Receptors
**GP156_HUMAN**	Probable G-protein coupled receptor 156	GPR156	Receptors
**MDGA1_HUMAN**	MAM domain-containing glycosylphosphatidylinositol anchor protein 1	MDGA1	Unclassified
**I17RE_HUMAN**	Interleukin-17 receptor E	IL17RE	Receptors
**DNER_HUMAN**	Delta and Notch-like epidermal growth factor-related receptor	DNER	Unclassified
**BEST4_HUMAN**	Bestrophin-4	BEST4	Transporters
**BEST2_HUMAN**	Bestrophin-2	BEST2	Transporters
**SEM6D_HUMAN**	Semaphorin-6D	SEMA6D	Miscellaneous
**NLGNY_HUMAN**	Neuroligin-4, Y-linked	NLGN4Y	Miscellaneous
**NLGN2_HUMAN**	Neuroligin-2	NLGN2	Miscellaneous
**VN1R2_HUMAN**	Vomeronasal type-1 receptor 2	VN1R2	Receptors
**CADM4_HUMAN**	Cell adhesion molecule 4	CADM4	Unclassified
**TSN14_HUMAN**	Tetraspanin-14	TSPAN14	Miscellaneous

**OR5T1_HUMAN**	Olfactory receptor 5T1	OR5T1	Receptors
**O2T33_HUMAN**	Olfactory receptor 2T33	OR2T33	Receptors
**O2T12_HUMAN**	Olfactory receptor 2T12	OR2T12	Receptors
**OR8G5_HUMAN**	Olfactory receptor 8G5	OR8G5	Receptors
**OR2L5_HUMAN**	Olfactory receptor 2L5	OR2L5	Receptors
**OR2M7_HUMAN**	Olfactory receptor 2M7	OR2M7	Receptors
**OR2M3_HUMAN**	Olfactory receptor 2M3	OR2M3	Receptors
**O2AK2_HUMAN**	Olfactory receptor 2AK2	OR2AK2	Receptors
**OR2L3_HUMAN**	Olfactory receptor 2L3	OR2L3	Receptors
**O13H1_HUMAN**	Olfactory receptor 13H1	OR13H1	Receptors
**O11H1_HUMAN**	Olfactory receptor 11H1	OR11H1	Receptors
**OR7G3_HUMAN**	Olfactory receptor 7G3	OR7G3	Receptors
**OR2Z1_HUMAN**	Olfactory receptor 2Z1	OR2Z1	Receptors
**OR7D4_HUMAN**	Olfactory receptor 7D4	OR7D4	Receptors
**OR7G2_HUMAN**	Olfactory receptor 7G2	OR7G2	Receptors
**OR7G1_HUMAN**	Olfactory receptor 7G1	OR7G1	Receptors
**OR1M1_HUMAN**	Olfactory receptor 1M1	OR1M1	Receptors
**OR7A2_HUMAN**	Putative olfactory receptor 7A2	OR7A2P	Receptors
**G32P1_HUMAN**	Putative G-protein coupled receptor GPR32P1	GPR32P1	Unclassified
**O10H4_HUMAN**	Olfactory receptor 10H4	OR10H4	Receptors
**O10H5_HUMAN**	Olfactory receptor 10H5	OR10H5	Receptors
**O4F17_HUMAN**	Olfactory receptor 4F17	OR4F17	Receptors
**OR4C5_HUMAN**	Olfactory receptor 4C5	OR4C5	Receptors
**OR4S1_HUMAN**	Olfactory receptor 4S1	OR4S1	Receptors
**OR4M2_HUMAN**	Olfactory receptor 4M2	OR4M2	Receptors
**O4F15_HUMAN**	Olfactory receptor 4F15	OR4F15	Receptors
**OR4F6_HUMAN**	Olfactory receptor 4F6	OR4F6	Receptors
**O5AU1_HUMAN**	Olfactory receptor 5AU1	OR5AU1	Receptors
**O11G2_HUMAN**	Olfactory receptor 11G2	OR11G2	Receptors
**OR4E2_HUMAN**	Olfactory receptor 4E2	OR4E2	Receptors
**O10G2_HUMAN**	Olfactory receptor 10G2	OR10G2	Receptors
**O10G3_HUMAN**	Olfactory receptor 10G3	OR10G3	Receptors
**OR6J1_HUMAN**	Olfactory receptor 6J1	OR6J1	Receptors
**OR4KH_HUMAN**	Olfactory receptor 4K17	OR4K17	Receptors
**O11H6_HUMAN**	Olfactory receptor 11H6	OR11H6	Receptors
**O11H7_HUMAN**	Olfactory receptor 11H7	OR11H7	Receptors
**O11H4_HUMAN**	Olfactory receptor 11H4	OR11H4	Receptors
**OR4M1_HUMAN**	Olfactory receptor 4M1	OR4M1	Receptors
**OR4N2_HUMAN**	Olfactory receptor 4N2	OR4N2	Receptors
**OR4K2_HUMAN**	Olfactory receptor 4K2	OR4K2	Receptors

**OR4K5_HUMAN**	Olfactory receptor 4K5	OR4K5	Receptors
**OR4K1_HUMAN**	Olfactory receptor 4K1	OR4K1	Receptors
**OR4KE_HUMAN**	Olfactory receptor 4K14	OR4K14	Receptors
**O10AD_HUMAN**	Olfactory receptor 10AD1	OR10AD1	Receptors
**OR6C4_HUMAN**	Olfactory receptor 6C4	OR6C4	Receptors
**O2AP1_HUMAN**	Olfactory receptor 2AP1	OR2AP1	Receptors
**O10P1_HUMAN**	Olfactory receptor 10P1	OR10P1	Receptors
**O10A7_HUMAN**	Olfactory receptor 10A7	OR10A7	Receptors
**OR9K2_HUMAN**	Olfactory receptor 9K2	OR9K2	Receptors
**OR4D9_HUMAN**	Olfactory receptor 4D9	OR4D9	Receptors
**OR9Q2_HUMAN**	Olfactory receptor 9Q2	OR9Q2	Receptors
**O52B6_HUMAN**	Olfactory receptor 52B6	OR52B6	Receptors
**O52R1_HUMAN**	Olfactory receptor 52R1	OR52R1	Receptors
**O51D1_HUMAN**	Olfactory receptor 51D1	OR51D1	Receptors
**O5AP2_HUMAN**	Olfactory receptor 5AP2	OR5AP2	Receptors
**O10W1_HUMAN**	Olfactory receptor 10W1	OR10W1	Receptors
**OR5BH_HUMAN**	Olfactory receptor 5B17	OR5B17	Receptors
**OR4B1_HUMAN**	Olfactory receptor 4B1	OR4B1	Receptors
**OR4X2_HUMAN**	Olfactory receptor 4X2	OR4X2	Receptors
**OR8J3_HUMAN**	Olfactory receptor 8J3	OR8J3	Receptors
**OR8J2_HUMAN**	Olfactory receptor 8J2	OR8J2	Receptors
**OR5T2_HUMAN**	Olfactory receptor 5T2	OR5T2	Receptors
**OR5T3_HUMAN**	Olfactory receptor 5T3	OR5T3	Receptors
**OR8H1_HUMAN**	Olfactory receptor 8H1	OR8H1	Receptors
**OR8K1_HUMAN**	Olfactory receptor 8K1	OR8K1	Receptors
**OR8BC_HUMAN**	Olfactory receptor 8B12	OR8B12	Receptors
**OR8A1_HUMAN**	Olfactory receptor 8A1	OR8A1	Receptors
**OR8B3_HUMAN**	Olfactory receptor 8B3	OR8B3	Receptors
**OR2D3_HUMAN**	Olfactory receptor 2D3	OR2D3	Receptors
**O56A1_HUMAN**	Olfactory receptor 56A1	OR56A1	Receptors
**O52L2_HUMAN**	Putative olfactory receptor 52L2	OR52L2P	Receptors
**O52L1_HUMAN**	Olfactory receptor 52L1	OR52L1	Receptors
**O56A4_HUMAN**	Olfactory receptor 56A4	OR56A4	Receptors
**O52E4_HUMAN**	Olfactory receptor 52E4	OR52E4	Receptors
**O52N2_HUMAN**	Olfactory receptor 52N2	OR52N2	Receptors
**O56B2_HUMAN**	Putative olfactory receptor 56B2	OR56B2P	Receptors
**O52N4_HUMAN**	Olfactory receptor 52N4	OR52N4	Receptors
**O56B1_HUMAN**	Olfactory receptor 56B1	OR56B1	Receptors
**OR4DB_HUMAN**	Olfactory receptor 4D11	OR4D11	Receptors
**OR4DA_HUMAN**	Olfactory receptor 4D10	OR4D10	Receptors

**O10V1_HUMAN**	Olfactory receptor 10V1	OR10V1	Receptors
**O5AN1_HUMAN**	Olfactory receptor 5AN1	OR5AN1	Receptors
**OR5A2_HUMAN**	Olfactory receptor 5A2	OR5A2	Receptors
**OR5A1_HUMAN**	Olfactory receptor 5A1	OR5A1	Receptors
**OR4D6_HUMAN**	Olfactory receptor 4D6	OR4D6	Receptors
**O52H1_HUMAN**	Olfactory receptor 52H1	OR52H1	Receptors
**O52E1_HUMAN**	Olfactory receptor 52E1	OR52E1	Receptors
**O52E2_HUMAN**	Olfactory receptor 52E2	OR52E2	Receptors
**O51L1_HUMAN**	Olfactory receptor 51L1	OR51L1	Receptors
**O51A4_HUMAN**	Olfactory receptor 51A4	OR51A4	Receptors
**O51A2_HUMAN**	Olfactory receptor 51A2	OR51A2	Receptors
**O51S1_HUMAN**	Olfactory receptor 51S1	OR51S1	Receptors
**O51T1_HUMAN**	Olfactory receptor 51T1	OR51T1	Receptors
**O51G2_HUMAN**	Olfactory receptor 51G2	OR51G2	Receptors
**O51G1_HUMAN**	Olfactory receptor 51G1	OR51G1	Receptors
**O52B4_HUMAN**	Olfactory receptor 52B4	OR52B4	Receptors
**O52K2_HUMAN**	Olfactory receptor 52K2	OR52K2	Receptors
**O52K1_HUMAN**	Olfactory receptor 52K1	OR52K1	Receptors
**O52M1_HUMAN**	Olfactory receptor 52M1	OR52M1	Receptors
**O52I1_HUMAN**	Olfactory receptor 52I1	OR52I1	Receptors
**OR5DG_HUMAN**	Olfactory receptor 5D16	OR5D16	Receptors
**OR5L2_HUMAN**	Olfactory receptor 5L2	OR5L2	Receptors
**OR5DI_HUMAN**	Olfactory receptor 5D18	OR5D18	Receptors
**OR5L1_HUMAN**	Olfactory receptor 5L1	OR5L1	Receptors
**OR5DE_HUMAN**	Olfactory receptor 5D14	OR5D14	Receptors
**OR5DD_HUMAN**	Olfactory receptor 5D13	OR5D13	Receptors
**O4A15_HUMAN**	Olfactory receptor 4A15	OR4A15	Receptors
**OR4P4_HUMAN**	Olfactory receptor 4P4	OR4P4	Receptors
**OR4CG_HUMAN**	Olfactory receptor 4C16	OR4C16	Receptors
**OR4CF_HUMAN**	Olfactory receptor 4C15	OR4C15	Receptors
**OR6M1_HUMAN**	Olfactory receptor 6M1	OR6M1	Receptors
**OR8D4_HUMAN**	Olfactory receptor 8D4	OR8D4	Receptors
**OR4D5_HUMAN**	Olfactory receptor 4D5	OR4D5	Receptors
**OR6T1_HUMAN**	Olfactory receptor 6T1	OR6T1	Receptors
**O10S1_HUMAN**	Olfactory receptor 10S1	OR10S1	Receptors
**O10G4_HUMAN**	Olfactory receptor 10G4	OR10G4	Receptors
**O10G9_HUMAN**	Olfactory receptor 10G9	OR10G9	Receptors
**O10G8_HUMAN**	Olfactory receptor 10G8	OR10G8	Receptors
**O10G7_HUMAN**	Olfactory receptor 10G7	OR10G7	Receptors
**O10D4_HUMAN**	Putative olfactory receptor 10D4	OR10D4P	Receptors

**OR4A4_HUMAN**	Putative olfactory receptor 4A4	OR4A4P	Receptors
**OR4CD_HUMAN**	Olfactory receptor 4C13	OR4C13	Receptors
**OR8J1_HUMAN**	Olfactory receptor 8J1	OR8J1	Receptors
**OR5M9_HUMAN**	Olfactory receptor 5M9	OR5M9	Receptors
**OR5M3_HUMAN**	Olfactory receptor 5M3	OR5M3	Receptors
**OR5M8_HUMAN**	Olfactory receptor 5M8	OR5M8	Receptors
**OR5M1_HUMAN**	Olfactory receptor 5M1	OR5M1	Receptors
**O5AR1_HUMAN**	Olfactory receptor 5AR1	OR5AR1	Receptors
**OR9G4_HUMAN**	Olfactory receptor 9G4	OR9G4	Receptors
**OR6Q1_HUMAN**	Olfactory receptor 6Q1	OR6Q1	Receptors
**OR1S2_HUMAN**	Olfactory receptor 1S2	OR1S2	Receptors
**O10Q1_HUMAN**	Olfactory receptor 10Q1	OR10Q1	Receptors
**OR9Q1_HUMAN**	Olfactory receptor 9Q1	OR9Q1	Receptors
**OR9I1_HUMAN**	Olfactory receptor 9I1	OR9I1	Receptors
**O13A1_HUMAN**	Olfactory receptor 13A1	OR13A1	Receptors
**OR1L6_HUMAN**	Olfactory receptor 1L6	OR1L6	Receptors
**OR1K1_HUMAN**	Olfactory receptor 1K1	OR1K1	Receptors
**OR5C1_HUMAN**	Olfactory receptor 5C1	OR5C1	Receptors
**OR1L4_HUMAN**	Olfactory receptor 1L4	OR1L4	Receptors
**OR1B1_HUMAN**	Olfactory receptor 1B1	OR1B1	Receptors
**OR1L8_HUMAN**	Olfactory receptor 1L8	OR1L8	Receptors
**OR1N2_HUMAN**	Olfactory receptor 1N2	OR1N2	Receptors
**OR1N1_HUMAN**	Olfactory receptor 1N1	OR1N1	Receptors
**OR1J4_HUMAN**	Olfactory receptor 1J4	OR1J4	Receptors
**OR1J2_HUMAN**	Olfactory receptor 1J2	OR1J2	Receptors
**OR1J1_HUMAN**	Olfactory receptor 1J1	OR1J1	Receptors
**O13F1_HUMAN**	Olfactory receptor 13F1	OR13F1	Receptors
**O13C4_HUMAN**	Olfactory receptor 13C4	OR13C4	Receptors
**O13C3_HUMAN**	Olfactory receptor 13C3	OR13C3	Receptors
**O13C8_HUMAN**	Olfactory receptor 13C8	OR13C8	Receptors
**O13C5_HUMAN**	Olfactory receptor 13C5	OR13C5	Receptors
**O13C2_HUMAN**	Olfactory receptor 13C2	OR13C2	Receptors
**O13C9_HUMAN**	Olfactory receptor 13C9	OR13C9	Receptors
**OR2K2_HUMAN**	Olfactory receptor 2K2	OR2K2	Receptors
**O13J1_HUMAN**	Olfactory receptor 13J1	OR13J1	Receptors
**OR9A2_HUMAN**	Olfactory receptor 9A2	OR9A2	Receptors
**O2A12_HUMAN**	Olfactory receptor 2A12	OR2A12	Receptors
**OR2A1_HUMAN**	Olfactory receptor 2A1/2A42	OR2A1	Receptors
**OR9A1_HUMAN**	Putative olfactory receptor 9A1	OR9A1P	Receptors
**OR9A4_HUMAN**	Olfactory receptor 9A4	OR9A4	Receptors

**OR2I1_HUMAN**	Putative olfactory receptor 2I1	OR2I1P	Receptors
**GP150_HUMAN**	Probable G-protein coupled receptor 150	GPR150	Receptors
**OR2Y1_HUMAN**	Olfactory receptor 2Y1	OR2Y1	Receptors
**O13D1_HUMAN**	Olfactory receptor 13D1	OR13D1	Receptors
**OR5H6_HUMAN**	Olfactory receptor 5H6	OR5H6	Receptors
**OR5H2_HUMAN**	Olfactory receptor 5H2	OR5H2	Receptors
**OR6B3_HUMAN**	Olfactory receptor 6B3	OR6B3	Receptors
**OR6K6_HUMAN**	Olfactory receptor 6K6	OR6K6	Receptors
**O11L1_HUMAN**	Olfactory receptor 11L1	OR11L1	Receptors
**O2T34_HUMAN**	Olfactory receptor 2T34	OR2T34	Receptors
**O2T35_HUMAN**	Olfactory receptor 2T35	OR2T35	Receptors
**O10T2_HUMAN**	Olfactory receptor 10T2	OR10T2	Receptors
**O10K1_HUMAN**	Olfactory receptor 10K1	OR10K1	Receptors
**O10R2_HUMAN**	Olfactory receptor 10R2	OR10R2	Receptors
**OR6Y1_HUMAN**	Olfactory receptor 6Y1	OR6Y1	Receptors
**OR6P1_HUMAN**	Olfactory receptor 6P1	OR6P1	Receptors
**O10X1_HUMAN**	Olfactory receptor 10X1	OR10X1	Receptors
**O10Z1_HUMAN**	Olfactory receptor 10Z1	OR10Z1	Receptors
**OR6K2_HUMAN**	Olfactory receptor 6K2	OR6K2	Receptors
**OR6K3_HUMAN**	Olfactory receptor 6K3	OR6K3	Receptors
**OR6N1_HUMAN**	Olfactory receptor 6N1	OR6N1	Receptors
**OR6N2_HUMAN**	Olfactory receptor 6N2	OR6N2	Receptors
**O10J6_HUMAN**	Putative olfactory receptor 10J6	OR10J6P	Receptors
**OR2L8_HUMAN**	Olfactory receptor 2L8	OR2L8	Receptors
**O2AJ1_HUMAN**	Olfactory receptor 2AJ1	OR2AJ1	Receptors
**O14K1_HUMAN**	Olfactory receptor 14K1	OR14K1	Receptors
**O13G1_HUMAN**	Olfactory receptor 13G1	OR13G1	Receptors
**OR2G3_HUMAN**	Olfactory receptor 2G3	OR2G3	Receptors
**OR2G2_HUMAN**	Olfactory receptor 2G2	OR2G2	Receptors
**OR6F1_HUMAN**	Olfactory receptor 6F1	OR6F1	Receptors
**O2T10_HUMAN**	Olfactory receptor 2T10	OR2T10	Receptors
**OR2T4_HUMAN**	Olfactory receptor 2T4	OR2T4	Receptors
**O2T11_HUMAN**	Olfactory receptor 2T11	OR2T11	Receptors
**O2T29_HUMAN**	Olfactory receptor 2T29	OR2T29	Receptors
**OR2T3_HUMAN**	Olfactory receptor 2T3	OR2T3	Receptors
**O2T27_HUMAN**	Olfactory receptor 2T27	OR2T27	Receptors
**OR4Q3_HUMAN**	Olfactory receptor 4Q3	OR4Q3	Receptors
**OR1P1_HUMAN**	Olfactory receptor 1P1	OR1P1	Receptors
**O11H2_HUMAN**	Olfactory receptor 11H2	OR11H2	Receptors
**O10AC_HUMAN**	Putative olfactory receptor 10AC1	OR10AC1P	

**OR8S1_HUMAN**	Olfactory receptor 8S1	OR8S1	Receptors
**OR8U1_HUMAN**	Olfactory receptor 8U1	OR8U1	Receptors
**OR2L2_HUMAN**	Olfactory receptor 2L2	OR2L2	Receptors
**OR5J2_HUMAN**	Olfactory receptor 5J2	OR5J2	Receptors
**O10AG_HUMAN**	Olfactory receptor 10AG1	OR10AG1	Receptors
**OR4F5_HUMAN**	Olfactory receptor 4F5	OR4F5	Receptors
**OR4C3_HUMAN**	Olfactory receptor 4C3	OR4C3	Receptors
**OR6S1_HUMAN**	Olfactory receptor 6S1	OR6S1	Receptors
**OR4KF_HUMAN**	Olfactory receptor 4K15	OR4K15	Receptors
**OR4KD_HUMAN**	Olfactory receptor 4K13	OR4K13	Receptors
**OR4L1_HUMAN**	Olfactory receptor 4L1	OR4L1	Receptors
**OR5B3_HUMAN**	Olfactory receptor 5B3	OR5B3	Receptors
**OR4X1_HUMAN**	Olfactory receptor 4X1	OR4X1	Receptors
**OR8K5_HUMAN**	Olfactory receptor 8K5	OR8K5	Receptors
**OR8K3_HUMAN**	Olfactory receptor 8K3	OR8K3	Receptors
**O52N1_HUMAN**	Olfactory receptor 52N1	OR52N1	Receptors
**O56A3_HUMAN**	Olfactory receptor 56A3	OR56A3	Receptors
**O52E5_HUMAN**	Olfactory receptor 52E5	OR52E5	Receptors
**O52N5_HUMAN**	Olfactory receptor 52N5	OR52N5	Receptors
**O52P1_HUMAN**	Putative olfactory receptor 52P1	OR52P1P	Receptors
**O51Q1_HUMAN**	Olfactory receptor 51Q1	OR51Q1	Receptors
**O52J3_HUMAN**	Olfactory receptor 52J3	OR52J3	Receptors
**O51F2_HUMAN**	Olfactory receptor 51F2	OR51F2	Receptors
**O51H1_HUMAN**	Putative olfactory receptor 51H1	OR51H1P	Receptors
**O51A7_HUMAN**	Olfactory receptor 51A7	OR51A7	Receptors
**O52I2_HUMAN**	Olfactory receptor 52I2	OR52I2	Receptors
**OR5W2_HUMAN**	Olfactory receptor 5W2	OR5W2	Receptors
**O4A16_HUMAN**	Olfactory receptor 4A16	OR4A16	Receptors
**OR4C6_HUMAN**	Olfactory receptor 4C6	OR4C6	Receptors
**OR4S2_HUMAN**	Olfactory receptor 4S2	OR4S2	Receptors
**O10A6_HUMAN**	Olfactory receptor 10A6	OR10A6	Receptors
**O56B4_HUMAN**	Olfactory receptor 56B4	OR56B4	Receptors
**OR6X1_HUMAN**	Olfactory receptor 6X1	OR6X1	Receptors
**O10D3_HUMAN**	Putative olfactory receptor 10D3	OR10D3	Receptors
**O10G6_HUMAN**	Olfactory receptor 10G6	OR10G6	Receptors
**OR4A5_HUMAN**	Olfactory receptor 4A5	OR4A5	Receptors
**OR5R1_HUMAN**	Olfactory receptor 5R1	OR5R1	Receptors
**OR9G1_HUMAN**	Olfactory receptor 9G1	OR9G1	Receptors
**O5AK3_HUMAN**	Putative olfactory receptor 5AK3	OR5AK3P	Unclassified
**O5AK2_HUMAN**	Olfactory receptor 5AK2	OR5AK2	Receptors

**OR1S1_HUMAN**	Olfactory receptor 1S1	OR1S1	Receptors
**OR1L3_HUMAN**	Olfactory receptor 1L3	OR1L3	Receptors
**OR1L1_HUMAN**	Olfactory receptor 1L1	OR1L1	Receptors
**O13C6_HUMAN**	Putative olfactory receptor 13C6	OR13C6P	Receptors
**O2AE1_HUMAN**	Olfactory receptor 2AE1	OR2AE1	Receptors
**OR2W6_HUMAN**	Putative olfactory receptor 2W6	OR2W6P	Receptors
**OR1FC_HUMAN**	Olfactory receptor 1F12	OR1F12	Receptors
**OR2V1_HUMAN**	Olfactory receptor 2V1	OR2V1	Receptors
**OR5K1_HUMAN**	Olfactory receptor 5K1	OR5K1	Receptors
**OR5K2_HUMAN**	Olfactory receptor 5K2	OR5K2	Receptors
**O10J5_HUMAN**	Olfactory receptor 10J5	OR10J5	Receptors
**O14AG_HUMAN**	Olfactory receptor 14A16	OR14A16	Receptors
**O14L1_HUMAN**	Putative olfactory receptor 14L1	OR14L1P	Receptors
**O14CZ_HUMAN**	Olfactory receptor 14C36	OR14C36	Receptors
**OR2T6_HUMAN**	Olfactory receptor 2T6	OR2T6	Receptors
**KI2LB_HUMAN**	Killer cell immunoglobulin-like receptor 2DL5B	KIR2DL5B	Receptors
**LIRB1_HUMAN**	Leukocyte immunoglobulin-like receptor subfamily B member 1	LILRB1	Receptors
**MFSD8_HUMAN**	Major facilitator superfamily domain-containing protein 8	MFSD8	Transporters
**CP052_HUMAN**	Uncharacterized protein C16orf52	C16orf52	Unclassified
**IL31R_HUMAN**	Interleukin-31 receptor subunit alpha	IL31RA	Receptors
**LPD6B_HUMAN**	Ly6/PLAUR domain-containing protein 6B	LYPD6B	Unclassified
**PCD19_HUMAN**	Protocadherin-19	PCDH19	Miscellaneous
**TMHS_HUMAN**	Tetraspan membrane protein of hair cell stereocilia	LHFPL5	Miscellaneous
**TIP_HUMAN**	T-cell immunomodulatory protein	ITFG1	Unclassified
**S7A14_HUMAN**	Probable cationic amino acid transporter	SLC7A14	Transporters
**FNDC9_HUMAN**	Fibronectin type III domain-containing protein 9	FNDC9	Unclassified
**SYNPR_HUMAN**	Synaptoporin	SYNPR	Miscellaneous
**LPPR1_HUMAN**	Lipid phosphate phosphatase-related protein type 1	LPPR1	Enzymes
**F174A_HUMAN**	Membrane protein FAM174A	FAM174A	Unclassified
**ADA32_HUMAN**	Disintegrin and metalloproteinase domain-containing protein 32	ADAM32	Enzymes
**O51E1_HUMAN**	Olfactory receptor 51E1	OR51E1	Receptors
**S22A8_HUMAN**	Solute carrier family 22 member 8	SLC22A8	Transporters
**STT3B_HUMAN**	Dolichyl-diphosphooligosaccharide--protein glycosyltransferase subunit STT3B	STT3B	Enzymes
**SPP2B_HUMAN**	Signal peptide peptidase-like 2B	SPPL2B	Unclassified
**SPP2A_HUMAN**	Signal peptide peptidase-like 2A	SPPL2A	Unclassified
**HM13_HUMAN**	Minor histocompatibility antigen H13	HM13	Unclassified
**NMD3A_HUMAN**	Glutamate receptor ionotropic, NMDA 3A	GRIN3A	Transporters
**ZPLD1_HUMAN**	Zona pellucida-like domain-containing protein 1	ZPLD1	Unclassified
**PKR1_HUMAN**	Prokineticin receptor 1	PROKR1	Receptors
**N2DL4_HUMAN**	NKG2D ligand 4	RAET1E	Miscellaneous

**GTR12_HUMAN**	Solute carrier family 2, facilitated glucose transporter member 12	SLC2A12	Transporters
**MO2R1_HUMAN**	Cell surface glycoprotein CD200 receptor 1	CD200R1	Receptors
**DSCL1_HUMAN**	Down syndrome cell adhesion molecule-like protein 1	DSCAML1	Miscellaneous
**GTR14_HUMAN**	Solute carrier family 2, facilitated glucose transporter member 14	SLC2A14	Transporters
**NETO1_HUMAN**	Neuropilin and tolloid-like protein 1	NETO1	Unclassified
**SACA4_HUMAN**	Sperm acrosome membrane-associated protein 4	SPACA4	
**KCNG4_HUMAN**	Potassium voltage-gated channel subfamily G member 4	KCNG4	Transporters
**KCNV2_HUMAN**	Potassium voltage-gated channel subfamily V member 2	KCNV2	Transporters
**HAVR2_HUMAN**	Hepatitis A virus cellular receptor 2	HAVCR2	Unclassified
**CLM1_HUMAN**	CMRF35-like molecule 1	CD300LF	Receptors
**HCAR2_HUMAN**	Hydroxycarboxylic acid receptor 2	HCAR2	Receptors
**OXER1_HUMAN**	Oxoeicosanoid receptor 1	OXER1	Receptors
**MRGRD_HUMAN**	Mas-related G-protein coupled receptor member D	MRGPRD	Receptors
**GP152_HUMAN**	Probable G-protein coupled receptor 152	GPR152	Receptors
**GPBAR_HUMAN**	G-protein coupled bile acid receptor 1	GPBAR1	Receptors
**RL3R2_HUMAN**	Relaxin-3 receptor 2	RXFP4	Receptors
**GP151_HUMAN**	Probable G-protein coupled receptor 151	GPR151	Receptors
**GP148_HUMAN**	Probable G-protein coupled receptor 148	GPR148	Receptors
**GP119_HUMAN**	Glucose-dependent insulinotropic receptor	GPR119	Receptors
**FAT3_HUMAN**	Protocadherin Fat 3	FAT3	Miscellaneous
**PK1L1_HUMAN**	Polycystic kidney disease protein 1-like 1	PKD1L1	Transporters
**IGDC4_HUMAN**	Immunoglobulin superfamily DCC subclass member 4	IGDCC4	Receptors
**TS1R2_HUMAN**	Taste receptor type 1 member 2	TAS1R2	Receptors
**RN128_HUMAN**	E3 ubiquitin-protein ligase RNF128	RNF128	Unclassified
**SPX2_HUMAN**	Sugar phosphate exchanger 2	SLC37A2	Transporters
**PO210_HUMAN**	Nuclear pore membrane glycoprotein 210	NUP210	Unclassified
**PIGO_HUMAN**	GPI ethanolamine phosphate transferase 3	PIGO	Unclassified
**LRC15_HUMAN**	Leucine-rich repeat-containing protein 15	LRRC15	Miscellaneous
**GDPD5_HUMAN**	Glycerophosphodiester phosphodiesterase domain-containing protein 5	GDPD5	Unclassified
**SCRB1_HUMAN**	Scavenger receptor class B member 1	SCARB1	Receptors
**CHPT1_HUMAN**	Cholinephosphotransferase 1	CHPT1	Enzymes
**S22AH_HUMAN**	Solute carrier family 22 member 17	SLC22A17	Transporters
**LRRN4_HUMAN**	Leucine-rich repeat neuronal protein 4	LRRN4	Miscellaneous
**S38A5_HUMAN**	Sodium-coupled neutral amino acid transporter 5	SLC38A5	Transporters
**T255B_HUMAN**	Transmembrane protein 255B	TMEM255B	Unclassified
**TM171_HUMAN**	Transmembrane protein 171	TMEM171	Unclassified
**SCTM1_HUMAN**	Secreted and transmembrane protein 1	SECTM1	Unclassified
**LMBR1_HUMAN**	Limb region 1 protein homolog	LMBR1	Unclassified
**BT2A2_HUMAN**	Butyrophilin subfamily 2 member A2	BTN2A2	Miscellaneous
**ITLN1_HUMAN**	Intelectin-1	ITLN1	

**NCUG1_HUMAN**	Lysosomal protein NCU-G1	C1orf85	Unclassified
**ZNRF4_HUMAN**	Zinc/RING finger protein 4	ZNRF4	Unclassified
**NRG4_HUMAN**	Pro-neuregulin-4, membrane-bound isoform	NRG4	Unclassified
**CTL1_HUMAN**	Choline transporter-like protein 1	SLC44A1	Transporters
**STAB2_HUMAN**	Stabilin-2	STAB2	Receptors
**S13A3_HUMAN**	Solute carrier family 13 member 3	SLC13A3	Transporters
**FCAMR_HUMAN**	High affinity immunoglobulin alpha and immunoglobulin mu Fc receptor	FCAMR	Receptors
**SC5AB_HUMAN**	Sodium/myo-inositol cotransporter 2	SLC5A11	Transporters
**ABCA5_HUMAN**	ATP-binding cassette sub-family A member 5	ABCA5	Transporters
**5HT3C_HUMAN**	5-hydroxytryptamine receptor 3C	HTR3C	Transporters
**RXFP2_HUMAN**	Relaxin receptor 2	RXFP2	Receptors
**GPR98_HUMAN**	G-protein coupled receptor 98	GPR98	Receptors
**MUC16_HUMAN**	Mucin-16	MUC16	Unclassified
**CCGL_HUMAN**	Voltage-dependent calcium channel gamma-like subunit	TMEM37	Transporters
**CCG8_HUMAN**	Voltage-dependent calcium channel gamma-8 subunit	CACNG8	Transporters
**CTR3_HUMAN**	Cationic amino acid transporter 3	SLC7A3	Transporters
**SORC1_HUMAN**	VPS10 domain-containing receptor SorCS1	SORCS1	Receptors
**CNTP5_HUMAN**	Contactin-associated protein-like 5	CNTNAP5	Receptors
**TM158_HUMAN**	Transmembrane protein 158	TMEM158	Unclassified
**OR8D1_HUMAN**	Olfactory receptor 8D1	OR8D1	Receptors
**OR5P2_HUMAN**	Olfactory receptor 5P2	OR5P2	Receptors
**OR5P3_HUMAN**	Olfactory receptor 5P3	OR5P3	Receptors
**OR1E3_HUMAN**	Olfactory receptor 1E3	OR1E3	Receptors
**ENK8_HUMAN**	Endogenous retrovirus group K member 8 Env polyprotein	ERVK-8	Miscellaneous
**EN113_HUMAN**	Endogenous retrovirus group K member 113 Env polyprotein	HERVK_113	Miscellaneous
**PIEZ1_HUMAN**	Piezo-type mechanosensitive ion channel component 1	PIEZO1	Unclassified
**YLAT2_HUMAN**	Y+L amino acid transporter 2	SLC7A6	Transporters
**K0247_HUMAN**	Uncharacterized protein KIAA0247	KIAA0247	Unclassified
**NICA_HUMAN**	Nicastrin	NCSTN	Unclassified
**TM9S4_HUMAN**	Transmembrane 9 superfamily member 4	TM9SF4	Miscellaneous
**SL9A6_HUMAN**	Sodium/hydrogen exchanger 6	SLC9A6	Transporters
**SGCD_HUMAN**	Delta-sarcoglycan	SGCD	Miscellaneous
**LPAR1_HUMAN**	Lysophosphatidic acid receptor 1	LPAR1	Receptors
**FCGRB_HUMAN**	High affinity immunoglobulin gamma Fc receptor IB	FCGR1B	Receptors
**SORL_HUMAN**	Sortilin-related receptor	SORL1	Receptors
**PVRL2_HUMAN**	Nectin-2	PVRL2	Receptors
**PTPRU_HUMAN**	Receptor-type tyrosine-protein phosphatase U	PTPRU	Receptors
**NRCAM_HUMAN**	Neuronal cell adhesion molecule	NRCAM	Miscellaneous
**PCSK5_HUMAN**	Proprotein convertase subtilisin/kexin type 5	PCSK5	Unclassified
**EDA_HUMAN**	Ectodysplasin-A	EDA	Unclassified

**GHSR_HUMAN**	Growth hormone secretagogue receptor type 1	GHSR	Receptors
**SEM4D_HUMAN**	Semaphorin-4D	SEMA4D	Miscellaneous
**NEO1_HUMAN**	Neogenin	NEO1	Receptors
**MRP2_HUMAN**	Canalicular multispecific organic anion transporter 1	ABCC2	Transporters
**SC5A5_HUMAN**	Sodium/iodide cotransporter	SLC5A5	Transporters
**PTPR2_HUMAN**	Receptor-type tyrosine-protein phosphatase N2	PTPRN2	Receptors
**TNR14_HUMAN**	Tumor necrosis factor receptor superfamily member 14	TNFRSF14	Receptors
**SO2A1_HUMAN**	Solute carrier organic anion transporter family member 2A1	SLCO2A1	Transporters
**IGSF2_HUMAN**	Immunoglobulin superfamily member 2	CD101	Receptors
**TNR25_HUMAN**	Tumor necrosis factor receptor superfamily member 25	TNFRSF25	Receptors
**NAR4_HUMAN**	Ecto-ADP-ribosyltransferase 4	ART4	
**1B38_HUMAN**	HLA class I histocompatibility antigen, B-38 alpha chain	HLA-B	Miscellaneous
**HMR1_HUMAN**	Major histocompatibility complex class I-related gene protein	MR1	Unclassified
**1C17_HUMAN**	HLA class I histocompatibility antigen, Cw-17 alpha chain	HLA-C	Miscellaneous
**2B1C_HUMAN**	HLA class II histocompatibility antigen, DRB1-12 beta chain	HLA-DRB1	Miscellaneous
**KISSR_HUMAN**	KiSS-1 receptor	KISS1R	Receptors
**S38A4_HUMAN**	Sodium-coupled neutral amino acid transporter 4	SLC38A4	Transporters
**PIGT_HUMAN**	GPI transamidase component PIG-T	PIGT	Unclassified
**TAAR8_HUMAN**	Trace amine-associated receptor 8	TAAR8	Receptors
**IGSF8_HUMAN**	Immunoglobulin superfamily member 8	IGSF8	Receptors
**MCHR2_HUMAN**	Melanin-concentrating hormone receptor 2	MCHR2	Receptors
**PMEPA_HUMAN**	Protein TMEPAI	PMEPA1	Unclassified
**TR19L_HUMAN**	Tumor necrosis factor receptor superfamily member 19L	RELT	Receptors
**T106A_HUMAN**	Transmembrane protein 106A	TMEM106A	Unclassified
**SLAF9_HUMAN**	SLAM family member 9	SLAMF9	Unclassified
**MRGRF_HUMAN**	Mas-related G-protein coupled receptor member F	MRGPRF	Receptors
**ESAM_HUMAN**	Endothelial cell-selective adhesion molecule	ESAM	Unclassified
**RGMA_HUMAN**	Repulsive guidance molecule A	RGMA	
**SO4A1_HUMAN**	Solute carrier organic anion transporter family member 4A1	SLCO4A1	Transporters
**TMIG2_HUMAN**	Transmembrane and immunoglobulin domain-containing protein 2	TMIGD2	Unclassified
**T4S18_HUMAN**	Transmembrane 4 L6 family member 18	TM4SF18	Miscellaneous
**GP146_HUMAN**	Probable G-protein coupled receptor 146	GPR146	Receptors
**NTNG2_HUMAN**	Netrin-G2	NTNG2	
**HAVR1_HUMAN**	Hepatitis A virus cellular receptor 1	HAVCR1	Unclassified
**SHSA4_HUMAN**	Protein shisa-4	SHISA4	Unclassified
**SLAF6_HUMAN**	SLAM family member 6	SLAMF6	Unclassified
**KLRG1_HUMAN**	Killer cell lectin-like receptor subfamily G member 1	KLRG1	Receptors
**NTCP4_HUMAN**	Sodium/bile acid cotransporter 4	SLC10A4	Transporters
**CK024_HUMAN**	Uncharacterized protein C11orf24	C11orf24	Unclassified
**I17RA_HUMAN**	Interleukin-17 receptor A	IL17RA	Receptors

**DISP1_HUMAN**	Protein dispatched homolog 1	DISP1	Unclassified
**LIGO1_HUMAN**	Leucine-rich repeat and immunoglobulin-like domain-containing nogo receptor-interacting protein 1	LINGO1	Miscellaneous
**P3IP1_HUMAN**	Phosphoinositide-3-kinase-interacting protein 1	PIK3IP1	Unclassified
**S47A1_HUMAN**	Multidrug and toxin extrusion protein 1	SLC47A1	Unclassified
**ASIC4_HUMAN**	Acid-sensing ion channel 4	ASIC4	
**TSN17_HUMAN**	Tetraspanin-17	TSPAN17	Miscellaneous
**P2Y11_HUMAN**	P2Y purinoceptor 11	P2RY11	Receptors
**SREC2_HUMAN**	Scavenger receptor class F member 2	SCARF2	Receptors
**PGCB_HUMAN**	Brevican core protein	BCAN	
**TECT2_HUMAN**	Tectonic-2	TCTN2	
**S41A3_HUMAN**	Solute carrier family 41 member 3	SLC41A3	Transporters
**TIMD4_HUMAN**	T-cell immunoglobulin and mucin domain-containing protein 4	TIMD4	Unclassified
**CA159_HUMAN**	Uncharacterized protein C1orf159	C1orf159	Unclassified
**VSIG2_HUMAN**	V-set and immunoglobulin domain-containing protein 2	VSIG2	Miscellaneous
**TXD15_HUMAN**	Thioredoxin domain-containing protein 15	TXNDC15	Unclassified
**MRP9_HUMAN**	Multidrug resistance-associated protein 9	ABCC12	Transporters
**ABCCB_HUMAN**	ATP-binding cassette sub-family C member 11	ABCC11	Transporters
**KIRR1_HUMAN**	Kin of IRRE-like protein 1	KIRREL	Miscellaneous
**LRIG1_HUMAN**	Leucine-rich repeats and immunoglobulin-like domains protein 1	LRIG1	Miscellaneous
**TMX3_HUMAN**	Protein disulfide-isomerase TMX3	TMX3	Enzymes
**CDHR1_HUMAN**	Cadherin-related family member 1	CDHR1	Miscellaneous
**PCD16_HUMAN**	Protocadherin-16	DCHS1	Miscellaneous
**S41A2_HUMAN**	Solute carrier family 41 member 2	SLC41A2	Transporters
**TM87B_HUMAN**	Transmembrane protein 87B	TMEM87B	Unclassified
**GP128_HUMAN**	Probable G-protein coupled receptor 128	GPR128	Receptors
**MEG10_HUMAN**	Multiple epidermal growth factor-like domains protein 10	MEGF10	Receptors
**MSLNL_HUMAN**	Mesothelin-like protein	MSLNL	
**KCNS1_HUMAN**	Potassium voltage-gated channel subfamily S member 1	KCNS1	Transporters
**O10C1_HUMAN**	Olfactory receptor 10C1	OR10C1	Receptors
**BT2A3_HUMAN**	Putative butyrophilin subfamily 2 member A3	BTN2A3P	Miscellaneous
**SUSD3_HUMAN**	Sushi domain-containing protein 3	SUSD3	Unclassified
**FCRL2_HUMAN**	Fc receptor-like protein 2	FCRL2	Receptors
**FCRL1_HUMAN**	Fc receptor-like protein 1	FCRL1	Receptors
**MRGX4_HUMAN**	Mas-related G-protein coupled receptor member X4	MRGPRX4	Receptors
**MRGX3_HUMAN**	Mas-related G-protein coupled receptor member X3	MRGPRX3	Receptors
**MRGX2_HUMAN**	Mas-related G-protein coupled receptor member X2	MRGPRX2	Receptors
**MRGX1_HUMAN**	Mas-related G-protein coupled receptor member X1	MRGPRX1	Receptors
**SIG10_HUMAN**	Sialic acid-binding Ig-like lectin 10	SIGLEC10	Miscellaneous
**SGCZ_HUMAN**	Zeta-sarcoglycan	SGCZ	Miscellaneous
**HIAT1_HUMAN**	Hippocampus abundant transcript 1 protein	HIAT1	Transporters

**ROBO3_HUMAN**	Roundabout homolog 3	ROBO3	Receptors
**KREM1_HUMAN**	Kremen protein 1	KREMEN1	Unclassified
**G137A_HUMAN**	Integral membrane protein GPR137	GPR137	Unclassified
**S6A18_HUMAN**	Sodium-dependent neutral amino acid transporter B(0)AT3	SLC6A18	Transporters
**LRFN5_HUMAN**	Leucine-rich repeat and fibronectin type-III domain-containing protein 5	LRFN5	Miscellaneous
**PTHD1_HUMAN**	Patched domain-containing protein 1	PTCHD1	Unclassified
**PCFT_HUMAN**	Proton-coupled folate transporter	SLC46A1	Transporters
**CNT3B_HUMAN**	Contactin-associated protein-like 3B	CNTNAP3B	Receptors
**PVRL4_HUMAN**	Nectin-4	PVRL4	Receptors
**FCRL3_HUMAN**	Fc receptor-like protein 3	FCRL3	Receptors
**QRFPR_HUMAN**	Pyroglutamylated RFamide peptide receptor	QRFPR	Receptors
**GP101_HUMAN**	Probable G-protein coupled receptor 101	GPR101	Receptors
**GPR82_HUMAN**	Probable G-protein coupled receptor 82	GPR82	Receptors
**OXGR1_HUMAN**	2-oxoglutarate receptor 1	OXGR1	Receptors
**GPR78_HUMAN**	G-protein coupled receptor 78	GPR78	Receptors
**GNRR2_HUMAN**	Putative gonadotropin-releasing hormone II receptor	GNRHR2	Receptors
**CASD1_HUMAN**	CAS1 domain-containing protein 1	CASD1	Unclassified
**LRC3B_HUMAN**	Leucine-rich repeat-containing protein 3B	LRRC3B	Miscellaneous
**DCBD2_HUMAN**	Discoidin, CUB and LCCL domain-containing protein 2	DCBLD2	Unclassified
**GP124_HUMAN**	G-protein coupled receptor 124	GPR124	Receptors
**OPALI_HUMAN**	Opalin	OPALIN	Unclassified
**FCRL4_HUMAN**	Fc receptor-like protein 4	FCRL4	Unclassified
**TECTB_HUMAN**	Beta-tectorin	TECTB	
**ERMAP_HUMAN**	Erythroid membrane-associated protein	ERMAP	Miscellaneous
**SORC2_HUMAN**	VPS10 domain-containing receptor SorCS2	SORCS2	Receptors
**SIG12_HUMAN**	Sialic acid-binding Ig-like lectin 12	SIGLEC12	Miscellaneous
**AQP10_HUMAN**	Aquaporin-10	AQP10	Transporters
**SLIK1_HUMAN**	SLIT and NTRK-like protein 1	SLITRK1	Miscellaneous
**CSMD1_HUMAN**	CUB and sushi domain-containing protein 1	CSMD1	Miscellaneous
**S38A2_HUMAN**	Sodium-coupled neutral amino acid transporter 2	SLC38A2	Transporters
**MYCT_HUMAN**	Proton myo-inositol cotransporter	SLC2A13	Transporters
**LR37B_HUMAN**	Leucine-rich repeat-containing protein 37B	LRRC37B	Miscellaneous
**PCD15_HUMAN**	Protocadherin-15	PCDH15	Miscellaneous
**OR5BC_HUMAN**	Olfactory receptor 5B12	OR5B12	Receptors
**OR5B2_HUMAN**	Olfactory receptor 5B2	OR5B2	Receptors
**OR2M4_HUMAN**	Olfactory receptor 2M4	OR2M4	Receptors
**OR2M2_HUMAN**	Olfactory receptor 2M2	OR2M2	Receptors
**OR2V2_HUMAN**	Olfactory receptor 2V2	OR2V2	Receptors
**OR2A7_HUMAN**	Olfactory receptor 2A7	OR2A7	Receptors
**O2A14_HUMAN**	Olfactory receptor 2A14	OR2A14	Receptors

**OR2A5_HUMAN**	Olfactory receptor 2A5	OR2A5	Receptors
**O14A2_HUMAN**	Olfactory receptor 14A2	OR14A2	Receptors
**OR4CC_HUMAN**	Olfactory receptor 4C12	OR4C12	Receptors
**OR4F4_HUMAN**	Olfactory receptor 4F4	OR4F4	Receptors
**OR4K3_HUMAN**	Olfactory receptor 4K3	OR4K3	Receptors
**OR1F2_HUMAN**	Putative olfactory receptor 1F2	OR1F2P	Receptors
**OR7D2_HUMAN**	Olfactory receptor 7D2	OR7D2	Receptors
**OR5MB_HUMAN**	Olfactory receptor 5M11	OR5M11	Receptors
**OR8B4_HUMAN**	Olfactory receptor 8B4	OR8B4	Receptors
**OR8B2_HUMAN**	Olfactory receptor 8B2	OR8B2	Receptors
**OR6C1_HUMAN**	Olfactory receptor 6C1	OR6C1	Receptors
**O52B2_HUMAN**	Olfactory receptor 52B2	OR52B2	Receptors
**O52E6_HUMAN**	Olfactory receptor 52E6	OR52E6	Receptors
**PANX2_HUMAN**	Pannexin-2	PANX2	Unclassified
**PANX1_HUMAN**	Pannexin-1	PANX1	Unclassified
**FCRL5_HUMAN**	Fc receptor-like protein 5	FCRL5	Unclassified
**PAR4_HUMAN**	Proteinase-activated receptor 4	F2RL3	Receptors
**TAAR6_HUMAN**	Trace amine-associated receptor 6	TAAR6	Receptors
**TAAR9_HUMAN**	Trace amine-associated receptor 9	TAAR9	Receptors
**TAAR1_HUMAN**	Trace amine-associated receptor 1	TAAR1	Receptors
**SIG11_HUMAN**	Sialic acid-binding Ig-like lectin 11	SIGLEC11	Miscellaneous
**S26A8_HUMAN**	Testis anion transporter 1	SLC26A8	Transporters
**PCX1_HUMAN**	Pecanex-like protein 1	PCNX	Unclassified
**S22AC_HUMAN**	Solute carrier family 22 member 12	SLC22A12	Transporters
**MYADM_HUMAN**	Myeloid-associated differentiation marker	MYADM	Miscellaneous
**SERC2_HUMAN**	Serine incorporator 2	SERINC2	Transporters
**TSN18_HUMAN**	Tetraspanin-18	TSPAN18	Miscellaneous
**DIRC2_HUMAN**	Disrupted in renal carcinoma protein 2	DIRC2	Unclassified
**SL9A7_HUMAN**	Sodium/hydrogen exchanger 7	SLC9A7	Transporters
**CSF3R_HUMAN**	Granulocyte colony-stimulating factor receptor	CSF3R	Receptors
**HBEGF_HUMAN**	Proheparin-binding EGF-like growth factor	HBEGF	Unclassified
**MUC4_HUMAN**	Mucin-4	MUC4	Unclassified
**SCN2A_HUMAN**	Sodium channel protein type 2 subunit alpha	SCN2A	Transporters
**GML_HUMAN**	Glycosyl-phosphatidylinositol-anchored molecule-like protein	GML	
**NPY6R_HUMAN**	Putative neuropeptide Y receptor type 6	NPY6R	Receptors
**NOTC4_HUMAN**	Neurogenic locus notch homolog protein 4	NOTCH4	Receptors
**CD180_HUMAN**	CD180 antigen	CD180	Receptors
**S1PR3_HUMAN**	Sphingosine 1-phosphate receptor 3	S1PR3	Receptors
**SORT_HUMAN**	Sortilin	SORT1	Receptors
**GPER1_HUMAN**	G-protein coupled estrogen receptor 1	GPER1	Receptors

**P2RX4_HUMAN**	P2X purinoceptor 4	P2RX4	Transporters
**P2RX7_HUMAN**	P2X purinoceptor 7	P2RX7	Transporters
**OSMR_HUMAN**	Oncostatin-M-specific receptor subunit beta	OSMR	Receptors
**I12R2_HUMAN**	Interleukin-12 receptor subunit beta-2	IL12RB2	Receptors
**LPAR4_HUMAN**	Lysophosphatidic acid receptor 4	LPAR4	Receptors
**GPR20_HUMAN**	G-protein coupled receptor 20	GPR20	Receptors
**GPR21_HUMAN**	Probable G-protein coupled receptor 21	GPR21	Receptors
**GPR22_HUMAN**	Probable G-protein coupled receptor 22	GPR22	Receptors
**MCHR1_HUMAN**	Melanin-concentrating hormone receptor 1	MCHR1	Receptors
**KI2L4_HUMAN**	Killer cell immunoglobulin-like receptor 2DL4	KIR2DL4	Receptors
**ABCA3_HUMAN**	ATP-binding cassette sub-family A member 3	ABCA3	Transporters
**CML1_HUMAN**	Chemokine-like receptor 1	CMKLR1	Receptors
**GPA33_HUMAN**	Cell surface A33 antigen	GPA33	Receptors
**TM9S2_HUMAN**	Transmembrane 9 superfamily member 2	TM9SF2	Miscellaneous
**S29A1_HUMAN**	Equilibrative nucleoside transporter 1	SLC29A1	Transporters
**SMO_HUMAN**	Smoothened homolog	SMO	Receptors
**SC6A7_HUMAN**	Sodium-dependent proline transporter	SLC6A7	Transporters
**GBRG3_HUMAN**	Gamma-aminobutyric acid receptor subunit gamma-3	GABRG3	Transporters
**ADAM2_HUMAN**	Disintegrin and metalloproteinase domain-containing protein 2	ADAM2	Enzymes
**P2Y13_HUMAN**	P2Y purinoceptor 13	P2RY13	Receptors
**PD1L2_HUMAN**	Programmed cell death 1 ligand 2	PDCD1LG2	Unclassified
**HEPH_HUMAN**	Hephaestin	HEPH	Unclassified
**CSTN3_HUMAN**	Calsyntenin-3	CLSTN3	Miscellaneous
**MXRA8_HUMAN**	Matrix-remodeling-associated protein 8	MXRA8	Unclassified
**S35A5_HUMAN**	Probable UDP-sugar transporter protein SLC35A5	SLC35A5	Transporters
**TTYH2_HUMAN**	Protein tweety homolog 2	TTYH2	Transporters
**TM204_HUMAN**	Transmembrane protein 204	TMEM204	Unclassified
**UPK3B_HUMAN**	Uroplakin-3b	UPK3B	Miscellaneous
**LRFN3_HUMAN**	Leucine-rich repeat and fibronectin type-III domain-containing protein 3	LRFN3	Miscellaneous
**CRUM3_HUMAN**	Protein crumbs homolog 3	CRB3	Miscellaneous
**LFG2_HUMAN**	Protein lifeguard 2	FAIM2	Unclassified
**BOC_HUMAN**	Brother of CDO	BOC	Receptors
**JAM3_HUMAN**	Junctional adhesion molecule C	JAM3	Unclassified
**PLVAP_HUMAN**	Plasmalemma vesicle-associated protein	PLVAP	Unclassified
**SUCR1_HUMAN**	Succinate receptor 1	SUCNR1	Receptors
**LGR4_HUMAN**	Leucine-rich repeat-containing G-protein coupled receptor 4	LGR4	Receptors
**HCAR1_HUMAN**	Hydroxycarboxylic acid receptor 1	HCAR1	Receptors
**GP174_HUMAN**	Probable G-protein coupled receptor 174	GPR174	Receptors
**VN1R3_HUMAN**	Vomeronasal type-1 receptor 3	VN1R3	Receptors
**AMNLS_HUMAN**	Protein amnionless	AMN	Unclassified

**CLC7A_HUMAN**	C-type lectin domain family 7 member A	CLEC7A	Receptors
**S12A9_HUMAN**	Solute carrier family 12 member 9	SLC12A9	Transporters
**TLR10_HUMAN**	Toll-like receptor 10	TLR10	Receptors
**S26A6_HUMAN**	Solute carrier family 26 member 6	SLC26A6	Transporters
**CCG6_HUMAN**	Voltage-dependent calcium channel gamma-6 subunit	CACNG6	Transporters
**S4A5_HUMAN**	Electrogenic sodium bicarbonate cotransporter 4	SLC4A5	Transporters
**TSCOT_HUMAN**	Thymic stromal cotransporter homolog	SLC46A2	Transporters
**TX101_HUMAN**	Testis-expressed sequence 101 protein	TEX101	
**EMR3_HUMAN**	EGF-like module-containing mucin-like hormone receptor-like 3	EMR3	Receptors
**GPR87_HUMAN**	G-protein coupled receptor 87	GPR87	Receptors
**CADM1_HUMAN**	Cell adhesion molecule 1	CADM1	Miscellaneous
**MFRP_HUMAN**	Membrane frizzled-related protein	MFRP	Unclassified
**TMPSD_HUMAN**	Transmembrane protease serine 13	TMPRSS13	Enzymes
**CDHR2_HUMAN**	Cadherin-related family member 2	CDHR2	Miscellaneous
**ACE2_HUMAN**	Angiotensin-converting enzyme 2	ACE2	Unclassified
**S17A9_HUMAN**	Solute carrier family 17 member 9	SLC17A9	Transporters
**ANO3_HUMAN**	Anoctamin-3	ANO3	Miscellaneous
**GTR11_HUMAN**	Solute carrier family 2, facilitated glucose transporter member 11	SLC2A11	Transporters
**ADA33_HUMAN**	Disintegrin and metalloproteinase domain-containing protein 33	ADAM33	Enzymes
**CNTP3_HUMAN**	Contactin-associated protein-like 3	CNTNAP3	Receptors
**PC11X_HUMAN**	Protocadherin-11 X-linked	PCDH11X	Miscellaneous
**PC11Y_HUMAN**	Protocadherin-11 Y-linked	PCDH11Y	Miscellaneous
**ABCA2_HUMAN**	ATP-binding cassette sub-family A member 2	ABCA2	Transporters
**S29A3_HUMAN**	Equilibrative nucleoside transporter 3	SLC29A3	Transporters
**PPAT_HUMAN**	Testicular acid phosphatase	ACPT	Enzymes
**LYNX1_HUMAN**	Ly-6/neurotoxin-like protein 1	LYNX1	Unclassified
**GPR63_HUMAN**	Probable G-protein coupled receptor 63	GPR63	Receptors
**GPR62_HUMAN**	Probable G-protein coupled receptor 62	GPR62	Receptors
**GPR61_HUMAN**	Probable G-protein coupled receptor 61	GPR61	Receptors
**N2DL3_HUMAN**	NKG2D ligand 3	ULBP3	Miscellaneous
**N2DL2_HUMAN**	NKG2D ligand 2	ULBP2	Miscellaneous
**N2DL1_HUMAN**	NKG2D ligand 1	ULBP1	Miscellaneous
**RTN4R_HUMAN**	Reticulon-4 receptor	RTN4R	
**S19A3_HUMAN**	Thiamine transporter 2	SLC19A3	Transporters
**IMPG2_HUMAN**	Interphotoreceptor matrix proteoglycan 2	IMPG2	Unclassified
**S13A1_HUMAN**	Solute carrier family 13 member 1	SLC13A1	Transporters
**CD244_HUMAN**	Natural killer cell receptor 2B4	CD244	Unclassified
**SN_HUMAN**	Sialoadhesin	SIGLEC1	Unclassified
**CNTP4_HUMAN**	Contactin-associated protein-like 4	CNTNAP4	Receptors
**ZDHC5_HUMAN**	Palmitoyltransferase ZDHHC5	ZDHHC5	Enzymes

**SEM4C_HUMAN**	Semaphorin-4C	SEMA4C	Miscellaneous
**TTYH3_HUMAN**	Protein tweety homolog 3	TTYH3	Transporters
**THS7B_HUMAN**	Thrombospondin type-1 domain-containing protein 7B	THSD7B	Unclassified
**S39A8_HUMAN**	Zinc transporter ZIP8	SLC39A8	Transporters
**2B1E_HUMAN**	HLA class II histocompatibility antigen, DRB1-14 beta chain	HLA-DRB1	Miscellaneous
**OR2B2_HUMAN**	Olfactory receptor 2B2	OR2B2	Receptors
**OR2H1_HUMAN**	Olfactory receptor 2H1	OR2H1	Receptors
**OR2J1_HUMAN**	Olfactory receptor 2J1	OR2J1	Receptors
**O11A1_HUMAN**	Olfactory receptor 11A1	OR11A1	Receptors
**OR8D2_HUMAN**	Olfactory receptor 8D2	OR8D2	Receptors
**GPR88_HUMAN**	Probable G-protein coupled receptor 88	GPR88	Receptors
**S6A16_HUMAN**	Orphan sodium- and chloride-dependent neurotransmitter transporter NTT5	SLC6A16	Transporters
**VN1R1_HUMAN**	Vomeronasal type-1 receptor 1	VN1R1	Receptors
**NMUR2_HUMAN**	Neuromedin-U receptor 2	NMUR2	Receptors
**NPFF1_HUMAN**	Neuropeptide FF receptor 1	NPFFR1	Receptors
**MCLN1_HUMAN**	Mucolipin-1	MCOLN1	Transporters
**SC5A7_HUMAN**	High affinity choline transporter 1	SLC5A7	Transporters
**ACH10_HUMAN**	Neuronal acetylcholine receptor subunit alpha-10	CHRNA10	Transporters
**GFRA4_HUMAN**	GDNF family receptor alpha-4	GFRA4	
**ADA19_HUMAN**	Disintegrin and metalloproteinase domain-containing protein 19	ADAM19	Enzymes
**S22A4_HUMAN**	Solute carrier family 22 member 4	SLC22A4	Transporters
**LMA2L_HUMAN**	VIP36-like protein	LMAN2L	Unclassified
**SLIK2_HUMAN**	SLIT and NTRK-like protein 2	SLITRK2	Miscellaneous
**PCDC1_HUMAN**	Protocadherin alpha-C1	PCDHAC1	
**CAD19_HUMAN**	Cadherin-19	CDH19	Miscellaneous
**ABCG4_HUMAN**	ATP-binding cassette sub-family G member 4	ABCG4	Transporters
**MUC3B_HUMAN**	Mucin-3B (Fragments)	MUC3B	Unclassified
**LPAR5_HUMAN**	Lysophosphatidic acid receptor 5	LPAR5	Receptors
**UN93B_HUMAN**	Protein unc-93 homolog B1	UNC93B1	Unclassified
**TRPV6_HUMAN**	Transient receptor potential cation channel subfamily V member 6	TRPV6	Transporters
**TMX4_HUMAN**	Thioredoxin-related transmembrane protein 4	TMX4	Unclassified
**MEGF9_HUMAN**	Multiple epidermal growth factor-like domains protein 9	MEGF9	Unclassified
**S6A17_HUMAN**	Sodium-dependent neutral amino acid transporter SLC6A17	SLC6A17	Transporters
**OPN3_HUMAN**	Opsin-3	OPN3	Receptors
**O2AG1_HUMAN**	Olfactory receptor 2AG1	OR2AG1	Receptors
**O10A5_HUMAN**	Olfactory receptor 10A5	OR10A5	Receptors
**O10A2_HUMAN**	Olfactory receptor 10A2	OR10A2	Receptors
**O10A4_HUMAN**	Olfactory receptor 10A4	OR10A4	Receptors
**OR2D2_HUMAN**	Olfactory receptor 2D2	OR2D2	Receptors
**ABCG5_HUMAN**	ATP-binding cassette sub-family G member 5	ABCG5	Transporters

**S1PR5_HUMAN**	Sphingosine 1-phosphate receptor 5	S1PR5	Receptors
**P2Y12_HUMAN**	P2Y purinoceptor 12	P2RY12	Receptors
**CAD23_HUMAN**	Cadherin-23	CDH23	Miscellaneous
**O51E2_HUMAN**	Olfactory receptor 51E2	OR51E2	Receptors
**DCSTP_HUMAN**	Dendritic cell-specific transmembrane protein	DCSTAMP	Unclassified
**CXL16_HUMAN**	C-X-C motif chemokine 16	CXCL16	Receptors
**S26A1_HUMAN**	Sulfate anion transporter 1	SLC26A1	Transporters
**O52A5_HUMAN**	Olfactory receptor 52A5	OR52A5	Receptors
**O51V1_HUMAN**	Olfactory receptor 51V1	OR51V1	Receptors
**SEM6A_HUMAN**	Semaphorin-6A	SEMA6A	Miscellaneous
**S38A1_HUMAN**	Sodium-coupled neutral amino acid transporter 1	SLC38A1	Transporters
**S6A15_HUMAN**	Sodium-dependent neutral amino acid transporter B(0)AT2	SLC6A15	Transporters
**ADAM7_HUMAN**	Disintegrin and metalloproteinase domain-containing protein 7	ADAM7	Enzymes
**M4A6A_HUMAN**	Membrane-spanning 4-domains subfamily A member 6A	MS4A6A	Miscellaneous
**CLC4M_HUMAN**	C-type lectin domain family 4 member M	CLEC4M	Receptors
**S12A5_HUMAN**	Solute carrier family 12 member 5	SLC12A5	Transporters
**SO5A1_HUMAN**	Solute carrier organic anion transporter family member 5A1	SLCO5A1	Transporters
**TTYH1_HUMAN**	Protein tweety homolog 1	TTYH1	Transporters
**TM245_HUMAN**	Transmembrane protein 245	TMEM245	Unclassified
**O51B5_HUMAN**	Olfactory receptor 51B5	OR51B5	Receptors
**O51B6_HUMAN**	Olfactory receptor 51B6	OR51B6	Receptors
**O51M1_HUMAN**	Olfactory receptor 51M1	OR51M1	Receptors
**O51J1_HUMAN**	Olfactory receptor 51J1	OR51J1	Receptors
**O51I1_HUMAN**	Olfactory receptor 51I1	OR51I1	Receptors
**O51I2_HUMAN**	Olfactory receptor 51I2	OR51I2	Receptors
**O52D1_HUMAN**	Olfactory receptor 52D1	OR52D1	Receptors
**HRH4_HUMAN**	Histamine H4 receptor	HRH4	Receptors
**MUC13_HUMAN**	Mucin-13	MUC13	Unclassified
**SEM4A_HUMAN**	Semaphorin-4A	SEMA4A	Miscellaneous
**TMPS5_HUMAN**	Transmembrane protease serine 5	TMPRSS5	Enzymes
**SEM6C_HUMAN**	Semaphorin-6C	SEMA6C	Miscellaneous
**SEM6B_HUMAN**	Semaphorin-6B	SEMA6B	Miscellaneous
**LRRN3_HUMAN**	Leucine-rich repeat neuronal protein 3	LRRN3	Miscellaneous
**FZD8_HUMAN**	Frizzled-8	FZD8	Receptors
**DPEP2_HUMAN**	Dipeptidase 2	DPEP2	Enzymes
**DPEP3_HUMAN**	Dipeptidase 3	DPEP3	Enzymes
**CSTN2_HUMAN**	Calsyntenin-2	CLSTN2	Miscellaneous
**VIAAT_HUMAN**	Vesicular inhibitory amino acid transporter	SLC32A1	Transporters
**PIEZ2_HUMAN**	Piezo-type mechanosensitive ion channel component 2	PIEZO2	Unclassified
**CDCP1_HUMAN**	CUB domain-containing protein 1	CDCP1	Unclassified

**SLIK6_HUMAN**	SLIT and NTRK-like protein 6	SLITRK6	Miscellaneous
**IGFR1_HUMAN**	IGF-like family receptor 1	IGFLR1	Unclassified
**CLMP_HUMAN**	CXADR-like membrane protein	CLMP	Miscellaneous
**FNDC4_HUMAN**	Fibronectin type III domain-containing protein 4	FNDC4	Unclassified
**TM231_HUMAN**	Transmembrane protein 231	TMEM231	Unclassified
**ANTR1_HUMAN**	Anthrax toxin receptor 1	ANTXR1	Unclassified
**RN167_HUMAN**	E3 ubiquitin-protein ligase RNF167	RNF167	Unclassified
**LRC19_HUMAN**	Leucine-rich repeat-containing protein 19	LRRC19	Miscellaneous
**AT133_HUMAN**	Probable cation-transporting ATPase 13A3	ATP13A3	Transporters
**GI24_HUMAN**	Platelet receptor Gi24	C10orf54	Unclassified
**NPAL2_HUMAN**	NIPA-like protein 2	NIPAL2	Transporters
**MANS1_HUMAN**	MANSC domain-containing protein 1	MANSC1	Enzymes
**CNNM2_HUMAN**	Metal transporter CNNM2	CNNM2	Unclassified
**ZDH11_HUMAN**	Probable palmitoyltransferase ZDHHC11	ZDHHC11	Enzymes
**MER34_HUMAN**	Endogenous retrovirus group MER34 member 1 Env polyprotein	ERVMER34-1	Miscellaneous
**CHODL_HUMAN**	Chondrolectin	CHODL	Unclassified
**FKRP_HUMAN**	Fukutin-related protein	FKRP	
**CAHM2_HUMAN**	Calcium homeostasis modulator protein 2	CALHM2	Enzymes
**S52A2_HUMAN**	Solute carrier family 52, riboflavin transporter, member 2	SLC52A2	Receptors
**LPHN3_HUMAN**	Latrophilin-3	LPHN3	Receptors
**S28A3_HUMAN**	Solute carrier family 28 member 3	SLC28A3	Transporters
**TNR27_HUMAN**	Tumor necrosis factor receptor superfamily member 27	EDA2R	Receptors
**ILRL2_HUMAN**	Interleukin-1 receptor-like 2	IL1RL2	Receptors
**NMUR1_HUMAN**	Neuromedin-U receptor 1	NMUR1	Receptors
**TRPV4_HUMAN**	Transient receptor potential cation channel subfamily V member 4	TRPV4	Transporters
**CDHR5_HUMAN**	Cadherin-related family member 5	CDHR5	Miscellaneous
**IL21R_HUMAN**	Interleukin-21 receptor	IL21R	Receptors
**LY9_HUMAN**	T-lymphocyte surface antigen Ly-9	LY9	Unclassified
**TMM27_HUMAN**	Collectrin	TMEM27	Unclassified
**LRTM1_HUMAN**	Leucine-rich repeat and transmembrane domain-containing protein 1	LRTM1	Miscellaneous
**CAD20_HUMAN**	Cadherin-20	CDH20	Miscellaneous
**SACA1_HUMAN**	Sperm acrosome membrane-associated protein 1	SPACA1	Unclassified
**LPAR2_HUMAN**	Lysophosphatidic acid receptor 2	LPAR2	Receptors
**LRRC4_HUMAN**	Leucine-rich repeat-containing protein 4	LRRC4	Miscellaneous
**ELTD1_HUMAN**	EGF, latrophilin and seven transmembrane domain-containing protein 1	ELTD1	Receptors
**LGR6_HUMAN**	Leucine-rich repeat-containing G-protein coupled receptor 6	LGR6	Receptors
**RXFP1_HUMAN**	Relaxin receptor 1	RXFP1	Receptors
**PCDH9_HUMAN**	Protocadherin-9	PCDH9	Miscellaneous
**NCKX3_HUMAN**	Sodium/potassium/calcium exchanger 3	SLC24A3	Transporters
**CRLF2_HUMAN**	Cytokine receptor-like factor 2	CRLF2	Unclassified

**GPR35_HUMAN**	G-protein coupled receptor 35	GPR35	Receptors
**GDPD2_HUMAN**	Glycerophosphoinositol inositolphosphodiesterase GDPD2	GDPD2	Unclassified
**ANKH_HUMAN**	Progressive ankylosis protein homolog	ANKH	Unclassified
**LRC4C_HUMAN**	Leucine-rich repeat-containing protein 4C	LRRC4C	Miscellaneous
**ROBO2_HUMAN**	Roundabout homolog 2	ROBO2	Receptors
**PCD18_HUMAN**	Protocadherin-18	PCDH18	Miscellaneous
**PLXA4_HUMAN**	Plexin-A4	PLXNA4	Receptors
**TMM8A_HUMAN**	Transmembrane protein 8A	TMEM8A	Unclassified
**GPVI_HUMAN**	Platelet glycoprotein VI	GP6	Unclassified
**CD248_HUMAN**	Endosialin	CD248	Receptors
**CELR2_HUMAN**	Cadherin EGF LAG seven-pass G-type receptor 2	CELSR2	Receptors
**AT131_HUMAN**	Manganese-transporting ATPase 13A1	ATP13A1	Transporters
**PTPRH_HUMAN**	Receptor-type tyrosine-protein phosphatase H	PTPRH	Receptors
**TM9S3_HUMAN**	Transmembrane 9 superfamily member 3	TM9SF3	Miscellaneous
**NRX3B_HUMAN**	Neurexin-3-beta	NRXN3	Receptors
**ENH3_HUMAN**	HERV-H_2q24.1 provirus ancestral Env polyprotein		Miscellaneous
**ENH1_HUMAN**	HERV-H_2q24.3 provirus ancestral Env polyprotein		Miscellaneous
**S40A1_HUMAN**	Solute carrier family 40 member 1	SLC40A1	Transporters
**IRPL2_HUMAN**	X-linked interleukin-1 receptor accessory protein-like 2	IL1RAPL2	Receptors
**ABCB9_HUMAN**	ATP-binding cassette sub-family B member 9	ABCB9	Transporters
**S6A20_HUMAN**	Sodium- and chloride-dependent transporter XTRP3	SLC6A20	Transporters
**S39A2_HUMAN**	Zinc transporter ZIP2	SLC39A2	Transporters
**TREM1_HUMAN**	Triggering receptor expressed on myeloid cells 1	TREM1	Receptors
**KCMB3_HUMAN**	Calcium-activated potassium channel subunit beta-3	KCNMB3	Transporters
**MMP25_HUMAN**	Matrix metalloproteinase-25	MMP25	
**ACKR4_HUMAN**	Atypical chemokine receptor 4	ACKR4	Receptors
**LT4R2_HUMAN**	Leukotriene B4 receptor 2	LTB4R2	Receptors
**SO1B3_HUMAN**	Solute carrier organic anion transporter family member 1B3	SLCO1B3	Transporters
**NRN1_HUMAN**	Neuritin	NRN1	
**CD320_HUMAN**	CD320 antigen	CD320	Unclassified
**FZD3_HUMAN**	Frizzled-3	FZD3	Receptors
**PCD12_HUMAN**	Protocadherin-12	PCDH12	Miscellaneous
**IL1AP_HUMAN**	Interleukin-1 receptor accessory protein	IL1RAP	Receptors
**NOX4_HUMAN**	NADPH oxidase 4	NOX4	Enzymes
**SEM4B_HUMAN**	Semaphorin-4B	SEMA4B	Miscellaneous
**GP108_HUMAN**	Protein GPR108	GPR108	Unclassified
**CN132_HUMAN**	Putative uncharacterized protein C14orf132	C14orf132	Unclassified
**C1QR1_HUMAN**	Complement component C1q receptor	CD93	Unclassified
**AT132_HUMAN**	Probable cation-transporting ATPase 13A2	ATP13A2	Transporters
**SLAF7_HUMAN**	SLAM family member 7	SLAMF7	Unclassified

**TMM9B_HUMAN**	Transmembrane protein 9B	TMEM9B	Unclassified
**S52A3_HUMAN**	Solute carrier family 52, riboflavin transporter, member 3	SLC52A3	Receptors
**EQTN_HUMAN**	Equatorin	EQTN	Unclassified
**GPC5C_HUMAN**	G-protein coupled receptor family C group 5 member C	GPRC5C	Receptors
**ANO2_HUMAN**	Anoctamin-2	ANO2	Miscellaneous
**TRPV5_HUMAN**	Transient receptor potential cation channel subfamily V member 5	TRPV5	Transporters
**OR2S1_HUMAN**	Olfactory receptor 2S2	OR2S2	Receptors
**PVRL3_HUMAN**	Nectin-3	PVRL3	Receptors
**GPR84_HUMAN**	G-protein coupled receptor 84	GPR84	Receptors
**ITM2C_HUMAN**	Integral membrane protein 2C	ITM2C	Miscellaneous
**C163B_HUMAN**	Scavenger receptor cysteine-rich type 1 protein M160	CD163L1	Unclassified
**DLL4_HUMAN**	Delta-like protein 4	DLL4	Miscellaneous
**TLR9_HUMAN**	Toll-like receptor 9	TLR9	Receptors
**TLR8_HUMAN**	Toll-like receptor 8	TLR8	Receptors
**S17A5_HUMAN**	Sialin	SLC17A5	Transporters
**DUOX2_HUMAN**	Dual oxidase 2	DUOX2	Enzymes
**DUOX1_HUMAN**	Dual oxidase 1	DUOX1	Enzymes
**PCDBG_HUMAN**	Protocadherin beta-16	PCDHB16	Miscellaneous
**GTR9_HUMAN**	Solute carrier family 2, facilitated glucose transporter member 9	SLC2A9	Transporters
**I17RB_HUMAN**	Interleukin-17 receptor B	IL17RB	Receptors
**TRYG1_HUMAN**	Tryptase gamma	TPSG1	Unclassified
**SERC1_HUMAN**	Serine incorporator 1	SERINC1	Transporters
**THSD1_HUMAN**	Thrombospondin type-1 domain-containing protein 1	THSD1	Unclassified
**RPRM_HUMAN**	Protein reprimo	RPRM	Unclassified
**GP173_HUMAN**	Probable G-protein coupled receptor 173	GPR173	Receptors
**GPR27_HUMAN**	Probable G-protein coupled receptor 27	GPR27	Receptors
**TNR19_HUMAN**	Tumor necrosis factor receptor superfamily member 19	TNFRSF19	Receptors
**CLTR2_HUMAN**	Cysteinyl leukotriene receptor 2	CYSLTR2	Receptors
**AAA1_HUMAN**	Asc-type amino acid transporter 1	SLC7A10	Transporters
**TM7S3_HUMAN**	Transmembrane 7 superfamily member 3	TM7SF3	Unclassified
**S22AB_HUMAN**	Solute carrier family 22 member 11	SLC22A11	Transporters
**S6A13_HUMAN**	Sodium- and chloride-dependent GABA transporter 2	SLC6A13	Transporters
**RL3R1_HUMAN**	Relaxin-3 receptor 1	RXFP3	Receptors
**IGSF5_HUMAN**	Immunoglobulin superfamily member 5	IGSF5	Receptors
**TEN2_HUMAN**	Teneurin-2	TENM2	Miscellaneous
**LRC4B_HUMAN**	Leucine-rich repeat-containing protein 4B	LRRC4B	Miscellaneous
**PKDRE_HUMAN**	Polycystic kidney disease and receptor for egg jelly-related protein	PKDREJ	Transporters
**SEM4G_HUMAN**	Semaphorin-4G	SEMA4G	Miscellaneous
**CXB4_HUMAN**	Gap junction beta-4 protein	GJB4	Miscellaneous
**GINM1_HUMAN**	Glycoprotein integral membrane protein 1	GINM1	Unclassified

**S39A9_HUMAN**	Zinc transporter ZIP9	SLC39A9	Transporters
**T106B_HUMAN**	Transmembrane protein 106B	TMEM106B	Unclassified
**LMBD1_HUMAN**	Probable lysosomal cobalamin transporter	LMBRD1	Unclassified
**TM140_HUMAN**	Transmembrane protein 140	TMEM140	Unclassified
**CC50A_HUMAN**	Cell cycle control protein 50A	TMEM30A	Miscellaneous
**S52A1_HUMAN**	Solute carrier family 52, riboflavin transporter, member 1	SLC52A1	Receptors
**RHBL2_HUMAN**	Rhomboid-related protein 2	RHBDL2	Enzymes
**T161A_HUMAN**	Transmembrane protein 161A	TMEM161A	Unclassified
**ENK13_HUMAN**	Endogenous retrovirus group K member 13-1 Env polyprotein	ERVK13-1	Miscellaneous
**SIDT1_HUMAN**	SID1 transmembrane family member 1	SIDT1	Unclassified
**STAB1_HUMAN**	Stabilin-1	STAB1	Receptors
**CLC5A_HUMAN**	C-type lectin domain family 5 member A	CLEC5A	Receptors
**CLDN1_HUMAN**	Claudin domain-containing protein 1	CLDND1	Unclassified
**ASIC5_HUMAN**	Acid-sensing ion channel 5	ASIC5	Unclassified
**SCN3A_HUMAN**	Sodium channel protein type 3 subunit alpha	SCN3A	Transporters
**GTR8_HUMAN**	Solute carrier family 2, facilitated glucose transporter member 8	SLC2A8	Transporters
**SCN3B_HUMAN**	Sodium channel subunit beta-3	SCN3B	Transporters
**VNN3_HUMAN**	Vascular non-inflammatory molecule 3	VNN3	Unclassified
**SC5A4_HUMAN**	Low affinity sodium-glucose cotransporter	SLC5A4	Transporters
**SO1C1_HUMAN**	Solute carrier organic anion transporter family member 1C1	SLCO1C1	Transporters
**DLL3_HUMAN**	Delta-like protein 3	DLL3	Miscellaneous
**TLR7_HUMAN**	Toll-like receptor 7	TLR7	Receptors
**GPR83_HUMAN**	Probable G-protein coupled receptor 83	GPR83	Receptors
**CELR1_HUMAN**	Cadherin EGF LAG seven-pass G-type receptor 1	CELSR1	Receptors
**CELR3_HUMAN**	Cadherin EGF LAG seven-pass G-type receptor 3	CELSR3	Receptors
**FAT2_HUMAN**	Protocadherin Fat 2	FAT2	Miscellaneous
**T2R16_HUMAN**	Taste receptor type 2 member 16	TAS2R16	Receptors
**T2R14_HUMAN**	Taste receptor type 2 member 14	TAS2R14	Receptors
**T2R10_HUMAN**	Taste receptor type 2 member 10	TAS2R10	Receptors
**TA2R9_HUMAN**	Taste receptor type 2 member 9	TAS2R9	Receptors
**TA2R8_HUMAN**	Taste receptor type 2 member 8	TAS2R8	Receptors
**TA2R7_HUMAN**	Taste receptor type 2 member 7	TAS2R7	Receptors
**TA2R4_HUMAN**	Taste receptor type 2 member 4	TAS2R4	Receptors
**TA2R3_HUMAN**	Taste receptor type 2 member 3	TAS2R3	Receptors
**TA2R1_HUMAN**	Taste receptor type 2 member 1	TAS2R1	Receptors
**CALY_HUMAN**	Neuron-specific vesicular protein calcyon	CALY	Unclassified
**SIGL8_HUMAN**	Sialic acid-binding Ig-like lectin 8	SIGLEC8	Miscellaneous
**PDXL2_HUMAN**	Podocalyxin-like protein 2	PODXL2	Unclassified
**NLGN3_HUMAN**	Neuroligin-3	NLGN3	Miscellaneous
**TREM2_HUMAN**	Triggering receptor expressed on myeloid cells 2	TREM2	Receptors

**GPC5D_HUMAN**	G-protein coupled receptor family C group 5 member D	GPRC5D	Receptors
**GPC5B_HUMAN**	G-protein coupled receptor family C group 5 member B	GPRC5B	Receptors
**MYOF_HUMAN**	Myoferlin	MYOF	Miscellaneous
**IRPL1_HUMAN**	Interleukin-1 receptor accessory protein-like 1	IL1RAPL1	Receptors
**OR6C3_HUMAN**	Olfactory receptor 6C3	OR6C3	Receptors
**OR6C2_HUMAN**	Olfactory receptor 6C2	OR6C2	Receptors
**O5AC2_HUMAN**	Olfactory receptor 5AC2	OR5AC2	Receptors
**PD1L1_HUMAN**	Programmed cell death 1 ligand 1	CD274	Unclassified
**LRP1B_HUMAN**	Low-density lipoprotein receptor-related protein 1B	LRP1B	Receptors
**KLRF1_HUMAN**	Killer cell lectin-like receptor subfamily F member 1	KLRF1	Receptors
**FLRT3_HUMAN**	Leucine-rich repeat transmembrane protein FLRT3	FLRT3	Miscellaneous
**FLRT1_HUMAN**	Leucine-rich repeat transmembrane protein FLRT1	FLRT1	Miscellaneous
**CRIM1_HUMAN**	Cysteine-rich motor neuron 1 protein	CRIM1	Unclassified
**ADA22_HUMAN**	Disintegrin and metalloproteinase domain-containing protein 22	ADAM22	Enzymes
**PK2L1_HUMAN**	Polycystic kidney disease 2-like 1 protein	PKD2L1	Transporters
**TMEM9_HUMAN**	Transmembrane protein 9	TMEM9	Unclassified
**SLAF8_HUMAN**	SLAM family member 8	SLAMF8	Unclassified
**CAC1I_HUMAN**	Voltage-dependent T-type calcium channel subunit alpha-1I	CACNA1I	Transporters
**NTRI_HUMAN**	Neurotrimin	NTM	Unclassified
**CLC1B_HUMAN**	C-type lectin domain family 1 member B	CLEC1B	Receptors
**TAAR2_HUMAN**	Trace amine-associated receptor 2	TAAR2	Receptors
**OR1A1_HUMAN**	Olfactory receptor 1A1	OR1A1	Receptors
**CSC1_HUMAN**	Calcium permeable stress-gated cation channel 1	TMEM63C	Miscellaneous
**SIRPG_HUMAN**	Signal-regulatory protein gamma	SIRPG	Miscellaneous
**CNTN3_HUMAN**	Contactin-3	CNTN3	Unclassified
**LRFN1_HUMAN**	Leucine-rich repeat and fibronectin type III domain-containing protein 1	LRFN1	Miscellaneous
**TEN3_HUMAN**	Teneurin-3	TENM3	Miscellaneous
**SEM5B_HUMAN**	Semaphorin-5B	SEMA5B	Miscellaneous
**C5AR2_HUMAN**	C5a anaphylatoxin chemotactic receptor 2	C5AR2	Receptors
**FPRP_HUMAN**	Prostaglandin F2 receptor negative regulator	PTGFRN	Unclassified
**PCD10_HUMAN**	Protocadherin-10	PCDH10	Miscellaneous
**TUTLA_HUMAN**	Protein turtle homolog A	IGSF9	Miscellaneous
**PTHD2_HUMAN**	Patched domain-containing protein 2	PTCHD2	Unclassified
**NRX2A_HUMAN**	Neurexin-2	NRXN2	Receptors
**VGLU1_HUMAN**	Vesicular glutamate transporter 1	SLC17A7	Transporters
**VGLU2_HUMAN**	Vesicular glutamate transporter 2	SLC17A6	Transporters
**LRIT1_HUMAN**	Leucine-rich repeat, immunoglobulin-like domain and transmembrane domain-containing protein 1	LRIT1	Miscellaneous
**1C05_HUMAN**	HLA class I histocompatibility antigen, Cw-5 alpha chain	HLA-C	Miscellaneous
**2B19_HUMAN**	HLA class II histocompatibility antigen, DRB1-9 beta chain	HLA-DRB1	Miscellaneous
**RHCG_HUMAN**	Ammonium transporter Rh type C	RHCG	Transporters

**MRC2_HUMAN**	C-type mannose receptor 2	MRC2	Receptors
**P2RX2_HUMAN**	P2X purinoceptor 2	P2RX2	Transporters
**CCG4_HUMAN**	Voltage-dependent calcium channel gamma-4 subunit	CACNG4	Transporters
**TR10D_HUMAN**	Tumor necrosis factor receptor superfamily member 10D	TNFRSF10D	Receptors
**GABR1_HUMAN**	Gamma-aminobutyric acid type B receptor subunit 1	GABBR1	Receptors
**SUCO_HUMAN**	SUN domain-containing ossification factor	SUCO	Unclassified
**SL9A2_HUMAN**	Sodium/hydrogen exchanger 2	SLC9A2	Transporters
**LPAR3_HUMAN**	Lysophosphatidic acid receptor 3	LPAR3	Receptors
**KLOT_HUMAN**	Klotho	KL	Enzymes
**CCG5_HUMAN**	Voltage-dependent calcium channel gamma-5 subunit	CACNG5	Transporters
**EPHA6_HUMAN**	Ephrin type-A receptor 6	EPHA6	Receptors
**O14J1_HUMAN**	Olfactory receptor 14J1	OR14J1	Receptors
**OR5V1_HUMAN**	Olfactory receptor 5V1	OR5V1	Receptors
**O12D3_HUMAN**	Olfactory receptor 12D3	OR12D3	Receptors
**S23A2_HUMAN**	Solute carrier family 23 member 2	SLC23A2	Transporters
**ACHA9_HUMAN**	Neuronal acetylcholine receptor subunit alpha-9	CHRNA9	Transporters
**CLM8_HUMAN**	CMRF35-like molecule 8	CD300A	Receptors
**GTR6_HUMAN**	Solute carrier family 2, facilitated glucose transporter member 6	SLC2A6	Transporters
**SUSD2_HUMAN**	Sushi domain-containing protein 2	SUSD2	Unclassified
**CNTP2_HUMAN**	Contactin-associated protein-like 2	CNTNAP2	Receptors
**NPCL1_HUMAN**	Niemann-Pick C1-like protein 1	NPC1L1	Unclassified
**I20RA_HUMAN**	Interleukin-20 receptor subunit alpha	IL20RA	Receptors
**S23A1_HUMAN**	Solute carrier family 23 member 1	SLC23A1	Transporters
**OPN4_HUMAN**	Melanopsin	OPN4	Receptors
**CLC2D_HUMAN**	C-type lectin domain family 2 member D	CLEC2D	Receptors
**S12A6_HUMAN**	Solute carrier family 12 member 6	SLC12A6	Transporters
**EMR2_HUMAN**	EGF-like module-containing mucin-like hormone receptor-like 2	EMR2	Receptors
**SCNBA_HUMAN**	Sodium channel protein type 11 subunit alpha	SCN11A	Transporters
**NCKX2_HUMAN**	Sodium/potassium/calcium exchanger 2	SLC24A2	Transporters
**SLAF5_HUMAN**	SLAM family member 5	CD84	Unclassified
**SO3A1_HUMAN**	Solute carrier organic anion transporter family member 3A1	SLCO3A1	Transporters
**TEFF2_HUMAN**	Tomoregulin-2	TMEFF2	Unclassified
**LCAP_HUMAN**	Leucyl-cystinyl aminopeptidase	LNPEP	Enzymes
**PLXA1_HUMAN**	Plexin-A1	PLXNA1	Receptors
**GGT7_HUMAN**	Gamma-glutamyltransferase 7	GGT7	Unclassified
**GP160_HUMAN**	Probable G-protein coupled receptor 160	GPR160	Unclassified
**CAD22_HUMAN**	Cadherin-22	CDH22	Miscellaneous
**ENPP5_HUMAN**	Ectonucleotide pyrophosphatase/phosphodiesterase family member 5	ENPP5	Enzymes
**LAMP5_HUMAN**	Lysosome-associated membrane glycoprotein 5	LAMP5	Unclassified
**NAGPA_HUMAN**	N-acetylglucosamine-1-phosphodiester alpha-N-acetylglucosaminidase	NAGPA	Enzymes

**AJAP1_HUMAN**	Adherens junction-associated protein 1	AJAP1	Unclassified
**ADA30_HUMAN**	Disintegrin and metalloproteinase domain-containing protein 30	ADAM30	Enzymes
**ADA29_HUMAN**	Disintegrin and metalloproteinase domain-containing protein 29	ADAM29	Enzymes
**S13A4_HUMAN**	Solute carrier family 13 member 4	SLC13A4	Transporters
**ENK9_HUMAN**	Endogenous retrovirus group K member 9 Env polyprotein	ERVK-9	Miscellaneous
**PILRB_HUMAN**	Paired immunoglobulin-like type 2 receptor beta	PILRB	Unclassified
**PILRA_HUMAN**	Paired immunoglobulin-like type 2 receptor alpha	PILRA	Unclassified
**ADA21_HUMAN**	Disintegrin and metalloproteinase domain-containing protein 21	ADAM21	Enzymes
**O52A1_HUMAN**	Olfactory receptor 52A1	OR52A1	Receptors
**CXD2_HUMAN**	Gap junction delta-2 protein	GJD2	Miscellaneous
**MUC12_HUMAN**	Mucin-12	MUC12	Unclassified
**UR2R_HUMAN**	Urotensin-2 receptor	UTS2R	Receptors
**ADA28_HUMAN**	Disintegrin and metalloproteinase domain-containing protein 28	ADAM28	Enzymes
**TRHDE_HUMAN**	Thyrotropin-releasing hormone-degrading ectoenzyme	TRHDE	Enzymes
**ITA11_HUMAN**	Integrin alpha-11	ITGA11	Receptors
**PRND_HUMAN**	Prion-like protein doppel	PRND	Unclassified
**TEN1_HUMAN**	Teneurin-1	TENM1	Miscellaneous
**TM11E_HUMAN**	Transmembrane protease serine 11E	TMPRSS11E	Enzymes
**NRX1A_HUMAN**	Neurexin-1	NRXN1	Receptors
**CADH9_HUMAN**	Cadherin-9	CDH9	Miscellaneous
**CADH7_HUMAN**	Cadherin-7	CDH7	Miscellaneous
**MUCEN_HUMAN**	Endomucin	EMCN	Unclassified
**S39AA_HUMAN**	Zinc transporter ZIP10	SLC39A10	Transporters
**LRFN2_HUMAN**	Leucine-rich repeat and fibronectin type-III domain-containing protein 2	LRFN2	Miscellaneous
**HEG1_HUMAN**	Protein HEG homolog 1	HEG1	Unclassified
**GRID1_HUMAN**	Glutamate receptor ionotropic, delta-1	GRID1	Transporters
**RN150_HUMAN**	RING finger protein 150	RNF150	Unclassified
**PLXB3_HUMAN**	Plexin-B3	PLXNB3	Receptors
**TPC1_HUMAN**	Two pore calcium channel protein 1	TPCN1	Transporters
**FZD4_HUMAN**	Frizzled-4	FZD4	Receptors
**FZD10_HUMAN**	Frizzled-10	FZD10	Receptors
**CAH14_HUMAN**	Carbonic anhydrase 14	CA14	Enzymes
**MMP17_HUMAN**	Matrix metalloproteinase-17	MMP17	
**HHLA2_HUMAN**	HERV-H LTR-associating protein 2	HHLA2	Miscellaneous
**NOTC3_HUMAN**	Neurogenic locus notch homolog protein 3	NOTCH3	Receptors
**ALK_HUMAN**	ALK tyrosine kinase receptor	ALK	Unclassified
**ICAM5_HUMAN**	Intercellular adhesion molecule 5	ICAM5	Unclassified
**S45A2_HUMAN**	Membrane-associated transporter protein	SLC45A2	Transporters
**PTPRQ_HUMAN**	Phosphatidylinositol phosphatase PTPRQ	PTPRQ	Receptors
**AT1B4_HUMAN**	Protein ATP1B4	ATP1B4	Transporters

**PCDB8_HUMAN**	Protocadherin beta-8	PCDHB8	Miscellaneous
**PCDBA_HUMAN**	Protocadherin beta-10	PCDHB10	Miscellaneous
**PCDGK_HUMAN**	Protocadherin gamma-C3	PCDHGC3	
**PCDGG_HUMAN**	Protocadherin gamma-B4	PCDHGB4	
**PCDA7_HUMAN**	Protocadherin alpha-7	PCDHA7	
**PCDA6_HUMAN**	Protocadherin alpha-6	PCDHA6	
**PCDA4_HUMAN**	Protocadherin alpha-4	PCDHA4	
**PCDAC_HUMAN**	Protocadherin alpha-12	PCDHA12	
**S6A14_HUMAN**	Sodium- and chloride-dependent neutral and basic amino acid transporter B(0+)	SLC6A14	Transporters
**GBRT_HUMAN**	Gamma-aminobutyric acid receptor subunit theta	GABRQ	Transporters
**EDAR_HUMAN**	Tumor necrosis factor receptor superfamily member EDAR	EDAR	Receptors
**TNF18_HUMAN**	Tumor necrosis factor ligand superfamily member 18	TNFSF18	Unclassified
**EPCR_HUMAN**	Endothelial protein C receptor	PROCR	Unclassified
**ABCG2_HUMAN**	ATP-binding cassette sub-family G member 2	ABCG2	Transporters
**GP132_HUMAN**	Probable G-protein coupled receptor 132	GPR132	Receptors
**FZD1_HUMAN**	Frizzled-1	FZD1	Receptors
**S12A4_HUMAN**	Solute carrier family 12 member 4	SLC12A4	Transporters
**GPR34_HUMAN**	Probable G-protein coupled receptor 34	GPR34	Receptors
**FLVC2_HUMAN**	Feline leukemia virus subgroup C receptor-related protein 2	FLVCR2	Unclassified
**NAC2_HUMAN**	Sodium/calcium exchanger 2	SLC8A2	Transporters
**SORC3_HUMAN**	VPS10 domain-containing receptor SorCS3	SORCS3	Receptors
**TUTLB_HUMAN**	Protein turtle homolog B	IGSF9B	Miscellaneous
**THS7A_HUMAN**	Thrombospondin type-1 domain-containing protein 7A	THSD7A	Unclassified
**CNTN6_HUMAN**	Contactin-6	CNTN6	Unclassified
**CLCA2_HUMAN**	Calcium-activated chloride channel regulator 2	CLCA2	Unclassified
**SCN8A_HUMAN**	Sodium channel protein type 8 subunit alpha	SCN8A	Transporters
**SYCY1_HUMAN**	Syncytin-1	ERVW-1	Miscellaneous
**NALDL_HUMAN**	N-acetylated-alpha-linked acidic dipeptidase-like protein	NAALADL1	
**LAMP3_HUMAN**	Lysosome-associated membrane glycoprotein 3	LAMP3	Receptors
**JAG2_HUMAN**	Protein jagged-2	JAG2	Miscellaneous
**S22AD_HUMAN**	Solute carrier family 22 member 13	SLC22A13	Transporters
**S22AE_HUMAN**	Solute carrier family 22 member 14	SLC22A14	Transporters
**CLTR1_HUMAN**	Cysteinyl leukotriene receptor 1	CYSLTR1	Receptors
**TN13B_HUMAN**	Tumor necrosis factor ligand superfamily member 13B	TNFSF13B	Unclassified
**SIGL7_HUMAN**	Sialic acid-binding Ig-like lectin 7	SIGLEC7	Miscellaneous
**ITM2B_HUMAN**	Integral membrane protein 2B	ITM2B	Miscellaneous
**SC5A6_HUMAN**	Sodium-dependent multivitamin transporter	SLC5A6	Transporters
**TLR6_HUMAN**	Toll-like receptor 6	TLR6	Receptors
**NTNG1_HUMAN**	Netrin-G1	NTNG1	
**GPR52_HUMAN**	Probable G-protein coupled receptor 52	GPR52	Receptors

**GPR55_HUMAN**	G-protein coupled receptor 55	GPR55	Receptors
**SIGL9_HUMAN**	Sialic acid-binding Ig-like lectin 9	SIGLEC9	Miscellaneous
**SC6A5_HUMAN**	Sodium- and chloride-dependent glycine transporter 2	SLC6A5	Transporters
**TMED7_HUMAN**	Transmembrane emp24 domain-containing protein 7	TMED7	Miscellaneous
**OR2W1_HUMAN**	Olfactory receptor 2W1	OR2W1	Receptors
**SIT1_HUMAN**	Signaling threshold-regulating transmembrane adapter 1	SIT1	Unclassified
**ADA18_HUMAN**	Disintegrin and metalloproteinase domain-containing protein 18	ADAM18	Enzymes
**VPP2_HUMAN**	V-type proton ATPase 116 kDa subunit a isoform 2	ATP6V0A2	Unclassified
**ZAN_HUMAN**	Zonadhesin	ZAN	Unclassified
**O10H1_HUMAN**	Olfactory receptor 10H1	OR10H1	Receptors
**NRX3A_HUMAN**	Neurexin-3	NRXN3	Receptors
**DGLA_HUMAN**	Sn1-specific diacylglycerol lipase alpha	DAGLA	Unclassified
**PLXD1_HUMAN**	Plexin-D1	PLXND1	Receptors
**LRP12_HUMAN**	Low-density lipoprotein receptor-related protein 12	LRP12	Receptors
**OR1A2_HUMAN**	Olfactory receptor 1A2	OR1A2	Receptors
**PCDB9_HUMAN**	Protocadherin beta-9	PCDHB9	Miscellaneous
**PCDB7_HUMAN**	Protocadherin beta-7	PCDHB7	Miscellaneous
**PCDB6_HUMAN**	Protocadherin beta-6	PCDHB6	Miscellaneous
**PCDB5_HUMAN**	Protocadherin beta-5	PCDHB5	Miscellaneous
**PCDB4_HUMAN**	Protocadherin beta-4	PCDHB4	Miscellaneous
**PCDB3_HUMAN**	Protocadherin beta-3	PCDHB3	Miscellaneous
**PCDB2_HUMAN**	Protocadherin beta-2	PCDHB2	Miscellaneous
**PCDBF_HUMAN**	Protocadherin beta-15	PCDHB15	Miscellaneous
**PCDBE_HUMAN**	Protocadherin beta-14	PCDHB14	Miscellaneous
**PCDBD_HUMAN**	Protocadherin beta-13	PCDHB13	Miscellaneous
**PCDBC_HUMAN**	Protocadherin beta-12	PCDHB12	Miscellaneous
**PCDBB_HUMAN**	Protocadherin beta-11	PCDHB11	Miscellaneous
**PCDB1_HUMAN**	Protocadherin beta-1	PCDHB1	Miscellaneous
**PCDGM_HUMAN**	Protocadherin gamma-C5	PCDHGC5	Unclassified
**PCDGL_HUMAN**	Protocadherin gamma-C4	PCDHGC4	Unclassified
**PCDGJ_HUMAN**	Protocadherin gamma-B7	PCDHGB7	
**PCDGI_HUMAN**	Protocadherin gamma-B6	PCDHGB6	
**PCDGH_HUMAN**	Protocadherin gamma-B5	PCDHGB5	
**PCDGF_HUMAN**	Protocadherin gamma-B3	PCDHGB3	
**PCDGE_HUMAN**	Protocadherin gamma-B2	PCDHGB2	
**PCDGD_HUMAN**	Protocadherin gamma-B1	PCDHGB1	
**PCDG9_HUMAN**	Protocadherin gamma-A9	PCDHGA9	
**PCDG8_HUMAN**	Protocadherin gamma-A8	PCDHGA8	
**PCDG7_HUMAN**	Protocadherin gamma-A7	PCDHGA7	
**PCDG6_HUMAN**	Protocadherin gamma-A6	PCDHGA6	

**PCDG5_HUMAN**	Protocadherin gamma-A5	PCDHGA5	Miscellaneous
**PCDG4_HUMAN**	Protocadherin gamma-A4	PCDHGA4	Unclassified
**PCDG3_HUMAN**	Protocadherin gamma-A3	PCDHGA3	
**PCDG2_HUMAN**	Protocadherin gamma-A2	PCDHGA2	
**PCDGB_HUMAN**	Protocadherin gamma-A11	PCDHGA11	Unclassified
**PCDGA_HUMAN**	Protocadherin gamma-A10	PCDHGA10	
**PCDG1_HUMAN**	Protocadherin gamma-A1	PCDHGA1	
**PCDA9_HUMAN**	Protocadherin alpha-9	PCDHA9	
**PCDA8_HUMAN**	Protocadherin alpha-8	PCDHA8	
**PCDA5_HUMAN**	Protocadherin alpha-5	PCDHA5	
**PCDA3_HUMAN**	Protocadherin alpha-3	PCDHA3	
**PCDA2_HUMAN**	Protocadherin alpha-2	PCDHA2	
**PCDAD_HUMAN**	Protocadherin alpha-13	PCDHA13	
**PCDAB_HUMAN**	Protocadherin alpha-11	PCDHA11	
**PCDAA_HUMAN**	Protocadherin alpha-10	PCDHA10	
**PCDA1_HUMAN**	Protocadherin alpha-1	PCDHA1	
**PCDC2_HUMAN**	Protocadherin alpha-C2	PCDHAC2	Unclassified
**CLD16_HUMAN**	Claudin-16	CLDN16	Miscellaneous
**HRH3_HUMAN**	Histamine H3 receptor	HRH3	Receptors
**O51B4_HUMAN**	Olfactory receptor 51B4	OR51B4	Receptors
**O51B2_HUMAN**	Olfactory receptor 51B2	OR51B2	Receptors
**CORIN_HUMAN**	Atrial natriuretic peptide-converting enzyme	CORIN	Enzymes
**TRPV2_HUMAN**	Transient receptor potential cation channel subfamily V member 2	TRPV2	Transporters
**TNR18_HUMAN**	Tumor necrosis factor receptor superfamily member 18	TNFRSF18	Receptors
**NPFF2_HUMAN**	Neuropeptide FF receptor 2	NPFFR2	Receptors
**FLVC1_HUMAN**	Feline leukemia virus subgroup C receptor-related protein 1	FLVCR1	Unclassified
**GPR45_HUMAN**	Probable G-protein coupled receptor 45	GPR45	Receptors
**PD2R2_HUMAN**	Prostaglandin D2 receptor 2	PTGDR2	Receptors
**LYVE1_HUMAN**	Lymphatic vessel endothelial hyaluronic acid receptor 1	LYVE1	Unclassified
**SCNAA_HUMAN**	Sodium channel protein type 10 subunit alpha	SCN10A	Transporters
**BACE2_HUMAN**	Beta-secretase 2	BACE2	Enzymes
**JAM1_HUMAN**	Junctional adhesion molecule A	F11R	Miscellaneous
**GPC6_HUMAN**	Glypican-6	GPC6	Unclassified
**NPTN_HUMAN**	Neuroplastin	NPTN	Unclassified
**GPR56_HUMAN**	G-protein coupled receptor 56	GPR56	Receptors
**S12A7_HUMAN**	Solute carrier family 12 member 7	SLC12A7	Transporters
**KCMB2_HUMAN**	Calcium-activated potassium channel subunit beta-2	KCNMB2	Transporters
**S22A7_HUMAN**	Solute carrier family 22 member 7	SLC22A7	Transporters
**CCG2_HUMAN**	Voltage-dependent calcium channel gamma-2 subunit	CACNG2	Transporters
**MRVI1_HUMAN**	Protein MRVI1	MRVI1	Unclassified

**CXA3_HUMAN**	Gap junction alpha-3 protein	GJA3	Miscellaneous
**SO1B1_HUMAN**	Solute carrier organic anion transporter family member 1B1	SLCO1B1	Transporters
**TEST_HUMAN**	Testisin	PRSS21	
**ZNT1_HUMAN**	Zinc transporter 1	SLC30A1	Transporters
**S4A7_HUMAN**	Sodium bicarbonate cotransporter 3	SLC4A7	Transporters
**ROBO1_HUMAN**	Roundabout homolog 1	ROBO1	Receptors
**CAD10_HUMAN**	Cadherin-10	CDH10	Miscellaneous
**TNR11_HUMAN**	Tumor necrosis factor receptor superfamily member 11A	TNFRSF11A	Receptors
**S4A4_HUMAN**	Electrogenic sodium bicarbonate cotransporter 1	SLC4A4	Transporters
**ICOS_HUMAN**	Inducible T-cell costimulator	ICOS	Unclassified
**ENPP4_HUMAN**	Bis(5'-adenosyl)-triphosphatase ENPP4	ENPP4	Enzymes

Supplementary Table S2. List of differentially expressed genes with a quasi H-score ≥150 per tumor type

Gene name	Cancer	High	Medium	Low	Not detected	Sum	Quasi H-Score
**ABCA2**	glioma	5	6	0	0	11	245.45
**ABCA2**	head and neck cancer	1	3	0	0	4	225
**ABCA2**	thyroid cancer	1	3	0	0	4	225
**ABCA2**	melanoma	2	10	0	0	12	216.67
**ABCA2**	ovarian cancer	1	11	0	0	12	208.33
**ABCA2**	breast cancer	0	10	0	0	10	200
**ABCA2**	endometrial cancer	1	9	1	0	11	200
**ABCA2**	pancreatic cancer	0	10	0	0	10	200
**ABCA2**	prostate cancer	0	12	0	0	12	200
**ABCA2**	skin cancer	0	11	0	0	11	200
**ABCA2**	stomach cancer	0	10	0	0	10	200
**ABCA2**	testis cancer	0	11	0	0	11	200
**ABCA2**	cervical cancer	0	11	1	0	12	191.67
**ABCA2**	lung cancer	0	11	1	0	12	191.67
**ABCA2**	liver cancer	0	9	2	0	11	181.82
**ABCA2**	urothelial cancer	1	8	1	1	11	181.82
**ABCA2**	carcinoid	1	2	0	1	4	175
**ABCA2**	renal cancer	0	8	4	0	12	166.67
**ABCA2**	lymphoma	0	8	2	2	12	150
**ABCB1**	liver cancer	1	6	1	2	10	160
**ABCB9**	testis cancer	0	10	2	0	12	183.33
**ABCB9**	colorectal cancer	0	9	3	0	12	175
**ABCB9**	breast cancer	0	8	4	0	12	166.67
**ABCC10**	thyroid cancer	0	4	0	0	4	200
**ABCC10**	renal cancer	2	8	1	1	12	191.67
**ABCC10**	prostate cancer	0	7	4	0	11	163.64
**ABCC10**	ovarian cancer	2	3	6	1	12	150
**ABCC11**	thyroid cancer	2	2	0	0	4	250
**ABCC11**	urothelial cancer	5	3	3	0	11	218.18
**ABCC11**	liver cancer	5	4	3	0	12	216.67
**ABCC11**	breast cancer	2	8	1	0	11	209.09
**ABCC11**	colorectal cancer	2	8	1	0	11	209.09
**ABCC11**	endometrial cancer	2	8	2	0	12	200
**ABCC11**	head and neck cancer	0	4	0	0	4	200
**ABCC11**	stomach cancer	0	10	0	0	10	200
**ABCC11**	pancreatic cancer	0	11	1	0	12	191.67
**ABCC11**	ovarian cancer	1	8	3	0	12	183.33
**ABCC11**	skin cancer	1	8	3	0	12	183.33

**ABCC11**	lung cancer	3	5	2	2	12	175
**ABCC11**	carcinoid	0	3	1	0	4	175
**ABCC11**	prostate cancer	0	9	3	0	12	175
**ABCC11**	testis cancer	0	7	4	0	11	163.64
**ABCC11**	melanoma	0	7	3	1	11	154.55
**ACVR1B**	liver cancer	4	4	1	2	11	190.91
**ADAM15**	liver cancer	1	9	2	0	12	191.67
**ADAM15**	melanoma	2	5	5	0	12	175
**ADAM15**	colorectal cancer	0	9	3	0	12	175
**ADAM15**	thyroid cancer	0	2	1	0	3	166.67
**ADAM15**	pancreatic cancer	0	7	4	0	11	163.64
**ADAM15**	prostate cancer	0	6	4	0	10	160
**ADAM15**	breast cancer	0	7	5	0	12	158.33
**ADAM15**	testis cancer	0	7	5	0	12	158.33
**ADAM17**	colorectal cancer	8	4	0	0	12	266.67
**ADAM17**	carcinoid	2	2	0	0	4	250
**ADAM17**	endometrial cancer	4	3	4	0	11	200
**ADAM17**	testis cancer	2	4	6	0	12	166.67
**ADAM17**	pancreatic cancer	1	3	4	0	8	162.5
**ADAM17**	lung cancer	2	5	3	2	12	158.33
**ADAM17**	urothelial cancer	1	5	6	0	12	158.33
**ADAM17**	ovarian cancer	3	3	4	2	12	158.33
**ADAM17**	glioma	0	8	2	2	12	150
**ADAM17**	head and neck cancer	0	2	2	0	4	150
**ADAM17**	stomach cancer	4	1	4	3	12	150
**ADAM23**	carcinoid	0	4	0	0	4	200
**ADAM23**	thyroid cancer	1	2	1	0	4	200
**ADAM23**	breast cancer	0	9	3	0	12	175
**ADAM23**	ovarian cancer	1	4	7	0	12	150
**ADAM33**	thyroid cancer	0	4	0	0	4	200
**ADAM8**	colorectal cancer	0	11	1	0	12	191.67
**ADAM8**	endometrial cancer	0	8	2	0	10	180
**ADAM8**	stomach cancer	0	7	4	1	12	150
**ADAM8**	thyroid cancer	0	2	2	0	4	150
**ADCY2**	prostate cancer	10	1	0	0	11	290.91
**ADCY2**	breast cancer	4	6	2	0	12	216.67
**ADCY2**	carcinoid	0	2	0	0	2	200
**ADCY2**	endometrial cancer	1	9	1	0	11	200
**ADCY2**	colorectal cancer	0	10	1	0	11	190.91
**ADCY2**	liver cancer	2	8	0	2	12	183.33

**ADCY2**	thyroid cancer	0	3	1	0	4	175
**ADCY2**	pancreatic cancer	1	6	2	1	10	170
**ADCY2**	melanoma	0	7	2	1	10	160
**ADCY2**	renal cancer	1	5	6	0	12	158.33
**ADCY3**	prostate cancer	12	0	0	0	12	300
**ADCY3**	renal cancer	0	7	5	0	12	158.33
**ADCY6**	liver cancer	2	6	2	1	11	181.82
**ADCY6**	breast cancer	1	4	7	0	12	150
**ADCY9**	colorectal cancer	0	9	2	0	11	181.82
**ADCY9**	head and neck cancer	0	3	1	0	4	175
**ADCY9**	melanoma	0	8	4	0	12	166.67
**ADCY9**	testis cancer	0	8	3	1	12	158.33
**ADCY9**	pancreatic cancer	0	4	4	0	8	150
**ALK**	skin cancer	4	1	4	1	10	180
**ALK**	prostate cancer	1	6	4	1	12	158.33
**ALPL**	testis cancer	4	3	1	3	11	172.73
**ANO1**	liver cancer	7	2	0	3	12	208.33
**ANO1**	stomach cancer	3	4	0	2	9	188.89
**ANO7**	prostate cancer	9	0	0	2	11	245.45
**ANO9**	head and neck cancer	1	1	1	0	3	200
**ANO9**	breast cancer	6	0	3	2	11	190.91
**ANO9**	melanoma	2	6	3	0	11	190.91
**ANO9**	ovarian cancer	2	7	2	1	12	183.33
**ANO9**	renal cancer	3	4	1	4	12	150
**AQP5**	endometrial cancer	4	6	2	0	12	216.67
**AQP5**	pancreatic cancer	3	2	0	3	8	162.5
**AQP8**	prostate cancer	3	4	3	0	10	200
**ATP13A1**	thyroid cancer	0	4	0	0	4	200
**ATP13A1**	colorectal cancer	1	8	3	0	12	183.33
**ATP1B3**	skin cancer	0	9	2	0	11	181.82
**ATP1B3**	thyroid cancer	0	3	1	0	4	175
**ATP1B3**	stomach cancer	0	7	3	0	10	170
**ATP1B3**	pancreatic cancer	0	8	4	0	12	166.67
**ATP1B3**	lung cancer	0	6	6	0	12	150
**ATP2B2**	glioma	0	9	1	0	10	190
**ATP2B2**	thyroid cancer	0	3	1	0	4	175
**ATP2B2**	carcinoid	0	2	1	0	3	166.67
**ATP2B2**	endometrial cancer	0	7	4	0	11	163.64
**ATP2B2**	liver cancer	1	5	5	1	12	150
**ATRN**	prostate cancer	1	7	4	0	12	175

**BACE1**	colorectal cancer	9	3	0	0	12	275
**BACE1**	liver cancer	8	3	0	0	11	272.73
**BACE1**	melanoma	8	4	0	0	12	266.67
**BACE1**	endometrial cancer	6	6	0	0	12	250
**BACE1**	testis cancer	5	5	0	0	10	250
**BACE1**	lung cancer	4	4	1	0	9	233.33
**BACE1**	thyroid cancer	2	0	1	0	3	233.33
**BACE1**	lymphoma	2	9	0	0	11	218.18
**BACE1**	pancreatic cancer	3	7	1	0	11	218.18
**BACE1**	stomach cancer	3	7	1	0	11	218.18
**BACE1**	ovarian cancer	2	9	1	0	12	208.33
**BACE1**	glioma	1	8	1	0	10	200
**BACE1**	prostate cancer	1	10	1	0	12	200
**BACE1**	urothelial cancer	2	7	3	0	12	191.67
**BACE1**	breast cancer	2	6	4	0	12	183.33
**BACE1**	cervical cancer	4	3	4	1	12	183.33
**BACE1**	renal cancer	1	8	1	2	12	166.67
**BACE2**	prostate cancer	0	7	1	2	10	150
**BAMBI**	prostate cancer	1	9	0	2	12	175
**BAMBI**	colorectal cancer	0	7	5	0	12	158.33
**C11orf24**	thyroid cancer	0	3	0	0	3	200
**CALCR**	prostate cancer	0	10	1	1	12	175
**CCR2**	skin cancer	8	1	1	1	11	245.45
**CCR2**	head and neck cancer	2	1	0	1	4	200
**CCR3**	liver cancer	5	2	0	5	12	158.33
**CCR9**	breast cancer	12	0	0	0	12	300
**CCR9**	thyroid cancer	4	0	0	0	4	300
**CCR9**	pancreatic cancer	10	0	1	0	11	281.82
**CCR9**	renal cancer	10	1	1	0	12	275
**CCR9**	endometrial cancer	9	1	1	0	11	272.73
**CCR9**	ovarian cancer	10	0	2	0	12	266.67
**CCR9**	prostate cancer	8	4	0	0	12	266.67
**CCR9**	urothelial cancer	8	2	1	0	11	263.64
**CCR9**	cervical cancer	7	4	1	0	12	250
**CCR9**	stomach cancer	7	4	1	0	12	250
**CCR9**	testis cancer	6	4	1	0	11	245.45
**CCR9**	lung cancer	6	2	2	0	10	240
**CCR9**	skin cancer	6	4	0	1	11	236.36
**CCR9**	liver cancer	6	2	1	1	10	230
**CCR9**	glioma	5	4	2	0	11	227.27

**CCR9**	carcinoid	2	1	1	0	4	225
**CCR9**	melanoma	4	5	2	0	11	218.18
**CCR9**	head and neck cancer	1	1	2	0	4	175
**CCR9**	colorectal cancer	3	1	7	0	11	163.64
**CD101**	thyroid cancer	1	2	1	0	4	200
**CD101**	melanoma	0	7	5	0	12	158.33
**CD19**	lymphoma	5	3	0	3	11	190.91
**CD1B**	prostate cancer	6	2	0	0	8	275
**CD1B**	thyroid cancer	2	1	0	0	3	266.67
**CD1B**	liver cancer	5	5	1	1	12	216.67
**CD1B**	head and neck cancer	0	2	1	0	3	166.67
**CD1B**	lung cancer	0	6	2	1	9	155.56
**CD276**	skin cancer	7	4	1	0	12	250
**CD276**	endometrial cancer	4	5	1	0	10	230
**CD276**	head and neck cancer	1	3	0	0	4	225
**CD276**	prostate cancer	3	9	0	0	12	225
**CD276**	melanoma	2	9	1	0	12	208.33
**CD276**	glioma	4	2	6	0	12	183.33
**CD276**	lung cancer	2	6	4	0	12	183.33
**CD276**	urothelial cancer	1	8	3	0	12	183.33
**CD276**	breast cancer	1	7	4	0	12	175
**CD276**	pancreatic cancer	2	4	6	0	12	166.67
**CD276**	stomach cancer	1	4	5	0	10	160
**CD276**	ovarian cancer	2	3	7	0	12	158.33
**CD276**	cervical cancer	0	7	4	1	12	150
**CD33**	colorectal cancer	1	7	4	0	12	175
**CD37**	lymphoma	6	3	3	0	12	225
**CD3D**	lymphoma	4	5	0	3	12	183.33
**CD3G**	thyroid cancer	1	2	0	0	3	233.33
**CD47**	ovarian cancer	6	2	2	1	11	218.18
**CD47**	thyroid cancer	1	2	0	1	4	175
**CD53**	stomach cancer	1	6	1	0	8	200
**CD53**	thyroid cancer	0	2	0	0	2	200
**CD53**	glioma	0	7	2	0	9	177.78
**CD53**	lymphoma	4	3	3	2	12	175
**CD53**	pancreatic cancer	0	8	3	0	11	172.73
**CD53**	colorectal cancer	0	6	5	0	11	154.55
**CD53**	liver cancer	0	6	6	0	12	150
**CD63**	melanoma	7	3	2	0	12	241.67
**CD63**	prostate cancer	0	6	6	0	12	150

**CD74**	lymphoma	0	9	0	0	9	200
**CD74**	endometrial cancer	1	6	3	0	10	180
**CD74**	thyroid cancer	0	3	1	0	4	175
**CD74**	head and neck cancer	0	3	0	1	4	150
**CD79A**	lymphoma	10	0	0	2	12	250
**CD79B**	lymphoma	5	4	3	0	12	216.67
**CD83**	lymphoma	1	8	1	0	10	200
**CD86**	colorectal cancer	0	10	0	0	10	200
**CD86**	lymphoma	5	3	2	2	12	191.67
**CD86**	stomach cancer	4	2	5	1	12	175
**CD86**	testis cancer	2	4	5	1	12	158.33
**CD86**	thyroid cancer	0	2	2	0	4	150
**CDCP1**	colorectal cancer	1	9	2	0	12	191.67
**CDCP1**	pancreatic cancer	2	5	2	3	12	150
**CDCP1**	endometrial cancer	0	8	2	2	12	150
**CDH11**	thyroid cancer	1	1	0	1	3	166.67
**CDH18**	carcinoid	0	2	2	0	4	150
**CDH20**	cervical cancer	5	4	3	0	12	216.67
**CDH20**	colorectal cancer	3	8	1	0	12	216.67
**CDH20**	prostate cancer	4	6	2	0	12	216.67
**CDH20**	ovarian cancer	4	5	3	0	12	208.33
**CDH20**	head and neck cancer	1	1	1	0	3	200
**CDH20**	thyroid cancer	1	1	1	0	3	200
**CDH20**	urothelial cancer	0	11	1	0	12	191.67
**CDH20**	pancreatic cancer	1	7	3	0	11	181.82
**CDH20**	lung cancer	2	5	2	1	10	180
**CDH20**	stomach cancer	1	7	4	0	12	175
**CDH20**	breast cancer	0	8	4	0	12	166.67
**CDH20**	carcinoid	0	2	1	0	3	166.67
**CDH20**	endometrial cancer	1	5	6	0	12	158.33
**CDH20**	renal cancer	0	7	4	1	12	150
**CDH5**	breast cancer	3	7	1	1	12	200
**CDH8**	colorectal cancer	5	6	0	0	11	245.45
**CELSR3**	colorectal cancer	3	8	1	0	12	216.67
**CELSR3**	carcinoid	0	3	1	0	4	175
**CELSR3**	stomach cancer	0	8	3	0	11	172.73
**CELSR3**	liver cancer	0	8	1	2	11	154.55
**CELSR3**	testis cancer	1	5	5	1	12	150
**CHPT1**	breast cancer	0	7	2	1	10	160
**CHPT1**	thyroid cancer	0	2	2	0	4	150

**CHRNA7**	thyroid cancer	1	3	0	0	4	225
**CHRNA7**	colorectal cancer	2	8	1	0	11	209.09
**CHRNA7**	melanoma	5	5	0	2	12	208.33
**CHRNA7**	carcinoid	0	4	0	0	4	200
**CHRNA7**	head and neck cancer	0	4	0	0	4	200
**CHRNA7**	ovarian cancer	2	7	2	0	11	200
**CHRNA7**	stomach cancer	1	6	1	0	8	200
**CHRNA7**	urothelial cancer	3	5	2	1	11	190.91
**CHRNA7**	glioma	0	8	1	0	9	188.89
**CHRNA7**	liver cancer	0	10	2	0	12	183.33
**CHRNA7**	endometrial cancer	1	9	1	1	12	183.33
**CHRNA7**	testis cancer	1	7	3	0	11	181.82
**CHRNA7**	prostate cancer	0	9	0	1	10	180
**CHRNA7**	pancreatic cancer	1	6	4	0	11	172.73
**CHRNA7**	breast cancer	0	7	3	0	10	170
**CHRNB1**	liver cancer	5	3	1	0	9	244.44
**CHRNB1**	colorectal cancer	4	8	0	0	12	233.33
**CHRNB1**	breast cancer	4	6	1	0	11	227.27
**CHRNB1**	ovarian cancer	5	5	2	0	12	225
**CHRNB1**	endometrial cancer	3	7	1	0	11	218.18
**CHRNB1**	prostate cancer	3	7	1	0	11	218.18
**CHRNB1**	cervical cancer	6	3	2	1	12	216.67
**CHRNB1**	glioma	4	4	1	1	10	210
**CHRNB1**	carcinoid	0	3	0	0	3	200
**CHRNB1**	head and neck cancer	0	4	0	0	4	200
**CHRNB1**	lymphoma	4	2	2	1	9	200
**CHRNB1**	melanoma	2	6	2	0	10	200
**CHRNB1**	urothelial cancer	2	7	2	0	11	200
**CHRNB1**	renal cancer	5	3	1	2	11	200
**CHRNB1**	testis cancer	5	4	1	2	12	200
**CHRNB1**	thyroid cancer	0	3	1	0	4	175
**CHRNB1**	pancreatic cancer	0	7	4	0	11	163.64
**CLCNKB**	testis cancer	0	8	1	0	9	188.89
**CLRN1**	thyroid cancer	0	4	0	0	4	200
**CLRN1**	colorectal cancer	0	11	1	0	12	191.67
**CLRN1**	prostate cancer	0	10	1	0	11	190.91
**CLRN1**	breast cancer	0	8	2	0	10	180
**CMKLR1**	thyroid cancer	0	4	0	0	4	200
**CNR1**	thyroid cancer	2	1	1	0	4	225
**CNTN6**	colorectal cancer	1	7	2	1	11	172.73

**CNTN6**	ovarian cancer	2	5	4	1	12	166.67
**CNTN6**	endometrial cancer	0	8	4	0	12	166.67
**CNTN6**	melanoma	0	7	2	1	10	160
**CNTN6**	stomach cancer	1	5	2	2	10	150
**CNTN6**	thyroid cancer	0	2	2	0	4	150
**CRIM1**	colorectal cancer	0	9	2	1	12	166.67
**CSF1R**	thyroid cancer	0	4	0	0	4	200
**CSF1R**	ovarian cancer	1	5	3	0	9	177.78
**CSF1R**	stomach cancer	0	5	5	0	10	150
**CSPG5**	testis cancer	0	8	3	0	11	172.73
**CXCR5**	prostate cancer	0	8	3	1	12	158.33
**DCBLD2**	colorectal cancer	0	10	0	0	10	200
**DCBLD2**	head and neck cancer	0	3	0	0	3	200
**DCBLD2**	melanoma	0	9	0	0	9	200
**DCBLD2**	endometrial cancer	0	9	2	0	11	181.82
**DCBLD2**	pancreatic cancer	0	9	0	1	10	180
**DCBLD2**	liver cancer	0	10	1	1	12	175
**DCBLD2**	ovarian cancer	0	8	4	0	12	166.67
**DCBLD2**	carcinoid	0	2	2	0	4	150
**DCBLD2**	thyroid cancer	0	2	2	0	4	150
**DSCAM**	glioma	6	2	1	3	12	191.67
**DSCAM**	testis cancer	2	5	1	3	11	154.55
**DSCAM**	carcinoid	1	1	1	1	4	150
**DUOXA1**	lymphoma	1	8	1	1	11	181.82
**DUOXA1**	head and neck cancer	0	3	1	0	4	175
**EDNRA**	head and neck cancer	0	4	0	0	4	200
**EDNRA**	skin cancer	1	9	2	0	12	191.67
**EDNRA**	pancreatic cancer	2	6	3	0	11	190.91
**EDNRA**	cervical cancer	0	9	3	0	12	175
**EDNRA**	thyroid cancer	0	3	1	0	4	175
**EDNRA**	ovarian cancer	0	8	3	0	11	172.73
**EDNRA**	glioma	1	8	1	2	12	166.67
**EDNRA**	stomach cancer	0	6	2	1	9	155.56
**EDNRA**	breast cancer	1	5	5	1	12	150
**EDNRA**	colorectal cancer	0	6	6	0	12	150
**EDNRB**	breast cancer	1	11	0	0	12	208.33
**EDNRB**	ovarian cancer	0	8	2	2	12	150
**EFNA3**	thyroid cancer	3	0	0	0	3	300
**EFNA3**	testis cancer	8	4	0	0	12	266.67
**EFNA3**	liver cancer	6	5	1	0	12	241.67

**EFNA3**	glioma	3	7	0	0	10	230
**EFNA3**	endometrial cancer	2	9	0	0	11	218.18
**EFNA3**	colorectal cancer	2	10	0	0	12	216.67
**EFNA3**	stomach cancer	4	6	2	0	12	216.67
**EFNA3**	urothelial cancer	2	9	1	0	12	208.33
**EFNA3**	breast cancer	1	10	1	0	12	200
**EFNA3**	carcinoid	1	2	1	0	4	200
**EFNA3**	head and neck cancer	0	3	0	0	3	200
**EFNA3**	melanoma	0	10	0	0	10	200
**EFNA3**	pancreatic cancer	2	8	2	0	12	200
**EFNA3**	renal cancer	2	7	2	0	11	200
**EFNA3**	prostate cancer	0	10	2	0	12	183.33
**EFNA3**	cervical cancer	1	6	5	0	12	166.67
**EFNA3**	skin cancer	0	8	4	0	12	166.67
**EFNA3**	ovarian cancer	0	7	4	0	11	163.64
**EFNA3**	lymphoma	0	7	5	0	12	158.33
**EFNA3**	lung cancer	1	5	5	1	12	150
**EFNA4**	colorectal cancer	3	9	0	0	12	225
**EFNA4**	endometrial cancer	1	11	0	0	12	208.33
**EFNA4**	head and neck cancer	0	3	0	0	3	200
**EFNA4**	melanoma	0	11	0	0	11	200
**EFNA4**	ovarian cancer	0	11	0	0	11	200
**EFNA4**	pancreatic cancer	1	8	1	0	10	200
**EFNA4**	stomach cancer	1	9	1	0	11	200
**EFNA4**	testis cancer	0	9	0	0	9	200
**EFNA4**	thyroid cancer	0	4	0	0	4	200
**EFNA4**	liver cancer	0	11	1	0	12	191.67
**EFNA4**	cervical cancer	0	11	0	1	12	183.33
**EFNA4**	lung cancer	0	8	3	1	12	158.33
**EFNA4**	carcinoid	0	2	2	0	4	150
**EFNA4**	urothelial cancer	0	7	4	1	12	150
**ENPP5**	testis cancer	3	8	0	0	11	227.27
**ENPP5**	breast cancer	1	10	1	0	12	200
**ENPP5**	carcinoid	0	4	0	0	4	200
**ENPP5**	prostate cancer	0	12	0	0	12	200
**ENPP5**	thyroid cancer	0	4	0	0	4	200
**ENPP5**	colorectal cancer	0	10	2	0	12	183.33
**ENPP5**	melanoma	0	9	0	1	10	180
**ENPP5**	head and neck cancer	1	2	0	1	4	175
**ENPP5**	renal cancer	0	9	2	1	12	166.67

**ENPP5**	endometrial cancer	0	7	5	0	12	158.33
**ENPP5**	urothelial cancer	0	7	3	1	11	154.55
**EPHA1**	colorectal cancer	6	6	0	0	12	250
**EPHA1**	endometrial cancer	1	11	0	0	12	208.33
**EPHA1**	carcinoid	0	4	0	0	4	200
**EPHA1**	liver cancer	0	12	0	0	12	200
**EPHA1**	pancreatic cancer	0	11	0	0	11	200
**EPHA1**	prostate cancer	0	11	0	0	11	200
**EPHA1**	testis cancer	0	12	0	0	12	200
**EPHA1**	thyroid cancer	0	4	0	0	4	200
**EPHA1**	urothelial cancer	0	11	1	0	12	191.67
**EPHA1**	breast cancer	1	8	3	0	12	183.33
**EPHA1**	ovarian cancer	1	8	3	0	12	183.33
**EPHA1**	stomach cancer	0	9	2	0	11	181.82
**EPHA1**	melanoma	0	9	3	0	12	175
**EPHA1**	renal cancer	0	8	3	0	11	172.73
**EPHA1**	head and neck cancer	0	2	1	0	3	166.67
**EPHA1**	skin cancer	0	7	5	0	12	158.33
**EPHA1**	cervical cancer	0	9	1	2	12	158.33
**EPHA3**	testis cancer	0	6	5	0	11	154.55
**EPHA4**	thyroid cancer	4	0	0	0	4	300
**ERBB2**	breast cancer	4	2	1	4	11	154.55
**ERBB4**	testis cancer	0	9	2	0	11	181.82
**ERMP1**	thyroid cancer	2	1	0	0	3	266.67
**ERMP1**	urothelial cancer	3	2	2	3	10	150
**ESAM**	colorectal cancer	1	10	0	0	11	209.09
**ESAM**	stomach cancer	1	10	0	0	11	209.09
**ESAM**	carcinoid	0	4	0	0	4	200
**ESAM**	endometrial cancer	0	12	0	0	12	200
**ESAM**	head and neck cancer	0	4	0	0	4	200
**ESAM**	liver cancer	0	12	0	0	12	200
**ESAM**	lung cancer	0	12	0	0	12	200
**ESAM**	melanoma	0	11	0	0	11	200
**ESAM**	prostate cancer	1	9	1	0	11	200
**ESAM**	testis cancer	0	11	0	0	11	200
**ESAM**	thyroid cancer	1	2	1	0	4	200
**ESAM**	breast cancer	3	6	2	1	12	191.67
**ESAM**	ovarian cancer	0	11	1	0	12	191.67
**ESAM**	pancreatic cancer	0	10	2	0	12	183.33
**ESAM**	urothelial cancer	0	11	0	1	12	183.33

**ESAM**	renal cancer	1	7	3	0	11	181.82
**ESAM**	lymphoma	0	10	1	1	12	175
**FAM171A1**	prostate cancer	0	10	2	0	12	183.33
**FAM171A1**	thyroid cancer	0	3	1	0	4	175
**FAM171A1**	breast cancer	0	7	5	0	12	158.33
**FAM171B**	urothelial cancer	1	7	4	0	12	175
**FAM171B**	melanoma	2	6	2	2	12	166.67
**FAM171B**	endometrial cancer	0	6	4	0	10	160
**FAM171B**	colorectal cancer	0	6	5	0	11	154.55
**FCAR**	thyroid cancer	0	3	1	0	4	175
**FFAR3**	pancreatic cancer	10	2	0	0	12	283.33
**FFAR3**	colorectal cancer	9	2	0	0	11	281.82
**FFAR3**	renal cancer	8	4	0	0	12	266.67
**FFAR3**	stomach cancer	6	3	0	0	9	266.67
**FFAR3**	ovarian cancer	5	3	3	1	12	200
**FFAR3**	endometrial cancer	3	6	2	1	12	191.67
**FFAR3**	melanoma	0	9	1	0	10	190
**FFAR3**	breast cancer	1	8	2	1	12	175
**FFAR3**	lung cancer	2	5	3	1	11	172.73
**FFAR3**	cervical cancer	1	7	2	2	12	158.33
**FFAR3**	lymphoma	0	7	3	1	11	154.55
**FFAR3**	carcinoid	0	3	0	1	4	150
**FFAR3**	head and neck cancer	0	3	0	1	4	150
**FFAR3**	thyroid cancer	0	2	2	0	4	150
**FFAR3**	urothelial cancer	1	5	2	2	10	150
**FGFR4**	colorectal cancer	0	8	2	1	11	163.64
**FGFRL1**	testis cancer	0	12	0	0	12	200
**FGFRL1**	colorectal cancer	0	7	3	1	11	154.55
**FGFRL1**	carcinoid	0	2	2	0	4	150
**FGFRL1**	thyroid cancer	0	2	2	0	4	150
**FLRT1**	thyroid cancer	1	3	0	0	4	225
**FLRT1**	testis cancer	0	8	0	0	8	200
**FLRT1**	liver cancer	0	11	1	0	12	191.67
**FLRT1**	ovarian cancer	0	8	3	0	11	172.73
**FLRT1**	pancreatic cancer	0	7	0	2	9	155.56
**FLRT1**	melanoma	0	7	3	1	11	154.55
**FLRT1**	head and neck cancer	0	3	0	1	4	150
**FLT1**	head and neck cancer	0	2	2	0	4	150
**FOLH1**	prostate cancer	7	0	0	0	7	300
**FRRS1**	colorectal cancer	4	7	0	0	11	236.36

**FRRS1**	stomach cancer	4	6	1	0	11	227.27
**FRRS1**	liver cancer	2	10	0	0	12	216.67
**FRRS1**	pancreatic cancer	3	7	0	1	11	209.09
**FRRS1**	melanoma	3	6	1	1	11	200
**FRRS1**	glioma	0	9	1	1	11	172.73
**FRRS1**	thyroid cancer	0	2	1	0	3	166.67
**FRRS1**	endometrial cancer	2	4	5	1	12	158.33
**FRRS1**	breast cancer	0	6	5	0	11	154.55
**GABBR1**	thyroid cancer	0	3	1	0	4	175
**GABBR1**	carcinoid	0	2	2	0	4	150
**GABRB1**	stomach cancer	4	6	0	0	10	240
**GABRB1**	breast cancer	4	6	2	0	12	216.67
**GABRB1**	ovarian cancer	3	8	1	0	12	216.67
**GABRB1**	head and neck cancer	0	2	0	0	2	200
**GABRB1**	pancreatic cancer	3	4	1	1	9	200
**GABRB1**	renal cancer	2	9	0	1	12	200
**GABRB1**	colorectal cancer	0	10	2	0	12	183.33
**GABRB1**	lung cancer	0	8	2	0	10	180
**GABRB1**	urothelial cancer	1	6	4	0	11	172.73
**GABRB1**	cervical cancer	0	7	4	0	11	163.64
**GABRB1**	endometrial cancer	0	6	4	0	10	160
**GALR2**	colorectal cancer	2	10	0	0	12	216.67
**GALR2**	carcinoid	0	4	0	0	4	200
**GALR2**	testis cancer	0	10	0	0	10	200
**GALR2**	thyroid cancer	0	4	0	0	4	200
**GALR2**	breast cancer	0	11	1	0	12	191.67
**GALR2**	ovarian cancer	0	9	2	0	11	181.82
**GALR2**	prostate cancer	0	8	2	1	11	163.64
**GALR2**	pancreatic cancer	1	5	3	1	10	160
**GIPR**	thyroid cancer	1	2	0	0	3	233.33
**GIPR**	prostate cancer	0	8	3	0	11	172.73
**GIPR**	breast cancer	0	8	3	1	12	158.33
**GIPR**	carcinoid	0	3	0	1	4	150
**GLRA4**	colorectal cancer	3	8	1	0	12	216.67
**GLRA4**	prostate cancer	0	10	1	0	11	190.91
**GLRA4**	pancreatic cancer	2	5	2	2	11	163.64
**GLRB**	glioma	6	3	1	0	10	250
**GP6**	glioma	0	9	2	0	11	181.82
**GPBAR1**	thyroid cancer	0	3	1	0	4	175
**GPC1**	thyroid cancer	0	3	0	1	4	150

**GPM6B**	endometrial cancer	6	1	1	4	12	175
**GPR143**	melanoma	8	0	1	3	12	208.33
**GPR143**	breast cancer	0	6	6	0	12	150
**GPR153**	glioma	4	2	6	0	12	183.33
**GPR34**	thyroid cancer	0	3	1	0	4	175
**GPR34**	melanoma	0	7	4	1	12	150
**GPR4**	colorectal cancer	6	2	1	1	10	230
**GPR4**	breast cancer	0	9	1	1	11	172.73
**GPR4**	ovarian cancer	0	8	2	2	12	150
**GPR4**	thyroid cancer	0	2	2	0	4	150
**GPR65**	prostate cancer	6	3	3	0	12	225
**GPR65**	pancreatic cancer	2	5	2	2	11	163.64
**GPR65**	colorectal cancer	1	6	3	2	12	150
**GPR82**	colorectal cancer	1	6	2	1	10	170
**GPRC5D**	lymphoma	8	2	0	2	12	233.33
**GPRC5D**	breast cancer	2	6	3	1	12	175
**GPRC5D**	carcinoid	0	3	1	0	4	175
**GPRC5D**	stomach cancer	0	7	4	0	11	163.64
**GPRC5D**	colorectal cancer	0	7	5	0	12	158.33
**GPRC5D**	liver cancer	0	7	3	1	11	154.55
**GRAMD1B**	head and neck cancer	1	1	1	1	4	150
**GRAMD1B**	stomach cancer	0	6	6	0	12	150
**GRIN1**	colorectal cancer	2	7	2	1	12	183.33
**GRM2**	head and neck cancer	0	3	1	0	4	175
**GRM2**	glioma	3	3	4	2	12	158.33
**GRM6**	glioma	3	5	4	0	12	191.67
**GSG1L**	head and neck cancer	1	1	1	1	4	150
**GSG1L**	testis cancer	4	2	2	4	12	150
**GYPC**	melanoma	0	12	0	0	12	200
**GYPC**	colorectal cancer	0	9	2	0	11	181.82
**GYPC**	testis cancer	1	7	2	1	11	172.73
**GYPC**	stomach cancer	0	7	4	0	11	163.64
**GYPC**	thyroid cancer	0	2	2	0	4	150
**HAVCR1**	breast cancer	2	8	2	0	12	200
**HAVCR1**	colorectal cancer	0	10	2	0	12	183.33
**HAVCR1**	carcinoid	0	3	1	0	4	175
**HAVCR1**	thyroid cancer	0	3	1	0	4	175
**HAVCR1**	prostate cancer	0	9	2	1	12	166.67
**HAVCR1**	ovarian cancer	0	7	5	0	12	158.33
**HAVCR1**	pancreatic cancer	0	7	5	0	12	158.33

**HAVCR1**	renal cancer	1	5	6	0	12	158.33
**HAVCR1**	stomach cancer	1	5	6	0	12	158.33
**HAVCR1**	head and neck cancer	0	2	2	0	4	150
**HCRTR1**	carcinoid	0	3	0	1	4	150
**HEG1**	thyroid cancer	2	2	0	0	4	250
**HEG1**	liver cancer	4	7	1	0	12	225
**HEG1**	endometrial cancer	3	8	1	0	12	216.67
**HEG1**	pancreatic cancer	2	4	4	1	11	163.64
**HEG1**	testis cancer	1	7	1	2	11	163.64
**HEPACAM**	glioma	2	6	1	3	12	158.33
**HEPH**	liver cancer	2	6	1	0	9	211.11
**HEPH**	pancreatic cancer	2	7	1	0	10	210
**HEPH**	colorectal cancer	2	9	1	0	12	208.33
**HEPH**	endometrial cancer	1	9	1	0	11	200
**HEPH**	thyroid cancer	0	4	0	0	4	200
**HEPH**	breast cancer	0	10	2	0	12	183.33
**HEPH**	ovarian cancer	0	10	2	0	12	183.33
**HEPH**	renal cancer	0	10	2	0	12	183.33
**HEPH**	testis cancer	0	9	2	0	11	181.82
**HEPH**	head and neck cancer	0	3	1	0	4	175
**HEPH**	stomach cancer	0	9	3	0	12	175
**HEPH**	urothelial cancer	0	7	4	0	11	163.64
**HEPH**	lung cancer	0	6	4	0	10	160
**HEPH**	prostate cancer	0	7	5	0	12	158.33
**HEPH**	cervical cancer	0	8	3	1	12	158.33
**HEPH**	lymphoma	0	7	4	1	12	150
**HLA-DMB**	lymphoma	1	8	1	2	12	166.67
**HLA-DOB**	lymphoma	9	2	0	1	12	258.33
**HRH1**	liver cancer	6	0	2	3	11	181.82
**HRH1**	colorectal cancer	0	6	6	0	12	150
**HTR2B**	breast cancer	8	3	0	1	12	250
**HTR2B**	pancreatic cancer	4	3	1	2	10	190
**HTR2B**	urothelial cancer	1	8	1	2	12	166.67
**HTR2B**	colorectal cancer	3	4	1	3	11	163.64
**HTR2B**	stomach cancer	4	2	1	4	11	154.55
**HTR3B**	testis cancer	2	6	1	0	9	211.11
**HTR3B**	melanoma	3	7	0	1	11	209.09
**HTR3B**	colorectal cancer	5	2	4	1	12	191.67
**HTR3B**	pancreatic cancer	2	8	0	2	12	183.33
**HTR3B**	stomach cancer	1	7	4	0	12	175

**HTR3B**	lung cancer	2	6	1	2	11	172.73
**HTR3B**	ovarian cancer	0	8	1	2	11	154.55
**HTR4**	prostate cancer	8	3	0	1	12	250
**HTR4**	thyroid cancer	1	2	0	1	4	175
**HTR4**	endometrial cancer	4	3	1	4	12	158.33
**HTR5A**	colorectal cancer	0	12	0	0	12	200
**HTR5A**	breast cancer	1	6	5	0	12	166.67
**HTR5A**	cervical cancer	0	9	1	2	12	158.33
**HTR5A**	thyroid cancer	0	3	0	1	4	150
**HTR6**	carcinoid	0	3	1	0	4	175
**HTR6**	thyroid cancer	0	3	1	0	4	175
**HTR7**	thyroid cancer	0	3	0	1	4	150
**ICOS**	testis cancer	0	8	4	0	12	166.67
**ICOS**	melanoma	0	8	1	2	11	154.55
**ICOS**	thyroid cancer	0	3	0	1	4	150
**IFNAR1**	thyroid cancer	0	3	0	0	3	200
**IFNAR1**	pancreatic cancer	0	10	0	2	12	166.67
**IGF2R**	thyroid cancer	2	1	0	0	3	266.67
**IGF2R**	prostate cancer	6	5	0	0	11	254.55
**IGF2R**	endometrial cancer	2	8	1	0	11	209.09
**IGF2R**	carcinoid	0	4	0	0	4	200
**IGF2R**	head and neck cancer	0	4	0	0	4	200
**IGF2R**	melanoma	3	6	1	1	11	200
**IGF2R**	colorectal cancer	1	10	0	1	12	191.67
**IGF2R**	liver cancer	2	6	3	0	11	190.91
**IGF2R**	testis cancer	1	8	2	0	11	190.91
**IGF2R**	stomach cancer	0	8	0	2	10	160
**IGF2R**	glioma	1	7	2	2	12	158.33
**IGF2R**	breast cancer	0	6	6	0	12	150
**IL10RB**	glioma	3	3	4	2	12	158.33
**IL1R1**	endometrial cancer	1	8	2	0	11	190.91
**IL1R1**	colorectal cancer	2	7	2	1	12	183.33
**IL1R1**	testis cancer	2	7	2	1	12	183.33
**IL1R1**	carcinoid	0	2	2	0	4	150
**IL1R2**	liver cancer	5	7	0	0	12	241.67
**IL1R2**	testis cancer	1	11	0	0	12	208.33
**IL1R2**	breast cancer	0	11	0	0	11	200
**IL1R2**	carcinoid	0	4	0	0	4	200
**IL1R2**	colorectal cancer	0	12	0	0	12	200
**IL1R2**	head and neck cancer	0	4	0	0	4	200

**IL1R2**	thyroid cancer	0	4	0	0	4	200
**IL1R2**	urothelial cancer	1	9	2	0	12	191.67
**IL1R2**	endometrial cancer	0	11	1	0	12	191.67
**IL1R2**	cervical cancer	0	11	0	1	12	183.33
**IL1R2**	pancreatic cancer	1	9	1	1	12	183.33
**IL1R2**	prostate cancer	0	11	0	1	12	183.33
**IL1R2**	glioma	0	9	3	0	12	175
**IL1R2**	renal cancer	0	9	2	1	12	166.67
**IL20RA**	prostate cancer	8	4	0	0	12	266.67
**IL20RA**	breast cancer	7	4	1	0	12	250
**IL20RA**	liver cancer	7	3	2	0	12	241.67
**IL20RA**	renal cancer	2	9	1	0	12	208.33
**IL20RA**	endometrial cancer	4	5	0	3	12	183.33
**IL20RA**	ovarian cancer	4	2	4	1	11	181.82
**IL20RA**	urothelial cancer	2	5	2	2	11	163.64
**IL20RA**	pancreatic cancer	3	3	3	2	11	163.64
**IL3RA**	glioma	2	8	1	0	11	209.09
**IL3RA**	colorectal cancer	0	11	0	0	11	200
**IL3RA**	head and neck cancer	0	4	0	0	4	200
**IL3RA**	ovarian cancer	0	11	0	0	11	200
**IL3RA**	pancreatic cancer	0	12	0	0	12	200
**IL3RA**	urothelial cancer	0	11	0	0	11	200
**IL3RA**	breast cancer	0	11	1	0	12	191.67
**IL3RA**	prostate cancer	0	11	1	0	12	191.67
**IL3RA**	testis cancer	0	11	1	0	12	191.67
**IL3RA**	lung cancer	0	10	2	0	12	183.33
**IL3RA**	carcinoid	0	3	1	0	4	175
**IL3RA**	endometrial cancer	0	9	3	0	12	175
**IL3RA**	liver cancer	0	8	4	0	12	166.67
**IL3RA**	melanoma	0	8	4	0	12	166.67
**IL3RA**	stomach cancer	0	8	2	1	11	163.64
**IL3RA**	cervical cancer	0	7	5	0	12	158.33
**IL3RA**	renal cancer	0	6	5	0	11	154.55
**IL3RA**	thyroid cancer	0	2	2	0	4	150
**ILDR1**	thyroid cancer	1	3	0	0	4	225
**ILDR1**	liver cancer	4	7	0	1	12	216.67
**ILDR1**	prostate cancer	1	3	2	0	6	183.33
**INSR**	carcinoid	0	3	1	0	4	175
**INSR**	thyroid cancer	0	3	1	0	4	175
**INSR**	colorectal cancer	0	8	3	0	11	172.73

**INSR**	breast cancer	0	6	4	0	10	160
**INSR**	liver cancer	0	8	3	1	12	158.33
**ITGA3**	thyroid cancer	3	1	0	0	4	275
**ITGA3**	urothelial cancer	9	2	0	1	12	258.33
**ITGA3**	pancreatic cancer	7	3	0	1	11	245.45
**ITGA3**	ovarian cancer	6	3	1	1	11	227.27
**ITGA3**	skin cancer	3	9	0	0	12	225
**ITGA3**	renal cancer	2	9	1	0	12	208.33
**ITGA3**	lung cancer	4	6	0	2	12	200
**ITGA3**	endometrial cancer	5	3	1	2	11	200
**ITGA3**	stomach cancer	3	5	4	0	12	191.67
**ITGA3**	head and neck cancer	1	2	0	1	4	175
**ITGA3**	cervical cancer	4	4	1	3	12	175
**ITGA6**	colorectal cancer	1	7	1	1	10	180
**ITGB5**	prostate cancer	2	5	0	0	7	228.57
**ITGB5**	head and neck cancer	1	2	1	0	4	200
**ITGB5**	endometrial cancer	1	9	0	1	11	190.91
**ITGB5**	glioma	3	4	4	0	11	190.91
**ITGB5**	thyroid cancer	1	1	2	0	4	175
**ITGB5**	liver cancer	2	4	3	1	10	170
**ITGB5**	breast cancer	0	9	2	1	12	166.67
**ITGB5**	melanoma	0	7	4	0	11	163.64
**ITGB5**	urothelial cancer	1	5	5	0	11	163.64
**ITGB5**	testis cancer	0	7	5	0	12	158.33
**ITGB5**	ovarian cancer	2	6	1	3	12	158.33
**ITGB6**	cervical cancer	2	7	3	0	12	191.67
**ITGB6**	urothelial cancer	4	3	1	4	12	158.33
**JAG1**	carcinoid	1	2	1	0	4	200
**JAG1**	stomach cancer	0	11	0	0	11	200
**JAG1**	thyroid cancer	0	4	0	0	4	200
**JAG1**	cervical cancer	0	10	2	0	12	183.33
**JAG1**	pancreatic cancer	0	10	2	0	12	183.33
**JAG1**	glioma	4	2	6	0	12	183.33
**JAG1**	liver cancer	0	9	2	0	11	181.82
**JAG1**	skin cancer	0	9	2	0	11	181.82
**JAG1**	colorectal cancer	0	10	1	1	12	175
**JAG1**	urothelial cancer	0	8	4	0	12	166.67
**JAG1**	endometrial cancer	0	5	5	0	10	150
**JAG1**	head and neck cancer	0	2	2	0	4	150
**JAM3**	glioma	9	2	0	0	11	281.82

**JAM3**	testis cancer	1	7	3	1	12	166.67
**JAM3**	carcinoid	0	2	1	0	3	166.67
**KCNMB2**	breast cancer	0	6	6	0	12	150
**KCNMB2**	prostate cancer	0	8	2	2	12	150
**KCNMB2**	thyroid cancer	0	2	2	0	4	150
**KISS1R**	thyroid cancer	2	1	0	0	3	266.67
**KISS1R**	endometrial cancer	5	7	0	0	12	241.67
**KISS1R**	melanoma	5	6	1	0	12	233.33
**KISS1R**	renal cancer	7	3	1	1	12	233.33
**KISS1R**	liver cancer	7	2	0	2	11	227.27
**KISS1R**	urothelial cancer	5	4	3	0	12	216.67
**KISS1R**	testis cancer	2	8	0	1	11	200
**KISS1R**	colorectal cancer	2	7	3	0	12	191.67
**KISS1R**	pancreatic cancer	2	7	0	2	11	181.82
**KISS1R**	stomach cancer	2	6	1	2	11	172.73
**KISS1R**	breast cancer	2	4	3	1	10	170
**KISS1R**	carcinoid	0	2	2	0	4	150
**KISS1R**	head and neck cancer	0	3	0	1	4	150
**KITLG**	prostate cancer	0	7	4	1	12	150
**LDLRAD3**	thyroid cancer	0	3	0	0	3	200
**LGR5**	colorectal cancer	2	7	3	0	12	191.67
**LGR5**	stomach cancer	2	7	1	2	12	175
**LGR5**	liver cancer	0	7	4	1	12	150
**LILRA4**	ovarian cancer	3	4	3	2	12	166.67
**LILRA4**	head and neck cancer	0	3	0	1	4	150
**LMAN2**	breast cancer	2	6	0	3	11	163.64
**LMBRD1**	carcinoid	1	2	0	1	4	175
**LMBRD1**	pancreatic cancer	0	9	3	0	12	175
**LMBRD1**	thyroid cancer	0	3	1	0	4	175
**LMBRD1**	colorectal cancer	0	7	3	1	11	154.55
**LMBRD1**	renal cancer	0	6	3	1	10	150
**LRFN3**	thyroid cancer	2	1	0	1	4	200
**LRFN3**	prostate cancer	0	7	4	1	12	150
**LRFN4**	glioma	6	2	2	1	11	218.18
**LRIG2**	renal cancer	0	12	0	0	12	200
**LRIG3**	endometrial cancer	0	12	0	0	12	200
**LRIG3**	thyroid cancer	0	4	0	0	4	200
**LRIG3**	urothelial cancer	0	12	0	0	12	200
**LRIG3**	ovarian cancer	0	11	1	0	12	191.67
**LRIG3**	skin cancer	0	10	1	0	11	190.91

**LRIG3**	breast cancer	0	10	2	0	12	183.33
**LRIG3**	melanoma	0	9	2	0	11	181.82
**LRIG3**	stomach cancer	0	9	2	0	11	181.82
**LRIG3**	glioma	2	5	5	0	12	175
**LRIG3**	carcinoid	0	3	1	0	4	175
**LRIG3**	cervical cancer	0	9	3	0	12	175
**LRIG3**	head and neck cancer	0	3	1	0	4	175
**LRIG3**	lung cancer	1	8	2	1	12	175
**LRIG3**	testis cancer	0	9	3	0	12	175
**LRIG3**	pancreatic cancer	0	9	2	1	12	166.67
**LRIG3**	prostate cancer	0	8	4	0	12	166.67
**LRIG3**	liver cancer	0	8	3	1	12	158.33
**LRP1B**	liver cancer	5	3	0	3	11	190.91
**LRP4**	colorectal cancer	1	10	1	0	12	200
**LRP4**	pancreatic cancer	0	12	0	0	12	200
**LRP4**	prostate cancer	0	10	0	0	10	200
**LRP4**	stomach cancer	0	10	2	0	12	183.33
**LRP4**	ovarian cancer	0	9	2	0	11	181.82
**LRP4**	liver cancer	0	10	1	1	12	175
**LRP4**	melanoma	0	9	2	1	12	166.67
**LRP4**	testis cancer	0	9	2	1	12	166.67
**LRP4**	urothelial cancer	0	8	4	0	12	166.67
**LRP4**	breast cancer	0	9	1	2	12	158.33
**LRP4**	endometrial cancer	0	6	3	1	10	150
**LRP4**	thyroid cancer	0	2	2	0	4	150
**LRP6**	carcinoid	0	3	0	0	3	200
**LRP6**	melanoma	1	8	3	0	12	183.33
**LRP6**	colorectal cancer	1	6	4	0	11	172.73
**LRP6**	ovarian cancer	0	7	4	0	11	163.64
**LRP6**	lung cancer	0	6	5	0	11	154.55
**LRP6**	thyroid cancer	0	2	2	0	4	150
**LRRC24**	prostate cancer	5	4	1	1	11	218.18
**LRRC24**	thyroid cancer	2	0	0	2	4	150
**LRRC25**	liver cancer	3	6	0	1	10	210
**LRRC25**	pancreatic cancer	2	4	2	0	8	200
**LRRC25**	thyroid cancer	1	1	1	0	3	200
**LRRC25**	endometrial cancer	1	6	5	0	12	166.67
**LRRC3B**	prostate cancer	9	1	0	0	10	290
**LRRC3B**	colorectal cancer	7	2	0	0	9	277.78
**LRRC3B**	carcinoid	3	1	0	0	4	275

**LRRC3B**	endometrial cancer	8	3	0	0	11	272.73
**LRRC3B**	breast cancer	7	4	0	0	11	263.64
**LRRC3B**	thyroid cancer	1	3	0	0	4	225
**LRRC3B**	cervical cancer	1	8	0	0	9	211.11
**LRRC3B**	glioma	1	10	0	0	11	209.09
**LRRC3B**	ovarian cancer	2	8	1	0	11	209.09
**LRRC3B**	lymphoma	2	7	1	1	11	190.91
**LRRC3B**	renal cancer	0	8	2	0	10	180
**LRRC3B**	liver cancer	1	6	3	1	11	163.64
**LRRC3B**	head and neck cancer	0	1	1	0	2	150
**LRRN2**	liver cancer	0	9	2	1	12	166.67
**LRRN4CL**	endometrial cancer	4	7	0	0	11	236.36
**LRRN4CL**	colorectal cancer	4	8	0	0	12	233.33
**LRRN4CL**	melanoma	1	10	0	0	11	209.09
**LRRN4CL**	breast cancer	3	6	3	0	12	200
**LRRN4CL**	carcinoid	1	2	0	1	4	175
**LRRN4CL**	head and neck cancer	0	3	1	0	4	175
**LRRN4CL**	urothelial cancer	1	7	3	1	12	166.67
**LRRN4CL**	pancreatic cancer	0	8	2	1	11	163.64
**LYPD5**	breast cancer	2	7	1	0	10	210
**LYPD5**	head and neck cancer	1	2	1	0	4	200
**LYPD5**	melanoma	1	6	1	0	8	200
**LYPD5**	glioma	0	9	2	0	11	181.82
**LYPD5**	colorectal cancer	2	5	2	1	10	180
**LYPD5**	urothelial cancer	2	6	3	1	12	175
**LYPD5**	cervical cancer	0	9	2	1	12	166.67
**LYPD5**	thyroid cancer	0	2	2	0	4	150
**MALRD1**	liver cancer	1	7	1	3	12	150
**MC3R**	thyroid cancer	0	3	1	0	4	175
**MC3R**	colorectal cancer	3	3	3	3	12	150
**MCOLN1**	thyroid cancer	2	1	0	0	3	266.67
**MCOLN1**	prostate cancer	6	2	2	0	10	240
**MCOLN1**	liver cancer	0	9	2	1	12	166.67
**MEGF8**	thyroid cancer	0	3	0	1	4	150
**MFSD12**	thyroid cancer	3	0	1	0	4	250
**MFSD12**	testis cancer	2	7	1	0	10	210
**MFSD12**	glioma	1	10	1	0	12	200
**MFSD12**	colorectal cancer	2	8	0	2	12	183.33
**MFSD12**	melanoma	1	9	1	1	12	183.33
**MFSD12**	breast cancer	1	8	1	1	11	181.82

**MFSD12**	carcinoid	0	3	1	0	4	175
**MFSD5**	head and neck cancer	1	0	0	0	1	300
**MFSD5**	thyroid cancer	0	2	2	0	4	150
**MMP14**	renal cancer	0	7	5	0	12	158.33
**MMP14**	head and neck cancer	0	3	0	1	4	150
**MPEG1**	colorectal cancer	0	7	2	0	9	177.78
**MPEG1**	head and neck cancer	0	3	1	0	4	175
**MPEG1**	pancreatic cancer	0	5	4	0	9	155.56
**MPEG1**	prostate cancer	0	6	5	0	11	154.55
**MPEG1**	breast cancer	0	6	6	0	12	150
**MPEG1**	renal cancer	0	8	2	2	12	150
**MPZL1**	prostate cancer	0	10	2	0	12	183.33
**MPZL1**	renal cancer	0	9	2	1	12	166.67
**MPZL1**	liver cancer	0	7	5	0	12	158.33
**MPZL2**	pancreatic cancer	0	7	1	0	8	187.5
**MPZL2**	prostate cancer	0	9	1	1	11	172.73
**MS4A1**	lymphoma	11	1	0	0	12	291.67
**MSLN**	pancreatic cancer	4	5	1	2	12	191.67
**MSLN**	ovarian cancer	3	6	1	2	12	183.33
**MUC15**	thyroid cancer	3	0	1	0	4	250
**MUC16**	ovarian cancer	5	5	0	1	11	227.27
**NCAM2**	renal cancer	2	4	4	1	11	163.64
**NEO1**	colorectal cancer	1	9	2	0	12	191.67
**NEO1**	breast cancer	0	10	2	0	12	183.33
**NEO1**	endometrial cancer	1	7	3	0	11	181.82
**NEO1**	thyroid cancer	0	3	1	0	4	175
**NEO1**	prostate cancer	0	6	4	0	10	160
**NEO1**	stomach cancer	0	6	6	0	12	150
**NETO2**	colorectal cancer	5	7	0	0	12	241.67
**NETO2**	endometrial cancer	3	8	0	0	11	227.27
**NETO2**	ovarian cancer	2	8	0	0	10	220
**NETO2**	prostate cancer	2	9	0	0	11	218.18
**NETO2**	pancreatic cancer	2	7	1	0	10	210
**NETO2**	stomach cancer	2	9	1	0	12	208.33
**NETO2**	glioma	0	12	0	0	12	200
**NETO2**	head and neck cancer	0	3	0	0	3	200
**NETO2**	thyroid cancer	1	2	1	0	4	200
**NETO2**	breast cancer	0	11	1	0	12	191.67
**NETO2**	lymphoma	0	11	1	0	12	191.67
**NETO2**	urothelial cancer	0	10	1	0	11	190.91

**NETO2**	cervical cancer	0	10	2	0	12	183.33
**NETO2**	melanoma	0	8	2	0	10	180
**NETO2**	carcinoid	0	3	1	0	4	175
**NETO2**	liver cancer	0	10	1	1	12	175
**NETO2**	lung cancer	0	8	4	0	12	166.67
**NETO2**	renal cancer	0	7	5	0	12	158.33
**NIPAL1**	colorectal cancer	1	6	3	1	11	163.64
**NLGN4X**	glioma	4	4	2	0	10	220
**NLGN4Y**	glioma	4	4	2	0	10	220
**NOTCH2**	testis cancer	0	11	1	0	12	191.67
**NOTCH2**	lung cancer	0	10	2	0	12	183.33
**NOTCH2**	melanoma	0	9	2	0	11	181.82
**NOTCH2**	ovarian cancer	0	9	3	0	12	175
**NOTCH2**	prostate cancer	0	8	4	0	12	166.67
**NOTCH2**	thyroid cancer	0	2	1	0	3	166.67
**NOTCH2**	skin cancer	0	7	4	0	11	163.64
**NOTCH2**	urothelial cancer	0	8	3	1	12	158.33
**NOTCH2**	cervical cancer	0	6	6	0	12	150
**NOTCH3**	carcinoid	2	1	1	0	4	225
**NOTCH3**	colorectal cancer	2	9	1	0	12	208.33
**NOTCH3**	thyroid cancer	0	4	0	0	4	200
**NOTCH3**	endometrial cancer	2	7	1	1	11	190.91
**NOTCH3**	breast cancer	3	6	1	2	12	183.33
**NOTCH3**	urothelial cancer	1	6	5	0	12	166.67
**NOTCH3**	skin cancer	2	5	3	2	12	158.33
**NOTCH3**	glioma	1	6	2	2	11	154.55
**NOTCH3**	cervical cancer	2	3	6	1	12	150
**NOTCH3**	head and neck cancer	0	2	2	0	4	150
**NOTCH3**	lymphoma	1	5	2	2	10	150
**NPY4R**	breast cancer	5	6	0	0	11	245.45
**NPY4R**	colorectal cancer	5	5	1	0	11	236.36
**NPY4R**	ovarian cancer	4	6	1	1	12	208.33
**NPY4R**	carcinoid	1	2	1	0	4	200
**NPY4R**	thyroid cancer	1	2	1	0	4	200
**NPY4R**	stomach cancer	3	6	1	2	12	183.33
**NPY4R**	pancreatic cancer	3	4	1	2	10	180
**NPY4R**	prostate cancer	0	7	5	0	12	158.33
**NPY4R**	cervical cancer	2	4	3	2	11	154.55
**NPY4R**	lung cancer	1	5	5	1	12	150
**NPY4R**	head and neck cancer	0	2	2	0	4	150

**NRCAM**	thyroid cancer	0	2	2	0	4	150
**NRG1**	lymphoma	3	4	5	0	12	183.33
**NRG2**	head and neck cancer	0	3	1	0	4	175
**NRP1**	ovarian cancer	2	9	1	0	12	208.33
**NRP1**	colorectal cancer	1	9	0	1	11	190.91
**NRP1**	stomach cancer	0	9	3	0	12	175
**NRP1**	testis cancer	0	8	3	1	12	158.33
**NRP1**	liver cancer	1	6	3	2	12	150
**OPALIN**	prostate cancer	0	9	0	0	9	200
**OPN3**	prostate cancer	0	6	5	0	11	154.55
**OPN3**	liver cancer	0	8	2	2	12	150
**OPN3**	thyroid cancer	0	2	2	0	4	150
**OPN5**	testis cancer	3	3	3	3	12	150
**OSMR**	endometrial cancer	2	8	1	0	11	209.09
**OSMR**	colorectal cancer	0	9	2	0	11	181.82
**OSMR**	cervical cancer	2	6	3	1	12	175
**OSMR**	skin cancer	0	9	1	1	11	172.73
**OSMR**	melanoma	2	6	0	3	11	163.64
**OSMR**	urothelial cancer	3	2	5	1	11	163.64
**OSMR**	stomach cancer	2	3	5	1	11	154.55
**OSMR**	lung cancer	1	6	3	2	12	150
**OSMR**	testis cancer	0	7	1	2	10	150
**OSMR**	thyroid cancer	0	3	0	1	4	150
**P2RX6**	prostate cancer	0	9	1	2	12	158.33
**P2RY11**	colorectal cancer	9	0	0	0	9	300
**P2RY11**	head and neck cancer	4	0	0	0	4	300
**P2RY11**	lymphoma	12	0	0	0	12	300
**P2RY11**	thyroid cancer	4	0	0	0	4	300
**P2RY11**	testis cancer	10	1	0	0	11	290.91
**P2RY11**	stomach cancer	9	1	0	0	10	290
**P2RY11**	carcinoid	3	1	0	0	4	275
**P2RY11**	melanoma	10	1	1	0	12	275
**P2RY11**	breast cancer	6	3	0	0	9	266.67
**P2RY11**	cervical cancer	8	3	0	1	12	250
**P2RY11**	endometrial cancer	6	3	1	0	10	250
**P2RY11**	ovarian cancer	7	3	0	1	11	245.45
**P2RY11**	skin cancer	7	3	0	1	11	245.45
**P2RY11**	prostate cancer	5	3	0	1	9	233.33
**P2RY11**	glioma	7	3	0	2	12	225
**P2RY11**	pancreatic cancer	5	3	2	1	11	209.09

**P2RY11**	urothelial cancer	3	6	2	0	11	209.09
**P2RY11**	lung cancer	2	6	1	2	11	172.73
**P2RY14**	head and neck cancer	3	0	0	0	3	300
**P2RY14**	ovarian cancer	5	5	0	0	10	250
**P2RY14**	colorectal cancer	5	7	0	0	12	241.67
**P2RY14**	liver cancer	3	7	1	0	11	218.18
**P2RY14**	breast cancer	2	10	0	0	12	216.67
**P2RY14**	stomach cancer	3	8	1	0	12	216.67
**P2RY14**	pancreatic cancer	2	7	1	0	10	210
**P2RY14**	skin cancer	2	7	1	0	10	210
**P2RY14**	melanoma	1	11	0	0	12	208.33
**P2RY14**	carcinoid	0	4	0	0	4	200
**P2RY14**	cervical cancer	3	6	3	0	12	200
**P2RY14**	thyroid cancer	0	4	0	0	4	200
**P2RY14**	endometrial cancer	2	7	1	1	11	190.91
**P2RY14**	lung cancer	2	7	0	2	11	181.82
**P2RY14**	prostate cancer	0	8	1	1	10	170
**P2RY14**	urothelial cancer	1	7	1	2	11	163.64
**PCDH19**	carcinoid	0	3	0	1	4	150
**PCDH7**	colorectal cancer	8	4	0	0	12	266.67
**PCDH7**	melanoma	3	7	1	0	11	218.18
**PCDH7**	ovarian cancer	4	5	3	0	12	208.33
**PCDH7**	pancreatic cancer	3	8	0	1	12	208.33
**PCDH7**	stomach cancer	3	6	1	1	11	200
**PCDH7**	lymphoma	0	7	5	0	12	158.33
**PCDHA12**	glioma	0	8	2	2	12	150
**PCDHA5**	carcinoid	0	4	0	0	4	200
**PCDHB10**	carcinoid	1	3	0	0	4	225
**PCDHB10**	liver cancer	0	10	0	0	10	200
**PCDHB10**	pancreatic cancer	2	5	2	0	9	200
**PCDHB10**	testis cancer	0	9	2	0	11	181.82
**PCDHB10**	endometrial cancer	0	9	3	0	12	175
**PCDHB10**	head and neck cancer	0	3	1	0	4	175
**PCDHB10**	thyroid cancer	1	1	2	0	4	175
**PCDHB10**	urothelial cancer	0	7	3	0	10	170
**PCDHB10**	stomach cancer	0	6	4	0	10	160
**PCDHB10**	colorectal cancer	0	6	3	1	10	150
**PCDHB5**	lymphoma	8	2	0	2	12	233.33
**PCDHB7**	ovarian cancer	3	6	2	0	11	209.09
**PCDHB7**	head and neck cancer	0	4	0	0	4	200

**PCDHB7**	skin cancer	1	10	1	0	12	200
**PCDHB7**	stomach cancer	1	8	3	0	12	183.33
**PCDHB7**	cervical cancer	1	8	1	1	11	181.82
**PCDHB7**	lung cancer	1	8	1	1	11	181.82
**PCDHB7**	breast cancer	1	6	1	1	9	177.78
**PCDHB7**	thyroid cancer	0	3	1	0	4	175
**PCDHB7**	glioma	0	8	3	0	11	172.73
**PCDHB7**	carcinoid	0	3	0	1	4	150
**PCDHGA11**	glioma	10	2	0	0	12	283.33
**PCDHGA11**	melanoma	6	5	1	0	12	241.67
**PCDHGA11**	carcinoid	1	1	2	0	4	175
**PCDHGA11**	head and neck cancer	0	3	1	0	4	175
**PCDHGA11**	prostate cancer	1	4	7	0	12	150
**PCDHGA11**	thyroid cancer	0	2	2	0	4	150
**PCDHGA12**	glioma	10	2	0	0	12	283.33
**PCDHGA12**	melanoma	6	5	1	0	12	241.67
**PCDHGA12**	carcinoid	1	1	2	0	4	175
**PCDHGA12**	head and neck cancer	0	3	1	0	4	175
**PCDHGA12**	prostate cancer	1	4	7	0	12	150
**PCDHGA12**	thyroid cancer	0	2	2	0	4	150
**PCDHGA2**	glioma	10	2	0	0	12	283.33
**PCDHGA2**	melanoma	6	5	1	0	12	241.67
**PCDHGA2**	carcinoid	1	1	2	0	4	175
**PCDHGA2**	head and neck cancer	0	3	1	0	4	175
**PCDHGA2**	prostate cancer	1	4	7	0	12	150
**PCDHGA2**	thyroid cancer	0	2	2	0	4	150
**PCDHGA5**	glioma	10	2	0	0	12	283.33
**PCDHGA5**	melanoma	6	5	1	0	12	241.67
**PCDHGA5**	carcinoid	1	1	2	0	4	175
**PCDHGA5**	head and neck cancer	0	3	1	0	4	175
**PCDHGA5**	prostate cancer	1	4	7	0	12	150
**PCDHGA5**	thyroid cancer	0	2	2	0	4	150
**PCDHGA6**	glioma	10	2	0	0	12	283.33
**PCDHGA6**	melanoma	6	5	1	0	12	241.67
**PCDHGA6**	carcinoid	1	1	2	0	4	175
**PCDHGA6**	head and neck cancer	0	3	1	0	4	175
**PCDHGA6**	prostate cancer	1	4	7	0	12	150
**PCDHGA6**	thyroid cancer	0	2	2	0	4	150
**PCDHGB7**	glioma	10	2	0	0	12	283.33
**PCDHGB7**	melanoma	6	5	1	0	12	241.67

**PCDHGB7**	carcinoid	1	1	2	0	4	175
**PCDHGB7**	head and neck cancer	0	3	1	0	4	175
**PCDHGB7**	prostate cancer	1	4	7	0	12	150
**PCDHGB7**	thyroid cancer	0	2	2	0	4	150
**PCDHGC3**	glioma	10	2	0	0	12	283.33
**PCDHGC3**	melanoma	6	5	1	0	12	241.67
**PCDHGC3**	carcinoid	1	1	2	0	4	175
**PCDHGC3**	head and neck cancer	0	3	1	0	4	175
**PCDHGC3**	prostate cancer	1	4	7	0	12	150
**PCDHGC3**	thyroid cancer	0	2	2	0	4	150
**PLXNA2**	lymphoma	9	2	1	0	12	266.67
**PLXNA2**	colorectal cancer	5	6	1	0	12	233.33
**PLXNA2**	stomach cancer	0	7	4	0	11	163.64
**PLXNA2**	endometrial cancer	2	2	7	0	11	154.55
**PLXNA2**	ovarian cancer	0	6	3	1	10	150
**PLXNA2**	urothelial cancer	0	7	4	1	12	150
**PQLC2**	testis cancer	0	6	3	1	10	150
**PRIMA1**	thyroid cancer	0	4	0	0	4	200
**PROM1**	colorectal cancer	10	2	0	0	12	283.33
**PROM1**	endometrial cancer	10	0	1	1	12	258.33
**PROM1**	pancreatic cancer	8	1	2	1	12	233.33
**PROM1**	prostate cancer	1	9	1	1	12	183.33
**PROM1**	stomach cancer	5	3	0	4	12	175
**PROM1**	testis cancer	3	5	1	3	12	166.67
**PROM1**	ovarian cancer	3	3	3	3	12	150
**PTGER3**	cervical cancer	4	3	0	4	11	163.64
**PTGFRN**	head and neck cancer	1	2	1	0	4	200
**PTGFRN**	prostate cancer	2	6	3	0	11	190.91
**PTGFRN**	endometrial cancer	2	5	2	1	10	180
**PTGFRN**	urothelial cancer	2	6	1	2	11	172.73
**PTGFRN**	skin cancer	0	7	3	1	11	154.55
**PTGFRN**	cervical cancer	4	2	2	4	12	150
**PTGFRN**	thyroid cancer	0	2	2	0	4	150
**PTH1R**	urothelial cancer	9	2	0	0	11	281.82
**PTH1R**	thyroid cancer	2	1	1	0	4	225
**PTH1R**	breast cancer	6	0	5	0	11	209.09
**PTH1R**	pancreatic cancer	4	2	3	2	11	172.73
**PTK7**	endometrial cancer	0	11	0	0	11	200
**PTK7**	colorectal cancer	1	7	2	0	10	190
**PTK7**	prostate cancer	0	9	1	0	10	190

**PTK7**	breast cancer	0	8	4	0	12	166.67
**PTK7**	thyroid cancer	0	2	1	0	3	166.67
**PTK7**	testis cancer	0	5	4	0	9	155.56
**PTK7**	skin cancer	0	6	5	0	11	154.55
**PTK7**	head and neck cancer	0	3	0	1	4	150
**PTPRJ**	carcinoid	0	4	0	0	4	200
**PTPRN**	carcinoid	1	1	1	1	4	150
**PTPRN2**	carcinoid	3	0	0	1	4	225
**PTPRR**	prostate cancer	3	6	2	0	11	209.09
**PTPRR**	renal cancer	1	11	0	0	12	208.33
**PTPRR**	thyroid cancer	1	1	1	0	3	200
**PTPRR**	liver cancer	2	6	3	0	11	190.91
**PTPRR**	pancreatic cancer	1	9	0	2	12	175
**PTPRR**	melanoma	1	6	5	0	12	166.67
**PTPRR**	glioma	1	6	4	1	12	158.33
**PTPRT**	carcinoid	1	1	1	1	4	150
**PTPRU**	endometrial cancer	2	9	0	0	11	218.18
**PTPRU**	breast cancer	2	9	1	0	12	208.33
**PTPRU**	liver cancer	2	9	1	0	12	208.33
**PTPRU**	ovarian cancer	2	9	1	0	12	208.33
**PTPRU**	carcinoid	1	2	1	0	4	200
**PTPRU**	glioma	2	7	2	0	11	200
**PTPRU**	head and neck cancer	1	2	1	0	4	200
**PTPRU**	prostate cancer	1	10	1	0	12	200
**PTPRU**	testis cancer	3	6	3	0	12	200
**PTPRU**	colorectal cancer	2	7	3	0	12	191.67
**PTPRU**	lung cancer	2	7	3	0	12	191.67
**PTPRU**	urothelial cancer	0	11	1	0	12	191.67
**PTPRU**	pancreatic cancer	1	8	2	0	11	190.91
**PTPRU**	cervical cancer	3	4	5	0	12	183.33
**PTPRU**	lymphoma	2	6	2	1	11	181.82
**PTPRU**	renal cancer	0	8	4	0	12	166.67
**PTPRU**	thyroid cancer	0	2	1	0	3	166.67
**PTPRU**	melanoma	1	5	5	0	11	163.64
**PTPRU**	skin cancer	1	5	6	0	12	158.33
**PTPRU**	stomach cancer	0	7	5	0	12	158.33
**PTPRZ1**	glioma	4	3	2	2	11	181.82
**QRFPR**	colorectal cancer	7	4	0	1	12	241.67
**QRFPR**	prostate cancer	1	6	3	2	12	150
**QRFPR**	thyroid cancer	0	3	0	1	4	150

**RNF167**	melanoma	1	5	2	2	10	150
**ROBO1**	glioma	3	9	0	0	12	225
**ROBO1**	ovarian cancer	1	11	0	0	12	208.33
**ROBO1**	carcinoid	0	4	0	0	4	200
**ROBO1**	cervical cancer	0	11	0	0	11	200
**ROBO1**	head and neck cancer	0	4	0	0	4	200
**ROBO1**	melanoma	0	12	0	0	12	200
**ROBO1**	skin cancer	0	12	0	0	12	200
**ROBO1**	stomach cancer	0	10	0	0	10	200
**ROBO1**	thyroid cancer	0	4	0	0	4	200
**ROBO1**	breast cancer	1	9	2	0	12	191.67
**ROBO1**	endometrial cancer	0	11	1	0	12	191.67
**ROBO1**	lung cancer	0	11	1	0	12	191.67
**ROBO1**	pancreatic cancer	0	9	1	0	10	190
**ROBO1**	prostate cancer	0	10	2	0	12	183.33
**ROBO1**	colorectal cancer	0	10	0	1	11	181.82
**ROBO1**	renal cancer	0	9	3	0	12	175
**ROBO1**	liver cancer	1	6	3	1	11	163.64
**ROBO1**	testis cancer	1	6	3	1	11	163.64
**ROR2**	pancreatic cancer	6	3	0	0	9	266.67
**ROR2**	ovarian cancer	7	4	0	0	11	263.64
**ROR2**	colorectal cancer	7	5	0	0	12	258.33
**ROR2**	cervical cancer	4	8	0	0	12	233.33
**ROR2**	skin cancer	4	8	0	0	12	233.33
**ROR2**	head and neck cancer	1	3	0	0	4	225
**ROR2**	stomach cancer	5	6	0	1	12	225
**ROR2**	thyroid cancer	1	3	0	0	4	225
**ROR2**	breast cancer	2	9	0	0	11	218.18
**ROR2**	melanoma	4	6	2	0	12	216.67
**ROR2**	urothelial cancer	3	8	1	0	12	216.67
**ROR2**	endometrial cancer	2	9	1	0	12	208.33
**ROR2**	liver cancer	6	3	1	2	12	208.33
**ROR2**	prostate cancer	0	11	0	0	11	200
**ROR2**	lymphoma	0	11	1	0	12	191.67
**ROR2**	testis cancer	3	4	1	2	10	180
**ROR2**	carcinoid	0	3	1	0	4	175
**ROR2**	lung cancer	2	6	1	2	11	172.73
**ROR2**	glioma	0	8	3	1	12	158.33
**RXFP1**	skin cancer	0	9	2	0	11	181.82
**SCN3A**	colorectal cancer	0	9	3	0	12	175

**SCN3A**	prostate cancer	2	6	3	1	12	175
**SCN3A**	head and neck cancer	0	2	2	0	4	150
**SCNN1A**	thyroid cancer	0	4	0	0	4	200
**SCNN1A**	colorectal cancer	0	11	1	0	12	191.67
**SCNN1A**	head and neck cancer	0	3	1	0	4	175
**SCNN1A**	breast cancer	1	6	5	0	12	166.67
**SCNN1A**	melanoma	0	9	2	1	12	166.67
**SCNN1A**	endometrial cancer	0	8	3	1	12	158.33
**SCNN1A**	ovarian cancer	1	6	2	2	11	154.55
**SCNN1A**	prostate cancer	0	8	2	2	12	150
**SDK1**	stomach cancer	0	10	0	0	10	200
**SDK1**	carcinoid	0	3	1	0	4	175
**SDK1**	head and neck cancer	0	3	1	0	4	175
**SDK1**	thyroid cancer	0	3	1	0	4	175
**SDK1**	testis cancer	0	8	3	0	11	172.73
**SDK1**	endometrial cancer	0	8	1	1	10	170
**SDK1**	ovarian cancer	0	9	2	1	12	166.67
**SDK1**	pancreatic cancer	0	7	4	1	12	150
**SEMA7A**	renal cancer	2	9	0	0	11	218.18
**SEMA7A**	liver cancer	2	8	1	1	12	191.67
**SEMA7A**	pancreatic cancer	1	5	3	0	9	177.78
**SEZ6L2**	carcinoid	0	4	0	0	4	200
**SGCA**	renal cancer	0	7	5	0	12	158.33
**SIDT2**	thyroid cancer	0	3	1	0	4	175
**SIDT2**	melanoma	0	6	4	0	10	160
**SIDT2**	lung cancer	0	7	5	0	12	158.33
**SIGLEC10**	lymphoma	4	5	0	3	12	183.33
**SIGLEC8**	ovarian cancer	1	9	2	0	12	191.67
**SIGLEC8**	stomach cancer	1	7	3	0	11	181.82
**SIGLEC8**	pancreatic cancer	0	5	4	0	9	155.56
**SIGLEC8**	endometrial cancer	0	6	6	0	12	150
**SIGLEC8**	liver cancer	0	6	6	0	12	150
**SIRPA**	glioma	7	2	0	1	10	250
**SIRPA**	lymphoma	4	8	0	0	12	233.33
**SIRPA**	head and neck cancer	1	3	0	0	4	225
**SIRPA**	colorectal cancer	5	3	1	1	10	220
**SIRPA**	melanoma	2	7	2	0	11	200
**SIRPA**	carcinoid	2	0	1	1	4	175
**SIRPA**	renal cancer	2	4	3	2	11	154.55
**SIRPB1**	colorectal cancer	1	5	5	0	11	163.64

**SIT1**	lymphoma	5	3	3	0	11	218.18
**SIT1**	stomach cancer	0	7	1	2	10	150
**SLAMF9**	colorectal cancer	0	10	0	1	11	181.82
**SLC10A1**	thyroid cancer	1	3	0	0	4	225
**SLC10A1**	prostate cancer	1	9	2	0	12	191.67
**SLC10A6**	head and neck cancer	1	1	1	1	4	150
**SLC11A2**	pancreatic cancer	5	3	2	0	10	230
**SLC11A2**	thyroid cancer	0	3	1	0	4	175
**SLC11A2**	stomach cancer	0	5	4	0	9	155.56
**SLC12A6**	carcinoid	0	4	0	0	4	200
**SLC12A6**	prostate cancer	0	12	0	0	12	200
**SLC12A6**	thyroid cancer	0	3	1	0	4	175
**SLC12A6**	testis cancer	0	8	1	2	11	154.55
**SLC12A9**	carcinoid	1	2	1	0	4	200
**SLC12A9**	melanoma	3	5	3	1	12	183.33
**SLC12A9**	thyroid cancer	0	3	1	0	4	175
**SLC12A9**	glioma	3	2	6	0	11	172.73
**SLC12A9**	pancreatic cancer	1	5	6	0	12	158.33
**SLC12A9**	prostate cancer	1	5	6	0	12	158.33
**SLC12A9**	ovarian cancer	1	3	5	0	9	155.56
**SLC12A9**	breast cancer	1	4	3	1	9	155.56
**SLC12A9**	colorectal cancer	0	5	5	0	10	150
**SLC15A1**	testis cancer	4	5	1	0	10	230
**SLC15A1**	thyroid cancer	1	3	0	0	4	225
**SLC15A1**	colorectal cancer	0	12	0	0	12	200
**SLC15A1**	endometrial cancer	0	11	0	0	11	200
**SLC15A1**	head and neck cancer	0	4	0	0	4	200
**SLC15A1**	breast cancer	0	11	1	0	12	191.67
**SLC15A1**	melanoma	0	10	1	0	11	190.91
**SLC15A1**	liver cancer	1	6	2	0	9	188.89
**SLC15A1**	renal cancer	2	5	4	0	11	181.82
**SLC15A1**	stomach cancer	0	9	2	0	11	181.82
**SLC15A1**	cervical cancer	0	8	3	0	11	172.73
**SLC15A1**	ovarian cancer	0	8	3	0	11	172.73
**SLC15A1**	prostate cancer	0	8	3	0	11	172.73
**SLC15A1**	carcinoid	0	2	1	0	3	166.67
**SLC15A1**	lymphoma	0	8	4	0	12	166.67
**SLC15A1**	lung cancer	0	7	5	0	12	158.33
**SLC15A1**	urothelial cancer	0	6	5	0	11	154.55
**SLC15A4**	testis cancer	4	6	1	0	11	227.27

**SLC22A16**	stomach cancer	0	6	3	0	9	166.67
**SLC22A16**	carcinoid	0	2	2	0	4	150
**SLC22A17**	renal cancer	5	5	2	0	12	225
**SLC22A17**	glioma	2	8	1	0	11	209.09
**SLC22A17**	testis cancer	2	3	7	0	12	158.33
**SLC22A17**	carcinoid	0	2	2	0	4	150
**SLC22A17**	cervical cancer	1	4	4	1	10	150
**SLC22A17**	head and neck cancer	1	0	3	0	4	150
**SLC22A2**	glioma	5	1	0	3	9	188.89
**SLC23A2**	thyroid cancer	1	3	0	0	4	225
**SLC23A2**	urothelial cancer	3	5	3	1	12	183.33
**SLC26A6**	colorectal cancer	0	7	3	1	11	154.55
**SLC26A6**	endometrial cancer	0	7	4	1	12	150
**SLC26A6**	head and neck cancer	0	2	2	0	4	150
**SLC2A14**	testis cancer	5	1	1	5	12	150
**SLC30A1**	endometrial cancer	2	7	2	1	12	183.33
**SLC30A1**	head and neck cancer	0	1	1	0	2	150
**SLC30A1**	liver cancer	1	5	2	2	10	150
**SLC37A2**	prostate cancer	9	2	0	0	11	281.82
**SLC37A2**	liver cancer	0	9	2	0	11	181.82
**SLC37A2**	endometrial cancer	0	10	1	1	12	175
**SLC37A2**	thyroid cancer	0	3	1	0	4	175
**SLC37A2**	colorectal cancer	0	7	3	0	10	170
**SLC37A2**	melanoma	1	5	2	1	9	166.67
**SLC37A2**	pancreatic cancer	0	5	5	0	10	150
**SLC38A1**	renal cancer	2	6	0	3	11	163.64
**SLC38A1**	liver cancer	4	3	1	4	12	158.33
**SLC38A1**	colorectal cancer	1	6	3	2	12	150
**SLC38A5**	carcinoid	0	2	1	0	3	166.67
**SLC39A10**	thyroid cancer	3	1	0	0	4	275
**SLC39A10**	breast cancer	7	5	0	0	12	258.33
**SLC39A10**	urothelial cancer	7	0	1	2	10	220
**SLC39A10**	pancreatic cancer	6	3	1	2	12	208.33
**SLC39A10**	lung cancer	5	4	0	3	12	191.67
**SLC39A10**	ovarian cancer	4	5	0	3	12	183.33
**SLC39A6**	breast cancer	3	7	2	0	12	208.33
**SLC39A6**	prostate cancer	1	8	2	0	11	190.91
**SLC39A6**	head and neck cancer	1	1	2	0	4	175
**SLC39A6**	colorectal cancer	0	8	4	0	12	166.67
**SLC39A6**	testis cancer	1	5	3	1	10	160

**SLC43A3**	colorectal cancer	0	6	6	0	12	150
**SLC44A3**	head and neck cancer	0	3	1	0	4	175
**SLC44A4**	prostate cancer	1	8	2	0	11	190.91
**SLC45A4**	pancreatic cancer	2	6	1	2	11	172.73
**SLC4A5**	prostate cancer	0	9	3	0	12	175
**SLC4A5**	breast cancer	0	9	1	1	11	172.73
**SLC4A5**	thyroid cancer	0	2	1	0	3	166.67
**SLC52A3**	head and neck cancer	1	2	1	0	4	200
**SLC52A3**	colorectal cancer	0	8	3	1	12	158.33
**SLC6A2**	thyroid cancer	0	3	0	1	4	150
**SLC6A6**	prostate cancer	1	7	0	1	9	188.89
**SLC6A6**	colorectal cancer	0	7	2	0	9	177.78
**SLC6A9**	head and neck cancer	0	3	0	0	3	200
**SLC6A9**	thyroid cancer	0	3	0	0	3	200
**SLC6A9**	colorectal cancer	2	6	3	0	11	190.91
**SLC6A9**	carcinoid	1	2	0	1	4	175
**SLC6A9**	melanoma	0	5	3	0	8	162.5
**SLC7A3**	urothelial cancer	6	4	0	1	11	236.36
**SLC7A3**	skin cancer	2	8	0	0	10	220
**SLC7A3**	pancreatic cancer	3	8	1	0	12	216.67
**SLC7A3**	breast cancer	2	9	1	0	12	208.33
**SLC7A3**	cervical cancer	1	10	1	0	12	200
**SLC7A3**	head and neck cancer	1	2	1	0	4	200
**SLC8B1**	colorectal cancer	8	2	0	1	11	254.55
**SLC8B1**	thyroid cancer	2	2	0	0	4	250
**SLC8B1**	lymphoma	7	3	2	0	12	241.67
**SLC8B1**	head and neck cancer	1	2	0	0	3	233.33
**SLC8B1**	cervical cancer	4	7	1	0	12	225
**SLC8B1**	testis cancer	4	6	0	1	11	218.18
**SLC8B1**	glioma	4	1	1	1	7	214.29
**SLC8B1**	breast cancer	3	4	3	0	10	200
**SLC8B1**	lung cancer	6	0	1	4	11	172.73
**SLC8B1**	stomach cancer	3	4	2	2	11	172.73
**SLC8B1**	skin cancer	3	3	2	2	10	170
**SLC8B1**	carcinoid	0	3	0	1	4	150
**SLC9A2**	liver cancer	2	6	3	1	12	175
**SLC9A2**	prostate cancer	2	3	4	1	10	160
**SLC9A2**	colorectal cancer	1	6	3	2	12	150
**SLC9A2**	testis cancer	0	7	4	1	12	150
**SLC9A2**	thyroid cancer	0	2	2	0	4	150

**SLC9A6**	glioma	0	7	4	1	12	150
**SLC9A6**	thyroid cancer	0	3	0	1	4	150
**SMO**	prostate cancer	0	12	0	0	12	200
**SMO**	melanoma	1	9	2	0	12	191.67
**SMO**	thyroid cancer	0	3	1	0	4	175
**SMO**	renal cancer	0	8	4	0	12	166.67
**SMO**	carcinoid	0	3	0	1	4	150
**SORCS1**	stomach cancer	4	6	0	1	11	218.18
**SORCS1**	lymphoma	5	3	2	2	12	191.67
**SORCS1**	thyroid cancer	1	2	0	1	4	175
**SORCS1**	pancreatic cancer	2	6	2	2	12	166.67
**SORCS1**	endometrial cancer	3	4	2	3	12	158.33
**SORCS1**	urothelial cancer	2	6	1	3	12	158.33
**SORCS1**	skin cancer	1	5	5	1	12	150
**SORCS1**	renal cancer	3	4	1	4	12	150
**SORL1**	thyroid cancer	0	3	0	0	3	200
**SORL1**	carcinoid	0	2	1	0	3	166.67
**SORL1**	breast cancer	0	6	3	1	10	150
**SORT1**	melanoma	5	5	0	1	11	227.27
**SORT1**	thyroid cancer	1	2	0	1	4	175
**SORT1**	glioma	3	3	2	2	10	170
**SPINT2**	prostate cancer	6	6	0	0	12	250
**SPINT2**	breast cancer	3	8	0	0	11	227.27
**SPINT2**	pancreatic cancer	4	7	1	0	12	225
**SPINT2**	colorectal cancer	2	10	0	0	12	216.67
**SPINT2**	stomach cancer	1	11	0	0	12	208.33
**SPINT2**	carcinoid	0	4	0	0	4	200
**SPINT2**	endometrial cancer	1	10	1	0	12	200
**SPINT2**	ovarian cancer	1	10	1	0	12	200
**SPINT2**	liver cancer	0	10	2	0	12	183.33
**SPINT2**	testis cancer	2	6	4	0	12	183.33
**SPINT2**	lung cancer	0	9	3	0	12	175
**SPINT2**	urothelial cancer	0	9	3	0	12	175
**SPINT2**	cervical cancer	0	8	4	0	12	166.67
**SPINT2**	thyroid cancer	0	2	1	0	3	166.67
**SSTR2**	liver cancer	0	9	2	1	12	166.67
**SSTR2**	carcinoid	0	2	2	0	4	150
**SSTR2**	stomach cancer	1	6	3	2	12	150
**SSTR2**	thyroid cancer	0	2	2	0	4	150
**STS**	thyroid cancer	0	3	1	0	4	175

**STS**	renal cancer	0	7	5	0	12	158.33
**STS**	glioma	0	6	5	0	11	154.55
**STS**	pancreatic cancer	0	7	4	1	12	150
**SUSD4**	colorectal cancer	0	8	3	1	12	158.33
**SUSD4**	thyroid cancer	0	2	2	0	4	150
**SV2A**	carcinoid	4	0	0	0	4	300
**SV2A**	glioma	3	2	2	3	10	150
**SV2B**	carcinoid	1	1	1	1	4	150
**SYP**	carcinoid	0	4	0	0	4	200
**TAS1R3**	prostate cancer	0	8	3	0	11	172.73
**TECTA**	head and neck cancer	1	1	1	1	4	150
**TGFA**	thyroid cancer	1	3	0	0	4	225
**TGFA**	pancreatic cancer	2	7	1	0	10	210
**TGFA**	liver cancer	2	9	0	1	12	200
**TGFA**	melanoma	2	8	0	1	11	200
**TGFA**	prostate cancer	0	11	0	0	11	200
**TGFA**	glioma	1	8	2	0	11	190.91
**TGFA**	renal cancer	1	7	3	1	12	166.67
**TGFA**	breast cancer	0	8	4	0	12	166.67
**TGFBR1**	stomach cancer	1	8	0	3	12	158.33
**THBD**	skin cancer	5	3	1	3	12	183.33
**TLR3**	renal cancer	2	9	1	0	12	208.33
**TLR3**	thyroid cancer	0	3	1	0	4	175
**TLR3**	pancreatic cancer	0	7	3	1	11	154.55
**TMCO3**	breast cancer	10	1	0	0	11	290.91
**TMCO3**	prostate cancer	10	1	0	0	11	290.91
**TMCO3**	endometrial cancer	10	2	0	0	12	283.33
**TMCO3**	pancreatic cancer	8	2	1	0	11	263.64
**TMCO3**	colorectal cancer	7	5	0	0	12	258.33
**TMCO3**	stomach cancer	7	2	2	0	11	245.45
**TMCO3**	carcinoid	1	2	0	0	3	233.33
**TMCO3**	ovarian cancer	5	5	2	0	12	225
**TMCO3**	skin cancer	4	3	2	1	10	200
**TMCO3**	glioma	2	8	2	0	12	200
**TMCO3**	head and neck cancer	1	2	1	0	4	200
**TMCO3**	cervical cancer	3	5	3	1	12	183.33
**TMCO3**	melanoma	1	8	1	1	11	181.82
**TMCO3**	lung cancer	5	2	1	3	11	181.82
**TMCO3**	thyroid cancer	1	1	0	1	3	166.67
**TMCO3**	urothelial cancer	2	6	0	3	11	163.64

**TMCO3**	liver cancer	4	2	1	4	11	154.55
**TMCO3**	renal cancer	3	3	2	3	11	154.55
**TMED7**	endometrial cancer	4	6	1	0	11	227.27
**TMED7**	carcinoid	1	2	1	0	4	200
**TMED7**	thyroid cancer	0	4	0	0	4	200
**TMED7**	liver cancer	1	8	3	0	12	183.33
**TMED7**	head and neck cancer	0	3	1	0	4	175
**TMED7**	prostate cancer	0	7	5	0	12	158.33
**TMED7**	breast cancer	0	6	5	0	11	154.55
**TMEFF2**	melanoma	1	8	2	1	12	175
**TMEFF2**	thyroid cancer	0	3	1	0	4	175
**TMEFF2**	prostate cancer	1	6	4	1	12	158.33
**TMEM108**	head and neck cancer	0	4	0	0	4	200
**TMEM108**	colorectal cancer	0	6	4	0	10	160
**TMEM154**	colorectal cancer	3	4	3	1	11	181.82
**TMEM161A**	carcinoid	2	1	1	0	4	225
**TMEM204**	thyroid cancer	2	1	0	1	4	200
**TMEM25**	liver cancer	0	10	2	0	12	183.33
**TMEM25**	ovarian cancer	0	6	5	0	11	154.55
**TMEM30A**	melanoma	7	4	0	0	11	263.64
**TMEM30A**	prostate cancer	0	10	0	0	10	200
**TNFRSF10C**	thyroid cancer	0	2	2	0	4	150
**TNFRSF13B**	thyroid cancer	3	1	0	0	4	275
**TNFRSF13B**	colorectal cancer	8	4	0	0	12	266.67
**TNFRSF13B**	lymphoma	7	2	0	2	11	227.27
**TNFRSF13B**	liver cancer	4	7	1	0	12	225
**TNFRSF13B**	testis cancer	3	7	2	0	12	208.33
**TNFRSF13B**	carcinoid	1	2	1	0	4	200
**TNFRSF13B**	breast cancer	0	11	1	0	12	191.67
**TNFRSF13B**	ovarian cancer	0	9	1	2	12	158.33
**TNFRSF13B**	stomach cancer	1	4	3	1	9	155.56
**TNFRSF13B**	melanoma	2	4	3	2	11	154.55
**TNFRSF13B**	head and neck cancer	1	1	1	1	4	150
**TNFRSF18**	lymphoma	3	5	1	3	12	166.67
**TNFRSF1A**	cervical cancer	1	7	4	0	12	175
**TNFRSF1A**	urothelial cancer	0	9	3	0	12	175
**TNFRSF1A**	breast cancer	2	6	2	2	12	166.67
**TNFRSF1A**	ovarian cancer	0	7	4	0	11	163.64
**TNFRSF1A**	testis cancer	0	7	4	0	11	163.64
**TNFRSF1A**	colorectal cancer	1	5	6	0	12	158.33

**TNFRSF1A**	stomach cancer	2	4	5	1	12	158.33
**TNFRSF1B**	renal cancer	4	6	2	0	12	216.67
**TNFRSF1B**	testis cancer	2	8	2	0	12	200
**TNFRSF1B**	liver cancer	1	8	2	0	11	190.91
**TNFRSF1B**	breast cancer	0	10	2	0	12	183.33
**TNFRSF1B**	prostate cancer	0	9	3	0	12	175
**TNFRSF1B**	thyroid cancer	1	1	2	0	4	175
**TNFRSF1B**	colorectal cancer	0	8	4	0	12	166.67
**TNFRSF1B**	urothelial cancer	0	8	4	0	12	166.67
**TNFRSF1B**	stomach cancer	0	7	4	0	11	163.64
**TNFRSF1B**	pancreatic cancer	0	7	5	0	12	158.33
**TNFRSF1B**	melanoma	0	8	3	1	12	158.33
**TNFRSF1B**	carcinoid	0	2	2	0	4	150
**TNFRSF1B**	head and neck cancer	0	2	2	0	4	150
**TNFRSF21**	breast cancer	0	10	2	0	12	183.33
**TNFRSF21**	prostate cancer	0	8	3	1	12	158.33
**TNFRSF21**	colorectal cancer	0	6	5	0	11	154.55
**TNFRSF21**	head and neck cancer	0	2	2	0	4	150
**TNFSF13B**	head and neck cancer	1	3	0	0	4	225
**TNFSF13B**	carcinoid	0	4	0	0	4	200
**TNFSF13B**	thyroid cancer	0	4	0	0	4	200
**TNFSF13B**	colorectal cancer	0	11	1	0	12	191.67
**TNFSF13B**	liver cancer	0	9	2	0	11	181.82
**TNFSF13B**	testis cancer	0	9	2	0	11	181.82
**TNFSF13B**	breast cancer	1	7	3	0	11	181.82
**TNFSF13B**	pancreatic cancer	0	9	3	0	12	175
**TNFSF13B**	stomach cancer	0	8	3	0	11	172.73
**TNFSF13B**	urothelial cancer	0	7	3	0	10	170
**TNFSF13B**	lymphoma	0	9	2	1	12	166.67
**TNFSF13B**	renal cancer	0	8	4	0	12	166.67
**TNFSF13B**	ovarian cancer	0	7	4	0	11	163.64
**TNFSF13B**	endometrial cancer	0	6	5	0	11	154.55
**TNFSF13B**	prostate cancer	0	6	5	0	11	154.55
**TPO**	thyroid cancer	1	2	0	1	4	175
**TRPV6**	melanoma	0	9	0	3	12	150
**TSPAN13**	renal cancer	1	10	1	0	12	200
**TSPAN13**	urothelial cancer	0	6	5	0	11	154.55
**TSPAN13**	carcinoid	0	2	2	0	4	150
**TSPAN15**	pancreatic cancer	3	6	0	3	12	175
**TSPAN2**	glioma	8	0	1	3	12	208.33

**TSPAN3**	colorectal cancer	6	4	1	1	12	225
**TSPAN3**	glioma	3	8	1	0	12	216.67
**TSPAN3**	pancreatic cancer	3	6	2	0	11	209.09
**TSPAN3**	thyroid cancer	0	4	0	0	4	200
**TSPAN31**	melanoma	2	6	3	0	11	190.91
**TSPAN9**	breast cancer	0	6	3	0	9	166.67
**TSPAN9**	thyroid cancer	0	2	2	0	4	150
**TXNDC15**	thyroid cancer	0	4	0	0	4	200
**TXNDC15**	breast cancer	0	10	2	0	12	183.33
**TXNDC15**	testis cancer	0	8	2	0	10	180
**TXNDC15**	prostate cancer	0	9	2	1	12	166.67
**TXNDC15**	head and neck cancer	0	2	2	0	4	150
**TXNDC15**	melanoma	0	8	2	2	12	150
**UBAC2**	colorectal cancer	1	8	2	1	12	175
**UGT8**	glioma	5	5	0	0	10	250
**UGT8**	stomach cancer	2	7	2	0	11	200
**UGT8**	colorectal cancer	1	8	2	1	12	175
**UGT8**	testis cancer	0	8	3	0	11	172.73
**UGT8**	thyroid cancer	1	1	0	1	3	166.67
**UPK1A**	thyroid cancer	2	1	0	0	3	266.67
**UPK1B**	urothelial cancer	5	1	3	2	11	181.82
**UPK1B**	endometrial cancer	2	4	4	2	12	150
**UPK3B**	renal cancer	10	2	0	0	12	283.33
**UPK3B**	urothelial cancer	3	8	0	1	12	208.33
**UPK3B**	head and neck cancer	0	3	0	0	3	200
**UPK3B**	endometrial cancer	1	6	3	0	10	180
**UPK3B**	ovarian cancer	2	5	4	1	12	166.67
**UPK3B**	testis cancer	0	7	2	1	10	160
**UPK3B**	melanoma	3	2	6	1	12	158.33
**UPK3B**	colorectal cancer	2	3	5	1	11	154.55
**UPK3B**	carcinoid	1	1	1	1	4	150
**VLDLR**	liver cancer	0	9	2	1	12	166.67
**VSTM4**	carcinoid	1	3	0	0	4	225
**VSTM4**	urothelial cancer	1	9	0	0	10	210
**VSTM4**	ovarian cancer	1	10	0	0	11	209.09
**VSTM4**	glioma	1	11	0	0	12	208.33
**VSTM4**	breast cancer	0	11	0	0	11	200
**VSTM4**	cervical cancer	0	11	0	0	11	200
**VSTM4**	head and neck cancer	0	4	0	0	4	200
**VSTM4**	melanoma	1	10	1	0	12	200

**VSTM4**	pancreatic cancer	0	9	0	0	9	200
**VSTM4**	skin cancer	0	12	0	0	12	200
**VSTM4**	testis cancer	0	11	0	0	11	200
**VSTM4**	thyroid cancer	0	4	0	0	4	200
**VSTM4**	lymphoma	0	11	1	0	12	191.67
**VSTM4**	colorectal cancer	0	10	1	0	11	190.91
**VSTM4**	stomach cancer	0	9	1	0	10	190
**VSTM4**	lung cancer	0	9	2	0	11	181.82
**VSTM4**	endometrial cancer	0	10	1	1	12	175
**VSTM4**	renal cancer	1	7	4	0	12	175
**VSTM4**	liver cancer	0	7	4	0	11	163.64
**ZDHHC11B**	head and neck cancer	0	1	1	0	2	150

Supplementary Table S3. Validation status for potential ADC targets (only green rows contain validated targets)

Gene name	Antibody Name	RNA consistency	Literature conformity	Verification of membrane localization
**ABCA2**	HPA042886	Mainly consistent with RNA expression data.	Partly consistent with gene/protein characterization data.	No
**ABCB1**	CAB001716	Mainly consistent with RNA expression data.	Partly consistent with extensive gene/protein characterization data.	Yes
**ABCB9**	HPA035114	Mainly not consistent with RNA expression data.	Partly consistent with gene/protein characterization data.	
**ABCC10**	HPA041607	Mainly not consistent with RNA expression data.	Partly consistent with gene/protein characterization data.	
**ABCC11**	HPA031981	Not consistent with RNA expression data.	Partly consistent with gene/protein characterization data.	
**ACVR1B**	HPA063761	Mainly not consistent with RNA expression data.	Partly consistent with extensive gene/protein characterization data.	
**ADAM15**	HPA011633	Mainly not consistent with RNA expression data.	Partly consistent with extensive gene/protein characterization data.	
**ADAM17**	HPA010738	Mainly consistent with RNA expression data.	Partly consistent with extensive gene/protein characterization data.	No
**ADAM23**	CAB033276	Mainly consistent with RNA expression data.	Partly consistent with gene/protein characterization data.	No
**ADAM33**	HPA067152	Mainly not consistent with RNA expression data.	Partly consistent with gene/protein characterization data.	
**ADAM8**	HPA064637	Mainly not consistent with RNA expression data.	Consistent with gene/protein characterization data.	
**ADCY2**	HPA038015	Mainly consistent with RNA expression data.	Partly consistent with gene/protein characterization data.	No
**ADCY3**	CAB010223	Mainly not consistent with RNA expression data.	Partly consistent with gene/protein characterization data.	
**ADCY6**	CAB018365	Mainly not consistent with RNA expression data.	Partly consistent with extensive gene/protein characterization data.	
**ADCY9**	HPA041328	Mainly consistent with RNA expression data.	Partly consistent with gene/protein characterization data.	No
**ALK**	HPA010694	Mainly not consistent with RNA expression data.	Partly consistent with extensive gene/protein characterization data.	
**ALPL**	HPA007105	Mainly not consistent with RNA expression data.	Partly consistent with extensive gene/protein characterization data.	
**ANO1**	HPA032148	Mainly consistent with RNA expression data.	Consistent with extensive gene/protein characterization data.	No
**ANO7**	HPA078464	Consistent with RNA expression data.	Consistent with extensive gene/protein characterization data.	Yes
**ANO9**	HPA039948	Mainly not consistent with RNA expression data.	No avaliable gene/protein characterization data.	
**AQP5**	HPA065008	Consistent with RNA expression data.	Consistent with extensive gene/protein characterization data.	Yes
**AQP8**	HPA046259	Mainly consistent with RNA expression data.	Partly consistent with gene/protein characterization data.	No
**ATP13A1**	HPA049717	Mainly not consistent with RNA expression data.	No avaliable gene/protein characterization data.	
**ATP1B3**	CAB020697	Mainly consistent with RNA expression data.	Partly consistent with extensive gene/protein characterization data.	No
**ATP2B2**	CAB005606	Mainly consistent with RNA expression data.	Consistent with extensive gene/protein characterization data.	Yes
**ATRN**	HPA008853	Mainly not consistent with RNA expression data.	Partly consistent with extensive gene/protein characterization data.	
**BACE1**	CAB016358	Mainly not consistent with RNA expression data.	Partly consistent with extensive gene/protein characterization data.	
**BACE2**	HPA035416	Mainly not consistent with RNA expression data.	Partly consistent with extensive gene/protein characterization data.	
**BAMBI**	HPA010866	Mainly not consistent with RNA expression data.	Partly consistent with gene/protein characterization data.	
**C11orf24**	HPA012748	Mainly not consistent with RNA expression data.	Partly consistent with gene/protein characterization data.	
**CALCR**	HPA061428	Mainly consistent with RNA expression data.	Consistent with extensive gene/protein characterization data.	No

**CCR2**	CAB003793	Mainly not consistent with RNA expression data.	Partly consistent with extensive gene/protein characterization data.	
**CCR3**	CAB003795	Mainly not consistent with RNA expression data.	Partly consistent with extensive gene/protein characterization data.	
**CCR9**	CAB006839	Mainly not consistent with RNA expression data.	Partly consistent with extensive gene/protein characterization data.	
**CD101**	HPA057763	Mainly not consistent with RNA expression data.	Partly consistent with extensive gene/protein characterization data.	
**CD19**	CAB016110	Mainly consistent with RNA expression data.	Consistent with extensive gene/protein characterization data.	Yes
**CD1B**	HPA021824	Mainly not consistent with RNA expression data.	Partly consistent with extensive gene/protein characterization data.	
**CD276**	HPA009285	Mainly consistent with RNA expression data.	Partly consistent with extensive gene/protein characterization data.	Yes
**CD33**	HPA035832	Mainly not consistent with RNA expression data.	Partly consistent with extensive gene/protein characterization data.	
**CD37**	CAB002492	Consistent with RNA expression data.	Consistent with extensive gene/protein characterization data.	No
**CD3D**	CAB013055	Mainly consistent with RNA expression data.	Consistent with extensive gene/protein characterization data.	No
**CD3G**	CAB017520	Mainly consistent with RNA expression data.	Partly consistent with extensive gene/protein characterization data.	No
**CD47**	HPA044659	Mainly not consistent with RNA expression data.	Partly consistent with extensive gene/protein characterization data.	
**CD53**	HPA047216	Mainly consistent with RNA expression data.	Partly consistent with extensive gene/protein characterization data.	No
**CD63**	HPA010088	Mainly consistent with RNA expression data.	Consistent with extensive gene/protein characterization data.	No
**CD74**	CAB002506	Mainly consistent with RNA expression data.	Consistent with extensive gene/protein characterization data.	No
**CD79A**	CAB000019	Consistent with RNA expression data.	Consistent with extensive gene/protein characterization data.	No
**CD79B**	HPA009178	Mainly consistent with RNA expression data.	Consistent with extensive gene/protein characterization data.	Yes
**CD83**	CAB002509	Mainly consistent with RNA expression data.	Consistent with extensive gene/protein characterization data.	No
**CD86**	CAB004319	Mainly consistent with RNA expression data.	Consistent with extensive gene/protein characterization data.	No
**CDCP1**	HPA010979	Mainly consistent with RNA expression data.	Partly consistent with extensive gene/protein characterization data.	Yes
**CDH11**	CAB013072	Mainly consistent with RNA expression data.	Consistent with extensive gene/protein characterization data.	No
**CDH18**	HPA014416	Mainly consistent with RNA expression data.	Partly consistent with gene/protein characterization data.	No
**CDH20**	HPA015490	Not consistent with RNA expression data.	No avaliable gene/protein characterization data.	
**CDH5**	CAB028366	Mainly not consistent with RNA expression data.	Partly consistent with gene/protein characterization data.	
**CDH8**	HPA014908	Mainly not consistent with RNA expression data.	Partly consistent with extensive gene/protein characterization data.	
**CELSR3**	HPA062866	Mainly not consistent with RNA expression data.	Partly consistent with gene/protein characterization data.	
**CHPT1**	HPA067643	Mainly not consistent with RNA expression data.	Partly consistent with extensive gene/protein characterization data.	
**CHRNA7**	HPA029422	Mainly consistent with RNA expression data.	Partly consistent with extensive gene/protein characterization data.	No
**CHRNB1**	HPA005822	Mainly not consistent with RNA expression data.	Partly consistent with extensive gene/protein characterization data.	
**CLCNKB**	HPA057717	Mainly consistent with RNA expression data.	Partly consistent with gene/protein characterization data.	No
**CLRN1**	HPA054636	Not consistent with RNA expression data.	Partly consistent with gene/protein characterization data.	
**CMKLR1**	HPA047865	Mainly not consistent with RNA expression data.	Not consistent with gene/protein characterization data.	
**CNR1**	HPA069945	Mainly consistent with RNA expression data.	Partly consistent with gene/protein characterization data.	Yes

**CNTN6**	HPA016645	Not consistent with RNA expression data.	Partly consistent with gene/protein characterization data.	
**CRIM1**	HPA000556	Mainly consistent with RNA expression data.	Partly consistent with gene/protein characterization data.	No
**CSF1R**	CAB008970	Mainly not consistent with RNA expression data.	Partly consistent with extensive gene/protein characterization data.	
**CSPG5**	HPA071779	Consistent with RNA expression data.	Consistent with extensive gene/protein characterization data.	No
**CXCR5**	HPA042432	Mainly consistent with RNA expression data.	Partly consistent with extensive gene/protein characterization data.	No
**DCBLD2**	HPA016909	Mainly consistent with RNA expression data.	Partly consistent with gene/protein characterization data.	No
**DSCAM**	HPA019324	Consistent with RNA expression data.	Consistent with extensive gene/protein characterization data.	No
**DUOXA1**	HPA041578	Mainly not consistent with RNA expression data.	Partly consistent with gene/protein characterization data.	
**EDNRA**	CAB018957	Mainly not consistent with RNA expression data.	Partly consistent with extensive gene/protein characterization data.	
**EDNRB**	HPA027546	Mainly consistent with RNA expression data.	Partly consistent with extensive gene/protein characterization data.	No
**EFNA3**	CAB010494	Mainly not consistent with RNA expression data.	Partly consistent with gene/protein characterization data.	
**EFNA4**	CAB021350	No internal RNA expression data available for correlation.	Consistent with extensive gene/protein characterization data.	
**ENPP5**	HPA016902	Mainly consistent with RNA expression data.	Partly consistent with gene/protein characterization data.	No
**EPHA1**	CAB026144	Mainly not consistent with RNA expression data.	Partly consistent with extensive gene/protein characterization data.	
**EPHA3**	CAB010462	Mainly not consistent with RNA expression data.	Partly consistent with extensive gene/protein characterization data.	
**EPHA4**	CAB028368	Mainly consistent with RNA expression data.	Partly consistent with extensive gene/protein characterization data.	No
**ERBB2**	CAB020416	Mainly consistent with RNA expression data.	Consistent with extensive gene/protein characterization data.	Yes
**ERBB4**	HPA012016	Mainly consistent with RNA expression data.	Partly consistent with extensive gene/protein characterization data.	No
**ERMP1**	HPA055582	Mainly not consistent with RNA expression data.	No avaliable gene/protein characterization data.	
**ESAM**	HPA056293	Mainly not consistent with RNA expression data.	Partly consistent with gene/protein characterization data.	
**FAM171A1**	HPA051345	Mainly consistent with RNA expression data.	No avaliable gene/protein characterization data.	
**FAM171B**	HPA010639	Mainly not consistent with RNA expression data.	No avaliable gene/protein characterization data.	
**FCAR**	HPA014050	Mainly consistent with RNA expression data.	Consistent with extensive gene/protein characterization data.	No
**FFAR3**	HPA044681	Mainly not consistent with RNA expression data.	Partly consistent with extensive gene/protein characterization data.	
**FGFR4**	HPA028251	Mainly not consistent with RNA expression data.	Partly consistent with extensive gene/protein characterization data.	
**FGFRL1**	CAB026019	Mainly not consistent with RNA expression data.	Partly consistent with extensive gene/protein characterization data.	
**FLRT1**	HPA054589	Mainly not consistent with RNA expression data.	Partly consistent with gene/protein characterization data.	
**FLT1**	HPA011740	Mainly consistent with RNA expression data.	Consistent with extensive gene/protein characterization data.	No
**FOLH1**	CAB001451	Consistent with RNA expression data.	Consistent with extensive gene/protein characterization data.	No
**FRRS1**	HPA017883	Mainly consistent with RNA expression data.	No avaliable gene/protein characterization data.	
**GABBR1**	HPA050483	Mainly not consistent with RNA expression data.	Partly consistent with extensive gene/protein characterization data.	
**GABRB1**	HPA051297	Not consistent with RNA expression data.	Partly consistent with gene/protein characterization data.	
**GALR2**	HPA044513	Mainly not consistent with RNA expression data.	Partly consistent with extensive gene/protein characterization data.	
**GIPR**	CAB022710	Mainly not consistent with RNA expression data.	Partly consistent with gene/protein characterization data.	

**GLRA4**	HPA044759	Not consistent with RNA expression data.	No avaliable gene/protein characterization data.	
**GLRB**	HPA052363	Mainly not consistent with RNA expression data.	Partly consistent with extensive gene/protein characterization data.	
**GP6**	HPA066482	Mainly not consistent with RNA expression data.	Partly consistent with extensive gene/protein characterization data.	
**GPBAR1**	HPA062890	Mainly consistent with RNA expression data.	Consistent with extensive gene/protein characterization data.	Yes
**GPC1**	HPA030571	Mainly consistent with RNA expression data.	Consistent with gene/protein characterization data.	No
**GPM6B**	HPA002913	Mainly not consistent with RNA expression data.	Consistent with extensive gene/protein characterization data.	
**GPR143**	HPA003648	Mainly consistent with RNA expression data.	Partly consistent with extensive gene/protein characterization data.	No
**GPR153**	HPA007159	Mainly consistent with RNA expression data.	No avaliable gene/protein characterization data.	
**GPR34**	CAB025490	Mainly consistent with RNA expression data.	No avaliable gene/protein characterization data.	
**GPR4**	HPA014278	Mainly not consistent with RNA expression data.	Partly consistent with extensive gene/protein characterization data.	
**GPR65**	HPA072624	Mainly consistent with RNA expression data.	Consistent with extensive gene/protein characterization data.	No
**GPR82**	HPA035914	Mainly not consistent with RNA expression data.	Partly consistent with gene/protein characterization data.	
**GPRC5D**	HPA071909	Mainly not consistent with RNA expression data.	Consistent with gene/protein characterization data.	
**GRAMD1B**	HPA008557	Mainly not consistent with RNA expression data.	No avaliable gene/protein characterization data.	
**GRIN1**	HPA067773	Mainly consistent with RNA expression data.	Consistent with extensive gene/protein characterization data.	No
**GRM2**	HPA065166	Mainly not consistent with RNA expression data.	Partly consistent with extensive gene/protein characterization data.	
**GRM6**	HPA014511	Mainly consistent with RNA expression data.	Partly consistent with extensive gene/protein characterization data.	No
**GSG1L**	HPA014479	Mainly consistent with RNA expression data.	Consistent with gene/protein characterization data.	No
**GYPC**	HPA008965	Mainly consistent with RNA expression data.	Consistent with extensive gene/protein characterization data.	No
**HAVCR1**	CAB075697	Mainly not consistent with RNA expression data.	Partly consistent with extensive gene/protein characterization data.	
**HCRTR1**	HPA014018	Mainly not consistent with RNA expression data.	Consistent with extensive gene/protein characterization data.	
**HEG1**	HPA011559	Mainly not consistent with RNA expression data.	No avaliable gene/protein characterization data.	
**HEPACAM**	CAB025486	Consistent with RNA expression data.	Consistent with extensive gene/protein characterization data.	No
**HEPH**	HPA005824	Mainly not consistent with RNA expression data.	Partly consistent with gene/protein characterization data.	
**HLA-DMB**	HPA012298	Mainly not consistent with RNA expression data.	Consistent with extensive gene/protein characterization data.	
**HLA-DOB**	HPA013846	Mainly consistent with RNA expression data.	Consistent with extensive gene/protein characterization data.	No
**HRH1**	HPA029740	Mainly not consistent with RNA expression data.	Partly consistent with extensive gene/protein characterization data.	
**HTR2B**	HPA012867	Mainly consistent with RNA expression data.	Partly consistent with extensive gene/protein characterization data.	Yes
**HTR3B**	HPA039559	Not consistent with RNA expression data.	Partly consistent with extensive gene/protein characterization data.	
**HTR4**	HPA040591	Mainly consistent with RNA expression data.	Consistent with extensive gene/protein characterization data.	No
**HTR5A**	CAB017467	Mainly not consistent with RNA expression data.	Partly consistent with extensive gene/protein characterization data.	
**HTR6**	HPA068165	Not consistent with RNA expression data.	Partly consistent with gene/protein characterization data.	
**HTR7**	HPA073617	Mainly not consistent with RNA expression data.	Consistent with extensive gene/protein characterization data.	
**ICOS**	CAB032575	Mainly not consistent with RNA expression data.	Consistent with extensive gene/protein characterization data.	

**IFNAR1**	HPA029226	Mainly not consistent with RNA expression data.	Partly consistent with extensive gene/protein characterization data.	
**IGF2R**	HPA011332	Mainly consistent with RNA expression data.	Consistent with extensive gene/protein characterization data.	No
**IL10RB**	HPA047550	Mainly consistent with RNA expression data.	Partly consistent with extensive gene/protein characterization data.	No
**IL1R1**	CAB007779	Mainly consistent with RNA expression data.	Partly consistent with extensive gene/protein characterization data.	No
**IL1R2**	HPA027597	No internal RNA expression data available for correlation.	Partly consistent with extensive gene/protein characterization data.	
**IL20RA**	CAB024990	Mainly not consistent with RNA expression data.	Partly consistent with gene/protein characterization data.	
**IL3RA**	HPA003539	Mainly not consistent with RNA expression data.	No avaliable gene/protein characterization data.	
**ILDR1**	HPA018207	Mainly not consistent with RNA expression data.	Partly consistent with gene/protein characterization data.	
**INSR**	HPA036302	Mainly consistent with RNA expression data.	Partly consistent with gene/protein characterization data.	No
**ITGA3**	HPA008572	Mainly consistent with RNA expression data.	Consistent with extensive gene/protein characterization data.	Yes
**ITGA6**	HPA012696	Mainly consistent with RNA expression data.	Consistent with extensive gene/protein characterization data.	No
**ITGB5**	HPA001820	Mainly consistent with RNA expression data.	Partly consistent with extensive gene/protein characterization data.	No
**ITGB6**	CAB073536	Mainly not consistent with RNA expression data.	Partly consistent with gene/protein characterization data.	
**JAG1**	CAB010343	Mainly consistent with RNA expression data.	Consistent with extensive gene/protein characterization data.	No
**JAM3**	HPA003417	Mainly not consistent with RNA expression data.	Partly consistent with extensive gene/protein characterization data.	
**KCNMB2**	CAB022649	Mainly not consistent with RNA expression data.	Partly consistent with gene/protein characterization data.	
**KISS1R**	HPA007156	Mainly not consistent with RNA expression data.	Partly consistent with extensive gene/protein characterization data.	
**KITLG**	HPA070395	Mainly not consistent with RNA expression data.	Partly consistent with extensive gene/protein characterization data.	
**LDLRAD3**	HPA038251	Mainly not consistent with RNA expression data.	No avaliable gene/protein characterization data.	
**LGR5**	HPA012530	Mainly not consistent with RNA expression data.	Consistent with extensive gene/protein characterization data.	
**LILRA4**	HPA049418	Mainly not consistent with RNA expression data.	Partly consistent with gene/protein characterization data.	
**LMAN2**	HPA003927	Mainly not consistent with RNA expression data.	Partly consistent with gene/protein characterization data.	
**LMBRD1**	HPA019547	Mainly consistent with RNA expression data.	No avaliable gene/protein characterization data.	
**LRFN3**	HPA048923	Mainly consistent with RNA expression data.	No avaliable gene/protein characterization data.	
**LRFN4**	HPA077570	Mainly consistent with RNA expression data.	Partly consistent with gene/protein characterization data.	No
**LRIG2**	HPA015538	Mainly consistent with RNA expression data.	Partly consistent with extensive gene/protein characterization data.	No
**LRIG3**	HPA010773	Mainly not consistent with RNA expression data.	Partly consistent with gene/protein characterization data.	
**LRP1B**	HPA069094	Mainly not consistent with RNA expression data.	Partly consistent with extensive gene/protein characterization data.	
**LRP4**	HPA011934	Mainly not consistent with RNA expression data.	Partly consistent with extensive gene/protein characterization data.	
**LRP6**	CAB004490	Mainly consistent with RNA expression data.	Partly consistent with extensive gene/protein characterization data.	No
**LRRC24**	HPA052250	Mainly consistent with RNA expression data.	No avaliable gene/protein characterization data.	
**LRRC25**	HPA029459	Mainly not consistent with RNA expression data.	Partly consistent with gene/protein characterization data.	
**LRRC3B**	HPA015568	Mainly not consistent with RNA expression data.	No avaliable gene/protein characterization data.	
**LRRN2**	HPA029124	Mainly not consistent with RNA expression data.	Partly consistent with gene/protein characterization data.	

**LRRN4CL**	HPA050317	Mainly consistent with RNA expression data.	No avaliable gene/protein characterization data.	
**LYPD5**	HPA054670	Mainly not consistent with RNA expression data.	No avaliable gene/protein characterization data.	
**MALRD1**	HPA046848	Mainly not consistent with RNA expression data.	No avaliable gene/protein characterization data.	
**MC3R**	HPA074739	Mainly not consistent with RNA expression data.	Partly consistent with gene/protein characterization data.	
**MCOLN1**	HPA031763	Mainly consistent with RNA expression data.	No avaliable gene/protein characterization data.	
**MEGF8**	HPA049248	Mainly not consistent with RNA expression data.	No avaliable gene/protein characterization data.	
**MFSD12**	HPA042149	Mainly consistent with RNA expression data.	No avaliable gene/protein characterization data.	
**MFSD5**	HPA039773	Mainly not consistent with RNA expression data.	No avaliable gene/protein characterization data.	
**MMP14**	CAB009918	Mainly consistent with RNA expression data.	Partly consistent with extensive gene/protein characterization data.	No
**MPEG1**	HPA046801	Mainly not consistent with RNA expression data.	No avaliable gene/protein characterization data.	
**MPZL1**	HPA026966	Mainly not consistent with RNA expression data.	Partly consistent with gene/protein characterization data.	
**MPZL2**	HPA060740	Mainly not consistent with RNA expression data.	No avaliable gene/protein characterization data.	
**MS4A1**	HPA014341	Consistent with RNA expression data.	Consistent with extensive gene/protein characterization data.	Yes
**MSLN**	CAB002216	Mainly consistent with RNA expression data.	Partly consistent with extensive gene/protein characterization data.	Yes
**MUC15**	HPA073304	Consistent with RNA expression data.	Consistent with extensive gene/protein characterization data.	No
**MUC16**	HPA065600	Consistent with RNA expression data.	Consistent with extensive gene/protein characterization data.	Yes
**NCAM2**	HPA030900	Mainly consistent with RNA expression data.	Partly consistent with extensive gene/protein characterization data.	No
**NECTIN4**	HPA010775	Mainly consistent with RNA expression data.	Partly consistent with extensive gene/protein characterization data.	Yes
**NEO1**	CAB009320	Mainly not consistent with RNA expression data.	Partly consistent with extensive gene/protein characterization data.	
**NETO2**	HPA013180	Mainly not consistent with RNA expression data.	Partly consistent with gene/protein characterization data.	
**NIPAL1**	HPA036765	Mainly not consistent with RNA expression data.	No avaliable gene/protein characterization data.	
**NLGN4X**	HPA001651	Mainly not consistent with RNA expression data.	Partly consistent with extensive gene/protein characterization data.	
**NLGN4Y**	HPA001651	Mainly not consistent with RNA expression data.	Partly consistent with extensive gene/protein characterization data.	
**NOTCH2**	HPA048743	Mainly consistent with RNA expression data.	Partly consistent with gene/protein characterization data.	No
**NOTCH3**	CAB005393	Mainly consistent with RNA expression data.	Partly consistent with extensive gene/protein characterization data.	No
**NPY4R**	HPA027863	Mainly not consistent with RNA expression data.	Partly consistent with extensive gene/protein characterization data.	
**NRCAM**	HPA012606	Mainly not consistent with RNA expression data.	Partly consistent with extensive gene/protein characterization data.	
**NRG1**	HPA010964	Mainly not consistent with RNA expression data.	Partly consistent with extensive gene/protein characterization data.	
**NRG2**	HPA047973	Mainly consistent with RNA expression data.	Partly consistent with gene/protein characterization data.	No
**NRP1**	HPA030278	No internal RNA expression data available for correlation.	Partly consistent with extensive gene/protein characterization data.	
**OPALIN**	HPA014372	Mainly not consistent with RNA expression data.	Consistent with extensive gene/protein characterization data.	
**OPN3**	CAB013682	Mainly not consistent with RNA expression data.	Partly consistent with extensive gene/protein characterization data.	
**OPN5**	HPA026084	Not consistent with RNA expression data.	Consistent with extensive gene/protein characterization data.	
**OSMR**	HPA017278	Mainly consistent with RNA expression data.	Consistent with extensive gene/protein characterization data.	No

**P2RX6**	HPA028777	Mainly not consistent with RNA expression data.	Partly consistent with gene/protein characterization data.	
**P2RY11**	HPA014232	Mainly not consistent with RNA expression data.	Partly consistent with gene/protein characterization data.	
**P2RY14**	CAB022646	Mainly not consistent with RNA expression data.	Partly consistent with gene/protein characterization data.	
**PCDH19**	HPA027533	Mainly not consistent with RNA expression data.	Partly consistent with extensive gene/protein characterization data.	
**PCDH7**	HPA011866	Mainly consistent with RNA expression data.	Partly consistent with extensive gene/protein characterization data.	Yes
**PCDHA12**	HPA035812	No internal RNA expression data available for correlation.	Consistent with gene/protein characterization data.	
**PCDHA5**	HPA044557	Mainly not consistent with RNA expression data.	Partly consistent with gene/protein characterization data.	
**PCDHB10**	HPA013445	Mainly not consistent with RNA expression data.	No avaliable gene/protein characterization data.	
**PCDHB5**	HPA013191	Mainly not consistent with RNA expression data.	No avaliable gene/protein characterization data.	
**PCDHB7**	HPA059271	Mainly not consistent with RNA expression data.	Partly consistent with extensive gene/protein characterization data.	
**PCDHGA11**	HPA008755	Mainly not consistent with RNA expression data.	No avaliable gene/protein characterization data.	
**PCDHGA12**	HPA008755	Mainly not consistent with RNA expression data.	No avaliable gene/protein characterization data.	
**PCDHGA2**	HPA008755	Mainly consistent with RNA expression data.	No avaliable gene/protein characterization data.	
**PCDHGA5**	HPA008755	Mainly not consistent with RNA expression data.	No avaliable gene/protein characterization data.	
**PCDHGA6**	HPA008755	Mainly not consistent with RNA expression data.	No avaliable gene/protein characterization data.	
**PCDHGB7**	HPA008755	Mainly not consistent with RNA expression data.	No avaliable gene/protein characterization data.	
**PCDHGC3**	HPA008755	Mainly not consistent with RNA expression data.	Partly consistent with gene/protein characterization data.	
**PLXNA2**	CAB009763	Mainly not consistent with RNA expression data.	Partly consistent with extensive gene/protein characterization data.	
**PQLC2**	HPA057810	Mainly not consistent with RNA expression data.	No avaliable gene/protein characterization data.	
**PRIMA1**	HPA060047	Mainly not consistent with RNA expression data.	Partly consistent with gene/protein characterization data.	
**PROM1**	HPA004922	Mainly consistent with RNA expression data.	Partly consistent with extensive gene/protein characterization data.	No
**PTGER3**	HPA010689	Mainly not consistent with RNA expression data.	Partly consistent with extensive gene/protein characterization data.	
**PTGFRN**	HPA017074	Mainly not consistent with RNA expression data.	Partly consistent with gene/protein characterization data.	
**PTH1R**	HPA007978	Not consistent with RNA expression data.	Partly consistent with extensive gene/protein characterization data.	
**PTK7**	HPA003222	Mainly not consistent with RNA expression data.	Partly consistent with extensive gene/protein characterization data.	
**PTPRJ**	HPA006026	Mainly consistent with RNA expression data.	Consistent with extensive gene/protein characterization data.	No
**PTPRN**	HPA007179	Consistent with RNA expression data.	Consistent with extensive gene/protein characterization data.	No
**PTPRN2**	HPA007255	Consistent with RNA expression data.	Consistent with extensive gene/protein characterization data.	No
**PTPRR**	CAB011461	Mainly not consistent with RNA expression data.	Partly consistent with extensive gene/protein characterization data.	
**PTPRT**	CAB069423	Mainly not consistent with RNA expression data.	Partly consistent with extensive gene/protein characterization data.	
**PTPRU**	CAB011476	Mainly not consistent with RNA expression data.	Partly consistent with extensive gene/protein characterization data.	
**PTPRZ1**	HPA015103	Mainly consistent with RNA expression data.	Consistent with extensive gene/protein characterization data.	No
**QRFPR**	HPA050236	Mainly not consistent with RNA expression data.	Partly consistent with extensive gene/protein characterization data.	
**RNF167**	HPA049810	Mainly consistent with RNA expression data.	No avaliable gene/protein characterization data.	

**ROBO1**	CAB013524	Mainly not consistent with RNA expression data.	Partly consistent with extensive gene/protein characterization data.	
**ROR2**	HPA021868	Mainly consistent with RNA expression data.	Consistent with gene/protein characterization data.	No
**RXFP1**	HPA027067	Mainly not consistent with RNA expression data.	Partly consistent with extensive gene/protein characterization data.	
**SCN3A**	HPA035396	Mainly not consistent with RNA expression data.	Consistent with gene/protein characterization data.	
**SCNN1A**	HPA012743	Mainly consistent with RNA expression data.	Partly consistent with extensive gene/protein characterization data.	No
**SDK1**	HPA011392	Mainly consistent with RNA expression data.	No avaliable gene/protein characterization data.	
**SEMA7A**	HPA042273	Mainly not consistent with RNA expression data.	Partly consistent with extensive gene/protein characterization data.	
**SEZ6L2**	HPA064471	Mainly consistent with RNA expression data.	Consistent with gene/protein characterization data.	No
**SGCA**	HPA007537	Mainly not consistent with RNA expression data.	Consistent with extensive gene/protein characterization data.	
**SIDT2**	HPA052352	Mainly not consistent with RNA expression data.	No avaliable gene/protein characterization data.	
**SIGLEC10**	CAB025807	Mainly consistent with RNA expression data.	Partly consistent with extensive gene/protein characterization data.	No
**SIGLEC8**	HPA012556	Not consistent with RNA expression data.	Partly consistent with extensive gene/protein characterization data.	
**SIRPA**	CAB002776	Mainly consistent with RNA expression data.	Consistent with extensive gene/protein characterization data.	No
**SIRPB1**	HPA047463	Mainly not consistent with RNA expression data.	Partly consistent with gene/protein characterization data.	
**SIT1**	HPA018506	Mainly consistent with RNA expression data.	Partly consistent with extensive gene/protein characterization data.	Yes
**SLAMF9**	HPA035153	Not consistent with RNA expression data.	No avaliable gene/protein characterization data.	
**SLC10A1**	HPA042727	Mainly not consistent with RNA expression data.	Consistent with gene/protein characterization data.	
**SLC10A6**	HPA016662	Mainly consistent with RNA expression data.	No avaliable gene/protein characterization data.	
**SLC11A2**	HPA032139	Mainly not consistent with RNA expression data.	Partly consistent with extensive gene/protein characterization data.	
**SLC12A6**	HPA034563	Mainly not consistent with RNA expression data.	Consistent with gene/protein characterization data.	
**SLC12A9**	HPA067536	Mainly consistent with RNA expression data.	Partly consistent with gene/protein characterization data.	No
**SLC15A1**	HPA002827	Mainly not consistent with RNA expression data.	Partly consistent with extensive gene/protein characterization data.	
**SLC15A4**	HPA016713	Mainly consistent with RNA expression data.	Partly consistent with gene/protein characterization data.	No
**SLC22A16**	HPA036902	Mainly not consistent with RNA expression data.	Partly consistent with gene/protein characterization data.	
**SLC22A17**	HPA002728	Mainly not consistent with RNA expression data.	Partly consistent with gene/protein characterization data.	
**SLC22A2**	CAB068236	Mainly consistent with RNA expression data.	Consistent with extensive gene/protein characterization data.	No
**SLC23A2**	HPA052825	Mainly not consistent with RNA expression data.	Partly consistent with extensive gene/protein characterization data.	
**SLC26A6**	HPA048363	Mainly consistent with RNA expression data.	Consistent with gene/protein characterization data.	No
**SLC2A14**	HPA006539	Mainly consistent with RNA expression data.	Consistent with extensive gene/protein characterization data.	Yes
**SLC30A1**	HPA015275	Mainly not consistent with RNA expression data.	No avaliable gene/protein characterization data.	
**SLC37A2**	HPA014948	Not consistent with RNA expression data.	No avaliable gene/protein characterization data.	
**SLC38A1**	HPA052272	Mainly not consistent with RNA expression data.	Partly consistent with extensive gene/protein characterization data.	
**SLC38A5**	HPA047411	Mainly consistent with RNA expression data.	Partly consistent with gene/protein characterization data.	No
**SLC39A10**	HPA036512	Mainly consistent with RNA expression data.	Partly consistent with gene/protein characterization data.	Yes

**SLC39A6**	HPA042377	Mainly consistent with RNA expression data.	Consistent with extensive gene/protein characterization data.	No
**SLC43A3**	HPA030551	Mainly consistent with RNA expression data.	Partly consistent with gene/protein characterization data.	No
**SLC44A3**	HPA047433	Mainly consistent with RNA expression data.	No avaliable gene/protein characterization data.	
**SLC44A4**	HPA046977	Mainly consistent with RNA expression data.	No avaliable gene/protein characterization data.	
**SLC45A4**	HPA023154	Mainly consistent with RNA expression data.	No avaliable gene/protein characterization data.	
**SLC4A5**	HPA036621	Mainly not consistent with RNA expression data.	Partly consistent with gene/protein characterization data.	
**SLC52A3**	HPA049391	Mainly consistent with RNA expression data.	Partly consistent with extensive gene/protein characterization data.	No
**SLC6A2**	HPA076311	Mainly consistent with RNA expression data.	Consistent with extensive gene/protein characterization data.	No
**SLC6A6**	HPA015028	Mainly consistent with RNA expression data.	Consistent with extensive gene/protein characterization data.	Yes
**SLC6A9**	HPA013977	Mainly not consistent with RNA expression data.	Partly consistent with gene/protein characterization data.	
**SLC7A3**	HPA003629	Mainly not consistent with RNA expression data.	Consistent with gene/protein characterization data.	
**SLC8B1**	HPA040668	Mainly not consistent with RNA expression data.	Consistent with gene/protein characterization data.	
**SLC9A2**	HPA035121	Mainly not consistent with RNA expression data.	Partly consistent with gene/protein characterization data.	
**SLC9A6**	HPA059445	Mainly consistent with RNA expression data.	Partly consistent with gene/protein characterization data.	No
**SMO**	CAB011446	Mainly consistent with RNA expression data.	Partly consistent with extensive gene/protein characterization data.	No
**SORCS1**	HPA011948	Mainly consistent with RNA expression data.	Consistent with gene/protein characterization data.	No
**SORL1**	HPA031321	No internal RNA expression data available for correlation.	Partly consistent with gene/protein characterization data.	
**SORT1**	CAB011498	Mainly consistent with RNA expression data.	Partly consistent with extensive gene/protein characterization data.	No
**SPINT2**	HPA011101	Mainly not consistent with RNA expression data.	No avaliable gene/protein characterization data.	
**SSTR2**	HPA007264	Not consistent with RNA expression data.	Partly consistent with extensive gene/protein characterization data.	
**STS**	HPA002904	Mainly consistent with RNA expression data.	Consistent with extensive gene/protein characterization data.	No
**SUSD4**	HPA058296	Mainly not consistent with RNA expression data.	No avaliable gene/protein characterization data.	
**SV2A**	CAB002226	Mainly consistent with RNA expression data.	Consistent with extensive gene/protein characterization data.	No
**SV2B**	HPA046247	Mainly not consistent with RNA expression data.	Partly consistent with gene/protein characterization data.	
**SYP**	CAB055505	Mainly consistent with RNA expression data.	Consistent with extensive gene/protein characterization data.	No
**TAS1R3**	HPA053439	Mainly not consistent with RNA expression data.	Consistent with gene/protein characterization data.	
**TECTA**	HPA018870	Mainly not consistent with RNA expression data.	Partly consistent with extensive gene/protein characterization data.	
**TGFA**	HPA042297	Mainly not consistent with RNA expression data.	No avaliable gene/protein characterization data.	
**TGFBR1**	CAB031481	Mainly not consistent with RNA expression data.	Partly consistent with extensive gene/protein characterization data.	
**THBD**	HPA002982	Mainly not consistent with RNA expression data.	Consistent with extensive gene/protein characterization data.	
**TLR3**	CAB025658	Mainly consistent with RNA expression data.	Consistent with extensive gene/protein characterization data.	No
**TMCO3**	HPA039561	Mainly consistent with RNA expression data.	No avaliable gene/protein characterization data.	
**TMED7**	HPA008960	Mainly consistent with RNA expression data.	Consistent with extensive gene/protein characterization data.	No
**TMEFF2**	HPA015587	No internal RNA expression data available for correlation.	Partly consistent with extensive gene/protein characterization data.	

**TMEM108**	HPA063350	Mainly consistent with RNA expression data.	No avaliable gene/protein characterization data.	
**TMEM154**	HPA019184	Mainly not consistent with RNA expression data.	No avaliable gene/protein characterization data.	
**TMEM161A**	HPA043365	Mainly consistent with RNA expression data.	Partly consistent with gene/protein characterization data.	No
**TMEM204**	HPA014028	Mainly not consistent with RNA expression data.	Partly consistent with gene/protein characterization data.	
**TMEM25**	HPA012163	Mainly not consistent with RNA expression data.	No avaliable gene/protein characterization data.	
**TMEM30A**	HPA014561	Mainly consistent with RNA expression data.	Partly consistent with gene/protein characterization data.	No
**TNFRSF10C**	CAB025635	Consistent with RNA expression data.	Consistent with gene/protein characterization data.	No
**TNFRSF13B**	HPA030453	Mainly not consistent with RNA expression data.	Partly consistent with gene/protein characterization data.	
**TNFRSF18**	HPA008025	No internal RNA expression data available for correlation.	Consistent with extensive gene/protein characterization data.	
**TNFRSF1A**	CAB010309	Mainly not consistent with RNA expression data.	Partly consistent with extensive gene/protein characterization data.	
**TNFRSF1B**	HPA004796	No internal RNA expression data available for correlation.	Partly consistent with extensive gene/protein characterization data.	
**TNFRSF21**	HPA006746	Mainly consistent with RNA expression data.	Partly consistent with extensive gene/protein characterization data.	No
**TNFSF13B**	CAB009188	No internal RNA expression data available for correlation.	Partly consistent with extensive gene/protein characterization data.	
**TPO**	HPA007987	Consistent with RNA expression data.	Consistent with extensive gene/protein characterization data.	No
**TRPV6**	HPA062864	Mainly consistent with RNA expression data.	Partly consistent with gene/protein characterization data.	No
**TSPAN13**	HPA007426	Mainly not consistent with RNA expression data.	Partly consistent with gene/protein characterization data.	
**TSPAN15**	HPA044657	Mainly consistent with RNA expression data.	No avaliable gene/protein characterization data.	
**TSPAN2**	HPA015640	Not consistent with RNA expression data.	Partly consistent with gene/protein characterization data.	
**TSPAN3**	HPA015996	Mainly not consistent with RNA expression data.	No avaliable gene/protein characterization data.	
**TSPAN31**	HPA057489	Mainly not consistent with RNA expression data.	No avaliable gene/protein characterization data.	
**TSPAN9**	HPA014002	Mainly consistent with RNA expression data.	No avaliable gene/protein characterization data.	
**TXNDC15**	HPA015483	Mainly consistent with RNA expression data.	No avaliable gene/protein characterization data.	
**UBAC2**	HPA013448	Mainly not consistent with RNA expression data.	No avaliable gene/protein characterization data.	
**UGT8**	HPA065785	Consistent with RNA expression data.	Consistent with gene/protein characterization data.	No
**UPK1A**	HPA049879	Mainly consistent with RNA expression data.	Consistent with gene/protein characterization data.	No
**UPK1B**	HPA031799	Mainly consistent with RNA expression data.	Consistent with gene/protein characterization data.	Yes
**UPK3B**	HPA010506	Mainly not consistent with RNA expression data.	Consistent with gene/protein characterization data.	
**VLDLR**	CAB032462	Mainly not consistent with RNA expression data.	Partly consistent with extensive gene/protein characterization data.	
**VSTM4**	HPA017279	Mainly not consistent with RNA expression data.	No avaliable gene/protein characterization data.	
**ZDHHC11B**	HPA057886	Mainly not consistent with RNA expression data.	No avaliable gene/protein characterization data.	
